# Pd-catalyzed regioselective synthesis of 3-aminoindoles *via* Larock-type annulation of *N*-protected 2-iodoanilines with ynamides

**DOI:** 10.1039/d6sc04203a

**Published:** 2026-07-07

**Authors:** Ruotong Chen, Jiayi Zhang, Junbiao Chang, Xiao-Na Wang

**Affiliations:** a Pingyuan Laboratory, State Key Laboratory of Antiviral Drugs, Key Laboratory of Advanced Drug Preparation Technologies, Ministry of Education, School of Pharmaceutical Sciences, Zhengzhou University Zhengzhou Henan 450001 P.R. China changjunbiao@zzu.edu.cn wangxn@zzu.edu.cn

## Abstract

A Pd-catalyzed Larock-type annulation of *N*-protected 2-iodoanilines with ynamides has been developed for the regioselective synthesis of 3-aminoindoles under mild conditions using commercially available PdCl_2_(PPh_3_)_2_. This concise one-pot protocol delivers a diverse array of 3-aminoindoles in good to excellent yields with broad functional-group tolerance for both coupling partners. Notably, the *N*-acetyl group on the aniline substrate plays a dual role: it directs C3 regioselectivity and acts as a masked amino surrogate, thus avoiding the need for external amination reagents. Gram-scale synthesis and post-synthetic transformations further validate the synthetic utility. This work provides a practical and regioselective platform for constructing 3-aminoindoles from readily available starting materials.

## Abstract 2

Pd-catalyzed annulation of *N*-protected 2-iodoanilines with ynamides enables regioselective 3-aminoindole synthesis. *N*-acetyl and *N*-sulfonyl direct C3-selectivity, but *N*-acetyl is uniquely effective as a dual directing and masked amino surrogate.

## Graphical abstract

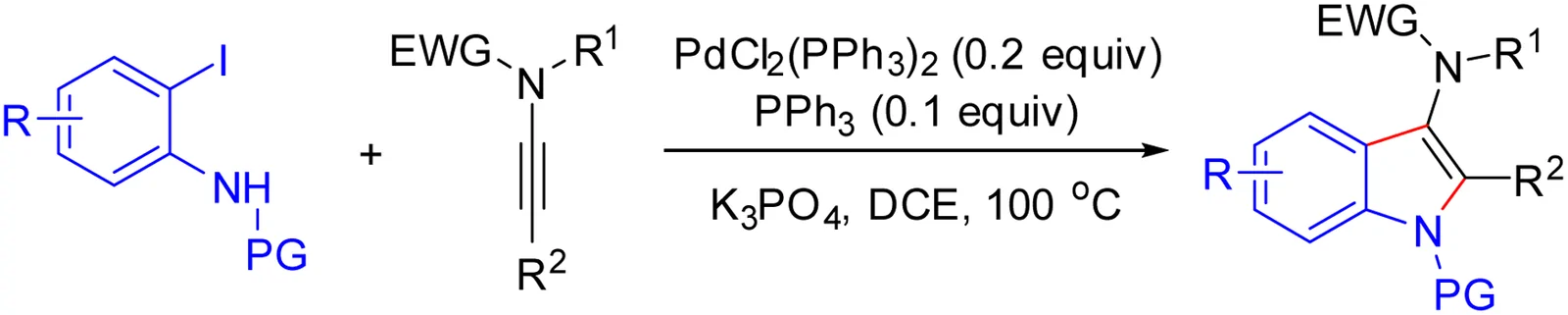

## Introduction

The 3-aminoindole motif represents a privileged scaffold in medicinal chemistry and natural product synthesis owing to its diverse bioactivities and versatile derivatization potential.^1^ Traditional methods for accessing this structure typically rely on post-functionalization of pre-formed indoles *via* nitration-reduction, C–H amination, or transition-metal-catalyzed coupling, which often require multi-step sequences, pre-activated substrates, or suffer from poor regiocontrol.^2^ Therefore, the development of a concise, catalytic, and regioselective method that directly assembles 3-aminoindoles from simple building blocks remains a significant challenge.

**Scheme 1:**
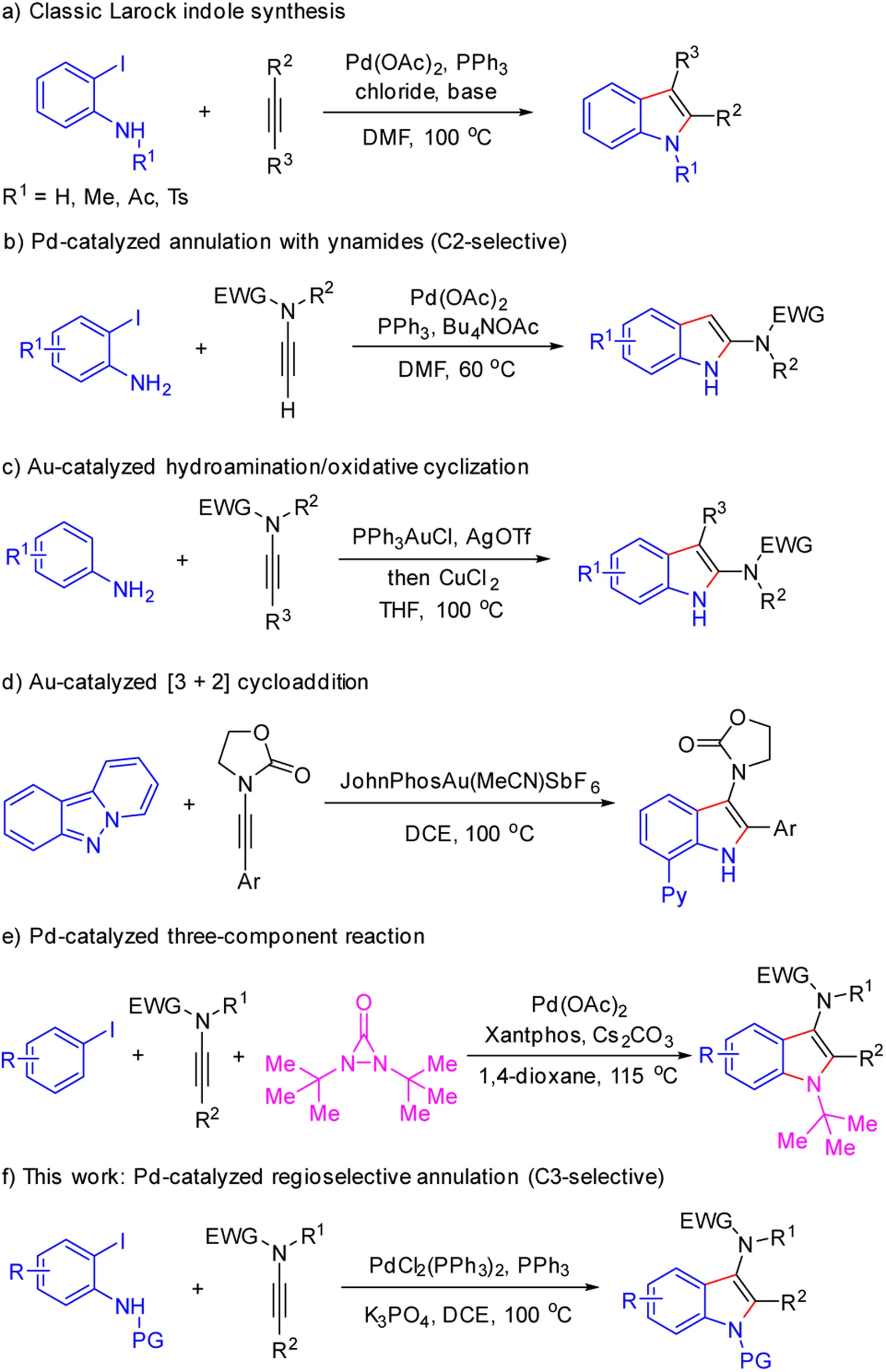
Strategies for the synthesis of 3-aminoindoles.

The classical Larock indole synthesis, a palladium-catalyzed annulation of 2-iodoanilines with internal alkynes, provides an efficient one-pot route to 2,3-disubstituted indoles (Scheme 1a).^3^ However, this paradigm offers no direct entry to aminoindoles, as installing an amino group at the C3 position demands an alkyne bearing a masked nitrogen moiety that survives reaction conditions. The emergence of ynamides as powerful building blocks, with their polarized triple bond and tunable *N*-substituent, offers a potential solution.^4^ Indeed, a palladium-catalyzed, one-pot synthesis from 2-iodoanilines and ynamides has been developed, which proceeds *via* Sonogashira coupling followed by spontaneous intramolecular hydroamination, delivering 2-amidoindoles (Scheme 1b).^5^ This result highlights a persistent regioselectivity challenge within the Larock-type annulation framework when using ynamides, favoring C2-substitution. Other catalytic approaches to aminoindoles exist but have notable limitations. A stepwise sequence involving gold-catalyzed hydroamination of anilines with ynamides, followed by a separate palladium-catalyzed cyclization or an oxidative cyclization, also affords 2-aminoindoles, and is not amenable to a practical one-pot process (Scheme 1c).^6^ While a gold-catalyzed formal [3 + 2] cycloaddition of ynamides with specialized pyrido[1,2-*b*]indazoles can deliver 3-amidoindoles, it relies on a pre-formed, complex N-heterocycle as both the nitrogen source and coupling partner, restricting substrate generality (Scheme 1d).^7^ A more modular approach is the palladium-catalyzed three-component reaction of aryl iodides, ynamides, and diaziridinones, which provides access to 3-aminoindoles. However, this method requires a specialized diaziridinone reagent as an external nitrogen source, which may reduce atom economy and increase synthetic complexity (Scheme 1e).^8*a*^ Alternatively, an electrochemical C–H amination of indoles with aniline derivatives has recently been reported, but this method affords 3-aminoindoles only when the C2 position is already substituted; otherwise, C2-amination predominates.^8*b*^

Inspired by the seminal work of Cao and Lai on related Pd-catalyzed transformations of 2-iodoanilines with ynamides,^9^ and by mechanistic insights into ynamide carbopalladation, we hypothesized that the regioselectivity of the Larock-type annulation could be intentionally switched using a traceless directing group. We proposed that an *N*-acyl or *N*-sulfonyl group on the 2-iodoaniline substrate could serve a dual role: (1) as a directing group to override the inherent C2 selectivity observed with ynamides, and (2) as a masked amino surrogate that is directly incorporated into the product. This strategy would circumvent the need for external amination reagents, specialized heterocyclic precursors, and address the regioselectivity issue of prior ynamide annulations in a single catalytic operation. Herein, we report the realization of this concept: a Pd-catalyzed, highly regioselective synthesis of 3-aminoindoles *via* Larock-type annulation of *N*-protected 2-iodoanilines with ynamides (Scheme 1f). This one-pot reaction employs commercially available PdCl_2_(PPh_3_)_2_ under mild conditions, delivering a broad range of 3-aminoindoles in good to excellent yields with broad functional-group tolerance. Based on mechanistic analogy, we propose a pathway involving oxidative addition, regioselective ynamide carbopalladation, and intramolecular C–H activation. The *N*-protecting group is pivotal, not only ensuring C3-selectivity but also offering a handle for further diversification, significantly enhancing the synthetic utility of this method.

## Results and discussion

We began by evaluating the annulation of *N*-acetyl-2-iodoaniline (1a) with *N*-Ts ynamide (2a) as model substrates. Initial experiments using Pd(OAc)_2_ in the absence of ligand and base at 25–60 °C produced no detectable product, indicating that both phosphine ligand and base are essential for catalytic activity (see SI for details). Systematic optimization of the catalyst, ligand, solvent, temperature, and catalyst loading established initial standard conditions: 1a (0.30 mmol), 2a (0.20 mmol), PdCl_2_(PPh_3_)_2_ (0.2 equiv.), PPh_3_ (0.1 equiv.), K_3_PO_4_ (2.0 equiv.), DCE (2.0 mL), 80 °C, 3.5 h, affording the desired 3-aminoindole 3aa in 94% yield (Table 1, entry 1). Control experiments further confirmed the necessity of each component: in the absence of PdCl_2_(PPh_3_)_2_ or K_3_PO_4_, no product was detected (entries 2 and 3). Omitting the additional PPh_3_ ligand significantly reduced the yield to 66% (entry 4), confirming the importance of the phosphine ligand. We next screened various bases. Replacing K_3_PO_4_ with Cs_2_CO_3_ gave 85% yield, while K_2_CO_3_ afforded 92% yield (entries 5 and 6). In contrast, Na_2_CO_3_ was completely ineffective (entry 7). Catalyst screening identified PdCl_2_(PPh_3_)_2_ as uniquely effective. Replacing it with Pd(OAc)_2_ at 80 °C afforded only 37% yield (entry 8). Other palladium sources, including Pd(dba)_2_, Pd_2_(dba)_3_, PdCl_2_(dppf), PdCl_2_(dppf)·CH_2_Cl_2_, and Pd(PPh_3_)_4_, all gave lower yields (30–57%, entries 9–13). A copper catalyst was completely ineffective (entry 14), highlighting the specificity of palladium in this transformation. Increasing the PPh_3_ loading to 0.2 equiv. did not improve the yield (85%, entry 15), whereas reducing the catalyst loading to 0.1 equiv. decreased the yield to 63% (entry 16). Solvent evaluation identified DCE as optimal. DCM, 1,4-dioxane, and THF afforded comparable yields (84–92%, entries 17–19), whereas toluene was less effective (51%, entry 20). Elevating the reaction temperature to 100 °C further improved the yield to 96% with a shorter reaction time of 2.5 h (entry 21). Accordingly, the conditions in entry 21 (100 °C, 2.5 h, 96%) were identified as optimal and used for subsequent substrate scope studies. Entry 1 served as the initial standard conditions for comparative optimization experiments. Replacing the iodo group with bromo under the optimal conditions gave the desired product in only 49% yield, confirming that the iodo group is superior due to faster oxidative addition (see SI).

**Table 1:** Optimization of reaction conditions^a^

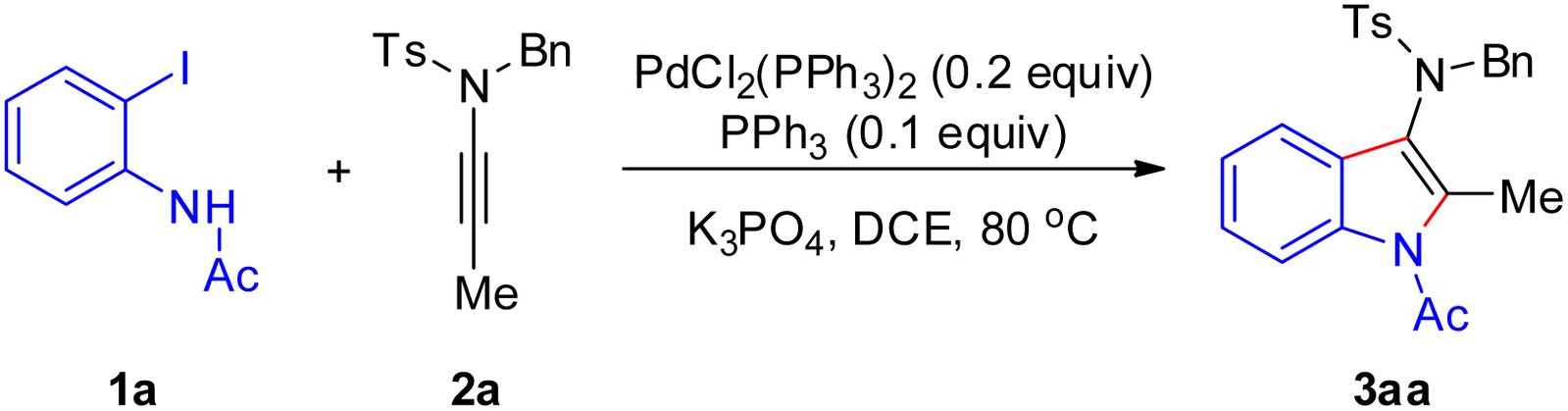		
Entry	Variation from the standard reaction conditions	Yield^b^ (%)
1	None	94
2	Without PdCl_2_(PPh_3_)_2_	0
3	Without K_3_PO_4_	0
4	Without PPh_3_	66
5	Cs_2_CO_3_ instead of K_3_PO_4_	85
6	K_2_CO_3_ instead of K_3_PO_4_	92
7	Na_2_CO_3_ instead of K_3_PO_4_	0
8	Pd(OAc)_2_ instead of PdCl_2_(PPh_3_)_2_	37
9	Pd(dba)_2_ instead of PdCl_2_(PPh_3_)_2_	55
10	Pd_2_(dba)_3_ instead of PdCl_2_(PPh_3_)_2_	31
11	PdCl_2_(dppf) instead of PdCl_2_(PPh_3_)_2_	41
12	PdCl_2_(dppf)·CH_2_Cl_2_ instead of PdCl_2_(PPh_3_)_2_	57
13	Pd(PPh_3_)_4_ instead of PdCl_2_(PPh_3_)_2_	30
14	Cu(OAc)_2_ instead of PdCl_2_(PPh_3_)_2_	NR
15	PPh_3_ (0.2 equiv.) instead of PPh_3_ (0.1 equiv.)	85
16	Catalyst loading: 0.1 equiv. instead of 0.2 equiv.	63
17	DCM instead of DCE	92
18	1,4-Dioxane instead of DCE	84
19	THF instead of DCE	88
20	Toluene instead of DCE	51
21	100 °C instead of 80 °C	96

a Standard reaction conditions: 1a (0.30 mmol), 2a (0.20 mmol), PdCl_2_(PPh_3_)_2_ (0.2 equiv., 0.04 mmol), PPh_3_ (0.1 equiv., 0.02 mmol), K_3_PO_4_ (2.0 equiv., 0.40 mmol), DCE (2.0 mL), 80 °C, 3.5 h.

b Isolated yields. NR = no reaction detected by thin layer chromatography.

With the optimal reaction conditions established (Table 1, entry 21), we next investigated the substrate scope of this Pd-catalyzed Larock-type annulation for the synthesis of 3-aminoindoles. Using *N*-acetyl-2-iodoaniline (1a) as the model substrate, we first examined the influence of the ynamide *N*-sulfonyl protecting group (Table 2). The reaction proved broadly tolerant of various sulfonyl groups. The Ts-protected ynamide delivered the desired 3-aminoindole 3aa in excellent yield (96%). Sulfonyl groups with electron-withdrawing groups on the aryl ring, such as Cs and Ns, were also well accommodated, affording the corresponding products 3ab and 3ac in 82% and 99% yields, respectively. Notably, the strongly electron-withdrawing Ns group provided the highest yield, suggesting that it may enhance the stability of key reaction intermediates through electronic effects. In contrast, the Mbs group, bearing an electron-donating methoxy substituent on the aryl ring, furnished product 3ad in a diminished yield (70%), implying that electron-rich sulfonyl groups may destabilize such intermediates. These results demonstrate that the electronic nature of the sulfonyl protecting group significantly influences reaction efficiency, with electron-deficient sulfonyl groups being particularly beneficial. We next evaluated substituents at the alkyne terminus. Ynamides bearing long-chain alkyl groups, such as *n*-propyl and *n*-hexyl, underwent smooth annulation to provide the corresponding 3-aminoindoles 3ae and 3af in near-quantitative yields, demonstrating that aliphatic substituents are well tolerated. Aryl-substituted ynamides, regardless of the nitrogen protecting group (Ts or Ns), also proved to be exceptional substrates, delivering products 3ag and 3ah in excellent yields (86–91%). Furthermore, a heteroaryl-substituted ynamide (2i) participated efficiently, affording 3-aminoindole 3ai in 94% yield and thereby extending the substrate scope to include heteroaromatic systems. Collectively, these results indicate that a wide range of alkyl, aryl, and heteroaryl substituents at the alkyne terminus are compatible with this catalytic system.

**Table 2:** Substrate scope of ynamides in the synthesis of 3-aminoindoles^a^

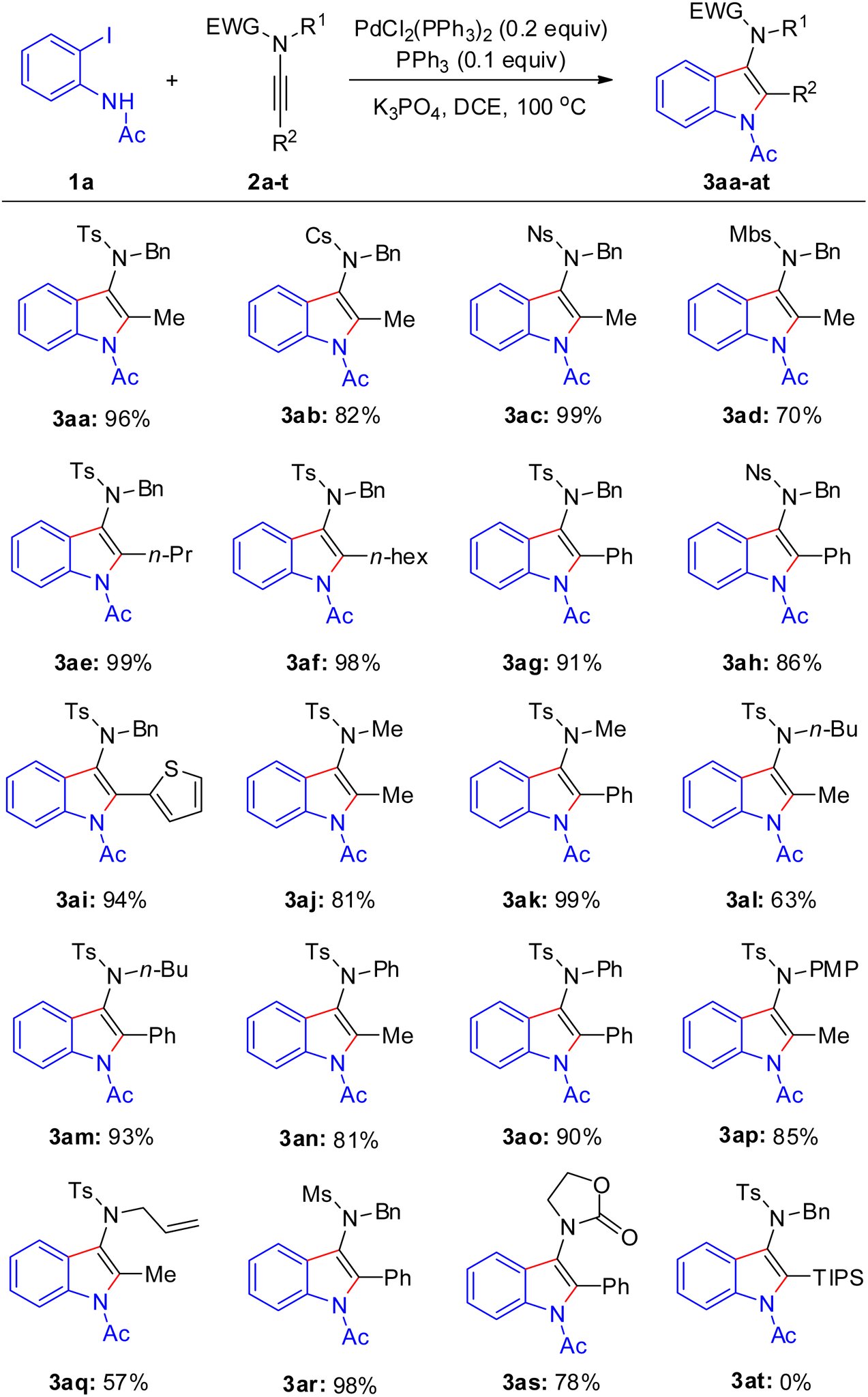

a Reaction conditions: 1a (0.30 mmol), 2a–t (0.20 mmol), PdCl_2_(PPh_3_)_2_ (0.2 equiv.), PPh_3_ (0.1 equiv.), K_3_PO_4_ (2.0 equiv.), DCE (2.0 mL), 100 °C. Cs = *p*-chlorobenzenesulfonyl; Ns = *p*-nitrobenzenesulfonyl; Mbs = *p*-methoxybenzenesulfonyl; PMP = *p*-methoxyphenyl; Ms = methylsulfonyl; TIPS = triisopropylsilyl.

We next investigated variations of the ynamide *N*-substituent. When this substituent was changed to alkyl groups (methyl or *n*-butyl), the reactions proceeded smoothly. In both cases, ynamides bearing a phenyl group at the alkyne terminus consistently delivered slightly higher yields (3aj*vs.*3ak; 3al*vs.*3am) compared to those with a methyl group, likely due to the enhanced stabilization of vinyl palladium intermediates by the conjugated phenyl moiety. Replacing the alkyl group with a phenyl substituent on the nitrogen also proved feasible, affording the C2-methyl- and C2-phenyl-substituted indoles 3an and 3ao in 81% and 90% yields, respectively. Consistent with the previous trend, the phenyl-substituted alkyne again outperformed its methyl counterpart. An ynamide bearing an *N*-PMP group (2p) reacted with 1a to deliver the desired product 3ap in 85% yield, further demonstrating the compatibility of aryl substituents on the nitrogen atom. Interestingly, when an *N*-allyl-substituted ynamide was employed, only a moderate yield of 3aq (57%) was obtained, potentially due to competitive coordination of the allyl group to the palladium catalyst. The substrate scope was further extended to ynamides bearing alkylsulfonyl groups: a Ms-substituted ynamide (2r) provided 3ar in 98% yield, and notably, an oxazolidinone-derived ynamide (2s) was also compatible, delivering the corresponding indole 3as in 78% yield. These results collectively demonstrate the excellent functional group compatibility of this catalytic system with respect to the ynamide nitrogen substituent. However, when the alkyne terminus bore a bulky triisopropylsilyl group (2t), no desired product 3at was detected, and the starting materials were recovered nearly quantitatively (98% and 97% for 1a and 2t, respectively). This outcome indicates that significant steric hindrance at the alkyne terminus completely precludes the annulation process.

**Scheme 2:**
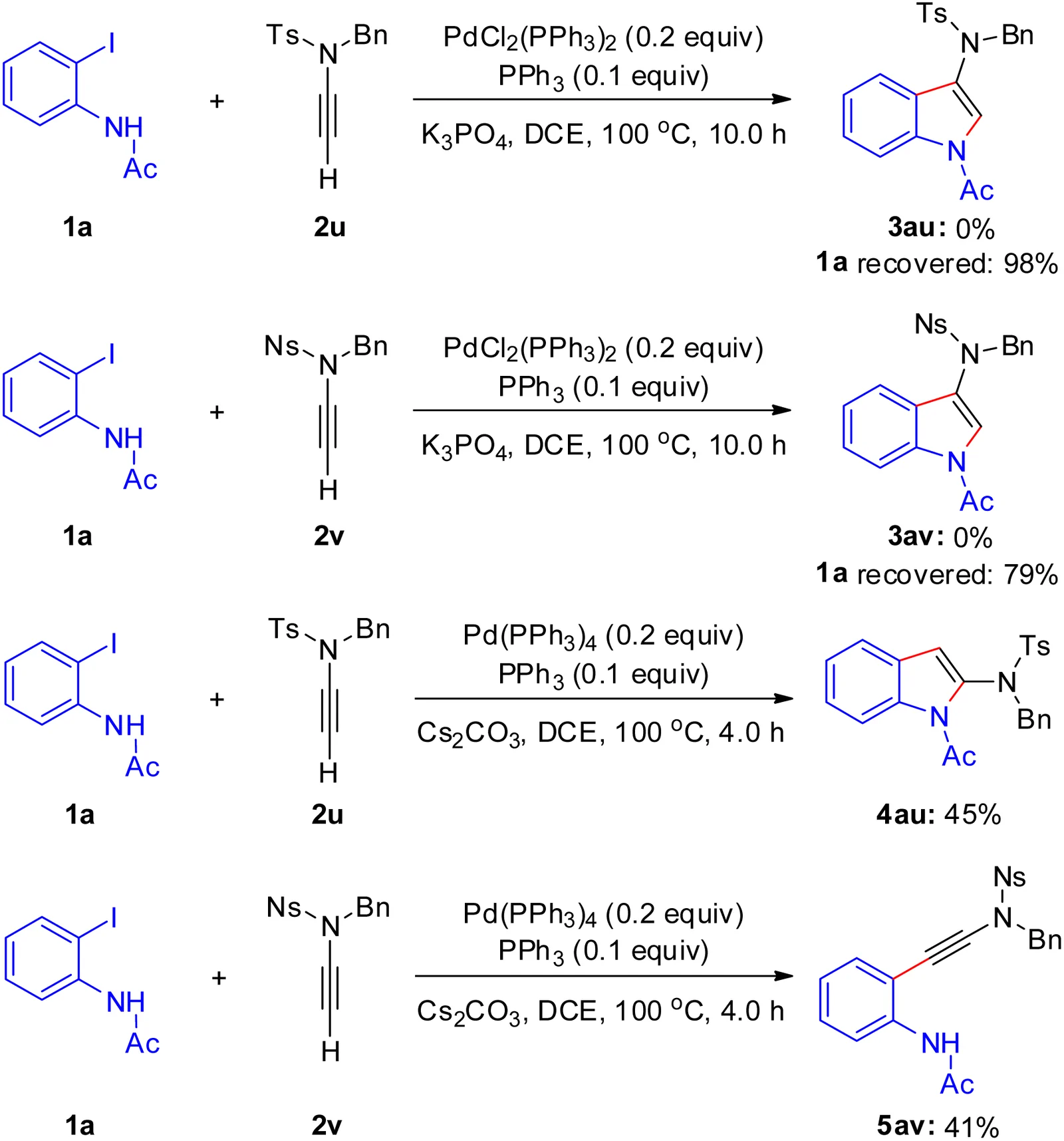
Reactions of terminal ynamides under standard and modified conditions.

We next examined terminal ynamides (2u and 2v) (Scheme 2). Under the optimal conditions, no desired products were detected, and starting material 1a was recovered in 98% and 79% yields, respectively. However, when the catalyst was switched to Pd(PPh_3_)_4_ and the base was changed from K_3_PO_4_ to Cs_2_CO_3_, the reaction of 1a with ynamide 2u afforded the 2-aminoindole product 4au in 45% yield. Notably, under these modified conditions, the Ns-protected ynamide 2v did not yield the corresponding indole; instead, the Sonogashira coupling product 5av between the ynamide and the aryl iodide was obtained. This result indicates that terminal ynamides follow a different mechanistic pathway, favoring C2-selectivity. These results highlight the unique behavior of terminal ynamides, which deviate from the standard annulation pathway and require modified reaction conditions to access alternative products.

**Fig. 1:**
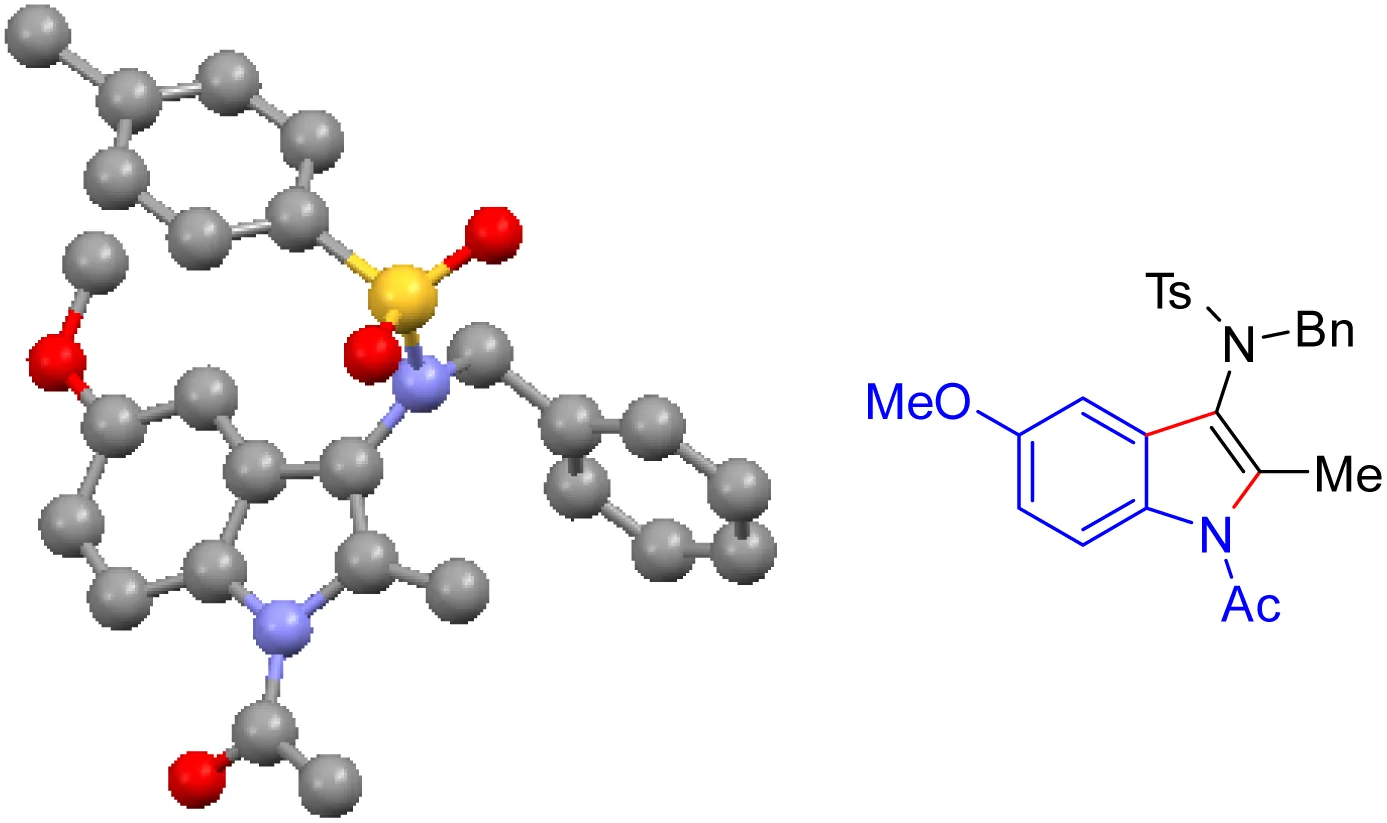
X-ray crystal structure of 3ca.

Having established the broad compatibility of various ynamides, we next examined *N*-protected 2-iodoanilines using ynamide 2a as the model substrate (Table 3). We first examined 2-iodoanilines bearing diverse substituents at the 4-position. Both electron-donating (methyl, methoxy) and electron-withdrawing groups (chloro, trifluoromethyl, cyano, nitro) were well accommodated, affording the corresponding 3-aminoindoles 3ba–3ga in good to excellent yields (75–94%). A discernible trend emerged: as the electron-withdrawing character of the substituent increased, a modest but consistent decrease in yield was observed, suggesting that electron-rich aniline derivatives facilitate the key C–H activation step. Substituents at the 5-position, including methyl, bromo, and fluoro, were also compatible, delivering products 3ha–3ja in 75–90% yields, with the same electronic trend persisting. These results demonstrate that a variety of substituents at both the 4- and 5-positions are well tolerated, with electron-donating groups generally providing higher yields. We next investigated the influence of substitution position in greater detail. A significant positional effect was observed with chloro-substituted substrates: 3-chloro substitution afforded 3ka in 70% yield, whereas 6-chloro substitution delivered 3la in a substantially higher 93% yield. This marked disparity suggests that steric hindrance *ortho* to the amino group exerts a much more pronounced deleterious effect on annulation efficiency than substitution *ortho* to the iodine atom. Interestingly, 6-fluoro substitution furnished 3ma in only 76% yield, likely reflecting the dominant electron-withdrawing effect of fluorine, which overrides its minimal steric demand. These findings underscore the critical influence of both steric and electronic factors. Finally, we varied the protecting group on the 2-iodoaniline nitrogen. Replacing the acetyl group with various arylsulfonyl moieties—bearing either electron-donating (methyl, methoxy) or electron-withdrawing (chloro, nitro) substituents on the aryl ring—afforded the corresponding 3-aminoindoles 3na–3qa in moderate yields (31–50%). These yields are substantially lower than those obtained with the *N*-acetyl protecting group, underscoring the unique role of the acyl group in facilitating the annulation process. Notably, in all these cases, the regioselectivity remained exclusively at the C3 position, and no C2-aminated products were detected. This observation supports our initial hypothesis regarding the dual function of the acyl moiety: it serves as both an effective directing group to govern regioselectivity and as a masked amino surrogate that is directly incorporated into the final product, thereby circumventing the need for external amination reagents. Control experiments further substantiate the necessity of the *N*-acetyl group: when unprotected 2-iodoaniline was subjected to the standard conditions, both starting materials were consumed within 8.0 hours, but the desired product was isolated in only 36% yield, and the reaction mixture became very messy (see SI for details), confirming that the free NH_2_ group leads to unproductive pathways and that the *N*-acetyl group is essential for achieving high efficiency and regioselectivity in this transformation. The structure of the 3-aminoindole products, as well as the regioselectivity of the annulation, was unambiguously confirmed by single-crystal X-ray diffraction analysis of compound 3ca (Fig. 1).

**Table 3:** Substrate scope of 2-iodoanilines in the synthesis of 3-aminoindoles^a^

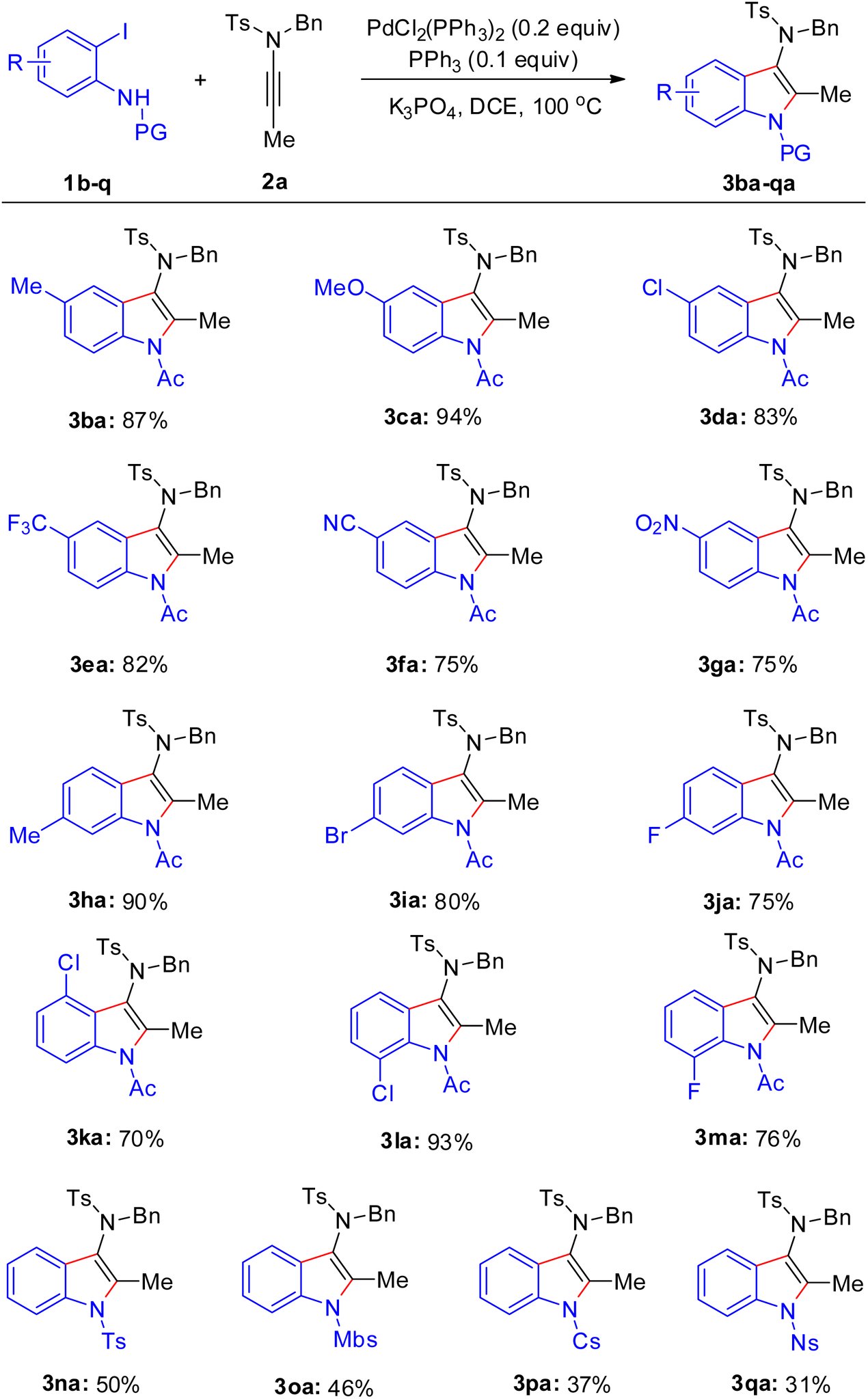

a Reaction conditions: 1b–q (0.30 mmol), 2a (0.20 mmol), PdCl_2_(PPh_3_)_2_ (0.2 equiv.), PPh_3_ (0.1 equiv.), K_3_PO_4_ (2.0 equiv.), DCE (2.0 mL), 100 °C.

**Scheme 3:**
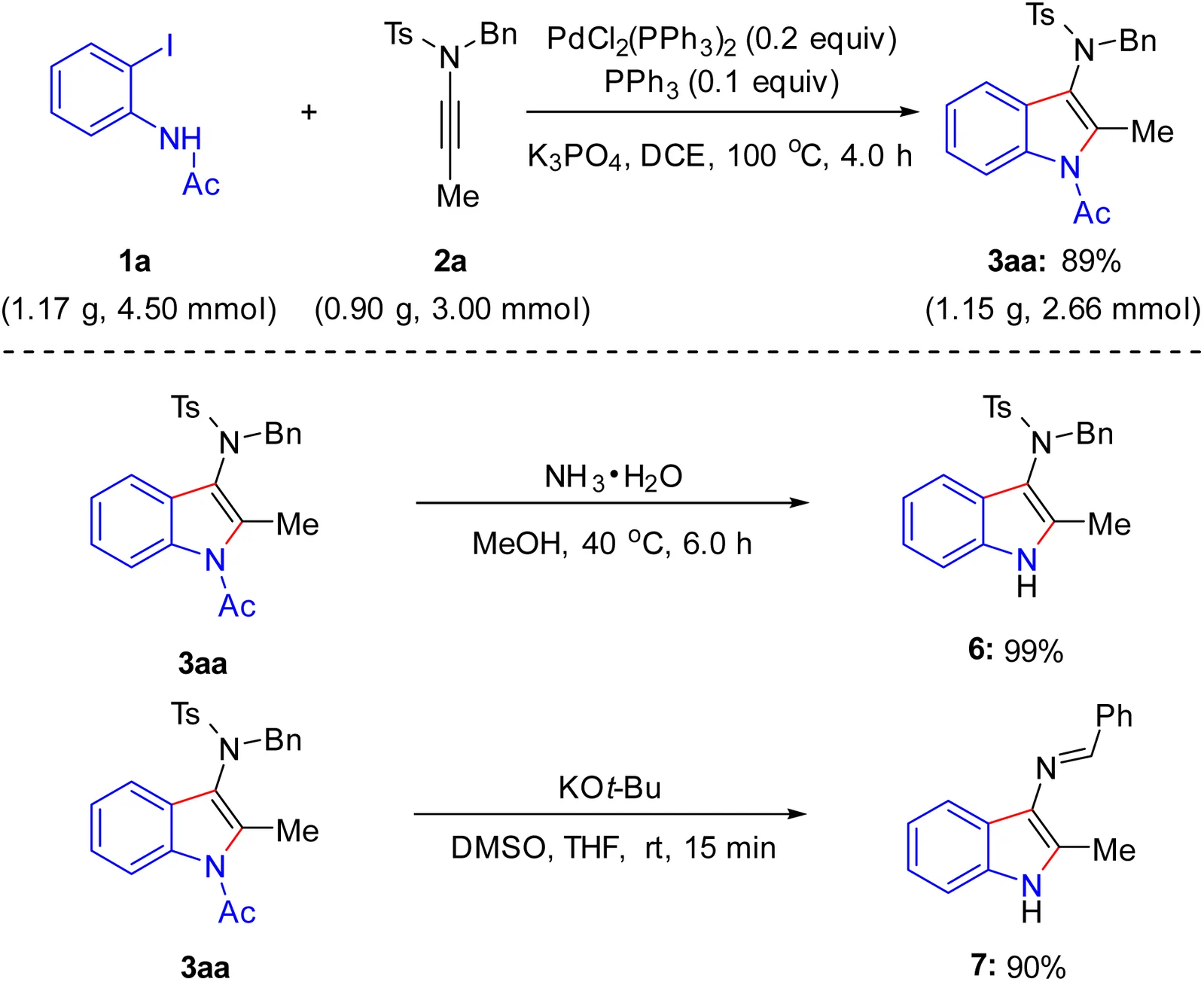
Gram-scale synthesis and product derivatization.

The gram-scale reaction of 1a (4.5 mmol) and 2a (3.0 mmol) under the optimal conditions (Scheme 3) afforded the desired 3-aminoindole 3aa in 89% isolated yield (1.15 g). This result is comparable to the 96% yield obtained on a 0.2 mmol scale, demonstrating the robustness and scalability of this protocol. We next explored synthetic transformations of 3aa to illustrate its utility. Treatment of 3aa with aqueous ammonia efficiently removed the *N*-acetyl protecting group, delivering the free NH-3-aminoindole 6 in quantitative yield.^10^ Furthermore, exposure of 3aa to KO*t*-Bu promoted sequential deprotection of both the acetyl and sulfonyl groups, accompanied by an elimination process, to afford 3-iminoindole 7 in 90% yield.^11^ These transformations demonstrate that the 3-aminoindole products serve as versatile intermediates for rapid diversification.

**Scheme 4:**
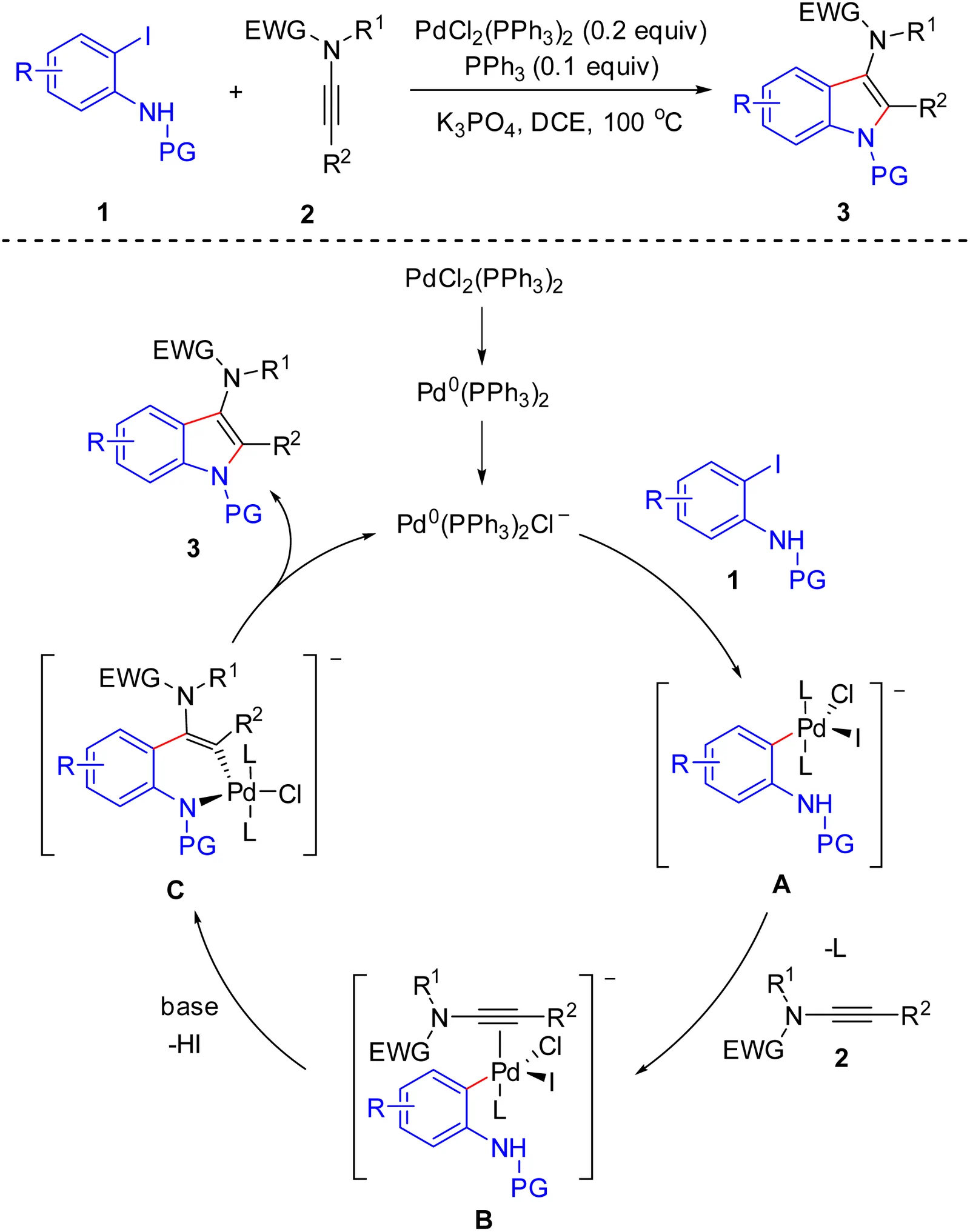
Proposed catalytic cycle.

Based on literature precedents and our experimental observations, a plausible mechanism for this Pd-catalyzed annulation is proposed in Scheme 4. The catalytic cycle is initiated by the reduction of PdCl_2_(PPh_3_)_2_ to generate the anionic Pd^0^ species [Pd^0^(PPh_3_)_2_Cl]^−^, a well-established process in palladium-catalyzed cross-couplings.^3*b*^ Oxidative addition of the aryl–iodine bond of 1 to this Pd^0^ complex affords the pentacoordinated arylpalladium(ii) intermediate A. Ligand exchange then affords the neutral *trans*-ArPdI(PPh_3_)_2_ complex, which coordinates with ynamide 2 to form intermediate B. Regioselective carbopalladation of ynamide 2 constitutes the key step determining the observed C3-selectivity.^8,12^ The *N*-protecting group on the aniline substrate is proposed to act as a directing group by coordinating to the palladium center during alkyne insertion. This chelation creates a rigid cyclic transition state that forces the ynamide to insert in an orientation placing the nitrogen-bearing acetylenic carbon in a position to bond with the aryl group. Following this regioselective alkyne insertion, the resulting alkenylpalladium intermediate undergoes nitrogen displacement of the halide—the nitrogen atom of the aniline attacks the palladium center, displacing the iodide ligand and forming a six-membered, heteroatom-containing palladacycle intermediate C. Reductive elimination from this palladacycle then occurs with concomitant formation of the C–N bond, delivering the 3-aminoindole product 3 and regenerating the Pd^0^ species to re-enter the catalytic cycle. The nitrogen atom originally from the ynamide substituent is thus incorporated at the C3 position, consistent with the observed regioselectivity.

## Conclusion

In summary, we developed a Pd-catalyzed regioselective synthesis of 3-aminoindoles *via* Larock-type annulation of *N*-protected 2-iodoanilines with ynamides. This method provides a concise one-pot approach to a diverse array of 3-aminoindoles under mild conditions using commercially available PdCl_2_(PPh_3_)_2_ as the catalyst, exhibiting broad substrate scope with respect to both coupling partners and delivering the desired products in good to excellent yields. The *N*-protecting group on the aniline substrate functions both as a regiochemical controller and as an intrinsic nitrogen source, obviating external amination reagents. The synthetic utility of this protocol was further demonstrated by gram-scale synthesis and diverse post-synthetic transformations, including deprotection and elimination. Based on literature precedents, a plausible catalytic pathway is proposed involving oxidative addition, regioselective carbopalladation directed by the *N*-protecting group, nitrogen displacement of the halide to form a six-membered palladacycle, and reductive elimination. This work provides a practical, scalable, and regioselective platform for constructing 3-aminoindoles from readily available starting materials, offering a valuable tool for medicinal chemistry and heterocyclic synthesis.

## Author contributions

Ruotong Chen performed the experiments and analyzed the results. Jiayi Zhang assisted in performing the experiments. Junbiao Chang and Xiao-Na Wang supervised and directed the project. Xiao-Na Wang conceptualized the project and wrote the manuscript.

## Conflicts of interest

There are no conflicts to declare.

## Supplementary Material

SC-017-D6SC04203A-s001

SC-017-D6SC04203A-s002

## Data Availability

The data supporting this article have been included as part of the supplementary information (SI). Supplementary information: further details of the experimental procedures, ^1^H NMR, ^13^C NMR, and ^19^F NMR spectra, HRMS data, and X-ray crystallographic data for 3ca. See DOI: https://doi.org/10.1039/d6sc04203a. CCDC 2554809 contains the supplementary crystallographic data for this paper.^13^

## References

[cit1] Haag S. M., Gulen M. F., Reymond L., Gibelin A., Abrami L., Decout A., Heymann M., van der Goot F. G., Turcatti G., Behrendt R., Ablasser A. (2018). Targeting STING with covalent small-molecule inhibitors. Nature.

[cit2] Bahekar R. H., Jain M. R., Goel A., Patel D. N., Prajapati V. M., Gupta A. A., Jadav P. A., Patel P. R. (2007). Design, synthesis, and biological evaluation of substituted-*N*-(thieno[2,3-*b*]pyridin-3-yl)-guanidines, *N*-(1*H*-pyrrolo[2,3-*b*]pyridin-3-yl)-guanidines, and *N*-(1*H*-indol-3-yl)-guanidines. Bioorg. Med. Chem..

[cit3] Larock R. C., Yum E. K. (1991). Synthesis of indoles *via* palladium-catalyzed heteroannulation of internal alkynes. J. Am. Chem. Soc..

[cit4] Pradhan T. R., Park J. K. (2025). Intermediate control: unlocking hitherto unknown reactivity and selectivity in *N*-conjugated allenes and alkynes. Acc. Chem. Res..

[cit5] Dooleweerdt K., Ruhland T., Skrydstrup T. (2009). Application of ynamides in the synthesis of 2-amidoindoles. Org. Lett..

[cit6] Kim Y. H., Yoo H. J., Youn S. W. (2020). Facile one-pot synthesis of 2-aminoindoles from simple anilines and ynamides. Chem. Commun..

[cit7] Yu Y., Chen G., Zhu L., Liao Y., Wu Y., Huang X. (2016). Gold-catalyzed β-regioselective formal [3 + 2] cycloaddition of ynamides with pyrido[1,2-*b*]indazoles: reaction development and mechanistic insights. J. Org. Chem..

[cit8] Wang J., Liu W., Liu J., Hong H., Shi Y., Yang E., Shi Y. (2025). Pd-Catalyzed regioselective tandem Heck/C–H activation/bisamination reactions with ynamides and diaziridinone. Rapid access to 3-aminoindoles. Org. Lett..

[cit9] Cao J., Xu Y., Kong Y., Cui Y., Hu Z., Wang G., Deng Y., Lai G. (2012). Synthesis of *δ*-carbolines *via* a Pd-catalyzed sequential reaction from 2-iodoanilines and *N*-tosyl-enynamines. Org. Lett..

[cit10] Han Q., Xu K., Tian F., Huang S., Zeng C. (2022). A practical transamidation strategy for the N-deacylation of amides. Chin. J. Org. Chem..

[cit11] Jiao L., Wang Y., Ding L., Zhang C., Wang X.-N., Chang J. (2022). Synthesis of 2-aminopyrroles *via* metal-free annulation of ynamides with 2*H*-azirines. J. Org. Chem..

[cit12] Campbell C. D., Greenaway R. L., Holton O. T., Walker P. R., Chapman H. A., Russell C. A., Carr G., Thomson A. L., Anderson E. A. (2015). Ynamide carbopalladation: A flexible route to mono-, bi- and tricyclic azacycles. Chem. Eur. J..

[cit13] CCDC 2554809: Experimental Crystal Structure Determination, 2026, 10.5517/ccdc.csd.cc2rrh67

