# Supplementary material for: Pd-catalyzed regioselective synthesis of 3-aminoindoles via Larock-type annulation of N-protected 2-iodoanilines with ynamides
Source: Chem Sci. 2026 Jul 7. Online ahead of print. doi: 10.1039/d6sc04203a (PMC13359594; doi:10.1039/d6sc04203a)
Supplement: SC-OLF-D6SC04203A-s001 [file SC-OLF-D6SC04203A-s001.pdf]

Supplementary Information (SI) for Chemical Science.
This journal is © The Royal Society of Chemistry 2026

SUPPORTING INFORMATION

Pd-Catalyzed Regioselective Synthesis of 3-Aminoindoles via Larock-Type Annulation of *N*-Protected 2-Iodoanilines with Ynamides

Ruotong Chen, Jiayi Zhang, Junbiao Chang,* and Xiao-Na Wang*

Pingyuan Laboratory, State Key Laboratory of Antiviral Drugs, Key Laboratory of Advanced Drug Preparation Technologies, Ministry of Education, School of Pharmaceutical Sciences, Zhengzhou University, Zhengzhou, Henan 450001, P.R. China

Table of Contents

Part I Experimental Part S2

General Information S2

1.1 Optimization of 3-Aminoindoles Synthesis Conditions S2

1.2 Synthesis of 3-Aminoindoles S4

1.3 Chemical Transformations of the Annulation Products S26

1.4 X-Ray Crystal Structures of **3ca** S27

1.5 Control Experiments S28

1.6 Failed Reactions S29

References S29

Part II Copies of ^{1}H NMR, ^{13}C NMR, and ^{19}F NMR Spectra S32

Part I Experimental Part

General Information

Unless otherwise indicated, all starting materials were obtained from commercial supplies and used as received. Ynamides and *N*-protected-2-iodoanilines were synthesized according to literature procedures, and their analytical data were consistent with reported values. All reactions were performed in oven-dried glassware under a nitrogen atmosphere unless otherwise specified. All catalysts were handled and weighed in a glove box. Solvents were dried and distilled prior to use. Chromatographic separations were performed using 200~300 mesh silica gel. ^{1}H NMR and ^{13}C NMR spectra were obtained on a Bruker Ascend^{TM} 400 NMR spectrometer using $CDCl_3$ as the solvent and the residual solvent peak as the internal standard. ^{13}C NMR (100 MHz) spectra were reported in ppm, with the internal chloroform signal at 77.2 ppm as the reference. TLC analysis was performed on 254 nm polyester-backed plates and visualized using UV light and $KMnO_4$ stain. High-resolution mass spectra (HRMS) were recorded on a Bruker MicrOTOF-Q II mass spectrometer.

1.1 Optimization of 3-Aminoindoles Synthesis Conditions

Entry 26 (Gram-Scale Synthesis of 3aa): To an oven-dried tube was added *N*-acetyl-2-iodoaniline **1a**[1] (1.2 g, 4.50 mmol), ynamide **2a**[2] (898.2 mg, 3.00 mmol), $PdCl_2(PPh_3)_2$ (421.1 mg, 0.60 mmol), PPh_3 (78.7 mg, 0.30 mmol), K_3PO_4 (1.3 g, 6.00 mmol), and DCE (30.0 mL, ynamide *concn* = 0.10 *M*). The tube was capped and stirred in an oil bath at 100 °C for 4.0 h. After the reaction was judged to be complete by TLC analysis, the reaction mixture was cooled to room temperature and filtered through a short pad of silica gel. The filtrate was concentrated under reduced pressure, and the residue was purified by flash column chromatography on silica gel (gradient eluent: 15:1~5:1 petroleum ether/EtOAc) to afford **3aa** (1.2 g, 2.66 mmol) in 89% yield.

Table S1 Optimization of Reaction Conditions

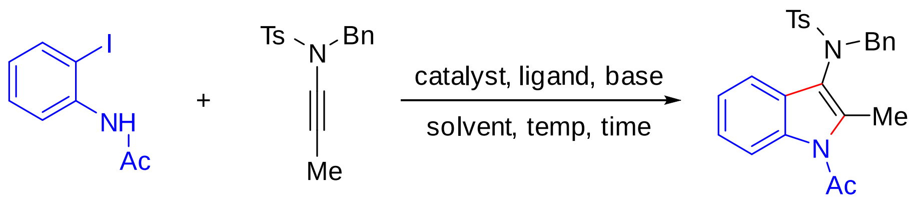

1a **2a** **3aa**

Entry	Catalyst	Ligand	Base	Solvent	Temp (°C)	Time (h)	Yield (%)[b]
1	$Pd(OAc)_2$	-	-	DCE	60	10	NR
2	$Pd(OAc)_2$	PPh_3	K_3PO_4	DCE	25	10	NR
3	$Pd(OAc)_2$	PPh_3	K_3PO_4	DCE	60	12	18
4	$Pd(OAc)_2$	PPh_3	K_3PO_4	DCE	80	4	37
5	$Pd(OAc)_2$	-	K_3PO_4	DCE	80	4	21
6	-	PPh_3	K_3PO_4	DCE	80	6.0	NR
7	$Pd(dba)_2$	PPh_3	K_3PO_4	DCE	80	10	55
8	$Pd_2(dba)_3$	PPh_3	K_3PO_4	DCE	80	10	31
9	$PdCl_2(dppf)$	PPh_3	K_3PO_4	DCE	80	10	41
10	$PdCl_2(dppf)\ CH_2Cl_2$	PPh_3	K_3PO_4	DCE	80	10	57
11	$Pd(PPh_3)_4$	PPh_3	K_3PO_4	DCE	80	10	30
12	$Cu(OAc)_2$	PPh_3	K_3PO_4	DCE	80	8	NR
13	$PdCl_2(PPh_3)_2$	PPh_3	K_3PO_4	DCE	80	3.5	94
14	$PdCl_2(PPh_3)_2$	-	K_3PO_4	DCE	80	3.5	66
15	$PdCl_2(PPh_3)_2$	PPh_3	-	DCE	80	6.0	NR
16	$PdCl_2(PPh_3)_2$	PPh_3	Cs_2CO_3	DCE	80	12.0	85
17	$PdCl_2(PPh_3)_2$	PPh_3	K_2CO_3	DCE	80	13.0	92
18	$PdCl_2(PPh_3)_2$	PPh_3	Na_2CO_3	DCE	80	11.0	0
19[c]	$PdCl_2(PPh_3)_2$	PPh_3	K_3PO_4	DCE	80	6	63
20[d]	$PdCl_2(PPh_3)_2$	PPh_3	K_3PO_4	DCE	80	3	85
21	$PdCl_2(PPh_3)_2$	PPh_3	K_3PO_4	DCM	80	5	92
22	$PdCl_2(PPh_3)_2$	PPh_3	K_3PO_4	1,4-dioxane	80	4.5	84
23	$PdCl_2(PPh_3)_2$	PPh_3	K_3PO_4	THF	80	4	88
24	$PdCl_2(PPh_3)_2$	PPh_3	K_3PO_4	toluene	80	4	51
25	$PdCl_2(PPh_3)_2$	PPh_3	K_3PO_4	DCE	100	2.5	96
26[e]	$PdCl_2(PPh_3)_2$	PPh_3	K_3PO_4	DCE	100	4.0	89

[a]Unless otherwise specified, reactions were carried out using **1a** (0.30 mmol), **2a** (0.20 mmol), catalyst (0.2 equiv, 0.04 mmol), base (2.0 equiv, 0.4 mmol), ligand (0.1 equiv, 0.02 mmol), and catalyst in solvent (2.0 mL). [b]Isolated yields. [c]Catalyst (0.1 equiv, 0.02 mmol) was added. [d]Ligand (0.2 equiv, 0.04 mmol) was added. [e]**1a** (4.50 mmol) and **2a** (3.00 mmol) were added.

1.2 Synthesis of 3-Aminoindoles

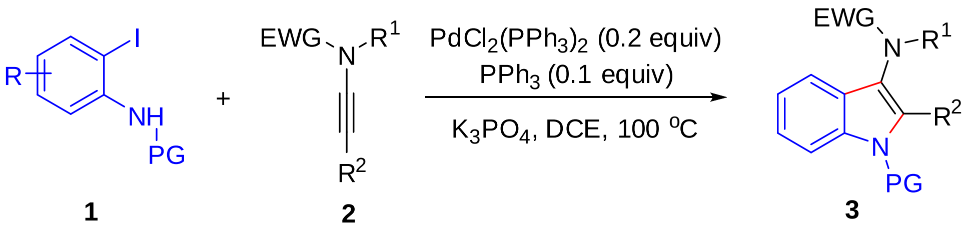

To an oven-dried tube was added *N*-acetyl-2-iodoaniline **1a** (78.3 mg, 0.30 mmol), ynamide **2a** (59.9 mg, 0.20 mmol), $PdCl_2(PPh_3)_2$ (28.1 mg, 0.04 mmol), PPh_3 (5.3 mg, 0.02 mmol), K_3PO_4 (84.9 mg, 0.40 mmol), and DCE (2.0 mL, ynamide *concn* = 0.10 *M*). The tube was capped and stirred in an oil bath at 100 ℃ for 2.5 h. After the reaction was judged to be complete by TLC analysis, the reaction mixture was cooled to room temperature and filtered through a short pad of silica gel. The filtrate was concentrated under reduced pressure, and the residue was purified by flash column chromatography on silica gel (gradient eluent: 15:1~5:1 petroleum ether/EtOAc) to afford **3aa** (83.3 mg, 0.19 mmol) in 96% yield.

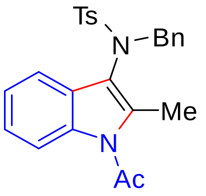

3aa

3aa: R_f = 0.32 [5:1 petroleum ether/EtOAc]; white solid; mp = 108-109 °C; ^{1}H NMR (400 MHz, $CDCl_3$) δ 8.07 (d, 1H, J = 8.4 Hz), 7.66 (d, 2H, J = 8.3 Hz), 7.26-7.12 (m, 8H), 6.95 (t, 1H, J = 7.1 Hz), 6.49 (d, 1H, J = 7.9 Hz), 5.14, 4.34 (ABq, 2H, J_{AB} = 13.6 Hz), 2.61 (s, 3H), 2.44 (s, 3H), 2.10 (s, 3H); ^{13}C NMR (101 MHz, $CDCl_3$) δ 170.5, 143.9, 138.5, 137.5, 136.0, 134.8, 129.9, 129.4, 128.4, 128.1, 127.7, 125.8, 124.3, 123.1, 118.9, 117.9, 115.9, 53.9, 27.6, 21.7, 14.1; HRMS (ESI): m/z calcd for $C_{25}H_{25}N_2O_3S$ $[M + H]^+$ 433.1580, found 433.1588.

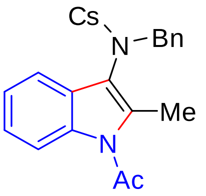

3ab

To an oven-dried tube was added *N*-acetyl-2-iodoaniline **1a** (78.3 mg, 0.30 mmol), ynamide **2b**[3] (64.0 mg, 0.20 mmol), $PdCl_2(PPh_3)_2$ (28.1 mg, 0.04 mmol), PPh_3 (5.3 mg, 0.02 mmol), K_3PO_4 (84.9 mg, 0.40 mmol), and DCE (2.0 mL, ynamide *concn* = 0.10 *M*). The tube was capped and stirred in an oil bath at 100 ℃ for 4.0 h. After the reaction was judged to be complete by TLC analysis, the

reaction mixture was cooled to room temperature and filtered through a short pad of silica gel. The filtrate was concentrated under reduced pressure, and the residue was purified by flash column chromatography on silica gel (gradient eluent: 15:1~5:1 petroleum ether/EtOAc) to afford **3ab** (73.9 mg, 0.16 mmol) in 82% yield.

3ab: R_f = 0.37 [5:1 petroleum ether/EtOAc]; white solid; mp = 156-157 °C; ^{1}H NMR (400 MHz, $CDCl_3$) δ 8.09 (d, 1H, J = 8.5 Hz), 7.71 (d, 2H, J = 8.6 Hz), 7.43 (d, 2H, J = 8.6 Hz), 7.25-7.14 (m, 6H), 7.00 (t, 1H, J = 7.5 Hz), 6.46 (d, 1H, J = 7.9 Hz), 5.16, 4.35 (ABq, 2H, J_{AB} = 13.5 Hz), 2.63 (s, 3H), 2.10 (s, 3H); ^{13}C NMR (101 MHz, $CDCl_3$) δ 170.5, 139.6, 138.9, 138.8, 135.7, 134.8, 129.6, 129.5, 129.1, 128.6, 128.3, 125.4, 124.5, 123.3, 118.3, 117.5, 116.1, 54.1, 27.7, 14.2; HRMS (ESI): m/z calcd for $C_{24}H_{22}ClN_2O_3S$ $[M + H]^+$ 453.1034, found 453.1040.

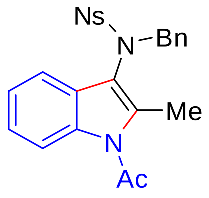

3ac

To an oven-dried tube was added *N*-acetyl-2-iodoaniline **1a** (78.3 mg, 0.30 mmol), ynamide **2c**[4] (78.5 mg, 0.20 mmol), $PdCl_2(PPh_3)_2$ (28.1 mg, 0.04 mmol), PPh_3 (5.3 mg, 0.02 mmol), K_3PO_4 (84.9 mg, 0.40 mmol), and DCE (2.0 mL, ynamide *concn* = 0.10 *M*). The tube was capped and stirred in an oil bath at 100 °C for 5.5 h. After the reaction was judged to be complete by TLC analysis, the reaction mixture was cooled to room temperature and filtered through a short pad of silica gel. The filtrate was concentrated under reduced pressure, and the residue was purified by flash column chromatography on silica gel (gradient eluent: 15:1~5:1 petroleum ether/EtOAc) to afford **3ac** (92.0 mg, 0.19 mmol) in 99% yield.

3ac: R_f = 0.32 [5:1 petroleum ether/EtOAc]; white solid; mp = 148-149 °C; ^{1}H NMR (400 MHz, $CDCl_3$) δ 8.28 (d, 2H, J = 8.8 Hz), 8.08 (d, 1H, J = 8.5 Hz), 7.95 (d, 2H, J = 8.8 Hz), 7.26-7.20 (m, 4H), 7.16-7.15 (m, 2H), 6.95 (t, 1H, J = 7.5 Hz), 6.39 (d, 1H, J = 7.8 Hz), 5.20, 4.39 (ABq, 2H, J_{AB} = 13.6 Hz), 2.65 (s, 3H), 2.10 (s, 3H); ^{13}C NMR (101 MHz, $CDCl_3$) δ 170.4, 150.3, 146.3, 139.0, 135.3, 134.8, 129.6, 129.0, 128.7, 128.6, 125.2, 124.8, 124.5, 123.4, 117.8, 117.0, 116.2, 54.5, 27.7, 14.2; HRMS (ESI): m/z calcd for $C_{24}H_{21}N_3NaO_5S$ $[M + Na]^+$ 486.1094, found 486.1100.

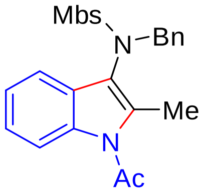

3ad

To an oven-dried tube was added *N*-acetyl-2-iodoaniline **1a** (78.3 mg, 0.30 mmol), ynamide **2d**[5] (63.1 mg, 0.20 mmol), $PdCl_2(PPh_3)_2$ (28.1 mg, 0.04 mmol), PPh_3 (5.3 mg, 0.02 mmol), K_3PO_4 (84.9 mg, 0.40 mmol), and DCE (2.0 mL, ynamide *concn* = 0.10 *M*). The tube was capped and stirred in an oil bath at 100 °C for 4.0 h. After the reaction was judged to be complete by TLC analysis, the reaction mixture was cooled to room temperature and filtered through a short pad of silica gel. The filtrate was concentrated under reduced pressure, and the residue was purified by flash column chromatography on silica gel (gradient eluent: 15:1~5:1 petroleum ether/EtOAc) to afford **3ad** (62.7 mg, 0.14 mmol) in 70% yield, and the starting material **1a** was recovered in 37% yield.

3ad: R_f = 0.28 [5:1 petroleum ether/EtOAc]; white solid; mp = 118-119 °C; ^{1}H NMR (400 MHz, $CDCl_3$) δ 8.09 (d, 1H, J = 8.4 Hz), 7.70 (d, 2H, J = 8.9 Hz), 7.21-7.14 (m, 6H), 6.99 (t, 1H, J = 7.5 Hz), 6.91 (d, 2H, J = 8.8 Hz), 6.53 (d, 1H, J = 7.8 Hz), 5.14, 4.34 (ABq, 2H, J_{AB} = 13.6 Hz), 3.87 (s, 3H), 2.62 (s, 3H), 2.11 (s, 3H); ^{13}C NMR (101 MHz, $CDCl_3$) δ 170.5, 163.3, 138.6, 136.1, 134.9, 132.1, 129.8, 129.4, 128.5, 128.1, 125.8, 124.4, 123.2, 118.9, 117.9, 116.0, 114.4, 55.9, 53.9, 27.7, 14.2; HRMS (ESI): m/z calcd for $C_{25}H_{25}N_2O_4S$ $[M + H]^+$ 449.1530, found 449.1535.

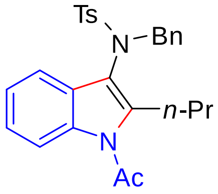

3ae

To an oven-dried tube was added *N*-acetyl-2-iodoaniline **1a** (78.3 mg, 0.30 mmol), ynamide **2e**[6] (65.5 mg, 0.20 mmol), $PdCl_2(PPh_3)_2$ (28.1 mg, 0.04 mmol), PPh_3 (5.3 mg, 0.02 mmol), K_3PO_4 (84.9 mg, 0.40 mmol), and DCE (2.0 mL, ynamide *concn* = 0.10 *M*). The tube was capped and stirred in an oil bath at 100 °C for 4.5 h. After the reaction was judged to be complete by TLC analysis, the reaction mixture was cooled to room temperature and filtered through a short pad of silica gel. The filtrate was concentrated under reduced pressure, and the residue was purified by flash column chromatography on silica gel (gradient eluent: 15:1~5:1 petroleum ether/EtOAc) to afford **3ae** (91.6 mg, 0.19 mmol) in 99% yield.

3ae: R_f = 0.40 [5:1 petroleum ether/EtOAc]; white solid; mp = 158-159 °C; ^{1}H NMR (400 MHz, $CDCl_3$) δ 7.86 (d, 1H, J = 8.4 Hz), 7.67 (d, 2H, J = 8.2 Hz), 7.26-7.16 (m, 8H), 6.93 (t, 1H, J = 7.5

Hz), 6.45 (d, 1H, J = 7.8 Hz), 5.16, 4.36 (ABq, 2H, J_{AB} = 13.7 Hz), 2.70-2.57 (m, 5H), 2.45 (s, 3H), 1.29-1.20 (m, 1H), 0.73 (t, 3H, J = 7.3 Hz), 0.37- 0.28 (m, 1H); ^{13}C NMR (101 MHz, $CDCl_3$) δ 170.4, 143.9, 143.6, 137.5, 136.3, 134.9, 129.9, 129.5, 128.5, 128.1, 127.9, 125.6, 124.1, 122.8, 118.4, 118.2, 115.1, 54.0, 28.8, 27.4, 22.8, 21.8, 14.6; HRMS (ESI): m/z calcd for $C_{27}H_{29}N_2O_3S$ $[M + H]^+$ 461.1893, found 461.1901.

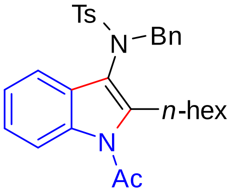

3af

To an oven-dried tube was added *N*-acetyl-2-iodoaniline **1a** (78.3 mg, 0.30 mmol), ynamide **2f**[2] (76.7 mg, 0.20 mmol), $PdCl_2(PPh_3)_2$ (28.1 mg, 0.04 mmol), PPh_3 (5.3 mg, 0.02 mmol), K_3PO_4 (84.9 mg, 0.40 mmol), and DCE (2.0 mL, ynamide *concn* = 0.10 *M*). The tube was capped and stirred in an oil bath at 100 °C for 3.0 h. After the reaction was judged to be complete by TLC analysis, the reaction mixture was cooled to room temperature and filtered through a short pad of silica gel. The filtrate was concentrated under reduced pressure, and the residue was purified by flash column chromatography on silica gel (gradient eluent: 15:1~5:1 petroleum ether/EtOAc) to afford **3af** (102.2 mg, 0.19 mmol) in 98% yield.

3af: R_f = 0.42 [5:1 petroleum ether/EtOAc]; white solid; mp = 133-134 °C; ^{1}H NMR (400 MHz, $CDCl_3$) δ 7.86 (d, 1H, J = 8.4 Hz), 7.67 (d, 2H, J = 8.2 Hz), 7.26-7.15 (m, 8H), 6.93 (t, 1H, J = 7.5 Hz), 6.46 (d, 1H, J = 7.8 Hz), 5.15, 4.36 (ABq, 2H, J_{AB} = 13.7 Hz), 2.71-2.58 (m, 5H), 2.45 (s, 3H), 1.26-1.08 (m, 7H), 0.87 (t, 3H, J = 7.3 Hz), 0.34-0.26 (m, 1H); ^{13}C NMR (101 MHz, $CDCl_3$) δ 170.4, 143.9, 143.7, 137.5, 136.3, 134.9, 129.9, 129.5, 128.5, 128.2, 127.9, 125.6, 124.1, 122.8, 118.4, 118.2, 115.1, 54.0, 31.5, 30.0, 29.5, 27.4, 26.9, 22.8, 21.8, 14.3; HRMS (ESI): m/z calcd for $C_{30}H_{35}N_2O_3S$ $[M + H]^+$ 503.2363, found 503.2370.

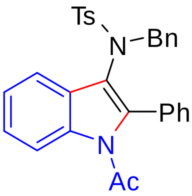

3ag

To an oven-dried tube was added *N*-acetyl-2-iodoaniline **1a** (78.3 mg, 0.30 mmol), ynamide **2g**[2] (72.3 mg, 0.20 mmol), $PdCl_2(PPh_3)_2$ (28.1 mg, 0.04 mmol), PPh_3 (5.3 mg, 0.02 mmol), K_3PO_4 (84.9 mg, 0.40 mmol), and DCE (2.0 mL, ynamide *concn* = 0.10 *M*). The tube was capped and stirred in an oil bath at 100 °C for 5.0 h. After the reaction was judged to be complete by TLC analysis, the

reaction mixture was cooled to room temperature and filtered through a short pad of silica gel. The filtrate was concentrated under reduced pressure, and the residue was purified by flash column chromatography on silica gel (gradient eluent: 15:1~5:1 petroleum ether/EtOAc) to afford **3ag** (90.2 mg, 0.18 mmol) in 91% yield.

3ag: R_f = 0.4 [5:1 petroleum ether/EtOAc]; white solid; mp = 124-125 °C; ^{1}H NMR (400 MHz, $CDCl_3$) δ 8.36 (d, 1H, J = 8.6 Hz), 7.67 (d, 2H, J = 8.3 Hz), 7.37-7.24 (m, 7H), 7.11-6.92 (m, 5H), 6.65 (t, 3H, J = 8.0 Hz), 4.81, 4.31 (ABq, 2H, J_{AB} = 13.4 Hz), 2.48 (s, 3H), 1.82 (s, 3H); ^{13}C NMR (101 MHz, $CDCl_3$) δ 171.7, 144.1, 139.7, 137.1, 135.8, 134.9, 131.0, 130.5, 129.9, 129.3, 128.9, 128.4, 128.3, 128.2, 127.8, 125.6, 125.3, 123.5, 119.6, 119.1, 116.5, 53.9, 27.9, 21.8; HRMS (ESI): m/z calcd for $C_{30}H_{27}N_2O_3S$ $[M + H]^+$ 495.1737, found 495.1745.

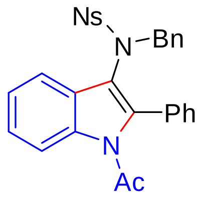

3ah

To an oven-dried tube was added *N*-acetyl-2-iodoaniline **1a** (78.3 mg, 0.30 mmol), ynamide **2h**[2] (78.5 mg, 0.20 mmol), $PdCl_2(PPh_3)_2$ (28.1 mg, 0.04 mmol), PPh_3 (5.3 mg, 0.02 mmol), K_3PO_4 (84.9 mg, 0.40 mmol), and DCE (2.0 mL, ynamide *concn* = 0.10 *M*). The tube was capped and stirred in an oil bath at 100 °C for 6.0 h. After the reaction was judged to be complete by TLC analysis, the reaction mixture was cooled to room temperature and filtered through a short pad of silica gel. The filtrate was concentrated under reduced pressure, and the residue was purified by flash column chromatography on silica gel (gradient eluent: 15:1~5:1 petroleum ether/EtOAc) to afford **3ah** (90.3 mg, 0.17 mmol) in 86% yield.

3ah: R_f = 0.41 [5:1 petroleum ether/EtOAc]; white solid; mp = 180-181 °C; ^{1}H NMR (400 MHz, $CDCl_3$) δ 8.39 (d, 1H, J = 8.4 Hz), 8.27 (d, 2H, J = 8.7 Hz), 7.89 (d, 2H, J = 8.7 Hz), 7.40-7.32 (m, 3H), 7.26-7.15 (m, 3H), 7.05 (dt, 4H, J = 15.4, 7.6 Hz), 6.76 (d, 2H, J = 7.5 Hz), 6.59 (d, 1H, J = 7.9 Hz), 4.87, 4.42 (ABq, 2H, J_{AB} = 14.9 Hz), 1.83 (s, 3H); ^{13}C NMR (101 MHz, $CDCl_3$) δ 171.5, 150.4, 146.0, 139.8, 135.7, 134.3, 130.7, 130.4, 129.6, 129.5, 129.3, 128.51, 128.49, 128.3, 126.0, 125.0, 124.4, 123.8, 118.8, 118.2, 116.9, 54.6, 27.9; HRMS (ESI): m/z calcd for $C_{29}H_{24}N_3O_5S$ $[M + H]^+$ 526.1431, found 526.1436.

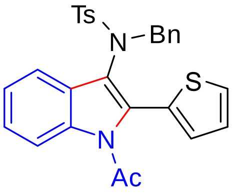

3ai

To an oven-dried tube was added *N*-acetyl-2-iodoaniline **1a** (78.3 mg, 0.30 mmol), ynamide **2i**[7] (73.4 mg, 0.20 mmol), $PdCl_2(PPh_3)_2$ (28.1 mg, 0.04 mmol), PPh_3 (5.3 mg, 0.02 mmol), K_3PO_4 (84.9 mg, 0.40 mmol), and DCE (2.0 mL, ynamide *concn* = 0.10 *M*). The tube was capped and stirred in an oil bath at 100 °C for 4.0 h. After the reaction was judged to be complete by TLC analysis, the reaction mixture was cooled to room temperature and filtered through a short pad of silica gel. The filtrate was concentrated under reduced pressure, and the residue was purified by flash column chromatography on silica gel (gradient eluent: 15:1~5:1 petroleum ether/EtOAc) to afford **3ai** (94.1 mg, 0.19 mmol) in 94% yield.

3ai: R_f = 0.33 [5:1 petroleum ether/EtOAc]; white solid; mp = 159-160 °C; ^{1}H NMR (400 MHz, $CDCl_3$) δ 8.31 (d, 1H, *J* = 8.5 Hz), 7.70 (d, 2H, *J* = 8.3 Hz), 7.42 (dd, 1H, *J* = 5.1, 1.2 Hz), 7.31-7.27 (m, 3H), 7.11-7.07 (m, 1H), 7.03-6.96 (m, 4H), 6.91 (dd, 1H, *J* = 3.6, 1.2 Hz), 6.78 (d, 2H, *J* = 6.7 Hz), 6.51 (d, 1H, *J* = 8.1 Hz), 4.90, 4.34 (ABq, 2H, J_{AB} = 13.6 Hz), 2.49 (s, 3H), 2.03 (s, 3H); ^{13}C NMR (101 MHz, $CDCl_3$) δ 171.7, 144.1, 137.2, 135.9, 135.2, 132.9, 131.1, 130.8, 129.9, 129.11, 129.07, 128.23, 128.21, 127.8, 127.2, 125.9, 125.0, 123.5, 121.0, 119.0, 116.3, 54.0, 26.9, 21.8; HRMS (ESI): m/z calcd for $C_{28}H_{25}N_2O_3S_2$ $[M + H]^+$ 501.1301, found 501,1309.

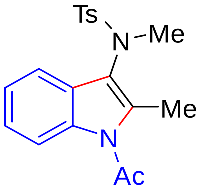

3aj

To an oven-dried tube was added *N*-acetyl-2-iodoaniline **1a** (78.3 mg, 0.30 mmol), ynamide **2j**[8] (44.7 mg, 0.20 mmol), $PdCl_2(PPh_3)_2$ (28.1 mg, 0.04 mmol), PPh_3 (5.3 mg, 0.02 mmol), K_3PO_4 (84.9 mg, 0.40 mmol), and DCE (2.0 mL, ynamide *concn* = 0.10 *M*). The tube was capped and stirred in an oil bath at 100 °C for 3.0 h. After the reaction was judged to be complete by TLC analysis, the reaction mixture was cooled to room temperature and filtered through a short pad of silica gel. The filtrate was concentrated under reduced pressure, and the residue was purified by flash column chromatography on silica gel (gradient eluent: 15:1~5:1 petroleum ether/EtOAc) to afford **3aj** (57.9 mg, 0.16 mmol) in 81% yield.

3aj: R_f = 0.20 [5:1 petroleum ether/EtOAc]; white solid; mp = 110-111 °C; ^{1}H NMR (400 MHz,

$CDCl_3$) δ 8.07 (d, 1H, J = 8.5 Hz), 7.63 (d, 2H, J = 8.3 Hz), 7.26-7.18 (m, 3H), 6.98 (t, 1H, J = 7.3 Hz), 6.56 (d, 1H, J = 7.8 Hz), 3.28 (s, 3H), 2.74 (s, 3H), 2.54 (s, 3H), 2.44 (s, 3H); ^{13}C NMR (101 MHz, $CDCl_3$) δ 170.6, 143.9, 136.7, 136.3, 134.7, 129.8, 127.9, 126.0, 124.4, 123.1, 121.9, 118.2, 115.8, 38.0, 27.7, 21.8, 14.5; HRMS (ESI): m/z calcd for $C_{19}H_{20}N_2NaO_3S$ [M + Na]$^+$ 379.1087, found 379.1095.

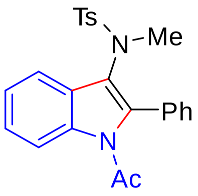

3ak

To an oven-dried tube was added *N*-acetyl-2-iodoaniline **1a** (78.3 mg, 0.30 mmol), ynamide **2k**[2] (57.1 mg, 0.20 mmol), $PdCl_2(PPh_3)_2$ (28.1 mg, 0.04 mmol), PPh_3 (5.3 mg, 0.02 mmol), K_3PO_4 (84.9 mg, 0.40 mmol), and DCE (2.0 mL, ynamide *concn* = 0.10 *M*). The tube was capped and stirred in an oil bath at 100 °C for 4.0 h. After the reaction was judged to be complete by TLC analysis, the reaction mixture was cooled to room temperature and filtered through a short pad of silica gel. The filtrate was concentrated under reduced pressure, and the residue was purified by flash column chromatography on silica gel (gradient eluent: 15:1~5:1 petroleum ether/EtOAc) to afford **3ak** (83.2 mg, 0.19 mmol) in 99% yield.

3ak: R_f = 0.35 [5:1 petroleum ether/EtOAc]; white solid; mp = 193-194 °C; ^{1}H NMR (400 MHz, $CDCl_3$) δ 8.40 (d, 1H, J = 8.4 Hz), 7.53 (d, 2H, J = 8.3 Hz), 7.45 (s, 5H), 7.33 (t, 1H, J = 7.3 Hz), 7.21 (d, 2H, J = 8.0 Hz), 7.12 (t, 1H, J = 7.2 Hz), 6.88 (d, 1H, J = 7.8 Hz), 3.07 (s, 3H), 2.44 (s, 3H), 1.96 (s, 3H); ^{13}C NMR (101 MHz, $CDCl_3$) δ 171.4, 143.8, 137.8, 136.3, 135.8, 131.5, 130.3, 129.7, 129.4, 128.8, 128.1, 125.82, 125.79, 123.7, 122.9, 119.1, 116.6, 38.3, 27.9, 21.7; HRMS (ESI): m/z calcd for $C_{24}H_{23}N_2O_3S$ [M + H]$^+$ 419.1424, found 419.1430.

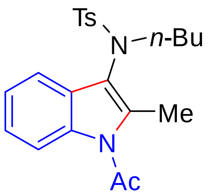

3al

To an oven-dried tube was added *N*-acetyl-2-iodoaniline **1a** (78.3 mg, 0.30 mmol), ynamide **2l**[6] (53.1 mg, 0.20 mmol), $PdCl_2(PPh_3)_2$ (28.1 mg, 0.04 mmol), PPh_3 (5.3 mg, 0.02 mmol), K_3PO_4 (84.9 mg, 0.40 mmol), and DCE (2.0 mL, ynamide *concn* = 0.10 *M*). The tube was capped and stirred in an oil bath at 100 °C for 3.0 h. After the reaction was judged to be complete by TLC analysis, the reaction mixture was cooled to room temperature and filtered through a short pad of silica gel. The

filtrate was concentrated under reduced pressure, and the residue was purified by flash column chromatography on silica gel (gradient eluent: 15:1~5:1 petroleum ether/EtOAc) to afford **3al** (50.5 mg, 0.13 mmol) in 63% yield.

3al: R_f = 0.37 [5:1 petroleum ether/EtOAc]; white solid; mp = 165-166 °C; ^{1}H NMR (400 MHz, $CDCl_3$) δ 8.07 (d, 1H, J = 8.4 Hz), 7.60 (d, 2H, J = 8.3 Hz), 7.21 (d, 3H, J = 8.0 Hz), 6.96 (t, 1H, J = 7.5 Hz), 6.53 (d, 1H, J = 7.8 Hz), 3.82-3.75 (m, 1H), 3.44-3.37 (m, 1H), 2.75 (s, 3H), 2.54 (s, 3H), 2.42 (s, 3H), 1.45-1.24 (m, 4H), 0.86 (t, 3H, J = 7.1 Hz); ^{13}C NMR (101 MHz, $CDCl_3$) δ 170.6, 143.7, 137.9, 137.2, 134.8, 129.8, 127.7, 126.3, 124.4, 123.1, 119.8, 118.2, 115.8, 50.3, 31.5, 27.8, 21.7, 20.0, 14.7, 13.9; HRMS (ESI): m/z calcd for $C_{22}H_{27}N_2O_3S$ $[M + H]^+$ 399.1737, found 399.1741.

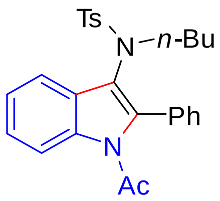

3am

To an oven-dried tube was added *N*-acetyl-2-iodoaniline **1a** (78.3 mg, 0.30 mmol), ynamide **2m**[9] (65.5 mg, 0.20 mmol), $PdCl_2(PPh_3)_2$ (28.1 mg, 0.04 mmol), PPh_3 (5.3 mg, 0.02 mmol), K_3PO_4 (84.9 mg, 0.40 mmol), and DCE (2.0 mL, ynamide *concn* = 0.10 *M*). The tube was capped and stirred in an oil bath at 100 °C for 4.0 h. After the reaction was judged to be complete by TLC analysis, the reaction mixture was cooled to room temperature and filtered through a short pad of silica gel. The filtrate was concentrated under reduced pressure, and the residue was purified by flash column chromatography on silica gel (gradient eluent: 15:1~5:1 petroleum ether/EtOAc) to afford **3am** (85.4 mg, 0.19 mmol) in 93% yield.

3am: R_f = 0.40 [5:1 petroleum ether/EtOAc]; white solid; mp = 204-205 °C; ^{1}H NMR (400 MHz, $CDCl_3$) δ 8.39 (d, 1H, J = 8.4 Hz), 7.70-7.58 (m, 4H), 7.47-7.46 (m, 3H), 7.31 (t, 1H, J = 7.8 Hz), 7.26-7.24 (m, 2H), 7.03 (t, 1H, J = 7.6 Hz), 6.61 (d, 1H, J = 7.9 Hz), 3.48-3.30 (m, 2H), 2.47 (s, 3H), 1.98 (s, 3H), 1.10-0.87 (m, 4H), 0.60 (t, 3H, J = 7.3 Hz); ^{13}C NMR (101 MHz, $CDCl_3$) δ 171.7, 143.8, 139.1, 137.2, 135.9, 131.6, 130.6, 129.8, 129.4, 128.8, 128.2, 125.8, 125.7, 123.5, 120.7, 119.3, 116.4, 50.5, 30.7, 28.0, 21.8, 19.7, 13.7; HRMS (ESI): m/z calcd for $C_{27}H_{29}N_2O_3S$ $[M + H]^+$ 461.1893, found 461.1900.

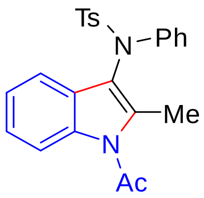

3an

To an oven-dried tube was added *N*-acetyl-2-iodoaniline **1a** (78.3 mg, 0.30 mmol), ynamide **2n**[6] (57.1 mg, 0.20 mmol), $PdCl_2(PPh_3)_2$ (28.1 mg, 0.04 mmol), PPh_3 (5.3 mg, 0.02 mmol), K_3PO_4 (84.9 mg, 0.40 mmol), and DCE (2.0 mL, ynamide *concn* = 0.10 *M*). The tube was capped and stirred in an oil bath at 100 °C for 6.0 h. After the reaction was judged to be complete by TLC analysis, the reaction mixture was cooled to room temperature and filtered through a short pad of silica gel. The filtrate was concentrated under reduced pressure, and the residue was purified by flash column chromatography on silica gel (gradient eluent: 15:1~5:1 petroleum ether/EtOAc) to afford **3an** (67.7 mg, 0.16 mmol) in 81% yield.

3an: R_f = 0.40 [5:1 petroleum ether/EtOAc]; white solid; mp = 152-153 °C; ^{1}H NMR (400 MHz, $CDCl_3$) δ 8.02 (d, 1H, *J* = 8.4 Hz), 7.67 (d, 2H, *J* = 8.3 Hz), 7.38 (d, 2H, *J* = 7.3 Hz), 7.29-7.23 (m, 5H), 7.18 (t, 1H, *J* = 7.3 Hz), 7.12 (t, 1H, *J* = 7.3 Hz), 7.09-7.07 (m, 1H), 2.75 (s, 3H), 2.58 (s, 3H), 2.45 (s, 3H); ^{13}C NMR (101 MHz, $CDCl_3$) δ 170.5, 144.1, 141.6, 138.1, 137.8, 134.7, 129.8, 129.4, 128.1, 127.5, 126.7, 125.6, 124.6, 123.6, 122.1, 118.8, 115.6, 27.8, 21.8, 14.7; HRMS (ESI): m/z calcd for $C_{24}H_{23}N_2O_3S$ $[M + H]^+$ 419.1424, found 419.1431.

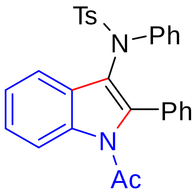

3ao

To an oven-dried tube was added *N*-acetyl-2-iodoaniline **1a** (78.3 mg, 0.30 mmol), ynamide **2o**[2] (69.5 mg, 0.20 mmol), $PdCl_2(PPh_3)_2$ (28.1 mg, 0.04 mmol), PPh_3 (5.3 mg, 0.02 mmol), K_3PO_4 (84.9 mg, 0.40 mmol) and DCE (2.0 mL, ynamide *concn* = 0.10 *M*). The tube was capped and stirred in an oil bath at 100 °C for 6.0 h. After the reaction was judged to be complete by TLC analysis, the reaction mixture was cooled to room temperature and filtered through a short pad of silica gel. The filtrate was concentrated under reduced pressure, and the residue was purified by flash column chromatography on silica gel (gradient eluent: 15:1~5:1 petroleum ether/EtOAc) to afford **3ao** (86.2 mg, 0.18 mmol) in 90% yield.

3ao: R_f = 0.34 [5:1 petroleum ether/EtOAc]; white solid; mp = 158-159 °C; ^{1}H NMR (400 MHz, $CDCl_3$) δ 8.42 (d, 1H, *J* = 8.5 Hz), 7.54-7.47 (m, 3H), 7.43 (t, 3H, *J* = 6.8 Hz), 7.39-7.33 (m, 2H),

7.24-7.16 (m, 4H), 7.12-7.07 (m, 3H), 6.91 (dd, 2H, *J* = 8.0, 1.9 Hz), 2.44 (s, 3H), 1.96 (s, 3H); ^{13}C NMR (101 MHz, $CDCl_3$) δ 171.4, 144.0, 141.3, 138.7, 137.7, 135.7, 131.4, 130.6, 129.6, 129.0, 128.8, 128.2, 127.2, 127.14, 127.08, 125.9, 124.1, 123.0, 119.5, 116.7, 28.0, 21.8, one carbon missing due to overlap, overlapped signal at 129.6 ppm; HRMS (ESI): m/z calcd for $C_{29}H_{25}N_2O_3S$ [M + H]$^+$ 481.1580, found 481.1587.

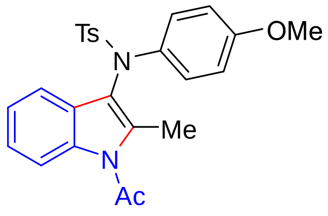

3ap

To an oven-dried tube was added *N*-acetyl-2-iodoaniline **1a** (78.3 mg, 0.30 mmol), ynamide **2p**[10] (63.1 mg, 0.20 mmol), $PdCl_2(PPh_3)_2$ (28.1 mg, 0.04 mmol), PPh_3 (5.3 mg, 0.02 mmol), K_3PO_4 (84.9 mg, 0.40 mmol), and DCE (2.0 mL, ynamide *concn* = 0.10 *M*). The tube was capped and stirred in an oil bath at 100 °C for 5.0 h. After the reaction was judged to be complete by TLC analysis, the reaction mixture was cooled to room temperature and filtered through a short pad of silica gel. The filtrate was concentrated under reduced pressure, and the residue was purified by flash column chromatography on silica gel (gradient eluent: 15:1~5:1 petroleum ether/EtOAc) to afford **3ap** (76.0 mg, 0.17 mmol) in 85% yield.

3ap: R_f = 0.5 [5:1 petroleum ether/EtOAc]; white solid; ^{1}H NMR (400 MHz, $CDCl_3$) δ 7.99 (d, 1H, *J* = 8.4 Hz), 7.63 (d, 2H, *J* = 8.3 Hz), 7.32 (d, 2H, *J* = 9.0 Hz), 7.27 (s, 1H), 7.26-7.22 (m, 2H), 7.16-7.09 (m, 2H), 6.79 (d, 2H, *J* = 9.0 Hz), 3.76 (s, 3H), 2.73 (s, 3H), 2.61 (s, 3H), 2.45 (s, 3H); ^{13}C NMR (101 MHz, $CDCl_3$) δ 170.5, 158.6, 143.9, 138.1, 137.3, 134.6, 134.2, 129.7, 128.5, 128.0, 127.5, 124.5, 123.5, 122.6, 118.7, 115.5, 114.5, 55.5, 27.7, 21.7, 14.6; Spectral data are in agreement with literature values[11].

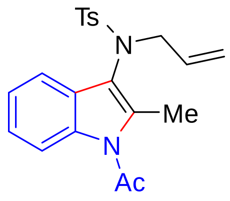

3aq

To an oven-dried tube was added *N*-acetyl-2-iodoaniline **1a** (78.3 mg, 0.30 mmol), ynamide **2q**[5] (49.9 mg, 0.20 mmol), $PdCl_2(PPh_3)_2$ (28.1 mg, 0.04 mmol), PPh_3 (5.3 mg, 0.02 mmol), K_3PO_4 (84.9 mg, 0.40 mmol), and DCE (2.0 mL, ynamide *concn* = 0.10 *M*). The tube was capped and stirred in an oil bath at 100 °C for 4.0 h. After the reaction was judged to be complete by TLC analysis, the

reaction mixture was cooled to room temperature and filtered through a short pad of silica gel. The filtrate was concentrated under reduced pressure, and the residue was purified by flash column chromatography on silica gel (gradient eluent: 15:1~5:1 petroleum ether/EtOAc) to afford **3aq** (43.7 mg, 0.11 mmol) in 57% yield, and starting material **1a** was recovered in 41% yield.

3aq: R_f = 0.25 [5:1 petroleum ether/EtOAc]; white solid; mp = 104-105 °C; ^{1}H NMR (400 MHz, $CDCl_3$) δ 8.05 (d, 1H, J = 8.4 Hz), 7.62 (d, 2H, J = 8.3 Hz), 7.21 (m, 3H), 6.98 (t, 1H, J = 7.8 Hz), 6.56 (d, 1H, J = 7.8 Hz), 5.88-5.78 (m, 1H), 5.01-4.96 (m, 2H), 4.46 (dd, 1H, J = 14.2, 5.7 Hz), 3.98 (dd, 1H, J = 14.2, 7.8 Hz), 2.74 (s, 3H), 2.51 (s, 3H), 2.43 (s, 3H); ^{13}C NMR (101 MHz, $CDCl_3$) δ 170.6, 143.9, 138.1, 137.3, 134.7, 133.0, 129.8, 127.7, 126.4, 124.4, 123.1, 119.6, 119.4, 118.1, 115.8, 53.4, 27.8, 21.8, 14.8; HRMS (ESI): m/z calcd for $C_{21}H_{23}N_2O_3S$ $[M + H]^+$ 383.1424, found 383.1433.

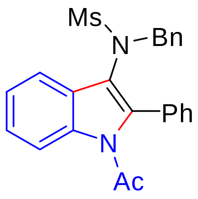

3ar

To an oven-dried tube was added *N*-acetyl-2-iodoaniline **1a** (78.3 mg, 0.30 mmol), ynamide **2r**[12] (57.1 mg, 0.20 mmol), $PdCl_2(PPh_3)_2$ (28.1 mg, 0.04 mmol), PPh_3 (5.3 mg, 0.02 mmol), K_3PO_4 (84.9 mg, 0.40 mmol), and DCE (2.0 mL, ynamide *concn* = 0.10 *M*). The tube was capped and stirred in an oil bath at 100 °C for 5.0 h. After the reaction was judged to be complete by TLC analysis, the reaction mixture was cooled to room temperature and filtered through a short pad of silica gel. The filtrate was concentrated under reduced pressure, and the residue was purified by flash column chromatography on silica gel (gradient eluent: 15:1~5:1 petroleum ether/EtOAc) to afford **3ar** (82.0 mg, 0.19 mmol) in 98% yield.

3ar: R_f = 0.24 [5:1 petroleum ether/EtOAc]; white solid; mp = 130-131 °C; ^{1}H NMR (400 MHz, $CDCl_3$) δ 8.43 (d, 1H, J = 8.2 Hz), 7.51 (d, 1H, J = 7.7 Hz), 7.43-7.33 (m, 4H), 7.30-7.26 (m, 3H), 7.19 (t, 1H, J = 7.4 Hz), 7.07 (t, 2H, J = 7.6 Hz), 6.87 (d, 2H, J = 7.2 Hz), 4.82, 4.54 (ABq, 2H, J = 13.9 Hz), 2.94 (s, 3H), 1.86 (s, 3H); ^{13}C NMR (101 MHz, $CDCl_3$) δ 171.5, 139.0, 135.8, 135.4, 131.2, 130.4, 129.5, 129.3, 128.6, 128.5, 128.1, 126.0, 125.9, 124.2, 120.3, 118.3, 117.0, 54.6, 41.0, 27.9; HRMS (ESI): m/z calcd for $C_{24}H_{23}N_2O_3S$ $[M + H]^+$ 419.1424, found 419.1432.

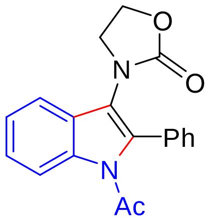

3as

To an oven-dried tube was added *N*-acetyl-2-iodoaniline **1a** (78.3 mg, 0.30 mmol), ynamide **2s**[2] (37.4 mg, 0.20 mmol), $PdCl_2(PPh_3)_2$ (28.1 mg, 0.04 mmol), PPh_3 (5.3 mg, 0.02 mmol), K_3PO_4 (84.9 mg, 0.40 mmol), and DCE (2.0 mL, ynamide *concn* = 0.10 *M*). The tube was capped and stirred in an oil bath at 100 °C for 5.0 h. After the reaction was judged to be complete by TLC analysis, the reaction mixture was cooled to room temperature and filtered through a short pad of silica gel. The filtrate was concentrated under reduced pressure, and the residue was purified by flash column chromatography on silica gel (gradient eluent: 15:1~5:1 petroleum ether/EtOAc) to afford **3as** (50.0 mg, 0.16 mmol) in 78% yield.

3as: R_f = 0.27 [3:1 petroleum ether/EtOAc]; white solid; mp = 189-190 °C; ^{1}H NMR (400 MHz, $CDCl_3$) δ 8.42 (d, 1H, J = 8.4 Hz), 7.52-7.50 (m, 6H), 7.42 (t, 1H, J = 7.8 Hz), 7.35 (t, 1H, J = 7.9 Hz), 4.36 (t, 2H, J = 8.0 Hz), 3.54 (t, 2H, J = 8.0 Hz), 2.02 (s, 3H); ^{13}C NMR (101 MHz, $CDCl_3$) δ 171.2, 158.0, 136.1, 135.9, 131.2, 129.80, 129.77, 129.3, 126.4, 125.3, 124.3, 119.2, 118.0, 116.7, 62.9, 46.7, 27.8; HRMS (ESI): m/z calcd for $C_{19}H_{17}N_2O_3$ $[M + H]^+$ 321.1234, found 321.1237.

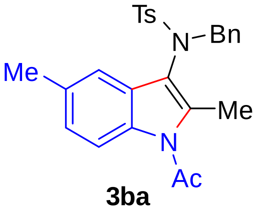

3ba

To an oven-dried tube was added *N*-protected *o*-iodoaniline **1b**[13] (82.5 mg, 0.30 mmol), ynamide **2a** (59.9 mg, 0.20 mmol), $PdCl_2(PPh_3)_2$ (28.1 mg, 0.04 mmol), PPh_3 (5.3 mg, 0.02 mmol), K_3PO_4 (84.9 mg, 0.40 mmol), and DCE (2.0 mL, ynamide *concn* = 0.10 *M*). The tube was capped and stirred in an oil bath at 100 °C for 6.0 h. After the reaction was judged to be complete by TLC analysis, the reaction mixture was cooled to room temperature and filtered through a short pad of silica gel. The filtrate was concentrated under reduced pressure, and the residue was purified by flash column chromatography on silica gel (gradient eluent: 15:1~5:1 petroleum ether/EtOAc) to afford **3ba** (77.6 mg, 0.17 mmol) in 87% yield.

3ba: R_f = 0.25 [5:1 petroleum ether/EtOAc]; white solid; mp = 158-159 °C; ^{1}H NMR (400 MHz, $CDCl_3$) δ 7.93 (d, 1H, J = 8.5 Hz), 7.66 (d, 2H, J = 8.3 Hz), 7.26-7.14 (m, 7H), 6.98 (m, 1H), 6.03 (s,

1H), 5.19, 4.29 (ABq, 2H, J_{AB} = 13.6 Hz), 2.59 (s, 3H), 2.46 (s, 3H), 2.15 (s, 3H), 2.07 (s, 3H); ^{13}C NMR (101 MHz, $CDCl_3$) δ 170.4, 143.8, 138.7, 137.6, 136.1, 133.2, 132.5, 129.8, 129.5, 128.5, 128.1, 127.9, 125.8, 125.5, 118.6, 117.9, 115.7, 54.1, 27.6, 21.7, 21.1, 14.1; HRMS (ESI): m/z calcd for $C_{26}H_{27}N_2O_3S$ [M + H]$^+$ 447.1737, found 447.1743.

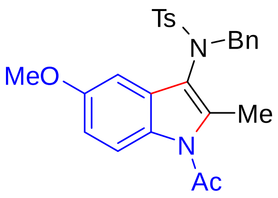

3ca

To an oven-dried tube was added *N*-protected *o*-iodoaniline **1c**[14] (87.3 mg, 0.30 mmol), ynamide **2a** (59.9 mg, 0.20 mmol), $PdCl_2(PPh_3)_2$ (28.1 mg, 0.04 mmol), PPh_3 (5.3 mg, 0.02 mmol), K_3PO_4 (84.9 mg, 0.40 mmol), and DCE (2.0 mL, ynamide *concn* = 0.10 *M*). The tube was capped and stirred in an oil bath at 100 °C for 6.0 h. After the reaction was judged to be complete by TLC analysis, the reaction mixture was cooled to room temperature and filtered through a short pad of silica gel. The filtrate was concentrated under reduced pressure, and the residue was purified by flash column chromatography on silica gel (gradient eluent: 15:1~5:1 petroleum ether/EtOAc) to afford **3ca** (87.1 mg, 0.19 mmol) in 94% yield.

3ca: R_f = 0.33 [5:1 petroleum ether/EtOAc]; white solid; mp = 172-173 °C; ^{1}H NMR (400 MHz, $CDCl_3$) δ 8.00 (d, 1H, J = 9.1 Hz), 7.69 (d, 2H, J = 8.3 Hz), 7.27-7.15 (m, 7H), 6.77 (dd, 1H, J = 9.1, 2.6 Hz), 5.83 (d, 1H, J = 2.6 Hz), 5.13, 4.37 (ABq, 2H, J_{AB} = 13.6 Hz), 3.52 (s, 3H), 2.58 (s, 3H), 2.43 (s, 3H), 2.08 (s, 3H); ^{13}C NMR (101 MHz, $CDCl_3$) δ 170.2, 155.9, 143.8, 139.1, 137.8, 136.1, 129.9, 129.6, 129.5, 128.5, 128.2, 127.8, 126.7, 118.8, 117.1, 113.1, 100.1, 55.2, 54.0, 27.5, 21.7, 14.2; HRMS (ESI): m/z calcd for $C_{26}H_{27}N_2O_4S$ [M + H]$^+$ 463.1686, found 463.1693.

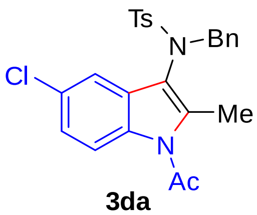

3da

To an oven-dried tube was added *N*-protected *o*-iodoaniline **1d**[14] (88.7 mg, 0.30 mmol), ynamide **2a** (59.9 mg, 0.20 mmol), $PdCl_2(PPh_3)_2$ (28.1 mg, 0.04 mmol), PPh_3 (5.3 mg, 0.02 mmol), K_3PO_4 (84.9 mg, 0.40 mmol), and DCE (2.0 mL, ynamide *concn* = 0.10 *M*). The tube was capped and stirred in an oil bath at 100 °C for 6.0 h. After the reaction was judged to be complete by TLC analysis, the reaction mixture was cooled to room temperature and filtered through a short pad of silica gel. The filtrate was concentrated under reduced pressure, and the residue was purified by flash

column chromatography on silica gel (gradient eluent: 15:1~5:1 petroleum ether/EtOAc) to afford **3da** (77.4 mg, 0.17 mmol) in 83% yield.

3da: R_f = 0.31 [5:1 petroleum ether/EtOAc]; white solid; mp = 173-174 °C; ^{1}H NMR (400 MHz, $CDCl_3$) δ 8.07 (d, 1H, J = 8.9 Hz), 7.63 (d, 2H, J = 8.3 Hz), 7.28 (d, 2H, J = 8.0 Hz), 7.25-7.10 (m, 6H), 6.04 (d, 1H, J = 2.1 Hz), 5.22, 4.22 (ABq, 2H, J_{AB} = 13.4 Hz), 2.58 (s, 3H), 2.48 (s, 3H), 2.12 (s, 3H); ^{13}C NMR (101 MHz, $CDCl_3$) δ 170.3, 144.6, 140.0, 137.1, 135.8, 133.3, 130.1, 129.5, 128.9, 128.6, 128.3, 127.7, 126.7, 124.4, 118.1, 117.4, 117.3, 54.1, 27.5, 21.8, 14.1; HRMS (ESI): m/z calcd for $C_{25}H_{24}ClN_2O_3S$ [M + H]$^+$ 467.1191, found 467.1195.

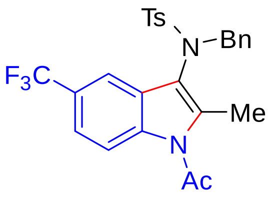

3ea

To an oven-dried tube was added *N*-protected *o*-iodoaniline **1e**[15] (98.7 mg, 0.30 mmol), ynamide **2a** (59.9 mg, 0.20 mmol), $PdCl_2(PPh_3)_2$ (28.1 mg, 0.04 mmol), PPh_3 (5.3 mg, 0.02 mmol), K_3PO_4 (84.9 mg, 0.40 mmol), and DCE (2.0 mL, ynamide *concn* = 0.10 *M*). The tube was capped and stirred in an oil bath at 100 °C for 6.0 h. After the reaction was judged to be complete by TLC analysis, the reaction mixture was cooled to room temperature and filtered through a short pad of silica gel. The filtrate was concentrated under reduced pressure, and the residue was purified by flash column chromatography on silica gel (gradient eluent: 15:1~5:1 petroleum ether/EtOAc) to afford **3ea** (82.0 mg, 0.16 mmol) in 82% yield.

3ea: R_f = 0.40 [5:1 petroleum ether/EtOAc]; white solid; mp = 166-167 °C; ^{1}H NMR (400 MHz, $CDCl_3$) δ 8.24 (d, 1H, J = 8.8 Hz), 7.62 (d, 2H, J = 8.3 Hz), 7.40 (dd, 1H, J = 8.8, 1.4 Hz), 7.25-7.19 (m, 5H), 7.16-7.13 (m, 2H), 6.34 (s, 1H), 5.27, 4.27 (ABq, 2H, J_{AB} = 13.5 Hz), 2.62 (s, 3H), 2.43 (s, 3H), 2.18 (s, 3H); ^{13}C NMR (101 MHz, $CDCl_3$) δ 170.4, 144.8, 140.6, 137.1, 136.4, 135.8, 130.1, 129.5, 128.6, 128.3, 127.5, 125.3 (q, $^2J_{C\text{-}F}$ = 32.1 Hz), 125.2, 124.4 (q, $^1J_{C\text{-}F}$ = 270.3 Hz), 121.1 (q, $^3J_{C\text{-}F}$ = 3.5 Hz), 118.7, 116.6,114.9 (q, $^3J_{C\text{-}F}$ = 4.2 Hz), 54.4, 27.6, 21.6, 14.1; ^{19}F NMR (376 MHz, $CDCl_3$) δ -61.6; HRMS (ESI): m/z calcd for $C_{26}H_{24}F_3N_2O_3S$ [M + H]$^+$ 501.1454, found 501.1460.

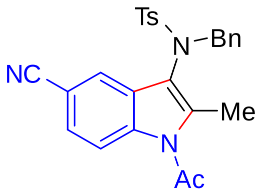

3fa

To an oven-dried tube was added *N*-protected *o*-iodoaniline **1f**[13] (85.8 mg, 0.30 mmol), ynamide **2a** (59.9 mg, 0.20 mmol), $PdCl_2(PPh_3)_2$ (28.1 mg, 0.04 mmol), PPh_3 (5.3 mg, 0.02 mmol), K_3PO_4 (84.9 mg, 0.40 mmol), and DCE (2.0 mL, ynamide *concn* = 0.10 *M*). The tube was capped and stirred in an oil bath at 100 °C for 6.0 h. After the reaction was judged to be complete by TLC analysis, the reaction mixture was cooled to room temperature and filtered through a short pad of silica gel. The filtrate was concentrated under reduced pressure, and the residue was purified by flash column chromatography on silica gel (gradient eluent: 15:1~5:1 petroleum ether/EtOAc) to afford **3fa** (69.0 mg, 0.15 mmol) in 75% yield.

3fa: R_f = 0.37 [5:1 petroleum ether/EtOAc]; white solid; mp = 156-157 °C; ^{1}H NMR (400 MHz, $CDCl_3$) δ 8.25 (d, 1H, *J* = 8.7 Hz), 7.63 (d, 2H, *J* = 8.3 Hz), 7.42 (dd, 1H, *J* = 8.7, 1.7 Hz), 7.31 (d, 2H, *J* = 8.0 Hz), 7.26-7.20 (m, 3H), 7.14-7.12 (m, 2H), 6.37 (s, 1H), 5.23, 4.24 (ABq, 2H, J_{AB} = 13.4 Hz), 2.62 (s, 3H), 2.53 (s, 3H), 2.17 (s, 3H); ^{13}C NMR (101 MHz, $CDCl_3$) δ 170.3, 145.1, 141.0, 136.9, 136.6, 135.5, 130.3, 129.5, 128.7, 128.4, 127.6, 127.5, 125.6, 122.3, 119.2, 118.3, 117.1, 106.6, 54.4, 27.6, 21.8, 14.1; HRMS (ESI): m/z calcd for $C_{26}H_{24}N_3O_3S$ [M + H]$^+$ 458.1533, found 458.1536.

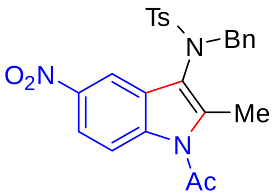

3ga

To an oven-dried tube was added *N*-protected *o*-iodoaniline **1g**[13] (91.8 mg, 0.30 mmol), ynamide **2a** (59.9 mg, 0.20 mmol), $PdCl_2(PPh_3)_2$ (28.1 mg, 0.04 mmol), PPh_3 (5.3 mg, 0.02 mmol), K_3PO_4 (84.9 mg, 0.40 mmol), and DCE (2.0 mL, ynamide *concn* = 0.10 *M*). The tube was capped and stirred in an oil bath at 100 °C for 7.0 h. After the reaction was judged to be complete by TLC analysis, the reaction mixture was cooled to room temperature and filtered through a short pad of silica gel. The filtrate was concentrated under reduced pressure, and the residue was purified by flash column chromatography on silica gel (gradient eluent: 15:1~5:1 petroleum ether/EtOAc) to afford **3ga** (71.2 mg, 0.15 mmol) in 75% yield.

3ga: R_f = 0.42 [5:1 petroleum ether/EtOAc]; white solid; mp = 160–161 °C; ^{1}H NMR (400 MHz, $CDCl_3$) δ 8.28 (d, 1H, *J* = 9.2 Hz), 8.06 (dd, 1H, *J* = 9.2, 2.3 Hz), 7.65 (d, 2H, *J* = 8.3 Hz), 7.29-7.27 (m, 2H), 7.26-7.21 (m, 3H), 7.16-7.13 (m, 2H), 7.00 (d, 1H, *J* = 2.3 Hz), 5.29, 4.29 (ABq, 2H, J_{AB} = 13.4 Hz), 2.63 (s, 3H), 2.45 (s, 3H), 2.19 (s, 3H); ^{13}C NMR (101 MHz, $CDCl_3$) δ 170.3, 145.2, 143.6, 141.9, 137.8, 136.8, 135.5, 130.2, 129.5, 128.7, 128.4, 127.5, 125.5, 119.7, 119.1, 116.6, 113.7, 54.5,

27.6, 21.8, 14.2; HRMS (ESI): m/z calcd for $C_{25}H_{24}N_3O_5S$ $[M + H]^+$ 478.1431, found 478.1434.

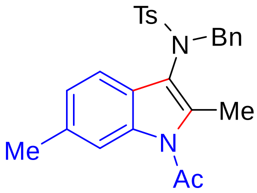

3ha

To an oven-dried tube was added *N*-protected *o*-iodoaniline **1h**[14] (82.5 mg, 0.30 mmol), ynamide **2a** (59.9 mg, 0.20 mmol), $PdCl_2(PPh_3)_2$ (28.1 mg, 0.04 mmol), PPh_3 (5.3 mg, 0.02 mmol), K_3PO_4 (84.9 mg, 0.40 mmol), and DCE (2.0 mL, ynamide *concn* = 0.10 *M*). The tube was capped and stirred in an oil bath at 100 °C for 6.0 h. After the reaction was judged to be complete by TLC analysis, the reaction mixture was cooled to room temperature and filtered through a short pad of silica gel. The filtrate was concentrated under reduced pressure, and the residue was purified by flash column chromatography on silica gel (gradient eluent: 15:1~5:1 petroleum ether/EtOAc) to afford **3ha** (80.4 mg, 0.18 mmol) in 90% yield.

3ha: R_f = 0.31 [5:1 petroleum ether/EtOAc]; white solid; mp = 135-136 °C; ^{1}H NMR (400 MHz, $CDCl_3$) δ 7.93 (s, 1H), 7.66 (d, 2H, J = 8.3 Hz), 7.26-7.12 (m, 7H), 6.79 (d, 1H, J = 7.9 Hz), 6.36 (d, 1H, J = 7.9 Hz), 5.13, 4.32 (ABq, 2H, J_{AB} = 13.6 Hz), 2.59 (s, 3H), 2.45 (s, 3H), 2.42 (s, 3H), 2.06 (s, 3H); ^{13}C NMR (101 MHz, $CDCl_3$) δ 170.6, 143.8, 137.7, 137.5, 136.1, 135.4, 134.3, 129.9, 129.5, 128.5, 128.1, 127.8, 124.5, 123.4, 118.9, 117.5, 116.4, 53.9, 27.6, 22.1, 21.8, 14.1; HRMS (ESI): m/z calcd for $C_{26}H_{27}N_2O_3S$ $[M + H]^+$ 447.1737, found 447.1743.

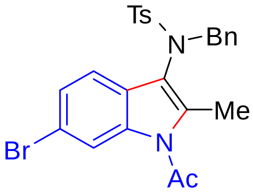

3ia

To an oven-dried tube was added *N*-protected *o*-iodoaniline **1i**[16] (102.0 mg, 0.30 mmol), ynamide **2a** (59.9 mg, 0.20 mmol), $PdCl_2(PPh_3)_2$ (28.1 mg, 0.04 mmol), PPh_3 (5.3 mg, 0.02 mmol), K_3PO_4 (84.9 mg, 0.40 mmol), and DCE (2.0 mL, ynamide *concn* = 0.10 *M*). The tube was capped and stirred in an oil bath at 100 °C for 6.0 h. After the reaction was judged to be complete by TLC analysis, the reaction mixture was cooled to room temperature and filtered through a short pad of silica gel. The filtrate was concentrated under reduced pressure, and the residue was purified by flash column chromatography on silica gel (gradient eluent: 15:1~5:1 petroleum ether/EtOAc) to afford **3ia** (81.8 mg, 0.16 mmol) in 80% yield.

3ia: R_f = 0.27 [5:1 petroleum ether/EtOAc]; white solid; mp = 186-187 °C; ^{1}H NMR (400 MHz, $CDCl_3$) δ 8.36 (s, 1H), 7.65 (d, 2H, J = 8.1 Hz), 7.27 (d, 2H, J = 8.0 Hz), 7.23-7.17 (m, 3H), 7.12-7.06 (m, 3H), 6.30 (d, 1H, J = 8.3 Hz), 5.12, 4.30 (ABq, 2H, J_{AB} = 13.6 Hz), 2.58 (s, 3H), 2.46 (s, 3H), 2.07 (s, 3H); ^{13}C NMR (101 MHz, $CDCl_3$) δ 170.3, 144.1, 138.9, 137.3, 135.8, 135.6, 130.0, 129.4, 128.5, 128.3, 127.7, 126.4, 124.6, 119.4, 118.8, 118.7, 118.2, 54.0, 27.5, 21.8, 14.1; HRMS (ESI): m/z calcd for $C_{25}H_{24}BrN_2O_3S$ [M + H]$^+$ 511.0686, found 511.0688.

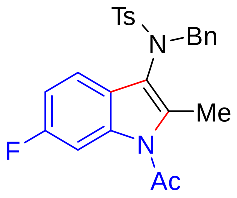

3ja

To an oven-dried tube was added *N*-protected *o*-iodoaniline **1j**[14] (83.7 mg, 0.30 mmol), ynamide **2a** (59.9 mg, 0.20 mmol), $PdCl_2(PPh_3)_2$ (28.1 mg, 0.04 mmol), PPh_3 (5.3 mg, 0.02 mmol), K_3PO_4 (84.9 mg, 0.40 mmol), and DCE (2.0 mL, ynamide *concn* = 0.10 *M*). The tube was capped and stirred in an oil bath at 100 °C for 7.0 h. After the reaction was judged to be complete by TLC analysis, the reaction mixture was cooled to room temperature and filtered through a short pad of silica gel. The filtrate was concentrated under reduced pressure, and the residue was purified by flash column chromatography on silica gel (gradient eluent: 15:1~5:1 petroleum ether/EtOAc) to afford **3ja** (67.4 mg, 0.15 mmol) in 75% yield.

3ja: R_f = 0.37 [5:1 petroleum ether/EtOAc]; white solid; mp = 108–109 °C; ^{1}H NMR (400 MHz, $CDCl_3$) δ 7.93 (dd, 1H, J = 10.9, 2.3 Hz), 7.65 (d, 2H, J = 8.2 Hz), 7.26 (t, 3H, J = 4.0 Hz), 7.21 (d, 2H, J = 7.3 Hz), 7.14-7.12 (m, 2H), 6.72 (td, 1H, J = 8.8, 2.3 Hz), 6.36 (dd, 1H, J = 8.6, 5.4 Hz), 5.15, 4.30 (ABq, 2H, J_{AB} = 13.5 Hz), 2.58 (s, 3H), 2.46 (s, 3H), 2.08 (s, 3H); ^{13}C NMR (101 MHz, $CDCl_3$) δ 170.4, 160.8 (d, $^1J_{C\text{-}F}$ = 238.8 Hz), 144.1, 138.4 (d, $^4J_{C\text{-}F}$ = 3.6 Hz), 137.4, 135.9, 135.1 (d, $^3J_{C\text{-}F}$ = 12.1 Hz), 130.0, 129.5, 128.5, 128.3, 127.7, 122.0, 118.7, 118.3 (d, $^3J_{C\text{-}F}$ = 10.5 Hz), 111.4 (d, $^2J_{C\text{-}F}$ = 24.0 Hz), 104.1 (d, $^2J_{C\text{-}F}$ = 28.9 Hz), 54.0, 27.4, 21.8, 14.2; ^{19}F NMR (376 MHz, $CDCl_3$) δ -117.2; HRMS (ESI): m/z calcd for $C_{25}H_{24}FN_2O_3S$ [M + H]$^+$ 451.1486, found 451.1491.

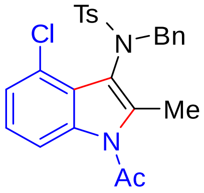

3ka

To an oven-dried tube was added *N*-protected *o*-iodoaniline **1k**[13] (88.7 mg, 0.30 mmol), ynamide **2a** (59.9 mg, 0.20 mmol), $PdCl_2(PPh_3)_2$ (28.1 mg, 0.04 mmol), PPh_3 (5.3 mg, 0.02 mmol), K_3PO_4

(84.9 mg, 0.40 mmol), and DCE (2.0 mL, ynamide *concn* = 0.10 *M*). The tube was capped and stirred in an oil bath at 100 °C for 6.5 h. After the reaction was judged to be complete by TLC analysis, the reaction mixture was cooled to room temperature and filtered through a short pad of silica gel. The filtrate was concentrated under reduced pressure, and the residue was purified by flash column chromatography on silica gel (gradient eluent: 15:1~5:1 petroleum ether/EtOAc) to afford **3ka** (65.7 mg, 0.14 mmol) in 70% yield.

3ka: R_f = 0.32 [5:1 petroleum ether/EtOAc]; white solid; mp = 167-168 °C; ^{1}H NMR (400 MHz, $CDCl_3$) δ 8.12 (d, 1H, J = 8.3 Hz), 7.63 (d, 2H, J = 8.3 Hz), 7.26-7.11 (m, 7H), 7.17 (d, 1H, J = 8.2 Hz), 7.12-7.10 (m, 1H), 5.26, 4.35 (ABq, 2H, J_{AB} = 14.2 Hz), 2.59 (s, 3H), 2.43 (s, 3H), 1.85 (s, 3H); ^{13}C NMR (101 MHz, $CDCl_3$) δ 170.5, 143.4, 140.2, 138.1, 136.5, 136.0, 130.2, 129.7, 128.5, 128.2, 127.7, 125.1, 123.7, 123.6, 117.0, 114.4, 56.3, 27.8, 21.8, 14.4, one carbon missing due to overlap, overlapped signal at 125.1 ppm; HRMS (ESI): m/z calcd for $C_{25}H_{24}ClN_2O_3S$ $[M + H]^+$ 467.1191, found 467.1196.

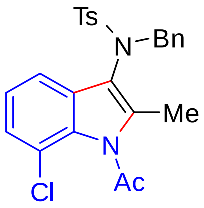

3la

To an oven-dried tube was added *N*-protected *o*-iodoaniline **1l**[13] (88.7 mg, 0.30 mmol), ynamide **2a** (59.9 mg, 0.20 mmol), $PdCl_2(PPh_3)_2$ (28.1 mg, 0.04 mmol), PPh_3 (5.3 mg, 0.02 mmol), K_3PO_4 (84.9 mg, 0.40 mmol), and DCE (2.0 mL, ynamide *concn* = 0.10 *M*). The tube was capped and stirred in an oil bath at 100 °C for 7.0 h. After the reaction was judged to be complete by TLC analysis, the reaction mixture was cooled to room temperature and filtered through a short pad of silica gel. The filtrate was concentrated under reduced pressure, and the residue was purified by flash column chromatography on silica gel (gradient eluent: 15:1~5:1 petroleum ether/EtOAc) to afford **3la** (86.7 mg, 0.19 mmol) in 93% yield.

3la: R_f = 0.39 [5:1 petroleum ether/EtOAc]; white solid; mp = 119–120 °C; ^{1}H NMR (400 MHz, $CDCl_3$) δ 7.66 (d, 2H, J = 8.2 Hz), 7.26-7.10 (m, 8H), 6.89 (t, 1H, J = 7.8 Hz), 6.51 (d, 1H, J = 7.8 Hz), 5.09, 4.36 (ABq, 2H, J_{AB} = 13.6 Hz), 2.50 (s, 3H), 2.44 (s, 3H), 1.84 (s, 3H); ^{13}C NMR (101 MHz, $CDCl_3$) δ 173.1, 143.9, 139.5, 137.4, 136.1, 130.9, 129.9, 129.4, 128.8, 128.5, 128.2, 127.7, 124.5, 123.3, 118.8, 117.0, 115.5, 54.3, 29.3, 21.8, 11.3; HRMS (ESI): m/z calcd for $C_{25}H_{24}ClN_2O_3S$ $[M + H]^+$ 467.1191, found 467.1194.

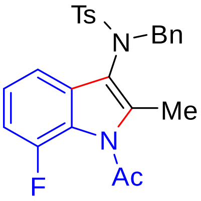

3ma

To an oven-dried tube was added *N*-protected *o*-iodoaniline **1m**[1] (83.7 mg, 0.30 mmol), ynamide **2a** (59.9 mg, 0.20 mmol), $PdCl_2(PPh_3)_2$ (28.1 mg, 0.04 mmol), PPh_3 (5.3 mg, 0.02 mmol), K_3PO_4 (84.9 mg, 0.40 mmol), and DCE (2.0 mL, ynamide *concn* = 0.10 *M*). The tube was capped and stirred in an oil bath at 100 °C for 7.0 h. After the reaction was judged to be complete by TLC analysis, the reaction mixture was cooled to room temperature and filtered through a short pad of silica gel. The filtrate was concentrated under reduced pressure, and the residue was purified by flash column chromatography on silica gel (gradient eluent: 15:1~5:1 petroleum ether/EtOAc) to afford **3ma** (68.6 mg, 0.15 mmol) in 76% yield.

3ma: R_f = 0.37 [5:1 petroleum ether/EtOAc]; white solid; mp = 128–129 °C; ^{1}H NMR (400 MHz, $CDCl_3$) δ 7.67 (d, 2H, J = 8.3 Hz), 7.27 (s, 1H), 7.25 (s, 1H), 7.22-7.18 (m, 3H), 7.14-7.11 (m, 2H), 6.92-6.88 (m, 2H), 6.42-6.30 (m, 1H), 5.05, 4.38 (ABq, 2H, J_{AB} = 13.6 Hz), 2.57 (d, 3H, J = 5.9 Hz), 2.45 (s, 3H), 1.96 (s, 3H); ^{13}C NMR (101 MHz, $CDCl_3$) δ 171.4, 149.6 (d, $^1J_{C\text{-}F}$ = 246.3 Hz), 143.9, 140.7, 137.4, 136.0, 130.1 (d, $^4J_{C\text{-}F}$ = 3.3 Hz), 129.9, 129.4, 128.5, 128.2, 127.7, 123.6 (d, $^3J_{C\text{-}F}$ = 7.3 Hz), 121.3 (d, $^3J_{C\text{-}F}$ = 10.1 Hz), 117.1 (d, $^4J_{C\text{-}F}$ = 3.2 Hz), 114.2 (d, $^4J_{C\text{-}F}$ = 3.3 Hz), 110.4 (d, $^2J_{C\text{-}F}$ = 20.9 Hz), 54.2, 26.9 (d, $^2J_{C\text{-}F}$ = 15.7 Hz), 21.8, 12.5; ^{19}F NMR (376 MHz, $CDCl_3$) δ -120.1; HRMS (ESI): m/z calcd for $C_{25}H_{24}FN_2O_3S$ $[M + H]^+$ 451.1486, found 451.1490.

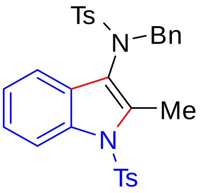

3na

To an oven-dried tube was added *N*-protected *o*-iodoaniline **1n**[17] (112.0 mg, 0.30 mmol), ynamide **2a** (59.9 mg, 0.20 mmol), $PdCl_2(PPh_3)_2$ (28.1 mg, 0.04 mmol), PPh_3 (5.3 mg, 0.02 mmol), K_3PO_4 (84.9 mg, 0.40 mmol), and DCE (2.0 mL, ynamide *concn* = 0.10 *M*). The tube was capped and stirred in an oil bath at 100 °C for 9.0 h. After the reaction was judged to be complete by TLC analysis, the reaction mixture was cooled to room temperature and filtered through a short pad of silica gel. The filtrate was concentrated under reduced pressure, and the residue was purified by flash column chromatography on silica gel (gradient eluent: 15:1~5:1 petroleum ether/EtOAc) to afford

3na (54.5 mg, 0.10 mmol) in 50% yield.

3na: R_f = 0.33 [5:1 petroleum ether/EtOAc]; white solid; mp = 143–144 °C; ^{1}H NMR (400 MHz, $CDCl_3$) δ 8.10 (d, 1H, J = 8.4 Hz), 7.50-7.46 (m, 4H), 7.20-7.14 (m, 6H), 7.08 (t, 2H, J = 7.5 Hz), 7.01-6.99 (m, 2H), 6.95 (t, 1H, J = 7.5 Hz), 6.52 (d, 1H, J = 7.8 Hz), 5.07, 4.30 (ABq, 2H, J_{AB} = 13.4 Hz), 2.41 (s, 3H), 2.39 (s, 3H), 2.01 (s, 3H); ^{13}C NMR (101 MHz, $CDCl_3$) δ 145.0, 143.8, 139.4, 137.1, 135.90, 135.85, 135.2, 130.0, 129.8, 129.3, 128.4, 128.0, 127.7, 126.5, 126.4, 124.3, 123.4, 119.0, 118.2, 114.9, 53.8, 21.8, 21.7, 12.5; HRMS (ESI): m/z calcd for $C_{30}H_{29}N_2O_4S_2$ $[M + H]^+$ 545.1563, found 545.1569.

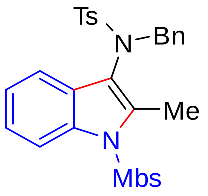

3oa

To an oven-dried tube was added *N*-protected *o*-iodoaniline **1g**[18] (116.8 mg, 0.30 mmol), ynamide **2a** (59.9 mg, 0.20 mmol), $PdCl_2(PPh_3)_2$ (28.1 mg, 0.04 mmol), PPh_3 (5.3 mg, 0.02 mmol), K_3PO_4 (84.9 mg, 0.40 mmol), and DCE (2.0 mL, ynamide *concn* = 0.10 *M*). The tube was capped and stirred in an oil bath at 100 °C for 11.0 h. After the reaction was judged to be complete by TLC analysis, the reaction mixture was cooled to room temperature and filtered through a short pad of silica gel. The filtrate was concentrated under reduced pressure, and the residue was purified by flash column chromatography on silica gel (gradient eluent: 15:1~5:1 petroleum ether/EtOAc) to afford **3oa** (51.7 mg, 0.09 mmol) in 46% yield.

3oa: R_f = 0.43 [5:1 petroleum ether/EtOAc]; white solid; mp = 121–122 °C; ^{1}H NMR (400 MHz, $CDCl_3$) δ 8.10 (d, 1H, J = 8.5 Hz), 7.51 (dd, 4H, J = 14.2, 8.5 Hz), 7.19-7.15 (m, 4H), 7.09 (t, 2H, J = 7.4 Hz), 7.01 (d, 2H, J = 7.2 Hz), 6.95 (t, 1H, J = 7.5 Hz), 6.85 (d, 2H, J = 9.0 Hz), 6.50 (d, 1H, J = 7.8 Hz), 5.08, 4.29 (ABq, 2H, J_{AB} = 13.4 Hz), 3.83 (s, 3H), 2.41 (s, 3H), 2.01 (s, 3H); ^{13}C NMR (101 MHz, $CDCl_3$) δ 163.8, 143.8, 139.4, 137.1, 135.8, 135.1, 130.2, 129.8, 129.3, 128.7, 128.4, 128.0, 127.6, 126.4, 124.3, 123.4, 118.8, 118.1, 114.9, 114.5, 55.9, 53.8, 21.8, 12.5; HRMS (ESI): m/z calcd for $C_{30}H_{29}N_2O_5S_2$ $[M + H]^+$ 561.1512, found 561.1506.

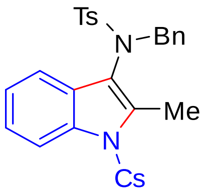

3pa

To an oven-dried tube was added *N*-protected *o*-iodoaniline **1p**[19] (118.1 mg, 0.30 mmol), ynamide **2a** (59.9 mg, 0.20 mmol), $PdCl_2(PPh_3)_2$ (28.1 mg, 0.04 mmol), PPh_3 (5.3 mg, 0.02 mmol), K_3PO_4 (84.9 mg, 0.40 mmol), and DCE (2.0 mL, ynamide *concn* = 0.10 *M*). The tube was capped and stirred in an oil bath at 100 °C for 10.0 h. After the reaction was judged to be complete by TLC analysis, the reaction mixture was cooled to room temperature and filtered through a short pad of silica gel. The filtrate was concentrated under reduced pressure, and the residue was purified by flash column chromatography on silica gel (gradient eluent: 15:1~5:1 petroleum ether/EtOAc) to afford **3pa** (41.8 mg, 0.07 mmol) in 37% yield.

3pa: R_f = 0.37 [5:1 petroleum ether/EtOAc]; white solid; mp = 118–119 °C; ^{1}H NMR (400 MHz, $CDCl_3$) δ 8.07 (d, 1H, *J* = 8.5 Hz), 7.51-7.47 (m, 4H), 7.36 (d, 2H, *J* = 8.7 Hz), 7.18-7.16 (m, 4H), 7.09 (t, 2H, *J* = 7.6 Hz), 7.01-6.95 (m, 3H), 6.50 (d, *J* = 7.8 Hz, 1H), 5.11, 4.26 (ABq, 2H, J_{AB} = 13.4 Hz), 2.42 (s, 3H), 2.04 (s, 3H); ^{13}C NMR (101 MHz, $CDCl_3$) δ 144.0, 140.6, 139.4, 137.1, 137.0, 135.7, 135.2, 129.9, 129.7, 129.3, 128.4, 128.1, 127.8, 127.7, 126.5, 124.6, 123.8, 119.6, 118.4, 114.9, 53.7, 21.8, 12.6; HRMS (ESI): m/z calcd for $C_{29}H_{26}ClN_2O_4S_2$ $[M + H]^+$ 565.1017, found 565.1014.

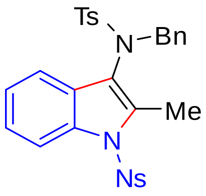

3qa

To an oven-dried tube was added *N*-protected *o*-iodoaniline **1q**[20] (121.3 mg, 0.30 mmol), ynamide **2a** (59.9 mg, 0.20 mmol), $PdCl_2(PPh_3)_2$ (28.1 mg, 0.04 mmol), PPh_3 (5.3 mg, 0.02 mmol), K_3PO_4 (84.9 mg, 0.40 mmol), and DCE (2.0 mL, ynamide *concn* = 0.10 *M*). The tube was capped and stirred in an oil bath at 100 °C for 9.0 h. After the reaction was judged to be complete by TLC analysis, the reaction mixture was cooled to room temperature and filtered through a short pad of silica gel. The filtrate was concentrated under reduced pressure, and the residue was purified by flash column chromatography on silica gel (gradient eluent: 15:1~5:1 petroleum ether/EtOAc) to afford **3qa** (35.36 mg, 0.06 mmol) in 31% yield.

3qa: R_f = 0.37 [5:1 petroleum ether/EtOAc]; white solid; mp = 109–110 °C; ^{1}H NMR (400 MHz, $CDCl_3$) δ 8.19 (d, 2H, *J* = 8.9 Hz), 8.04 (d, 1H, *J* = 8.4 Hz), 7.67 (d, 2H, *J* = 8.8 Hz), 7.52 (d, 2H, *J* = 8.2 Hz), 7.22-7.15 (m, 4H), 7.09 (t, 2H, *J* = 7.5 Hz), 7.02-6.97 (m, 3H), 6.50 (d, 1H, *J* = 7.9 Hz), 5.11, 4.27 (ABq, 2H, J_{AB} = 13.4 Hz), 2.43 (s, 3H), 2.13 (s, 3H); ^{13}C NMR (101 MHz, $CDCl_3$) δ 150.6, 144.2, 143.8, 139.4, 137.0, 135.5, 135.1, 129.9, 129.3, 128.4, 128.2, 127.7, 127.6, 126.6, 125.0,

124.6, 124.2, 120.4, 118.6, 114.9, 53.6, 21.8, 12.9; HRMS (ESI): m/z calcd for $C_{29}H_{26}N_3O_6S_2$ [M + H]$^+$ 576.1258, found 576.1259.

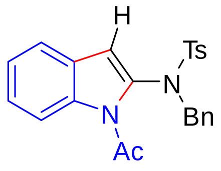

4au

To an oven-dried tube was added *N*-acetyl-2-iodoaniline **1a** (78.3 mg, 0.30 mmol), ynamide **2u**[6] (57.1 mg, 0.20 mmol), $Pd(PPh_3)_4$ (46.2 mg, 0.04 mmol), PPh_3 (5.3 mg, 0.02 mmol), Cs_2CO_3 (130.3, 0.40 mmol), and DCE (2.0 mL, ynamide *concn* = 0.10 *M*). The tube was capped and stirred in an oil bath at 100 °C for 4.0 h. After the reaction was judged to be complete by TLC analysis, the reaction mixture was cooled to room temperature and filtered through a short pad of silica gel. The filtrate was concentrated under reduced pressure, and the residue was purified by flash column chromatography on silica gel (gradient eluent: 15:1~5:1 petroleum ether/EtOAc) to afford **4au** (38.0 mg, 0.09 mmol) in 45% yield.

4au: R_f = 0.37 [5:1 petroleum ether/EtOAc]; white solid; ^{1}H NMR (400 MHz, $CDCl_3$) δ 8.31 (d, 1H, J = 8.4 Hz), 7.63 (d, 2H, J = 8.3 Hz), 7.44 (d, 1H, J = 7.6 Hz), 7.39-7.32 (m, 4H), 7.25-7.20 (m, 3H), 7.20-7.17 (m, 2H), 6.01 (s, 1H), 5.11, 4.21 (ABq, 2H, J_{AB} = 12.9 Hz), 2.51 (s, 3H), 2.50 (s, 3H); ^{13}C NMR (101 MHz, $CDCl_3$) δ 170.7, 144.9, 135.4, 134.4, 133.9, 133.2, 130.1, 129.8, 129.0, 128.9, 126.6, 125.9, 123.5, 120.6, 117.1, 107.0, 57.9, 26.8, 21.9; Spectral data are in agreement with literature values[21].

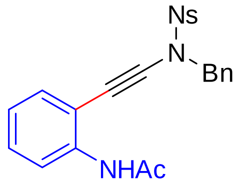

5av

To an oven-dried tube was added *N*-acetyl-2-iodoaniline **1a** (78.3 mg, 0.30 mmol), ynamide **2v**[22] (63.3 mg, 0.20 mmol), $Pd(PPh_3)_4$ (46.2 mg, 0.04 mmol), PPh_3 (5.3 mg, 0.02 mmol), Cs_2CO_3 (130.3, 0.40 mmol), and DCE (2.0 mL, ynamide *concn* = 0.10 *M*). The tube was capped and stirred in an oil bath at 100 °C for 4.5 h. After the reaction was judged to be complete by TLC analysis, the reaction mixture was cooled to room temperature and filtered through a short pad of silica gel. The filtrate was concentrated under reduced pressure, and the residue was purified by flash column chromatography on silica gel (gradient eluent: 15:1~5:1 petroleum ether/EtOAc) to afford **5av** (37.0 mg, 0.08 mmol) in 41% yield.

5av: R_f = 0.32 [5:1 petroleum ether/EtOAc]; white solid; mp = 124-125 °C; ^{1}H NMR (400 MHz, $CDCl_3$) δ 8.39 (d, 1H, J = 8.4 Hz), 8.33 (d, 2H, J = 8.6 Hz), 8.01 (d, 2H, J = 8.6 Hz), 7.92 (s, 1H), 7.32 (s, 6H), 7.19 (d, 1H, J = 7.5 Hz), 6.99 (t, 1H, J = 7.5 Hz), 4.68 (s, 2H), 2.15 (s, 3H); ^{13}C NMR (101 MHz, $CDCl_3$) δ 169.1, 150.8, 143.1, 139.5, 133.4, 130.7, 129.8, 129.2, 129.0, 128.9, 128.8, 124.7, 123.3, 119.6, 110.6, 88.2, 68.0, 55.8, 24.9; HRMS (ESI): m/z calcd for $C_{23}H_{19}N_3NaO_5S$ [M + Na]$^+$ 472.0938, found 472.0946.

1.3 Chemical Transformations of the Annulation Products

To an oven-dried tube was added 3-aminoindole **3aa** (108.1 mg, 0.25 mmol), $NH_3 \cdot H_2O$ (0.5 ml), and MeOH (1.0 mL).[23] The tube was capped and stirred in an oil bath at 40 °C for 6.0 h. After the reaction was judged to be complete by TLC analysis, the reaction mixture was extracted with ethyl acetate, washed with brine solution, dried over anhydrous Na_2SO_4, filtered, concentrated in vacuo, and purified by flash silica gel column chromatography (gradient eluent: 10:1~2:1 petroleum ether/EtOAc) to afford **6** (97.0 mg, 0.25 mmol) in 99% yield.

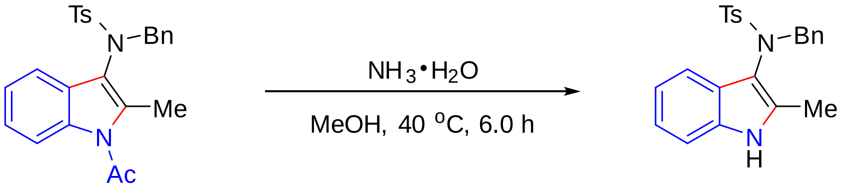

3aa **6:** 99%

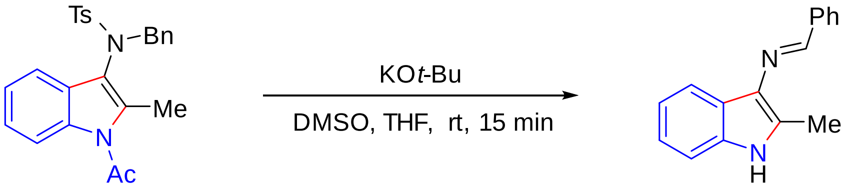

3aa **7:** 90%

6: R_f = 0.36 [5:1 petroleum ether/EtOAc]; white solid; mp = 116-117 °C; ^{1}H NMR (400 MHz, $CDCl_3$) δ 7.79 (s, 1H), 7.64 (d, 2H, J = 8.3 Hz), 7.26-7.12 (m, 8H), 7.02 (t, 1H, J = 7.2 Hz), 6.79 (t, 1H, J = 7.8 Hz), 6.43 (d, 1H, J = 7.9 Hz), 5.22, 4.20 (ABq, 2H, J_{AB} = 13.5 Hz), 2.44 (s, 3H), 1.90 (s, 3H); ^{13}C NMR (101 MHz, $CDCl_3$) δ 143.4, 137.7, 137.0, 136.8, 133.9, 129.7, 129.4, 128.3, 127.82, 127.76, 124.3, 121.3, 119.7, 117.6, 111.3, 111.0, 54.2, 21.8, 11.2; HRMS (ESI): m/z calcd for $C_{23}H_{23}N_2O_2S$ [M + H]$^+$ 391.1475, found 391.1475.

To an oven-dried tube was added 3-aminoindole **3aa** (86.5 mg, 0.20 mmol), DMSO (140.0 μL, 3-Aminoindoles concn = 1.4 *M*), and THF (1.0 mL, 3-Aminoindoles *concn* = 0.2 *M*). The reaction mixture was stirred, and 1.0 *M* KOt-Bu in THF solution (0.4 mL, 0.40 mmol) was added dropwise at

room temperature.[24] The reaction vessel was capped and stirred at room temperature for 15.0 minutes. After the reaction was judged to be complete by TLC analysis, the reaction mixture was diluted with EtOAc (3.0 mL), quenched with H_2O (4.0 mL), and extracted with EtOAc (2 × 3.0 mL). The combined extract was dried over anhydrous Na_2SO_4 and evaporated in vacuo. The residue was purified by flash silica gel column chromatography (gradient eluent: 10:1~2:1 petroleum ether/EtOAc) to afford **7** (42.1 mg, 0.18 mmol) in 90% yield.

7: R_f = 0.47 [5:1 petroleum ether/EtOAc]; white solid; mp = 106-107 °C; ^{1}H NMR (400 MHz, CDCl3), δ 9.08 (s, 1H), 8.01-7.90 (m, 4H), 7.49-7.39 (m, 3H), 7.31 (dd, 1H, J = 6.0, 3.2 Hz), 7.18 (dd, 2H, J = 6.0, 3.1 Hz), 2.63 (s, 3H); ^{13}C NMR (101 MHz, CDCl3) δ 153.0, 138.3, 135.0, 133.7, 129.9, 128.8, 127.6, 124.9, 122.0, 121.7, 120.8, 119.4, 111.2, 11.5.; HRMS (ESI): m/z calcd for $C_{16}H_{15}N_2$ $[M + H]^+$ 235.1230, found 235.1228.

1.4 X-Ray Crystal Structures of 3ca

The relative configuration of the **3ca** was determined by X-ray. The crystal was obtained by slow evaporation of the solution of **3ca** in petroleum ether/ethyl acetate (4:1) at room temperature. A colorless crystal of approximate dimensions 0.3 x 0.2 x 0.2 mm was selected and collected by an Agilent Xcalibur Eos Gemini diffractometer. The crystal was kept at 293 K during data collection. The structure was solved by direct methods using Olex2 software with the SHELXS structure solution program. The found structural model was further refined with the SHELXL refinement package using Least Squares minimisation.

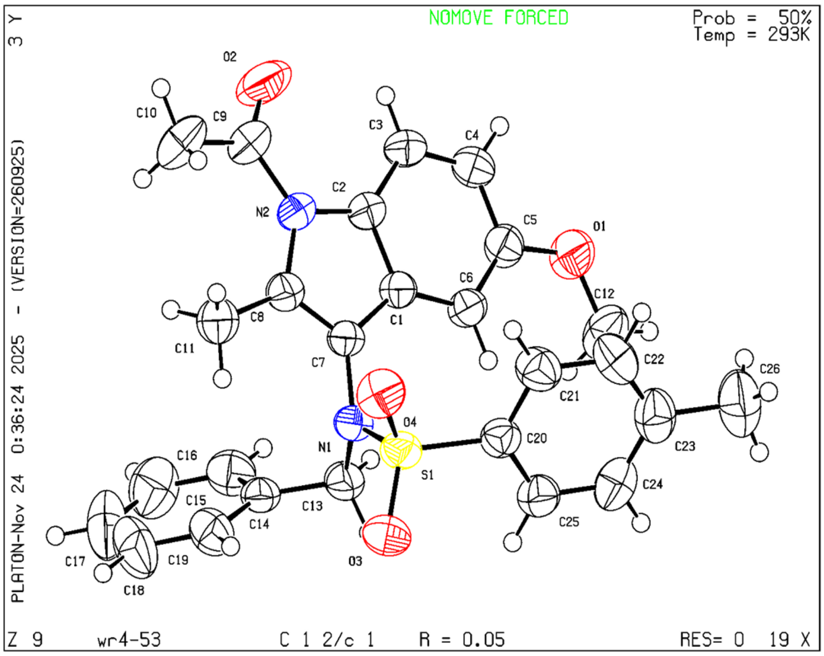

Figure S1. The thermal ellipsoid plot of **3ca**

Table S2. Crystal Data and Structure Refinement for 3ca

Empirical formula	$C_{26}H_{26}N_2O_4S$
Formula weight	462.55
Temperature/K	293(2)
Crystal system	monoclinic
Space group	C2/c
a/Å	43.8290(8)
b/Å	10.08920(10)
c/Å	11.4190(2)
α/°	90
β/°	110.632(2)
γ/°	90
Volume/Å^3	4725.62(14)
Z	8
ρ_{calc}g/cm^3	1.300
μ/mm^{-1}	1.504
F(000)	1952.0
Crystal size/mm^3	0.3 × 0.2 × 0.2
Radiation	CuKα (λ = 1.54184)
2Θ range for data collection/°	8.622 to 141.814
Index ranges	$-53 \leq h \leq 47$, $-10 \leq k \leq 12$, $-10 \leq l \leq 13$
Reflections collected	9345
Independent reflections	4459 [R_{int} = 0.0248, R_{sigma} = 0.0342]
Data/restraints/parameters	4459/0/302
Goodness-of-fit on F^2	1.020
Final R indexes [I>=2σ (I)]	R_1 = 0.0497, wR_2 = 0.1376
Final R indexes [all data]	R_1 = 0.0629, wR_2 = 0.1504
Largest diff. peak/hole / e Å^{-3}	0.26/-0.24

1.5 Control Experiments

Control experiment with unprotected 2-iodoaniline

The reaction mixture became messy, and the desired product was isolated in only 36% yield, indicating that the free NH_2 group is detrimental to the annulation.

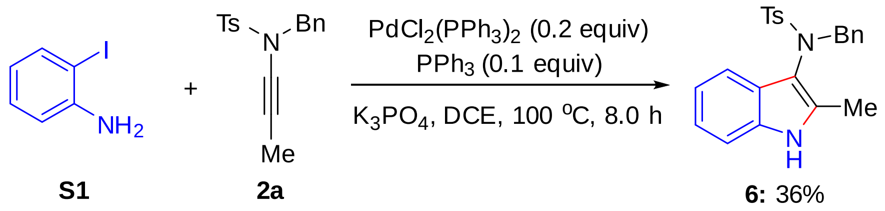

Attempt with *N*-acetyl-2-bromoaniline

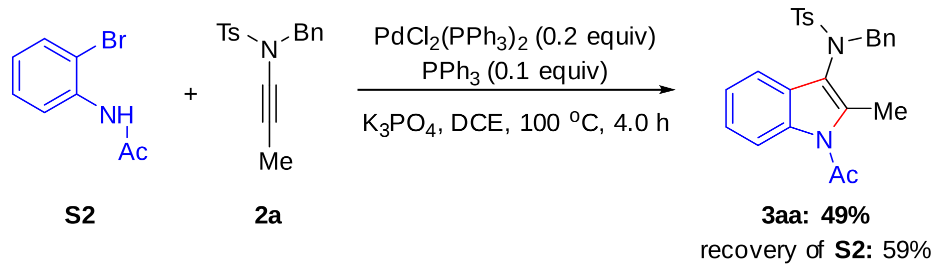

Attempt with *N*-TFA-2-iodoaniline

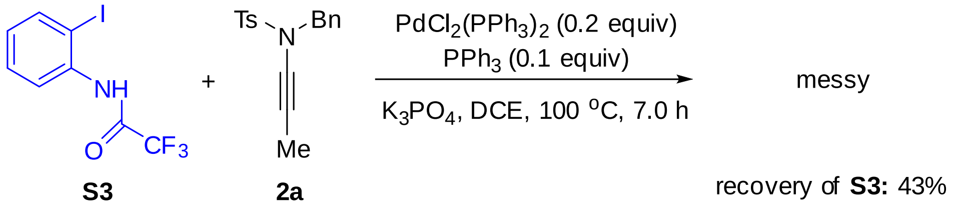

1.6 Failed Reactions

Attempted cross-coupling reactions of product 3da

Both Suzuki–Miyaura and Sonogashira cross-couplings on **3da** were attempted. In both cases, no desired cross-coupling product was detected. Instead, preferential cleavage of the *N*-acetyl group occurred, affording the deprotected 3-aminoindole in 43% yield (Suzuki attempt) and 38% yield (Sonogashira attempt), along with recovered starting material **3da** (55% and 42%, respectively).

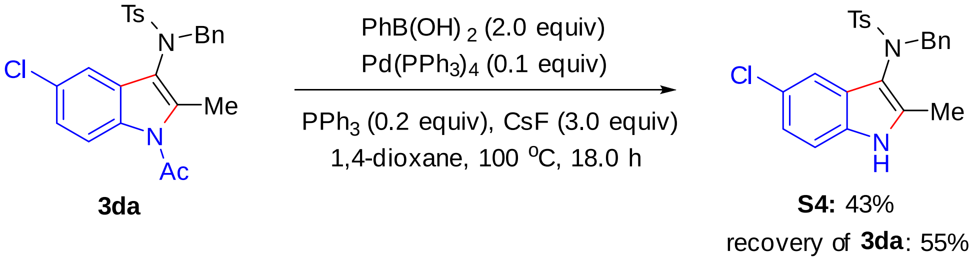

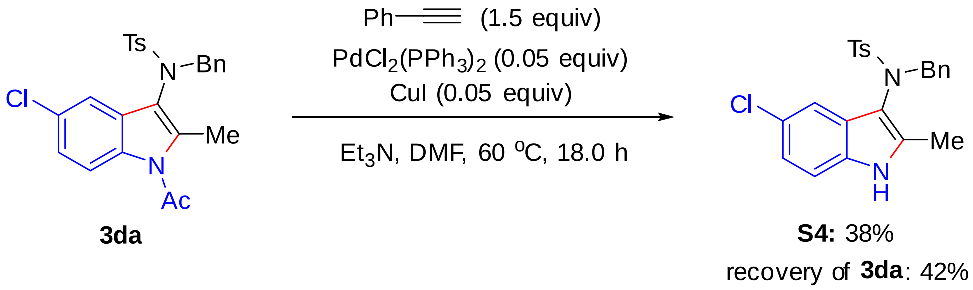

References

1 Y.-H. Qiu, P.-Y. Ma, W.-H. Shao, C.-Q. Huang, Y. Wen, Z.-Y. Huang, W. Luo, L. Long, X. Peng and D. Yu, Trifluoromethyl group (CF_3) induced regioselective Larock indole synthesis from unsymmetric β-CF_3-1,3-enynes, *Org. Lett.*, 2025, **27**, 3217–3224.

2 Y. Zhang, R. P. Hsung, M. R. Tracey, K. C. M. Kurtz and E. L. Vera, Copper sulfate-pentahydrate-1,10-phenanthroline catalyzed amidations of alkynyl bromides. Synthesis of

heteroaromatic amine substituted ynamides, *Org. Lett.*, 2004, **6**, 1151–1154.

3 Y. Yang, H. Liu, C. Peng, J. Wu, J. Zhang, Y. Qiao, X.-N. Wang and J. Chang, $AlCl_3$-Catalyzed annulations of ynamides involving a torquoselective process for the simultaneous control of central and axial chirality, *Org. Lett.*, 2016, **18**, 5022–5025.

4 H. Liu, Y. Yang, S. Wang, J. Wu, X.-N. Wang and J. Chang, Synthesis of 3-substituted 2-aminochromones via Sn(IV)-promoted annulation of ynamides with 2-methoxyaroyl chlorides, *Org. Lett.*, 2015, **17**, 4472–4475.

5 H. Li, R. P. Hsung, K. A. DeKorver and Y. Wei, Copper-catalyzed Ficini [2 + 2] cycloaddition of ynamides, *Org. Lett.*, 2010, **12**, 3780–3783.

6 S. J. Mansfield, C. D. Campbell, M. W. Jones and E. A. Anderson, A robust and modular synthesis of ynamides, *Chem. Commun.*, 2015, **51**, 3316–3319.

7 A. Coste, G. Karthikeyan, F. Couty and G. Evano, Copper-mediated coupling of 1,1-dibromo-1-alkenes with nitrogen nucleophiles: a general method for the synthesis of ynamides, *Angew. Chem. Int. Ed.*, 2009, **48**, 4381–4385.

8 X. Y. Mak, A. L. Crombie and R. L. Danheiser, Synthesis of polycyclic benzofused nitrogen heterocycles via a tandem ynamide benzannulation/ring-closing metathesis strategy. Application in a formal total synthesis of (+)-FR900482, *J. Org. Chem.*, 2011, **76**, 1852–1873.

9 Y. Tu, X. Zeng, H. Wang and J. Zhao, A robust one-step approach to ynamides, *Org. Lett.*, 2018, **20**, 280–283.

10 S. J. Mansfield, C. D. Campbell, M. W. Jones and E. A. Anderson, A robust and modular synthesis of ynamides, *Chem. Commun.*, 2015, **51**, 3316–3319.

11 X. Peng, J. Zhao, G. Ma, Y. Wu, S. Hu, Z. Ruan and P. Feng, Electro-oxidative C–H amination of heteroarenes with aniline derivatives *via* radical–radical cross coupling, *Green Chem.*, 2021, **23**, 8853–8858.

12 Y. Zhao, Y. Hu, C. Wang, X. Li and B. Wan, Tf_2NH-Catalyzed formal [3 + 2] cycloaddition of ynamides with dioxazoles: a metal-free approach to polysubstituted 4-aminooxazoles, *J. Org. Chem.*, 2017, **82**, 3935–3942.

13 N. Chaisan, W. Kaewsri, C. Thongsornkleeb, J. Tummatorn and S. Ruchirawat, $PtCl_4$-catalyzed cyclization of *N*-acetyl-2-alkynylanilines: A mild and efficient synthesis of *N*-acetyl-2-substituted indoles, *Tetrahedron Lett.*, 2018, **59**, 675–680.

14 Z.-l. Li, K.-k. Sun and C. Cai, Cobalt(ii)-catalyzed regioselective C–H halogenation of anilides, *Org. Biomol. Chem.*, 2018, **16**, 5433–5440.

15 S. Wu, W.-Y. Hu and S.-L. Zhang, Potassium carbonate-mediated tandem C–S and C–N coupling reaction for the synthesis of phenothiazines under transition-metal-free and ligand-free conditions, *RSC Adv.*, 2016, **6**, 24257–24260.

16 S. Redon, Y. Kabri, M. D. Crozet and P. Vanelle, One-pot preparation of 2-(alkyl)arylbenzoselenazoles from the corresponding *N*-(acetyl)benzoyl-2-iodoanilines via a microwave-assisted methodology, *Tetrahedron Lett.*, 2014, **55**, 5052–5054.

17 Y. P. Bogza, A. A. Rastrepin, V. V. Nider, T. Y. Zheleznova, A. J. Stasyuk, A. Kurowska, K. Laba, E. B. Ulyankin, W. Domagala and A. S. Fisyuk, Synthesis and optical properties of 2-functionally substituted 4,5-dihydrothieno[3,2-*c*]quinolines, *Dyes and Pigments*, 2018, **159**, 419–428.

18 F. Ujjainwalla, M. L. E. N. da Mata, A. M. K. Pennell, C. Escolano, W. B. Motherwell and S. Vázquez, Synthesis of biaryls via intramolecular free radical *ipso*-substitution reactions, *Tetrahedron*, 2015, **71**, 6701–6719.

19 A. J. McCarroll, T. D. Bradshaw, A. D. Westwell, C. S. Matthews and M. F. G. Stevens, Quinols as novel

therapeutic agents. 7. Synthesis of antitumor 4-[1-(Arylsulfonyl-1*H*-indol-2-yl)]-4-hydroxycyclohexa-2,5-dien-1-ones by Sonogashira reactions, *J. Med. Chem.*, 2007, **50**, 1707–1710.

20 A. Le Pera, A. Leggio and A. Liguori, Highly specific *N*-monomethylation of primary aromatic amines, *Tetrahedron*, 2006, **62**, 6100–6106.

21 S. E. Kiruthika and P. T. Perumal, CuI-Catalyzed coupling of *gem*-dibromovinylanilides and sulfonamides: An efficient method for the synthesis of 2-amidoindoles and indolo[1,2-*a*]quinazolines, *Org. Lett.*, 2014, **16**, 484–487.

22 H. Clavier, A. Lepronier, N. Bengobesse-Mintsa, D. Gatineau, H. Pellissier, L. Giordano, A. Tenaglia and G. Buono, Palladium-mediated [2 + 1] cycloaddition of norbornene derivatives with ynamides, *Adv. Synth. Catal.*, 2013, **355**, 403–408.

23 Q. Han, K. Xu, F. Tian, S. Huang and C. Zeng, A practical transamidation strategy for the N-deacylation of amides, *Chin. J. Org. Chem.*, 2022, **42**, 1123–1128.

24 L. Jiao, Y. Wang, L. Ding, C. Zhang, X.-N. Wang and J. Chang, Synthesis of 2-aminopyrroles via metal-free annulation of ynamides with 2*H*-azirines, *J. Org. Chem.*, 2022, **87**, 15564–15570.

Part II Copies of ^{1}H NMR, ^{13}C NMR, and ^{19}F NMR Spectra.

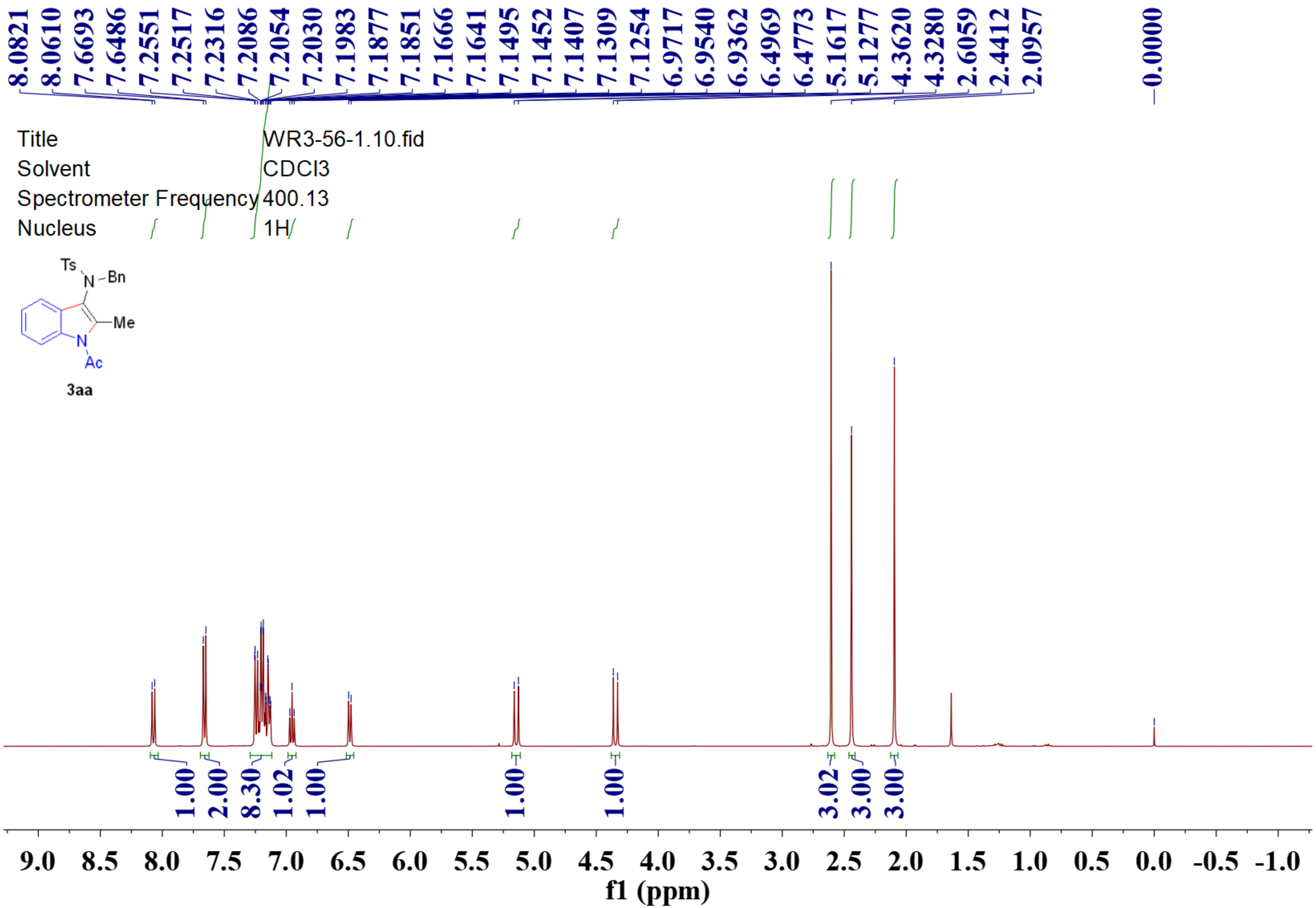

Title WR3-36-1.10.fid
Solvent CDCl3
Spectrometer Frequency 100.62
Nucleus 13C

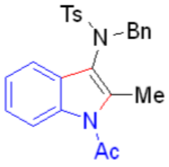

3aa

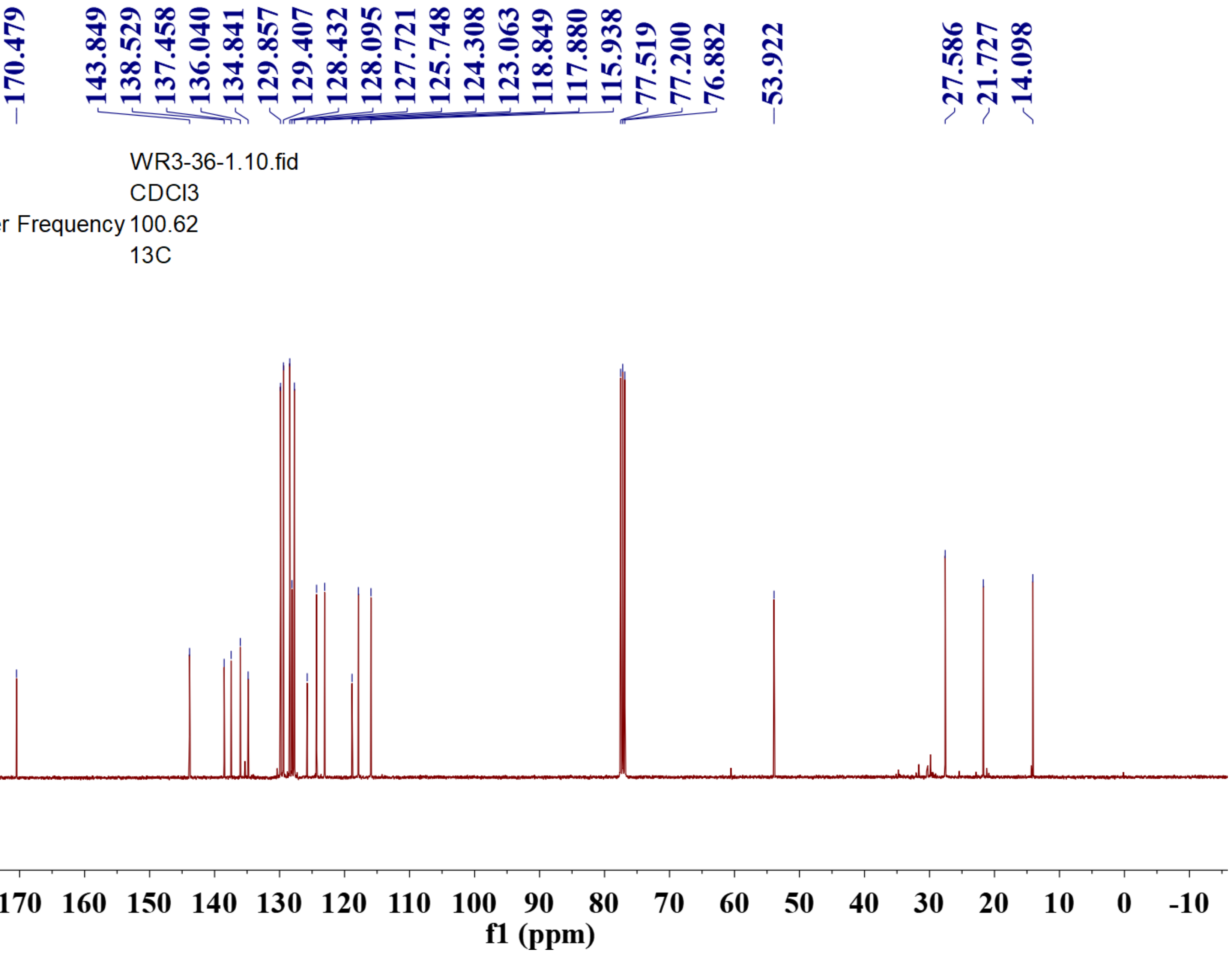

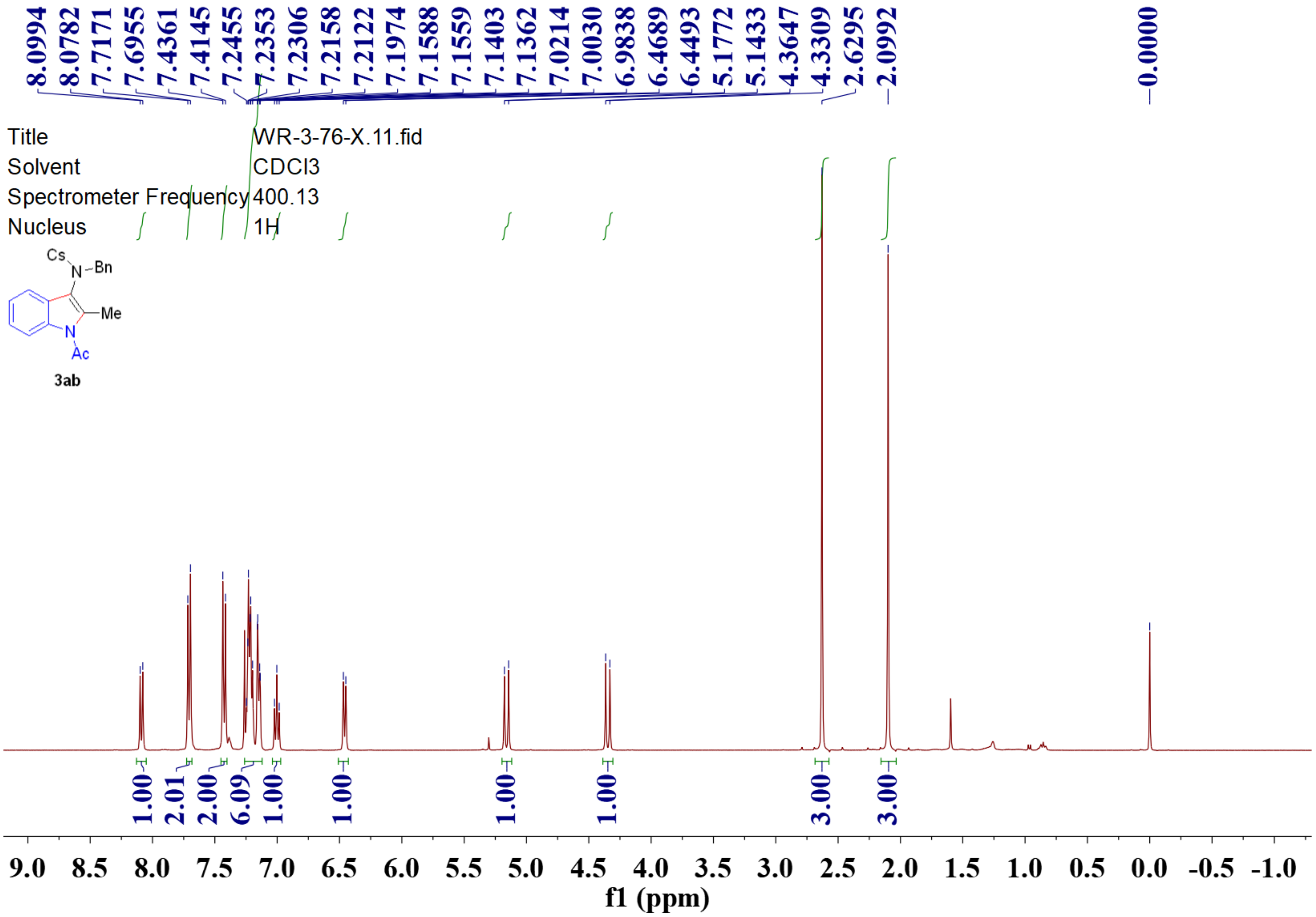
8.0994
8.0782
7.7171
7.6955
7.4361
7.4145
7.2455
7.2353
7.2306
7.2158
7.2122
7.1974
7.1588
7.1559
7.1403
7.1362
7.0214
7.0030
6.9838
6.4689
6.4493
5.1772
5.1433
4.3647
4.3309
2.6295
2.0992
0.0000
Title WR-3-76-X.11.fid
Solvent CDCl3
Spectrometer Frequency 400.13
Nucleus 1H
Cs
N
Bn
Me
N
Ac
3ab
1.00
2.01
2.00
6.09
1.00
1.00
1.00
1.00
3.00
3.00
9.0 8.5 8.0 7.5 7.0 6.5 6.0 5.5 5.0 4.5 4.0 3.5 3.0 2.5 2.0 1.5 1.0 0.5 0.0 -0.5 -1.0
f1 (ppm)

Title WR-3-76-X.10.fid
Solvent CDCl3
Spectrometer Frequency 100.62
Nucleus 13C

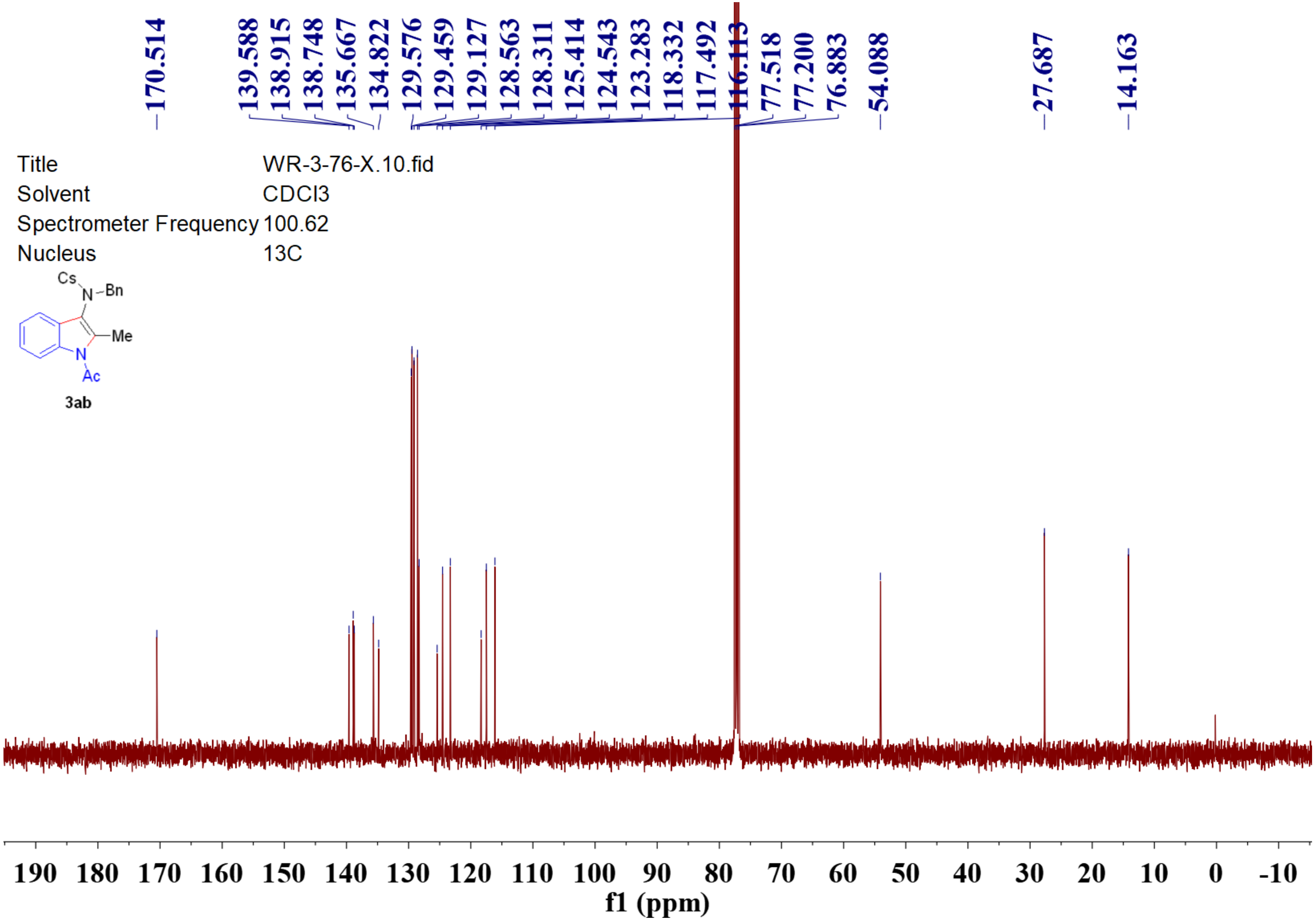

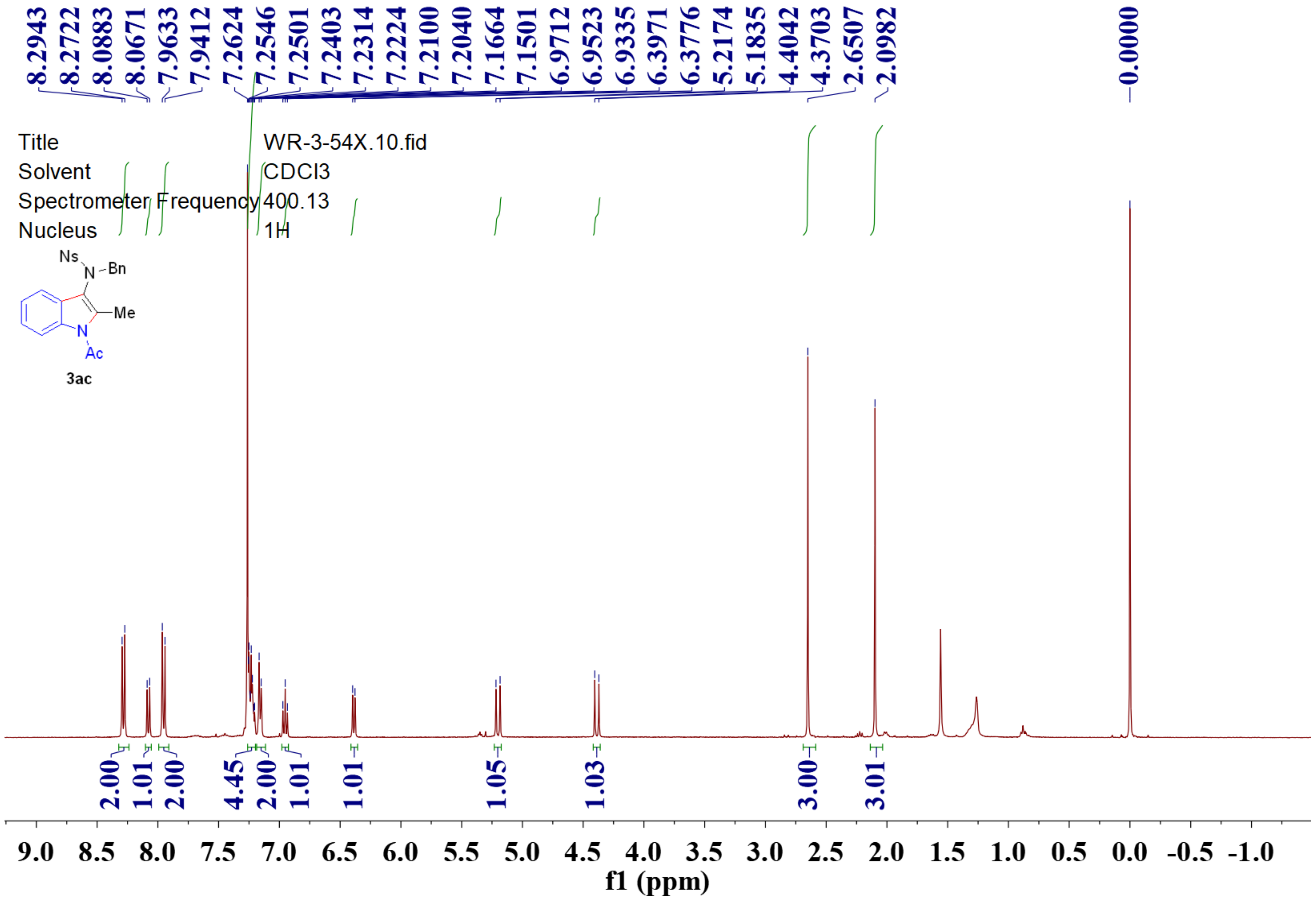

8.2943
8.2722
8.0883
8.0671
7.9633
7.9412
7.2624
7.2546
7.2501
7.2403
7.2314
7.2224
7.2100
7.2040
7.1664
7.1501
6.9712
6.9523
6.9335
6.3971
6.3776
5.2174
5.1835
4.4042
4.3703
2.6507
2.0982
-0.0000
Title WR-3-54X.10.fid
Solvent CDCl3
Spectrometer Frequency 400.13
Nucleus 1H
Ns
N
Bn
Me
N
Ac
3ac
2.00
1.01
2.00
4.45
2.00
1.01
1.01
1.05
1.03
3.00
3.01
9.0 8.5 8.0 7.5 7.0 6.5 6.0 5.5 5.0 4.5 4.0 3.5 3.0 2.5 2.0 1.5 1.0 0.5 0.0 -0.5 -1.0
f1 (ppm)

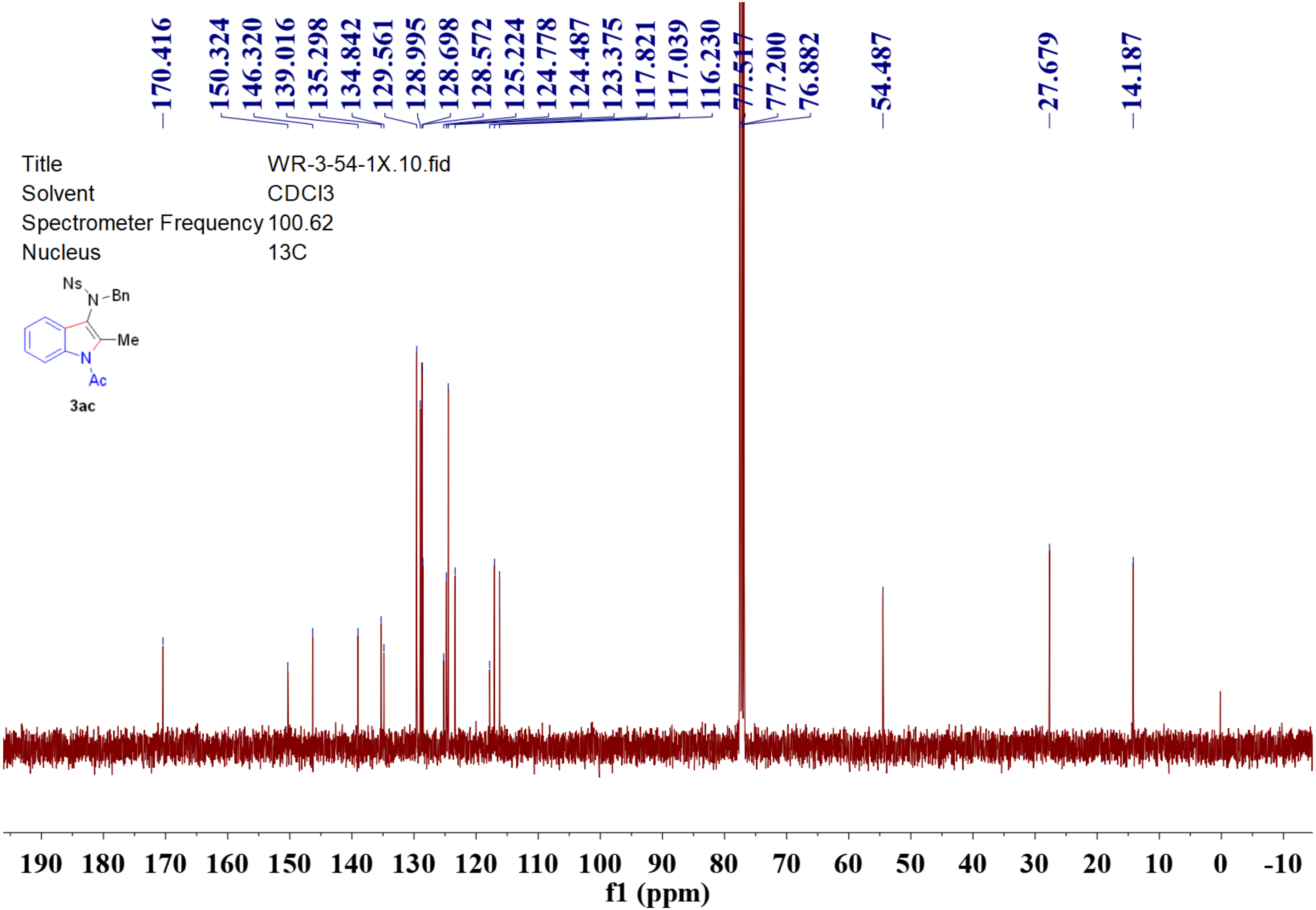

170.416
150.324
146.320
139.016
135.298
134.842
129.561
128.995
128.698
128.572
125.224
124.778
124.487
123.375
117.821
117.039
116.230
77.517
77.200
76.882
54.487
27.679
14.187
Title WR-3-54-1X.10.fid
Solvent CDCl3
Spectrometer Frequency 100.62
Nucleus 13C
Ns
N
Bn
Me
N
Ac
3ac
190 180 170 160 150 140 130 120 110 100 90 80 70 60 50 40 30 20 10 0 -10
f1 (ppm)

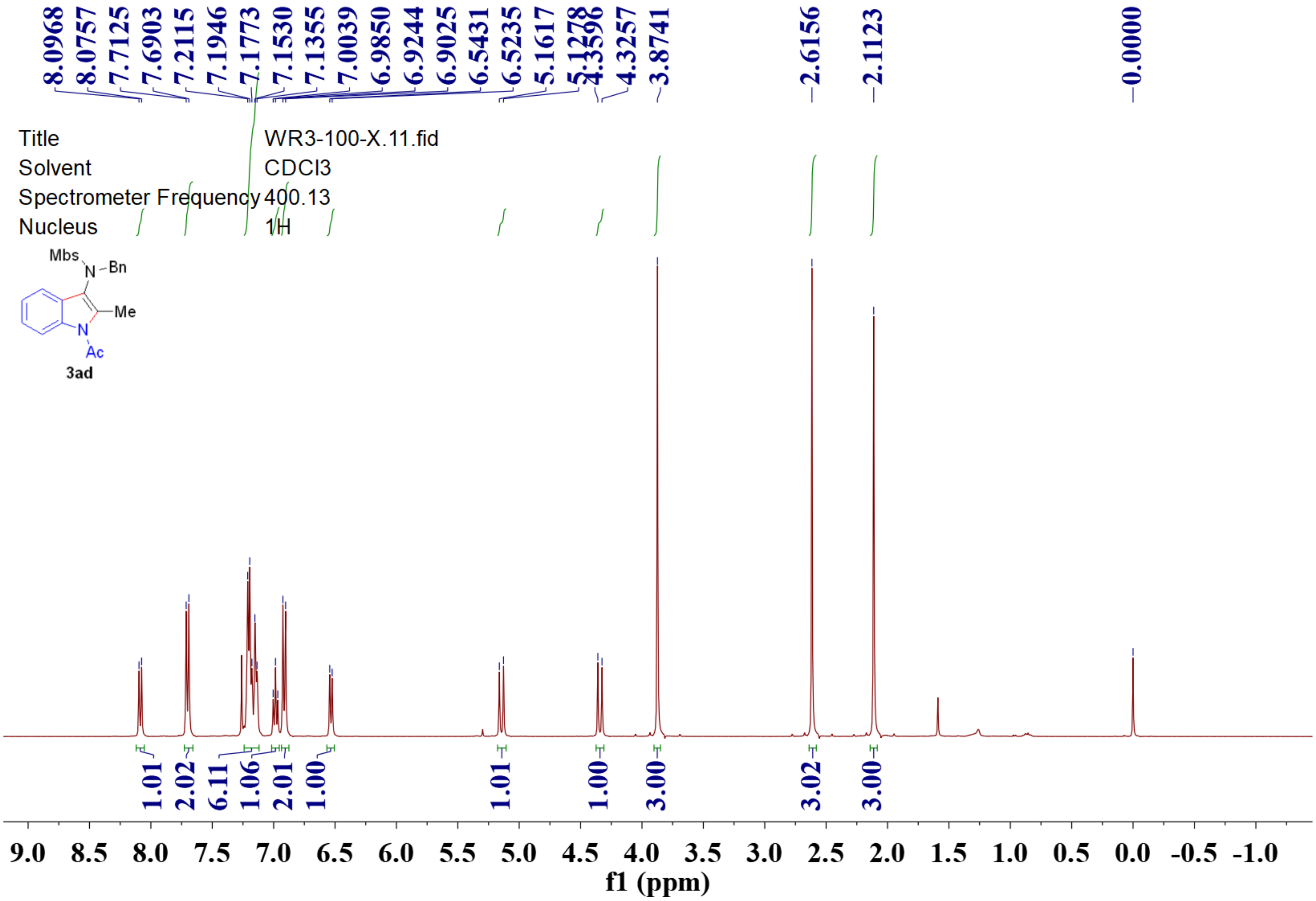
8.0968
8.0757
7.7125
7.6903
7.2115
7.1946
7.1773
7.1530
7.1355
7.0039
6.9850
6.9244
6.9025
6.5431
6.5235
5.1617
5.1278
4.3596
4.3257
3.8741
2.6156
2.1123
0.0000
Title WR3-100-X.11.fid
Solvent CDCl3
Spectrometer Frequency 400.13
Nucleus 1H
Mbs
N
Bn
Me
N
Ac
3ad
1.01
2.02
6.11
1.06
2.01
1.00
1.01
1.00
3.00
3.02
3.00
9.0 8.5 8.0 7.5 7.0 6.5 6.0 5.5 5.0 4.5 4.0 3.5 3.0 2.5 2.0 1.5 1.0 0.5 0.0 -0.5 -1.0
f1 (ppm)

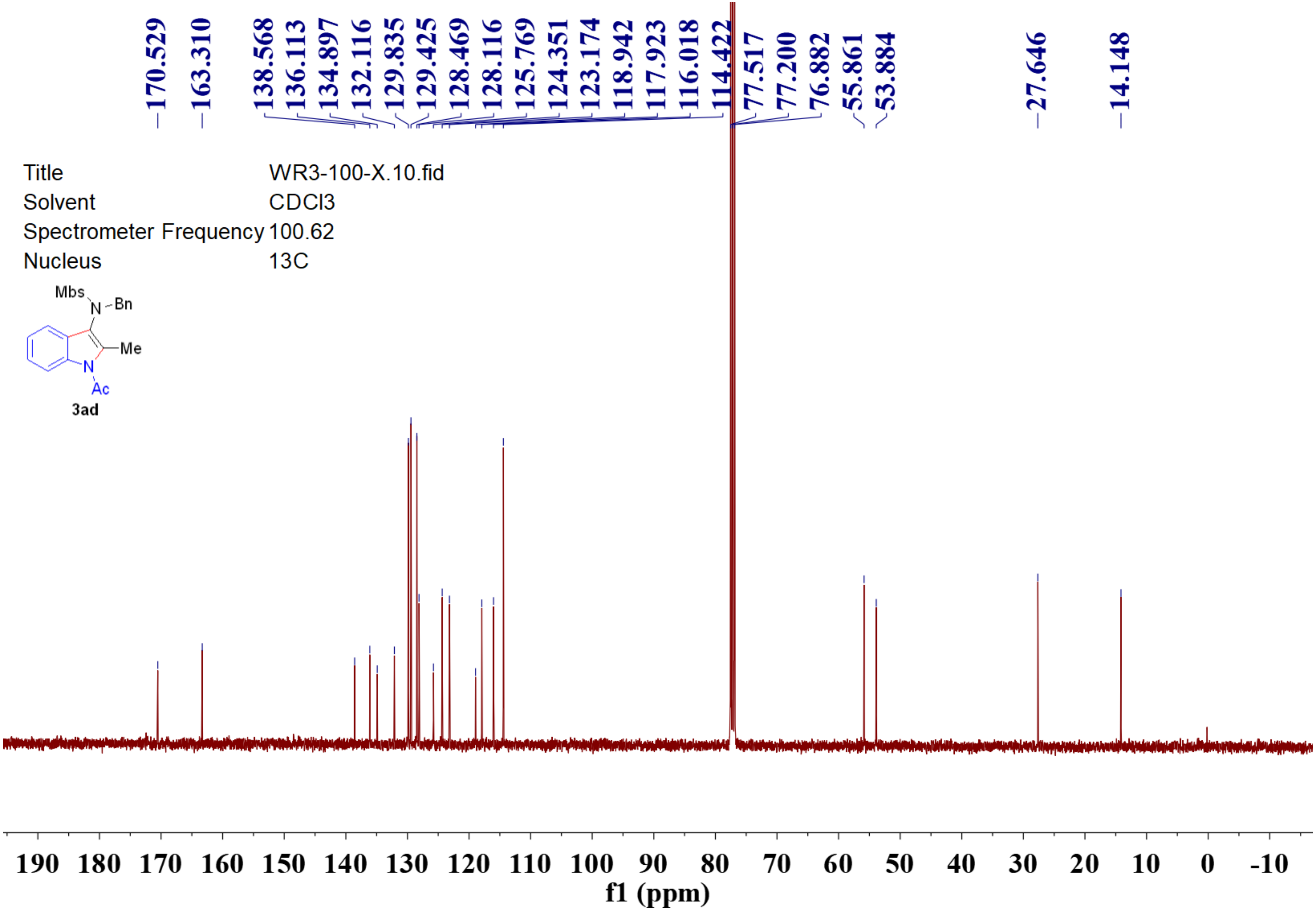
170.529
163.310
138.568
136.113
134.897
132.116
129.835
129.425
128.469
128.116
125.769
124.351
123.174
118.942
117.923
116.018
114.422
77.517
77.200
76.882
55.861
53.884
27.646
14.148
Title WR3-100-X.10.fid
Solvent CDCl3
Spectrometer Frequency 100.62
Nucleus 13C
Mbs
N
Bn
Me
N
Ac
3ad
190 180 170 160 150 140 130 120 110 100 90 80 70 60 50 40 30 20 10 0 -10
f1 (ppm)

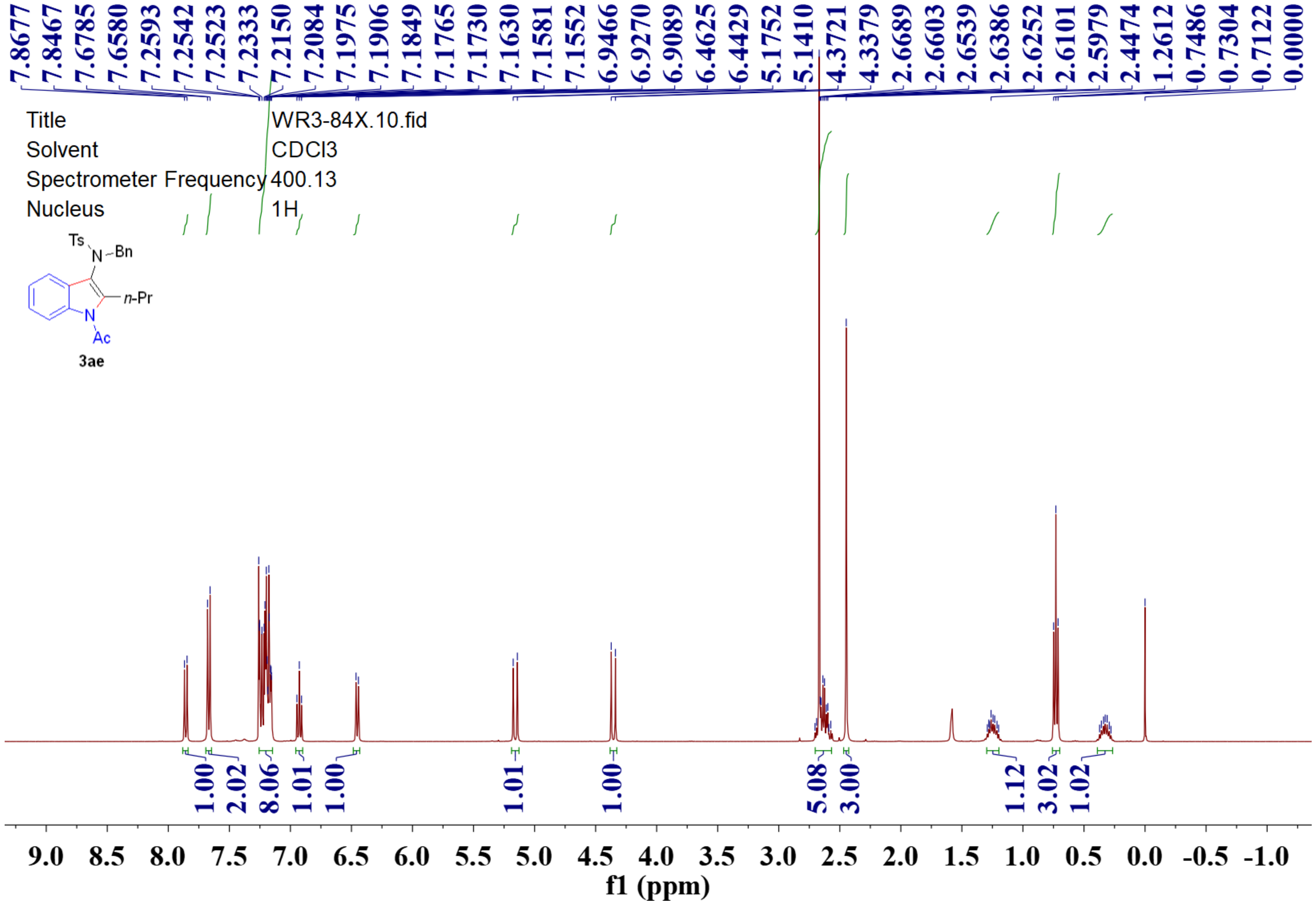

7.8677
7.8467
7.6785
7.6580
7.2593
7.2542
7.2523
7.2333
7.2150
7.2084
7.1975
7.1906
7.1849
7.1765
7.1730
7.1630
7.1581
7.1552
6.9466
6.9270
6.9089
6.4625
6.4429
5.1752
5.1410
4.3721
4.3379
2.6689
2.6603
2.6539
2.6386
2.6252
2.6101
2.5979
2.4474
1.2612
0.7486
0.7304
0.7122
0.0000
Title WR3-84X.10.fid
Solvent CDCl3
Spectrometer Frequency 400.13
Nucleus 1H
Ts
N
Bn
n-Pr
N
Ac
3ae
1.00
2.02
8.06
1.01
1.00
1.01
1.00
5.08
3.00
1.12
3.02
1.02
9.0 8.5 8.0 7.5 7.0 6.5 6.0 5.5 5.0 4.5 4.0 3.5 3.0 2.5 2.0 1.5 1.0 0.5 0.0 -0.5 -1.0
f1 (ppm)

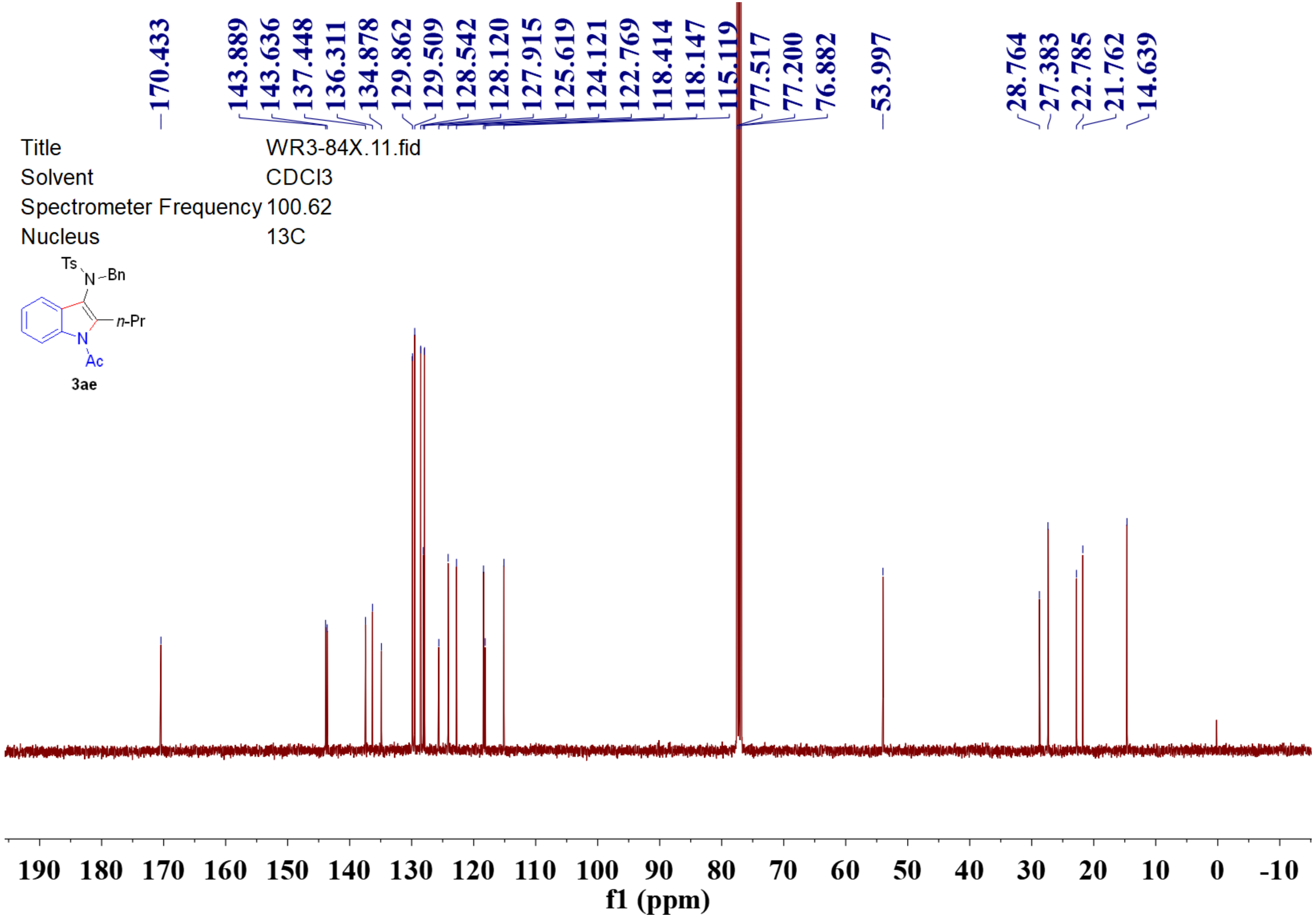

170.433
143.889
143.636
137.448
136.311
134.878
129.862
129.509
128.542
128.120
127.915
125.619
124.121
122.769
118.414
118.147
115.119
77.517
77.200
76.882
53.997
28.764
27.383
22.785
21.762
14.639
Title WR3-84X.11.fid
Solvent CDCl3
Spectrometer Frequency 100.62
Nucleus 13C
190 180 170 160 150 140 130 120 110 100 90 80 70 60 50 40 30 20 10 0 -10
f1 (ppm)
Ts
N
Bn
n-Pr
N
Ac

3ae

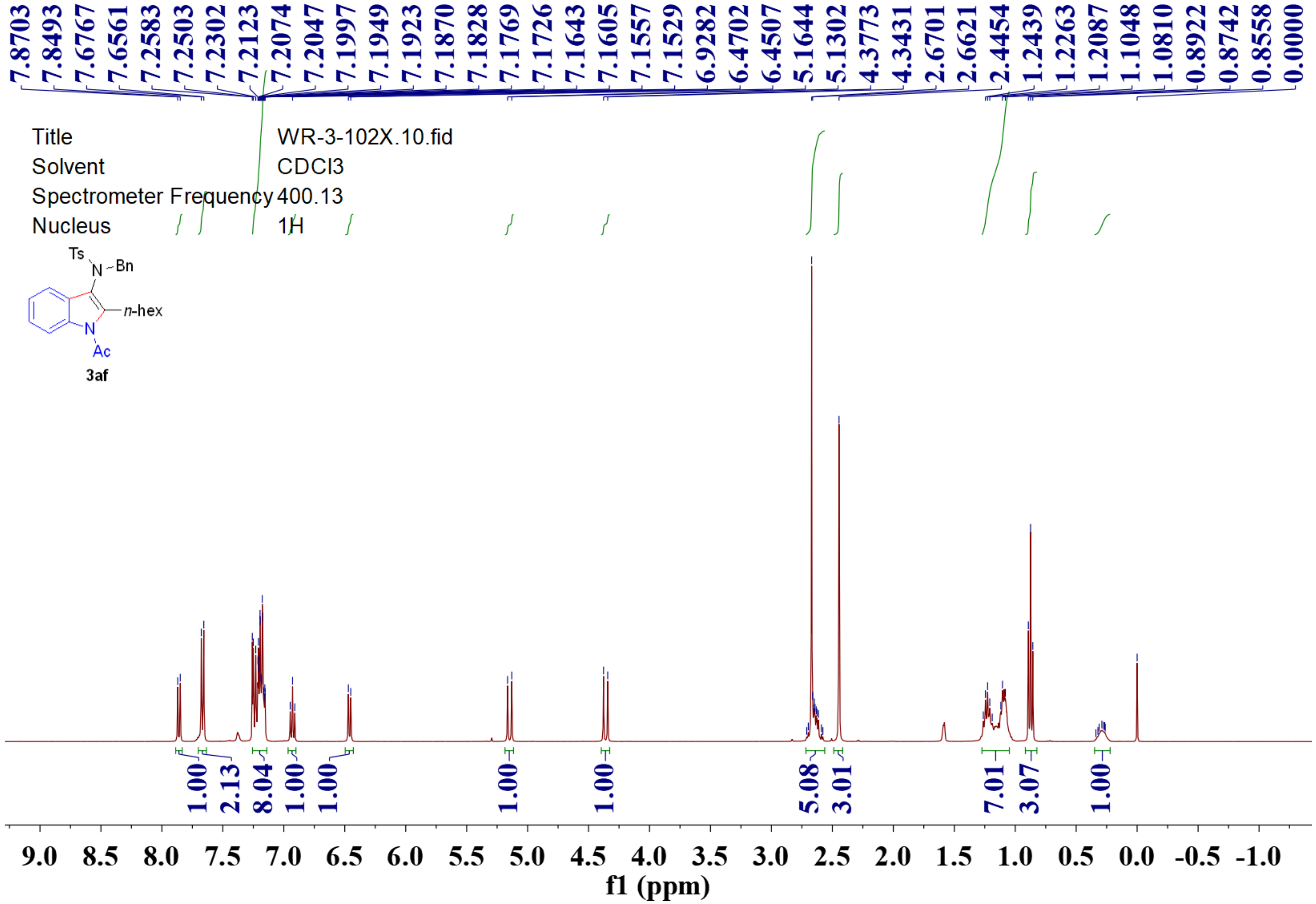

7.8703
7.8493
7.6767
7.6561
7.2583
7.2503
7.2302
7.2123
7.2074
7.2047
7.1997
7.1949
7.1923
7.1870
7.1828
7.1769
7.1726
7.1643
7.1605
7.1557
7.1529
6.9282
6.4702
6.4507
5.1644
5.1302
4.3773
4.3431
2.6701
2.6621
2.4454
1.2439
1.2263
1.2087
1.1048
1.0810
0.8922
0.8742
0.8558
0.0000
Title WR-3-102X.10.fid
Solvent CDCl3
Spectrometer Frequency 400.13
Nucleus 1H
Ts
N
Bn
n-hex
N
Ac
3af
1.00
2.13
8.04
1.00
1.00
1.00
1.00
5.08
3.01
7.01
3.07
1.00
9.0 8.5 8.0 7.5 7.0 6.5 6.0 5.5 5.0 4.5 4.0 3.5 3.0 2.5 2.0 1.5 1.0 0.5 0.0 -0.5 -1.0
f1 (ppm)

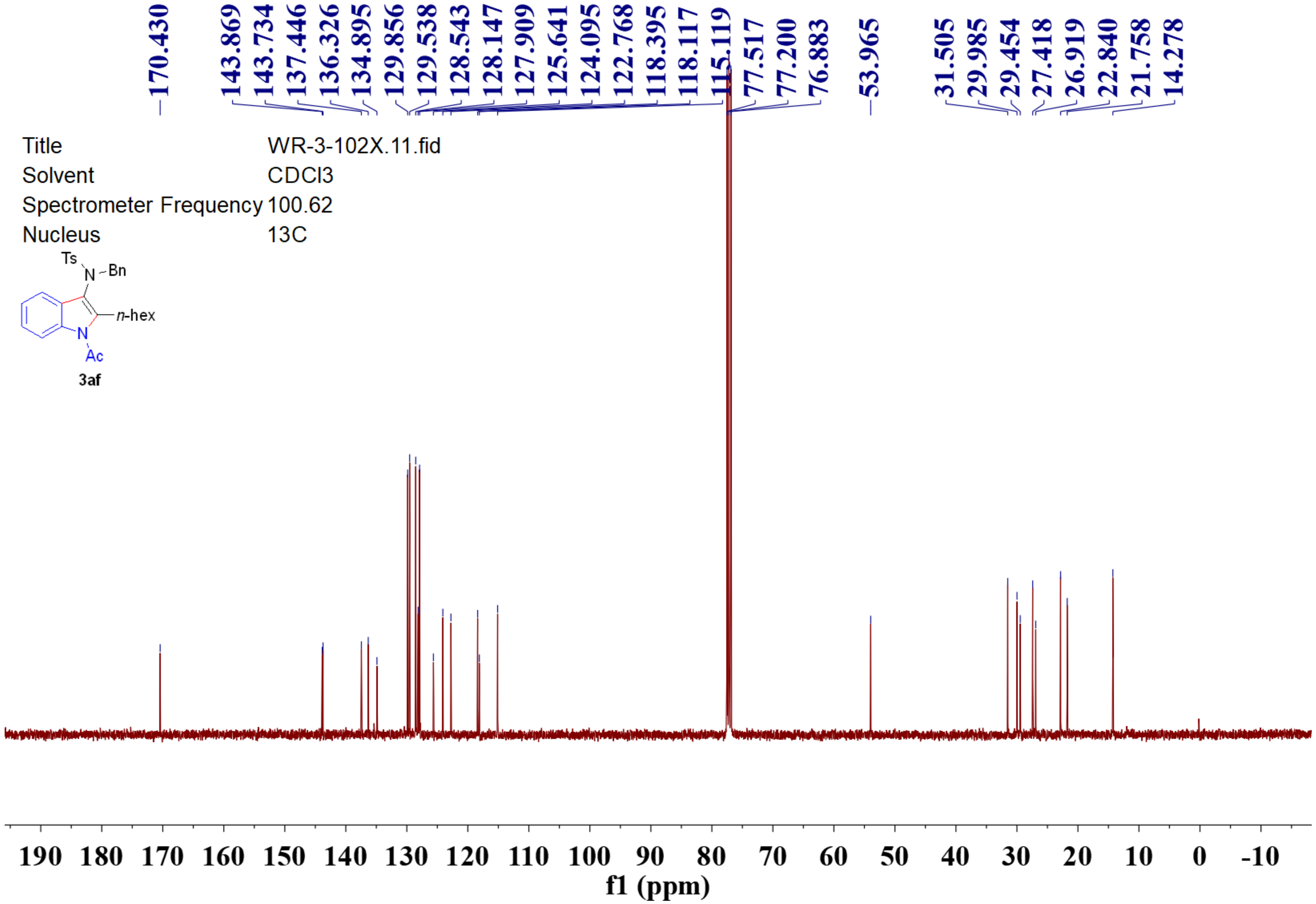

170.430
143.869
143.734
137.446
136.326
134.895
129.856
129.538
128.543
128.147
127.909
125.641
124.095
122.768
118.395
118.117
115.119
77.517
77.200
76.883
53.965
31.505
29.985
29.454
27.418
26.919
22.840
21.758
14.278
Title WR-3-102X.11.fid
Solvent CDCl3
Spectrometer Frequency 100.62
Nucleus 13C
Ts
N
Bn
n-hex
N
Ac
3af
190 180 170 160 150 140 130 120 110 100 90 80 70 60 50 40 30 20 10 0 -10
f1 (ppm)

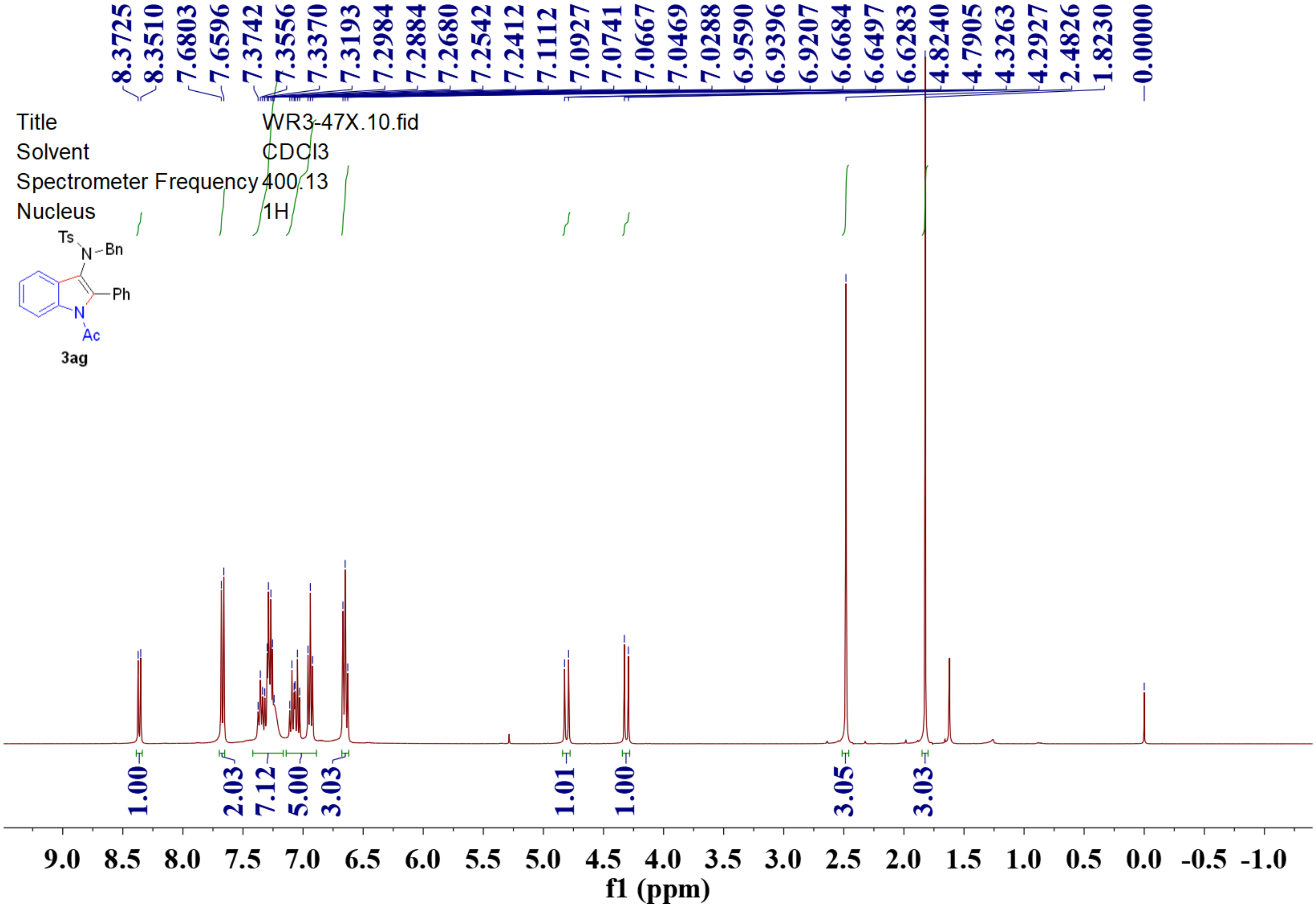
8.3725
8.3510
7.6803
7.6596
7.3742
7.3556
7.3370
7.3193
7.2984
7.2884
7.2680
7.2542
7.2412
7.1112
7.0927
7.0741
7.0667
7.0469
7.0288
6.9590
6.9396
6.9207
6.6684
6.6497
6.6283
4.8240
4.7905
4.3263
4.2927
2.4826
1.8230
-0.0000
Title WR3-47X.10.fid
Solvent CDCl3
Spectrometer Frequency 400.13
Nucleus 1H
Ts
N
Bn
Ph
N
Ac
3ag
1.00
2.03
7.12
5.00
3.03
1.01
1.00
3.05
3.03
9.0 8.5 8.0 7.5 7.0 6.5 6.0 5.5 5.0 4.5 4.0 3.5 3.0 2.5 2.0 1.5 1.0 0.5 0.0 -0.5 -1.0
f1 (ppm)

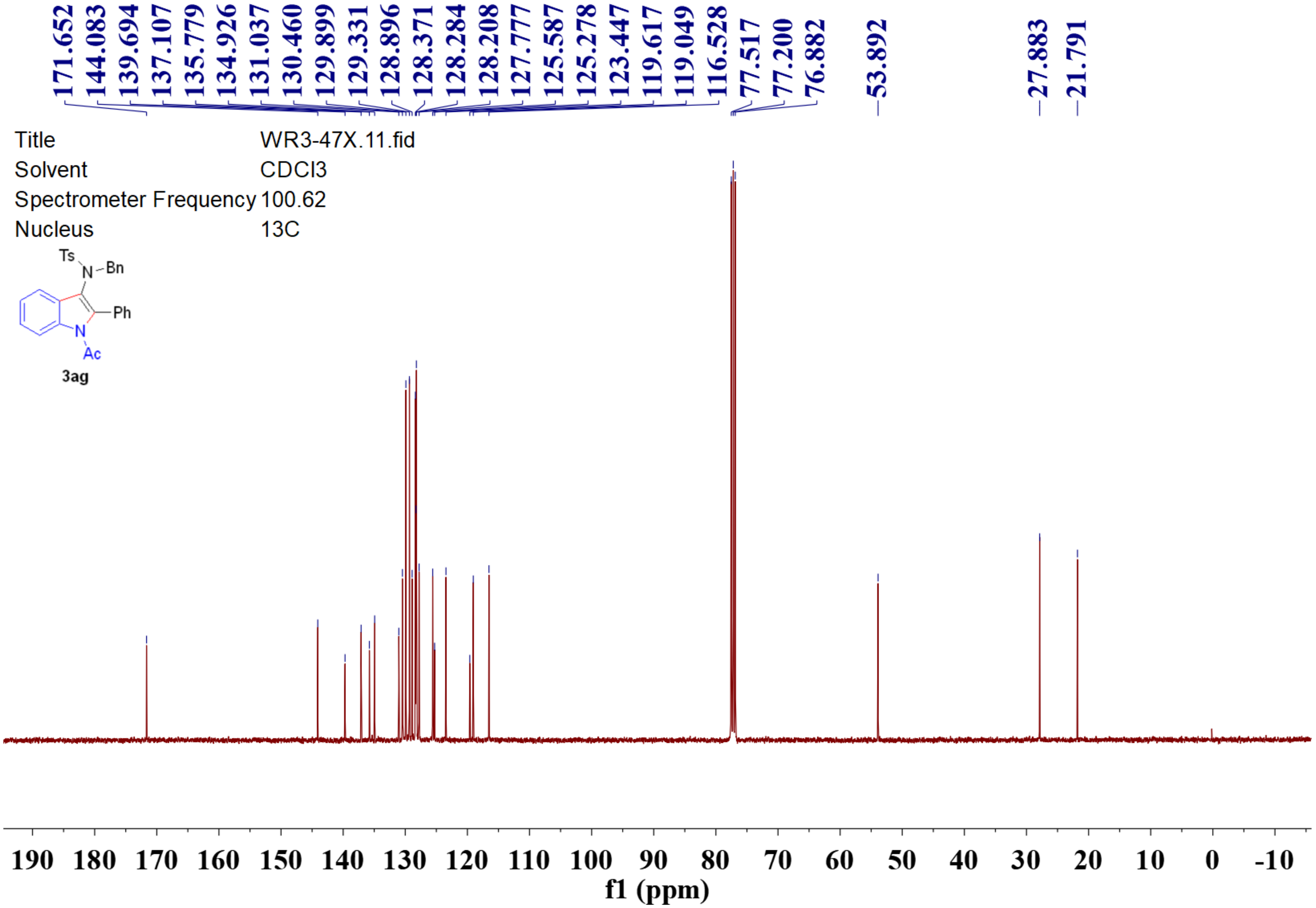
171.652
144.083
139.694
137.107
135.779
134.926
131.037
130.460
129.899
129.331
128.896
128.371
128.284
128.208
127.777
125.587
125.278
123.447
119.617
119.049
116.528
77.517
77.200
76.882
53.892
27.883
21.791
Title WR3-47X.11.fid
Solvent CDCl3
Spectrometer Frequency 100.62
Nucleus 13C
190 180 170 160 150 140 130 120 110 100 90 80 70 60 50 40 30 20 10 0 -10
f1 (ppm)
Ts
N
Bn
Ph
N
Ac

3ag

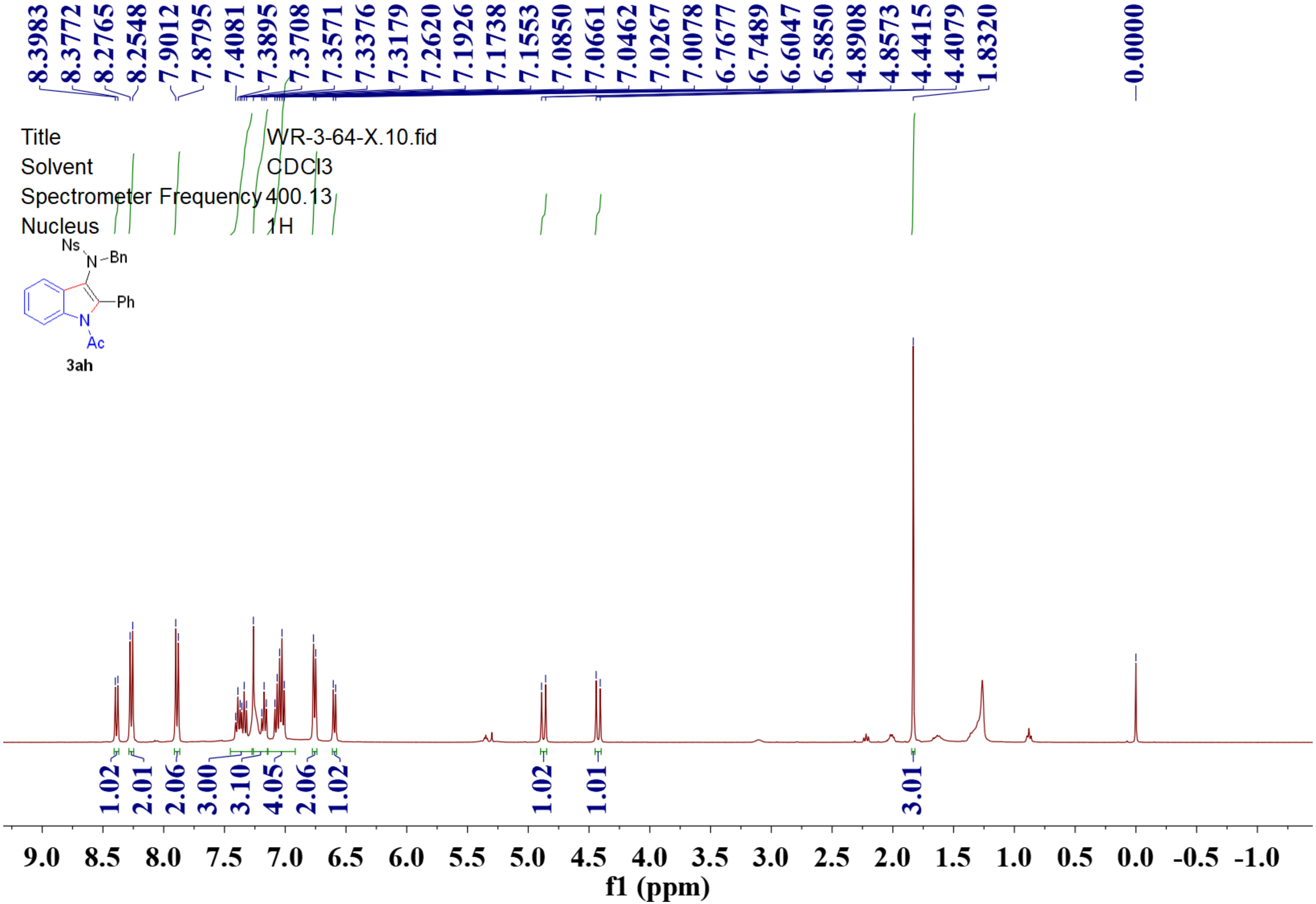
8.3983
8.3772
8.2765
8.2548
7.9012
7.8795
7.4081
7.3895
7.3708
7.3571
7.3376
7.3179
7.2620
7.1926
7.1738
7.1553
7.0850
7.0661
7.0462
7.0267
7.0078
6.7677
6.7489
6.6047
6.5850
4.8908
4.8573
4.4415
4.4079
1.8320
-0.0000
Title WR-3-64-X.10.fid
Solvent CDCl3
Spectrometer Frequency 400.13
Nucleus 1H
Ns
N
Bn
Ph
N
Ac
3ah
1.02
2.01
2.06
3.00
3.10
4.05
2.06
1.02
1.02
1.01
3.01
9.0 8.5 8.0 7.5 7.0 6.5 6.0 5.5 5.0 4.5 4.0 3.5 3.0 2.5 2.0 1.5 1.0 0.5 0.0 -0.5 -1.0
f1 (ppm)

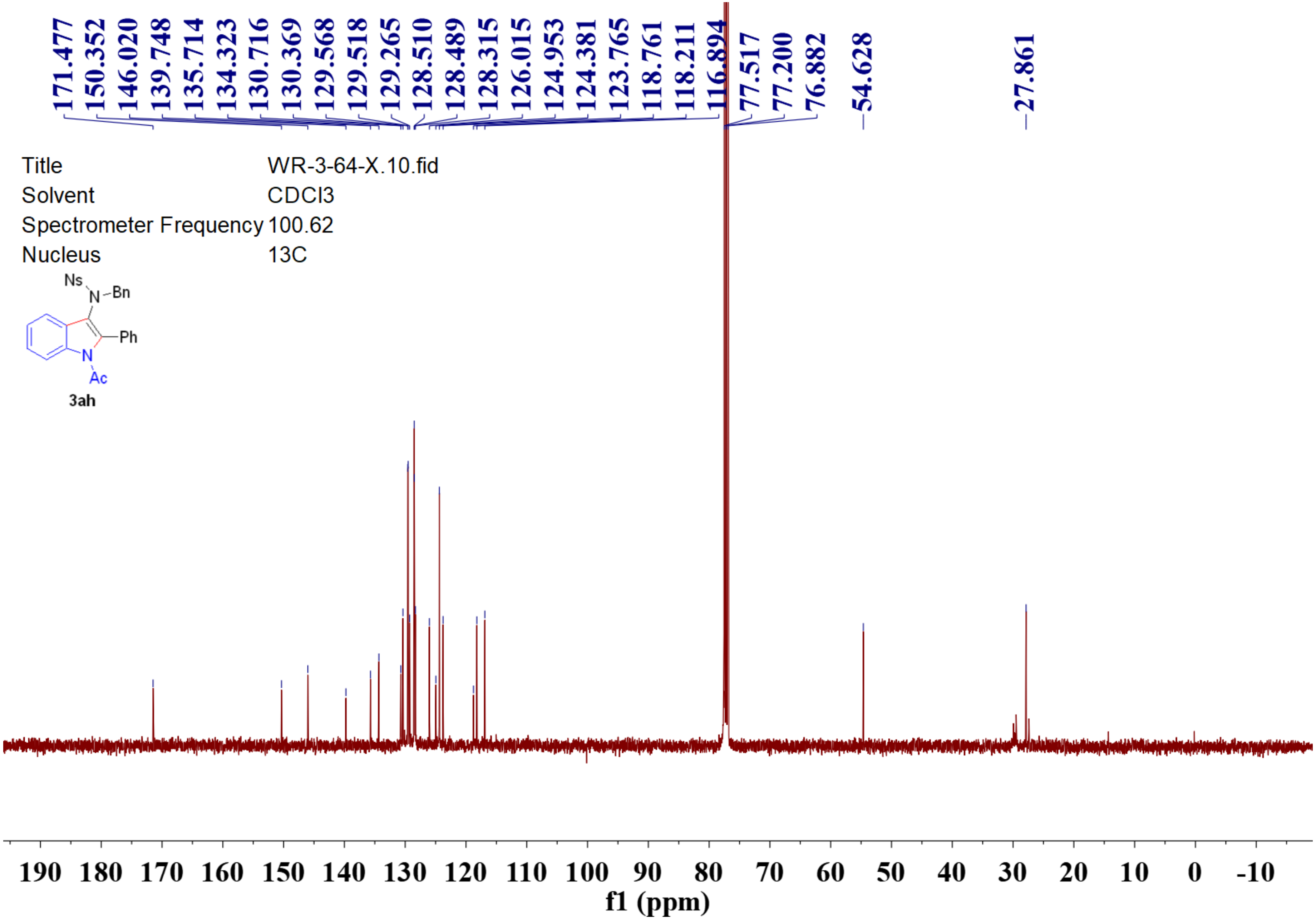
171.477
150.352
146.020
139.748
135.714
134.323
130.716
130.369
129.568
129.518
129.265
128.510
128.489
128.315
126.015
124.953
124.381
123.765
118.761
118.211
116.894
77.517
77.200
76.882
54.628
27.861
Title WR-3-64-X.10.fid
Solvent CDCl3
Spectrometer Frequency 100.62
Nucleus 13C
190 180 170 160 150 140 130 120 110 100 90 80 70 60 50 40 30 20 10 0 -10
f1 (ppm)
Ns
N
Bn
Ph
N
Ac

3ah

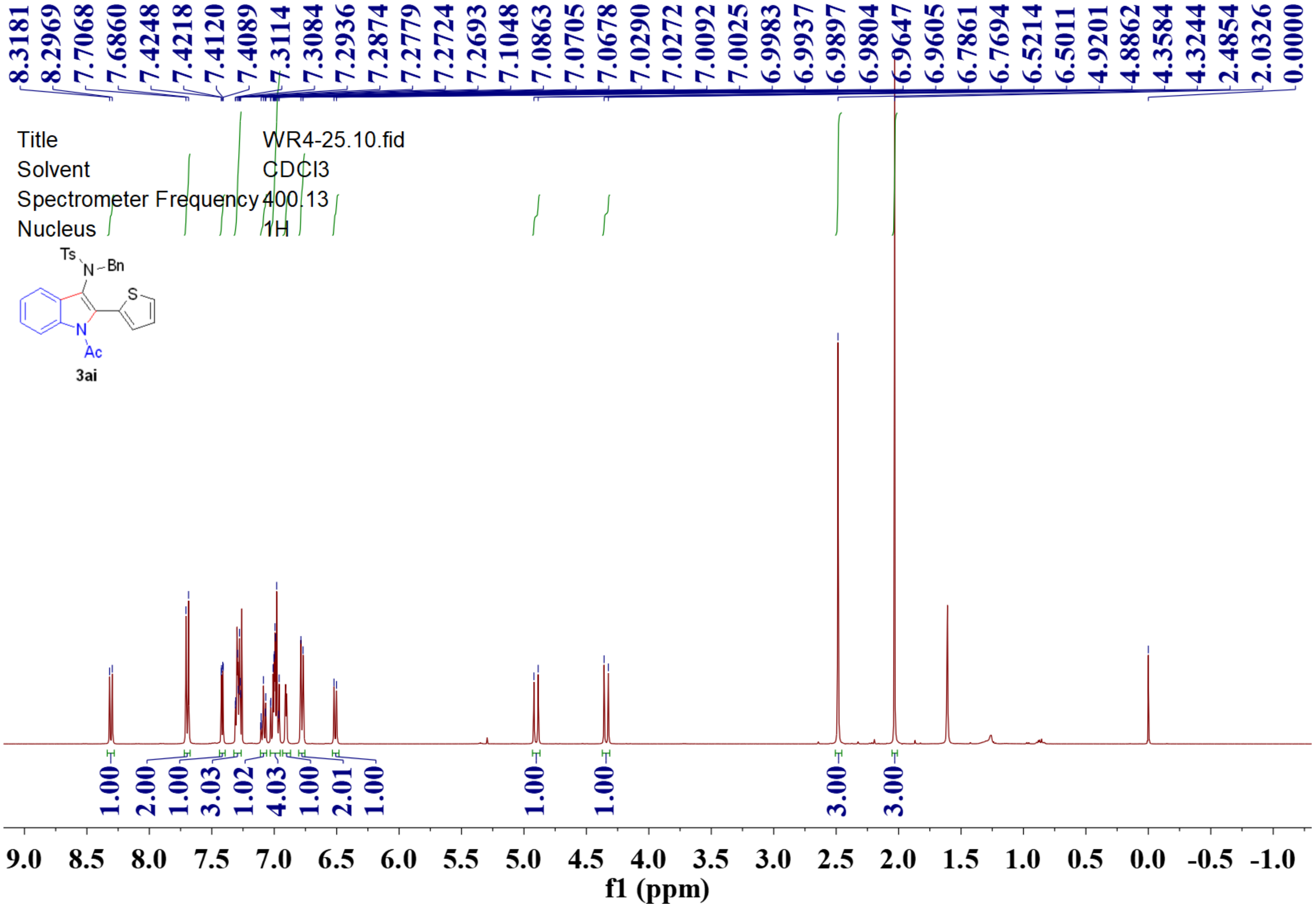
8.3181
8.2969
7.7068
7.6860
7.4248
7.4218
7.4120
7.4089
7.3114
7.3084
7.2936
7.2874
7.2779
7.2724
7.2693
7.1048
7.0863
7.0705
7.0678
7.0290
7.0272
7.0092
7.0025
6.9983
6.9937
6.9897
6.9804
6.9647
6.9605
6.7861
6.7694
6.5214
6.5011
4.9201
4.8862
4.3584
4.3244
2.4854
2.0326
0.0000
Title WR4-25.10.fid
Solvent CDCl3
Spectrometer Frequency 400.13
Nucleus 1H
Ts
N
Bn
S
N
Ac
3ai
1.00
2.00
1.00
3.03
1.02
4.03
1.00
2.01
1.00
1.00
1.00
3.00
3.00
9.0 8.5 8.0 7.5 7.0 6.5 6.0 5.5 5.0 4.5 4.0 3.5 3.0 2.5 2.0 1.5 1.0 0.5 0.0 -0.5 -1.0
f1 (ppm)

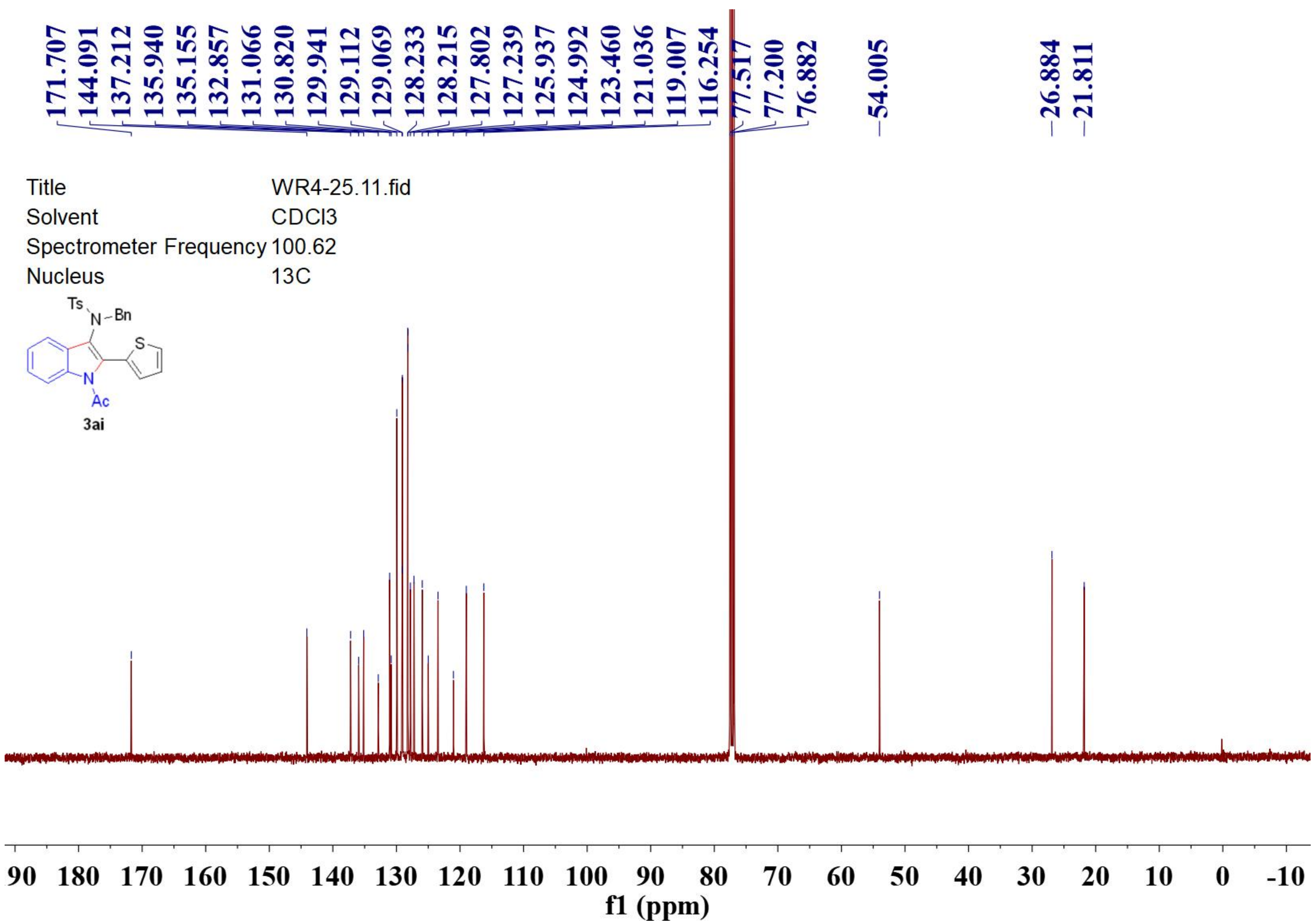

171.707
144.091
137.212
135.940
135.155
132.857
131.066
130.820
129.941
129.112
129.069
128.233
128.215
127.802
127.239
125.937
124.992
123.460
121.036
119.007
116.254
77.517
77.200
76.882
54.005
26.884
21.811
Title WR4-25.11.fid
Solvent CDCl3
Spectrometer Frequency 100.62
Nucleus 13C
Ts
N
Bn
S
N
Ac
3ai
90 180 170 160 150 140 130 120 110 100 90 80 70 60 50 40 30 20 10 0 -10
f1 (ppm)

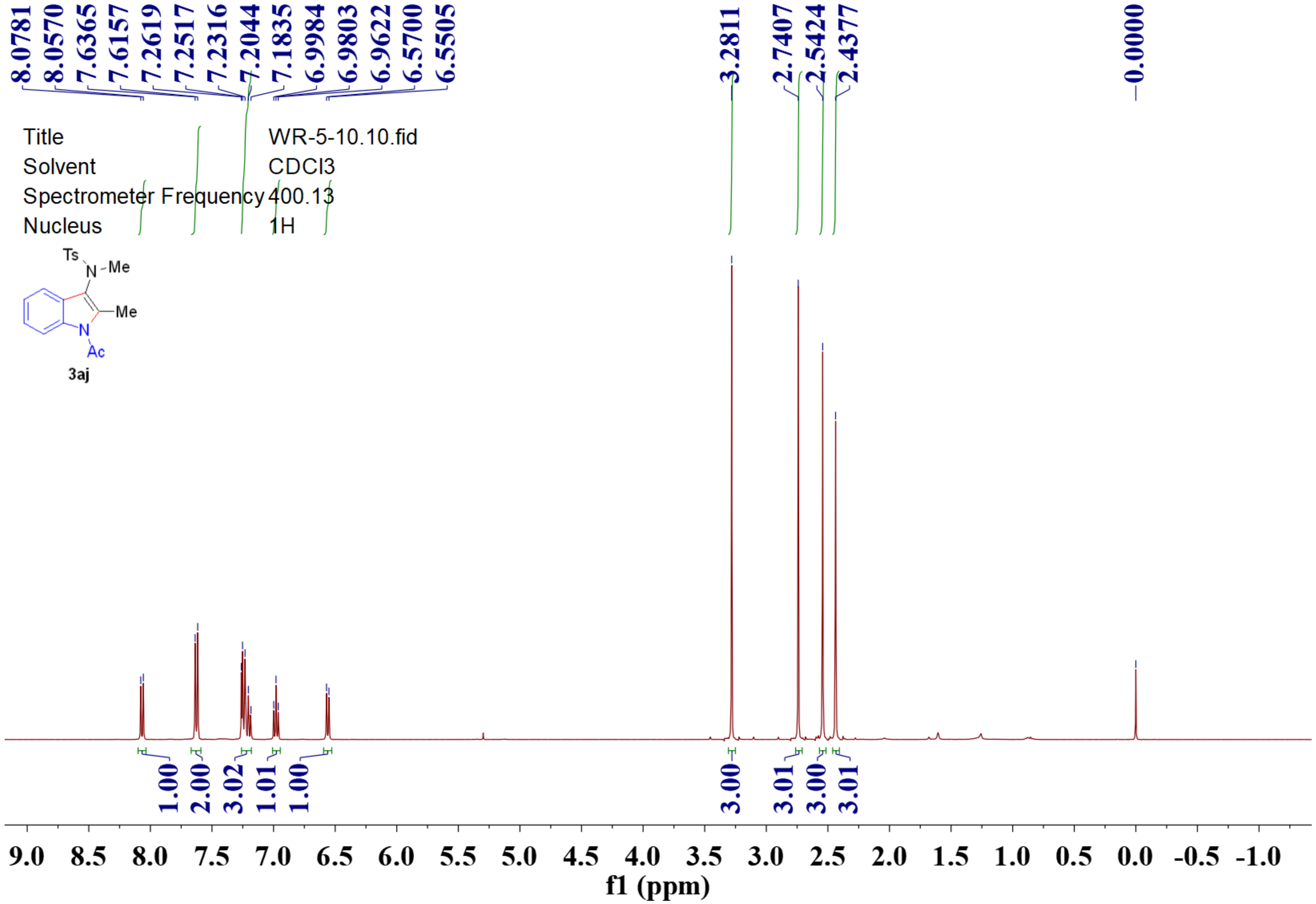

8.0781
8.0570
7.6365
7.6157
7.2619
7.2517
7.2316
7.2044
7.1835
6.9984
6.9803
6.9622
6.5700
6.5505
3.2811
2.7407
2.5424
2.4377
-0.0000
Title WR-5-10.10.fid
Solvent CDCl3
Spectrometer Frequency 400.13
Nucleus 1H
Ts
N
Me
Me
N
Ac
1.00
2.00
3.02
1.01
1.00
3.00
3.01
3.00
3.01
9.0 8.5 8.0 7.5 7.0 6.5 6.0 5.5 5.0 4.5 4.0 3.5 3.0 2.5 2.0 1.5 1.0 0.5 0.0 -0.5 -1.0
f1 (ppm)

3aj

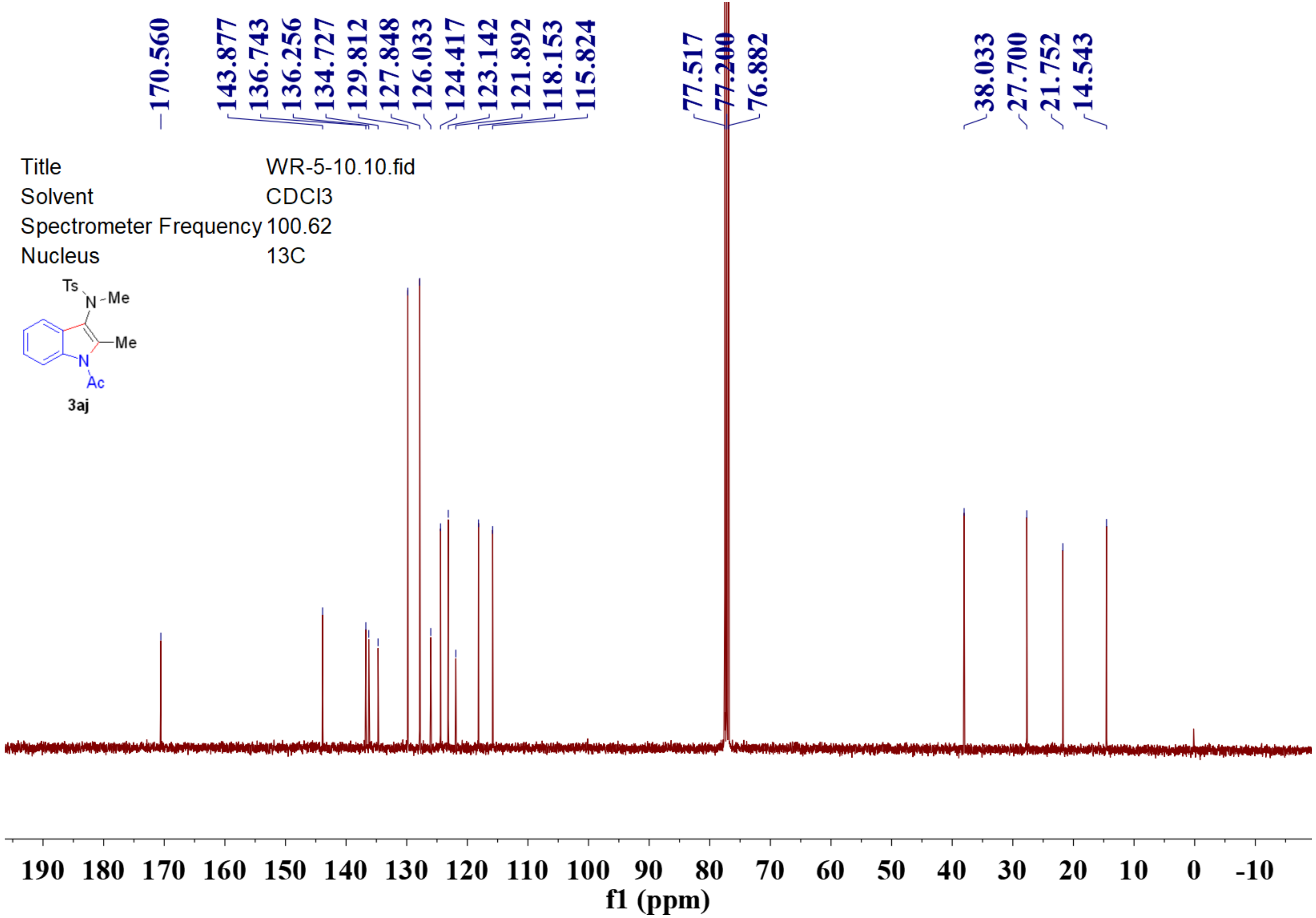

170.560
143.877
136.743
136.256
134.727
129.812
127.848
126.033
124.417
123.142
121.892
118.153
115.824
77.517
77.200
76.882
38.033
27.700
21.752
14.543
Title WR-5-10.10.fid
Solvent CDCl3
Spectrometer Frequency 100.62
Nucleus 13C
Ts
N
Me
Me
N
Ac
3aj
190 180 170 160 150 140 130 120 110 100 90 80 70 60 50 40 30 20 10 0 -10
f1 (ppm)

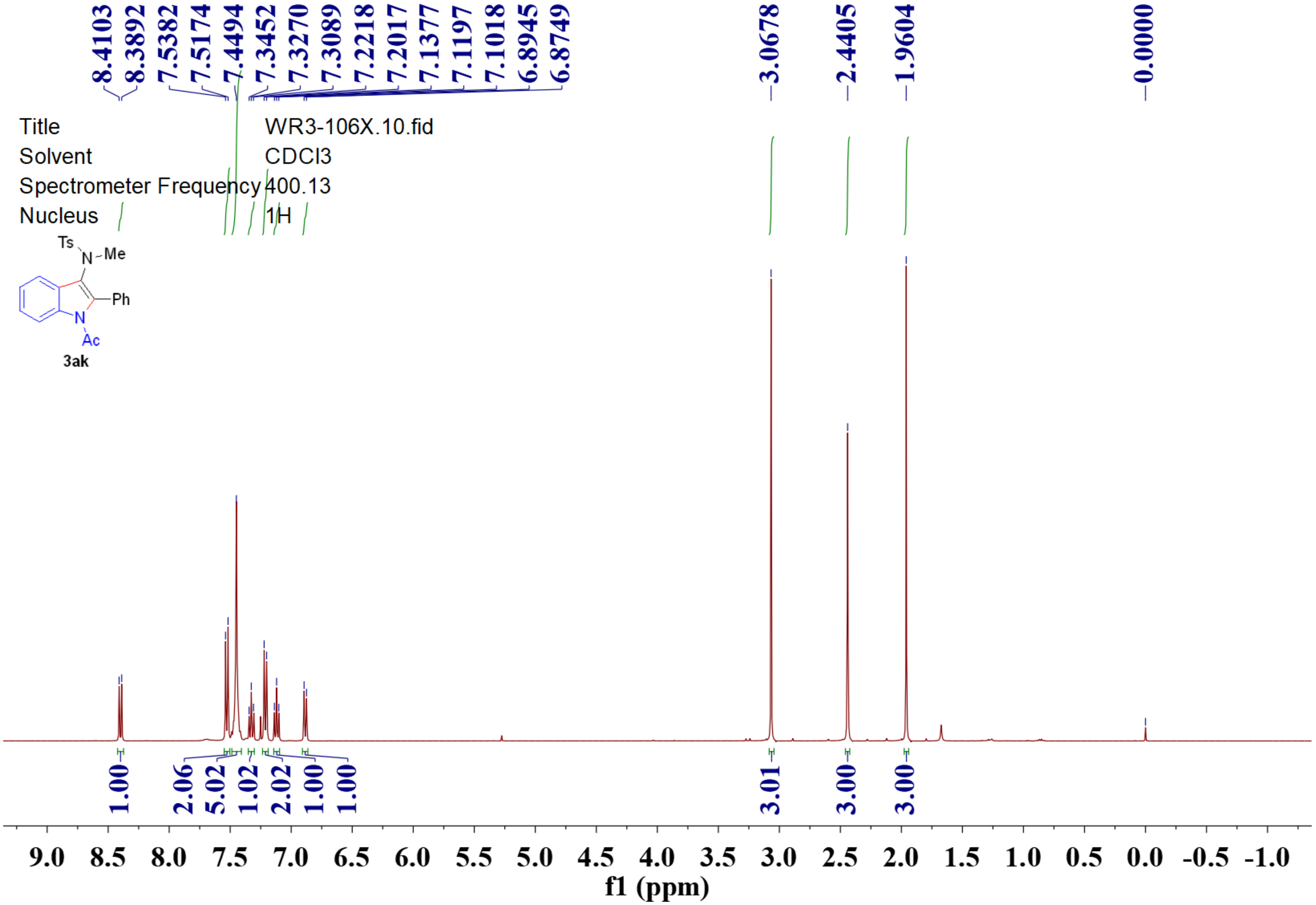
8.4103
8.3892
7.5382
7.5174
7.4494
7.3452
7.3270
7.3089
7.2218
7.2017
7.1377
7.1197
7.1018
6.8945
6.8749
3.0678
2.4405
1.9604
0.0000
Title WR3-106X.10.fid
Solvent CDCl3
Spectrometer Frequency 400.13
Nucleus 1H
Ts
N
Me
Ph
N
Ac
3ak
1.00
2.06
5.02
1.02
2.02
1.00
1.00
3.01
3.00
3.00
9.0 8.5 8.0 7.5 7.0 6.5 6.0 5.5 5.0 4.5 4.0 3.5 3.0 2.5 2.0 1.5 1.0 0.5 0.0 -0.5 -1.0
f1 (ppm)

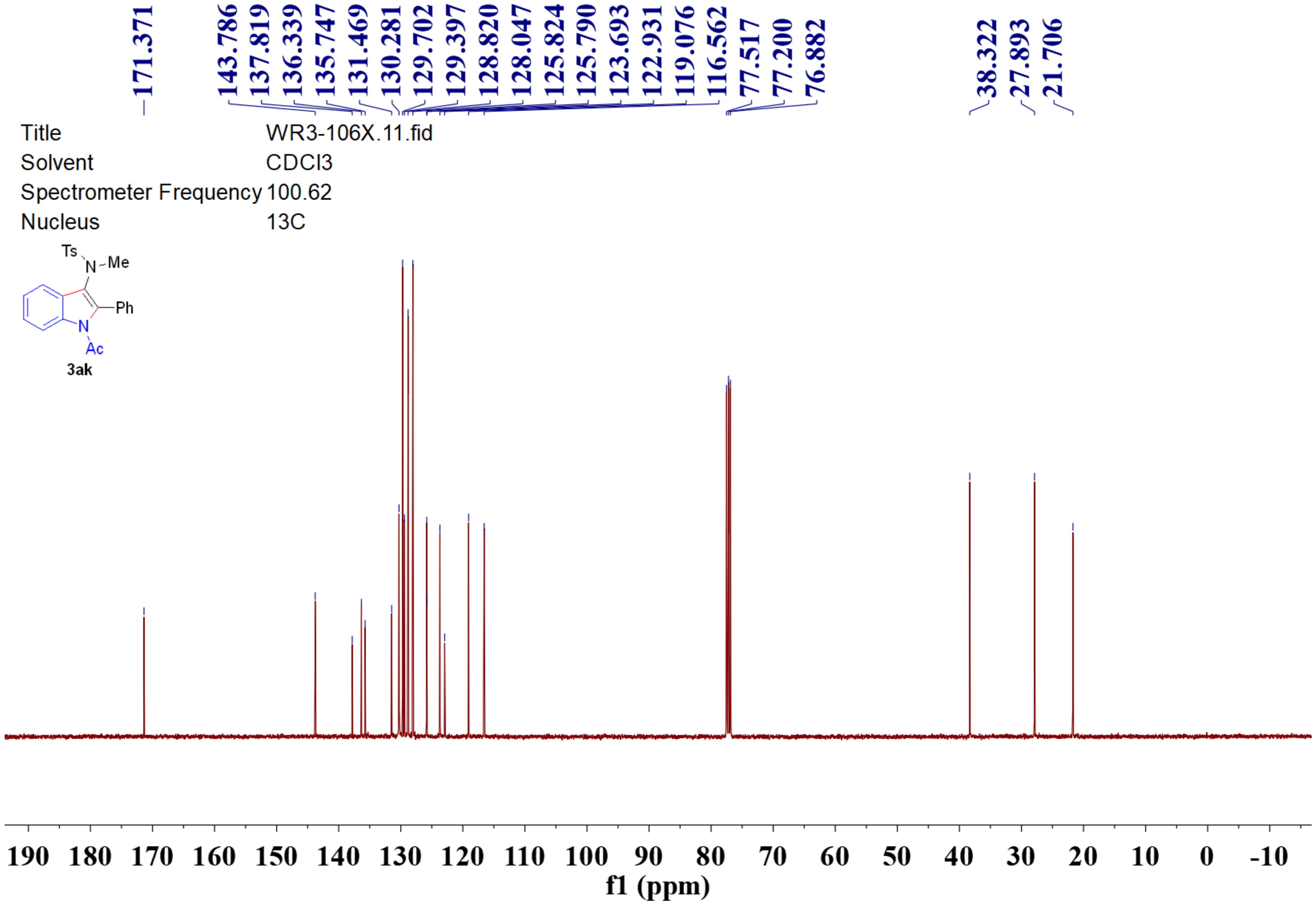
171.371
143.786
137.819
136.339
135.747
131.469
130.281
129.702
129.397
128.820
128.047
125.824
125.790
123.693
122.931
119.076
116.562
77.517
77.200
76.882
38.322
27.893
21.706
Title WR3-106X.11.fid
Solvent CDCl3
Spectrometer Frequency 100.62
Nucleus 13C
Ts
N
Me
Ph
N
Ac
3ak
190 180 170 160 150 140 130 120 110 100 90 80 70 60 50 40 30 20 10 0 -10
f1 (ppm)

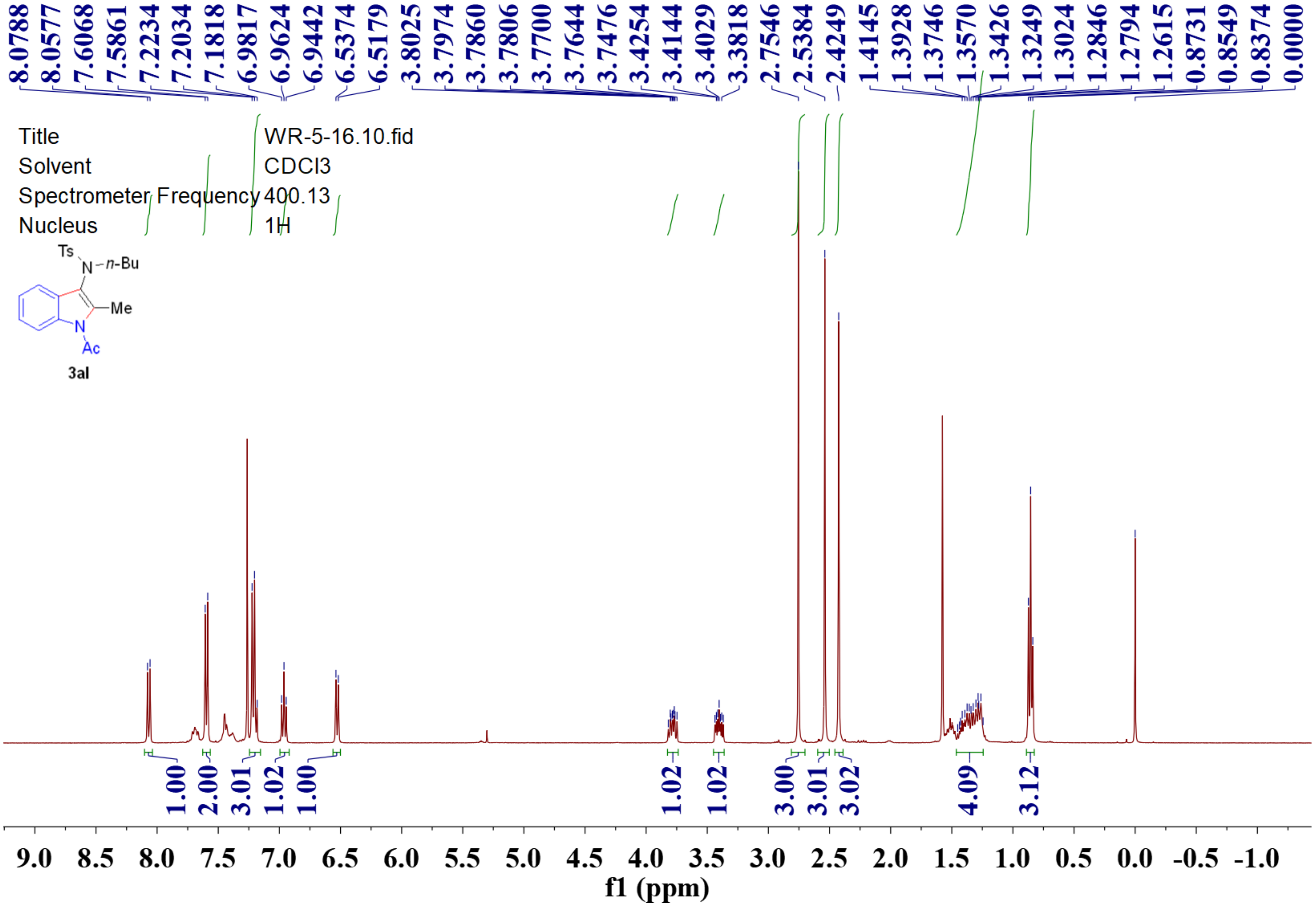
Title WR-5-16.10.fid
Solvent CDCl3
Spectrometer Frequency 400.13
Nucleus 1H
Ts
N
n-Bu
Me
N
Ac
3al
8.0788
8.0577
7.6068
7.5861
7.2234
7.2034
7.1818
6.9817
6.9624
6.9442
6.5374
6.5179
3.8025
3.7974
3.7860
3.7806
3.7700
3.7644
3.7476
3.4254
3.4144
3.4029
3.3818
2.7546
2.5384
2.4249
1.4145
1.3928
1.3746
1.3570
1.3426
1.3249
1.3024
1.2846
1.2794
1.2615
0.8731
0.8549
0.8374
0.0000
1.00
2.00
3.01
1.02
1.00
1.02
1.02
3.00
3.01
3.02
4.09
3.12
9.0 8.5 8.0 7.5 7.0 6.5 6.0 5.5 5.0 4.5 4.0 3.5 3.0 2.5 2.0 1.5 1.0 0.5 0.0 -0.5 -1.0
f1 (ppm)

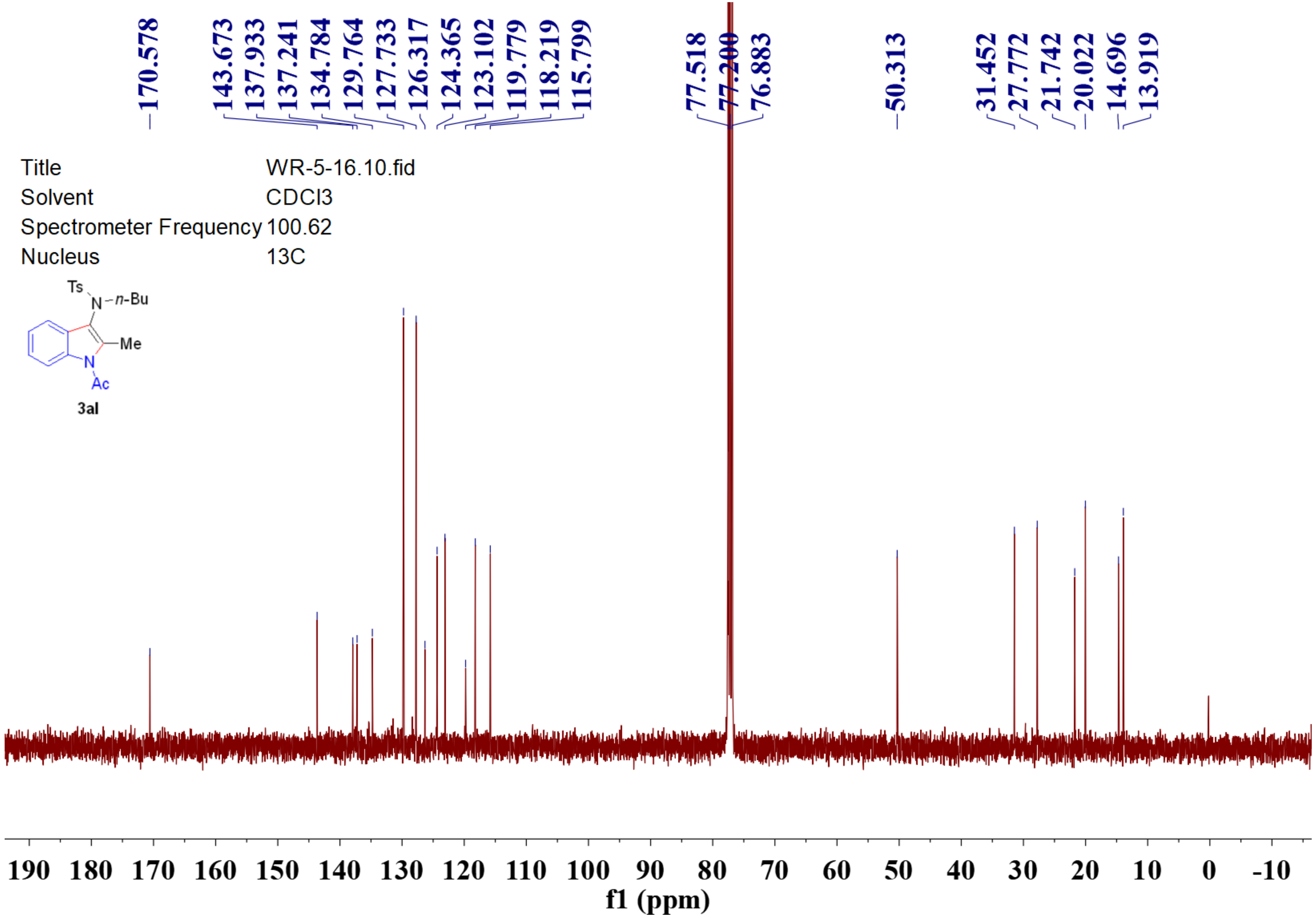

170.578
143.673
137.933
137.241
134.784
129.764
127.733
126.317
124.365
123.102
119.779
118.219
115.799
77.518
77.200
76.883
50.313
31.452
27.772
21.742
20.022
14.696
13.919
Title WR-5-16.10.fid
Solvent CDCl3
Spectrometer Frequency 100.62
Nucleus 13C
Ts
N
n-Bu
Me
N
Ac
3al
190 180 170 160 150 140 130 120 110 100 90 80 70 60 50 40 30 20 10 0 -10
f1 (ppm)

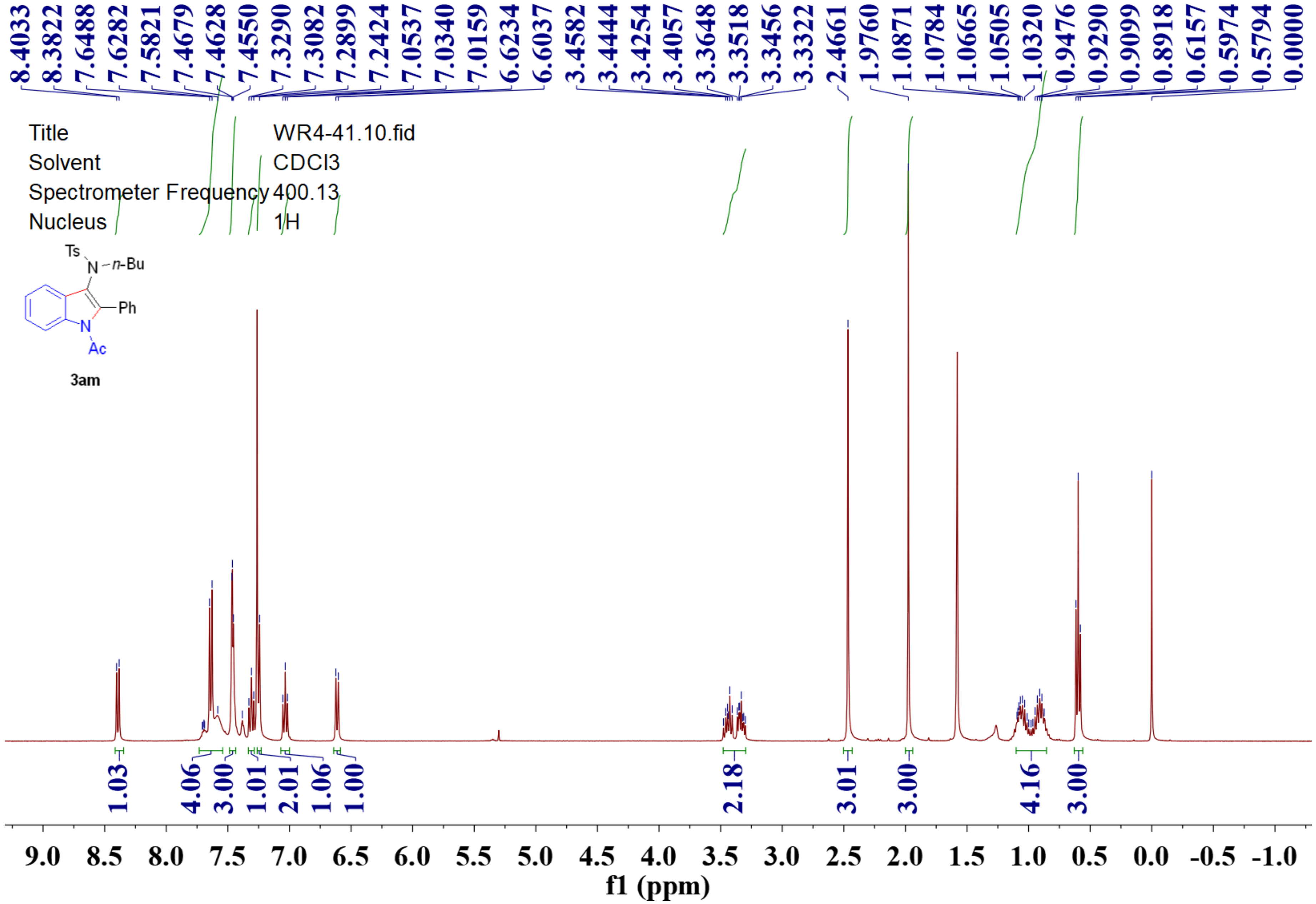

Title WR4-41.10.fid
Solvent CDCl3
Spectrometer Frequency 400.13
Nucleus 1H
3am
f1 (ppm)

Title WR-4-41.10.fid

Solvent CDCl3

Spectrometer Frequency 100.62

Nucleus 13C

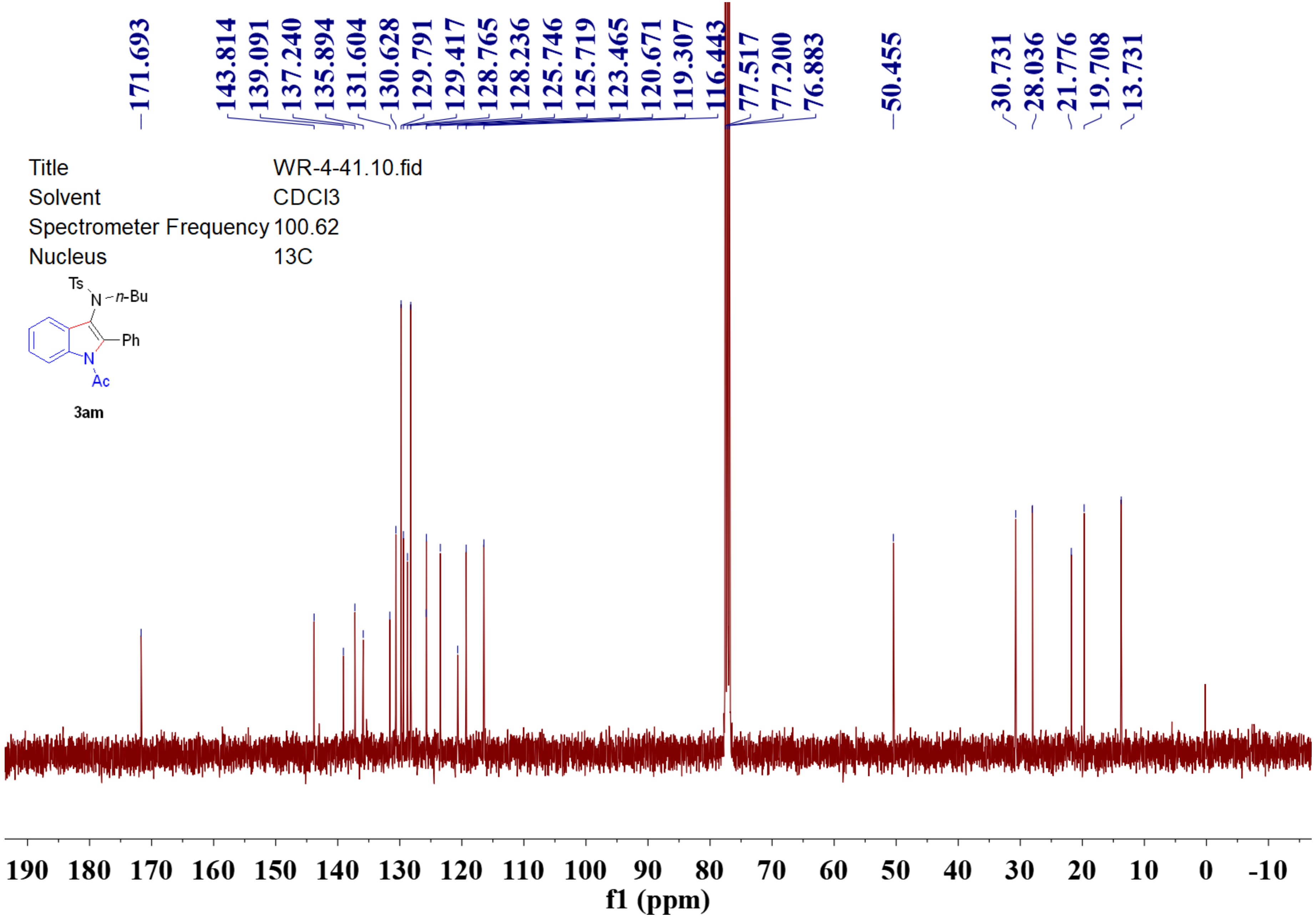

3am

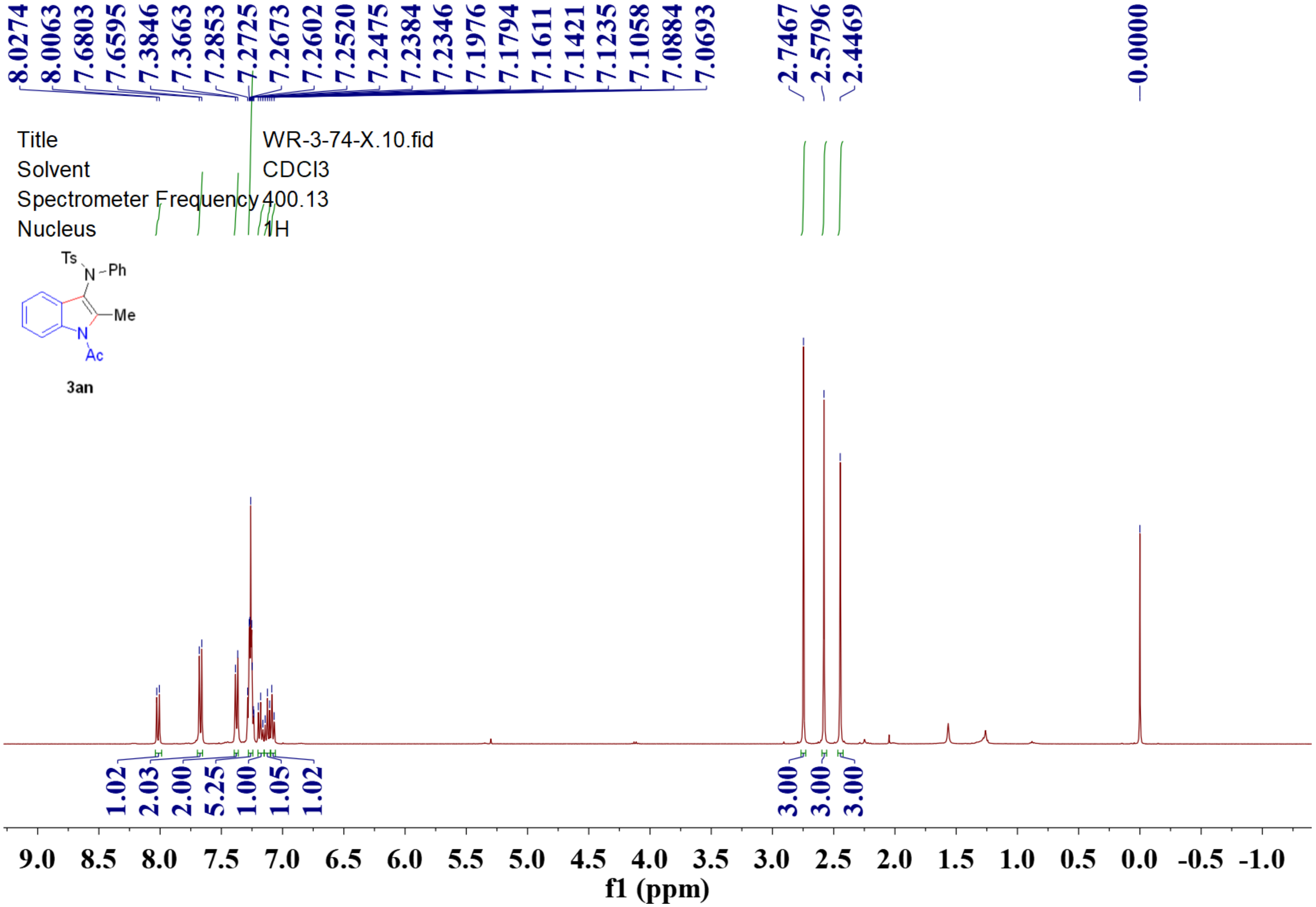
8.0274
8.0063
7.6803
7.6595
7.3846
7.3663
7.2853
7.2725
7.2673
7.2602
7.2520
7.2475
7.2384
7.2346
7.1976
7.1794
7.1611
7.1421
7.1235
7.1058
7.0884
7.0693
2.7467
2.5796
2.4469
-0.0000
Title WR-3-74-X.10.fid
Solvent CDCl3
Spectrometer Frequency 400.13
Nucleus 1H
Ts
N
Ph
Me
N
Ac
3an
1.02
2.03
2.00
5.25
1.00
1.05
1.02
3.00
3.00
3.00
9.0 8.5 8.0 7.5 7.0 6.5 6.0 5.5 5.0 4.5 4.0 3.5 3.0 2.5 2.0 1.5 1.0 0.5 0.0 -0.5 -1.0
f1 (ppm)

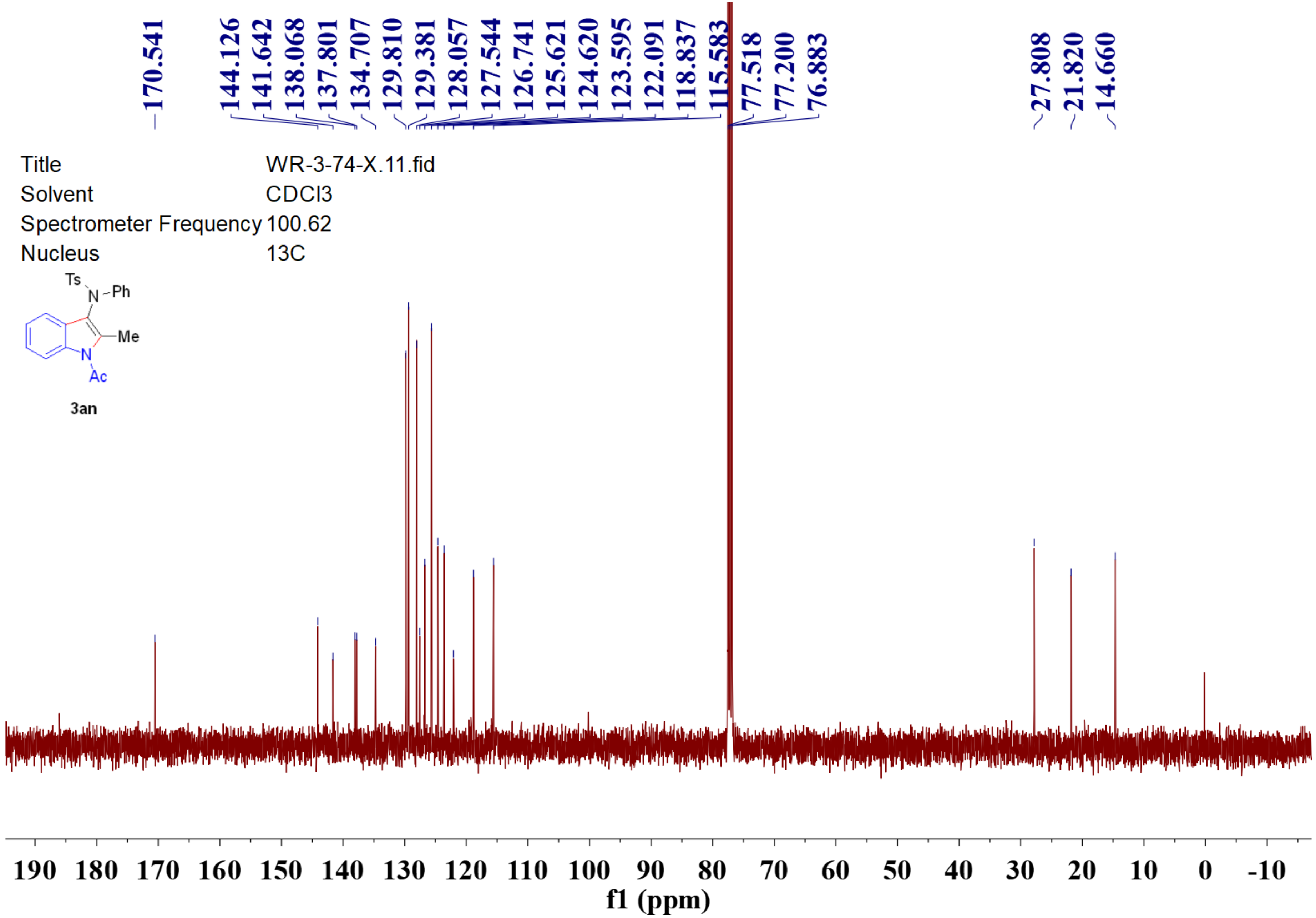

170.541
144.126
141.642
138.068
137.801
134.707
129.810
129.381
128.057
127.544
126.741
125.621
124.620
123.595
122.091
118.837
115.583
77.518
77.200
76.883
27.808
21.820
14.660
Title WR-3-74-X.11.fid
Solvent CDCl3
Spectrometer Frequency 100.62
Nucleus 13C
Ts
N
Ph
Me
N
Ac
3an
190 180 170 160 150 140 130 120 110 100 90 80 70 60 50 40 30 20 10 0 -10
f1 (ppm)

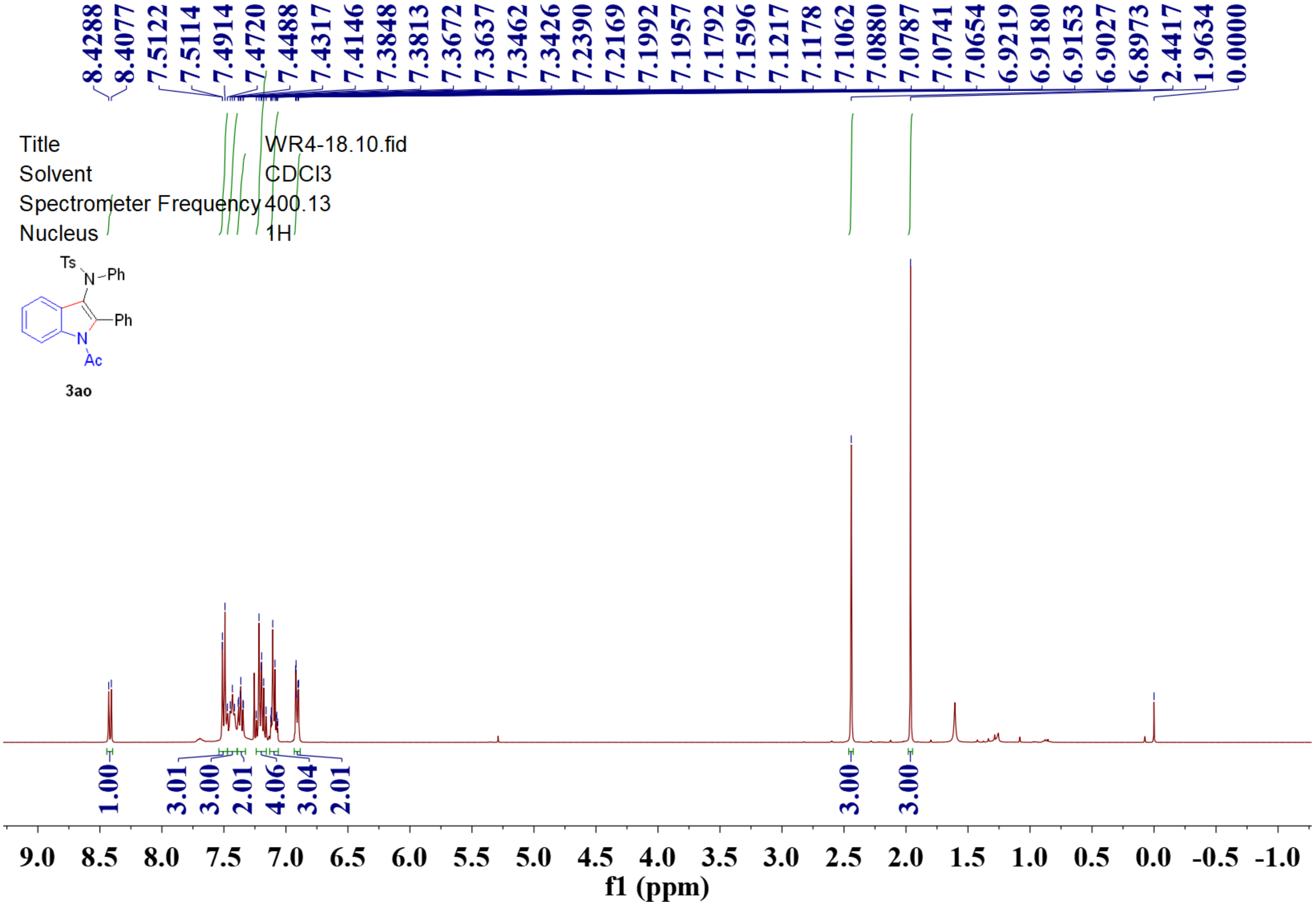
8.4288
8.4077
7.5122
7.5114
7.4914
7.4720
7.4488
7.4317
7.4146
7.3848
7.3813
7.3672
7.3637
7.3462
7.3426
7.2390
7.2169
7.1992
7.1957
7.1792
7.1596
7.1217
7.1178
7.1062
7.0880
7.0787
7.0741
7.0654
6.9219
6.9180
6.9153
6.9027
6.8973
2.4417
1.9634
0.0000
Title WR4-18.10.fid
Solvent CDCl3
Spectrometer Frequency 400.13
Nucleus 1H
Ts
N
Ph
Ph
N
Ac
3ao
1.00
3.01
3.00
2.01
4.06
3.04
2.01
3.00
3.00
9.0 8.5 8.0 7.5 7.0 6.5 6.0 5.5 5.0 4.5 4.0 3.5 3.0 2.5 2.0 1.5 1.0 0.5 0.0 -0.5 -1.0
f1 (ppm)

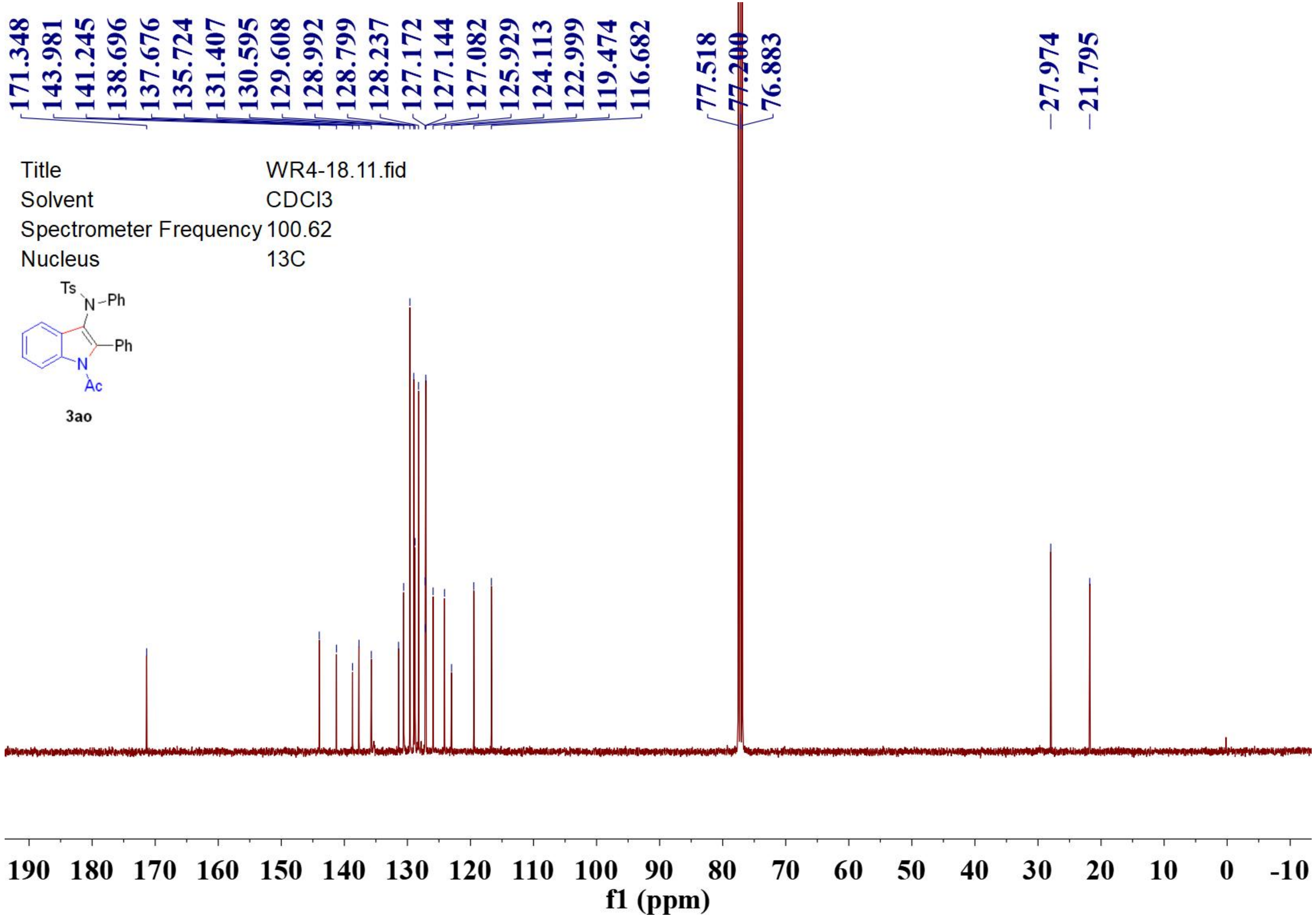
171.348
143.981
141.245
138.696
137.676
135.724
131.407
130.595
129.608
128.992
128.799
128.237
127.172
127.144
127.082
125.929
124.113
122.999
119.474
116.682
77.518
77.200
76.883
27.974
21.795
Title WR4-18.11.fid
Solvent CDCl3
Spectrometer Frequency 100.62
Nucleus 13C
Ts
N
Ph
Ph
N
Ac
3ao
190 180 170 160 150 140 130 120 110 100 90 80 70 60 50 40 30 20 10 0 -10
f1 (ppm)

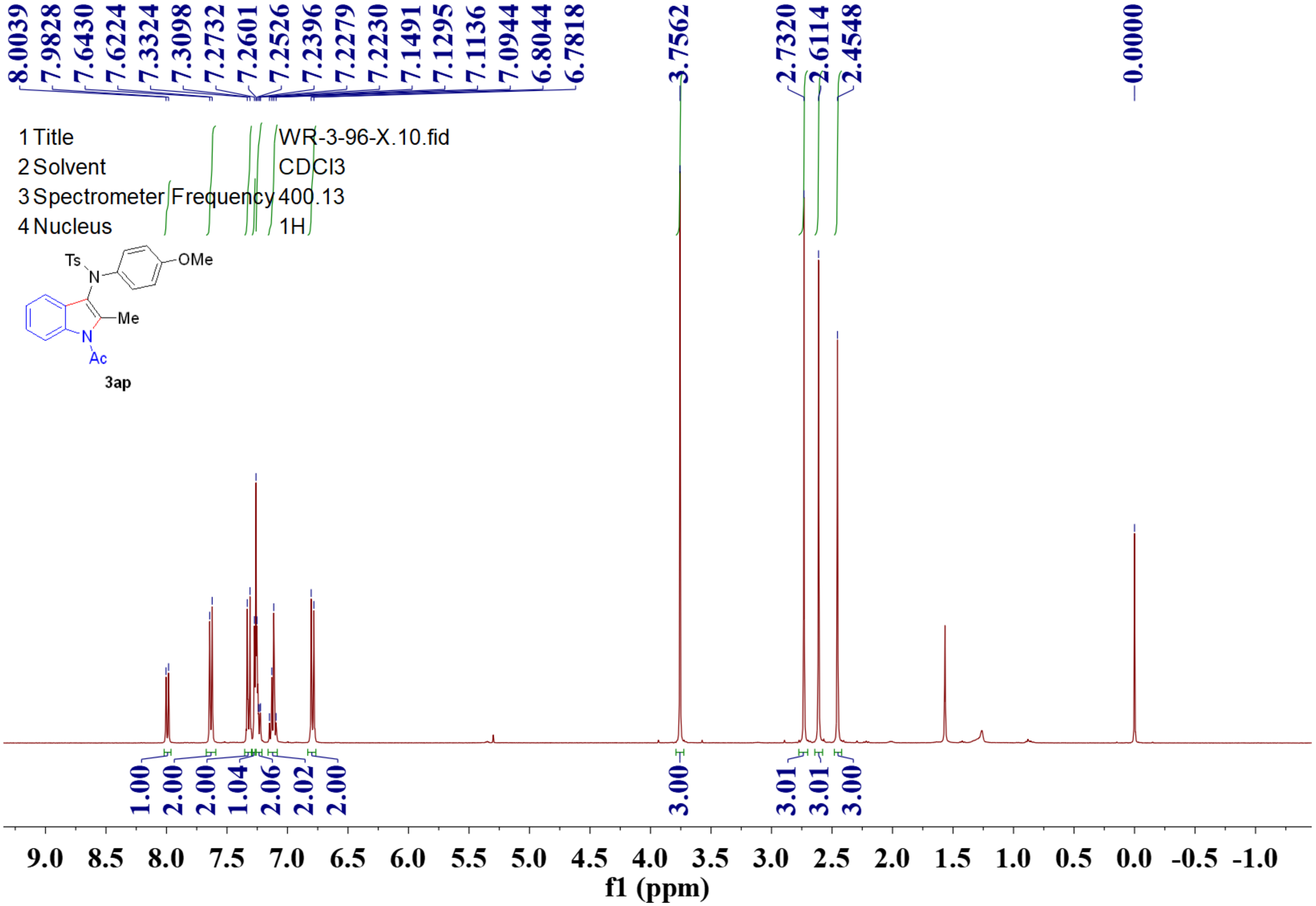

8.0039
7.9828
7.6430
7.6224
7.3324
7.3098
7.2732
7.2601
7.2526
7.2396
7.2279
7.2230
7.1491
7.1295
7.1136
7.0944
6.8044
6.7818
3.7562
2.7320
2.6114
2.4548
-0.0000
1 Title WR-3-96-X.10.fid
2 Solvent CDCl3
3 Spectrometer Frequency 400.13
4 Nucleus 1H
Ts
N
OMe
Me
N
Ac
3ap
1.00
2.00
2.00
1.04
2.06
2.02
2.00
3.00
3.01
3.01
3.00
9.0 8.5 8.0 7.5 7.0 6.5 6.0 5.5 5.0 4.5 4.0 3.5 3.0 2.5 2.0 1.5 1.0 0.5 0.0 -0.5 -1.0
f1 (ppm)

Title WR3-96-2.10.fid
Solvent CDCl3
Spectrometer Frequency 100.62
Nucleus 13C

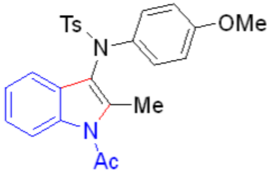

3ap

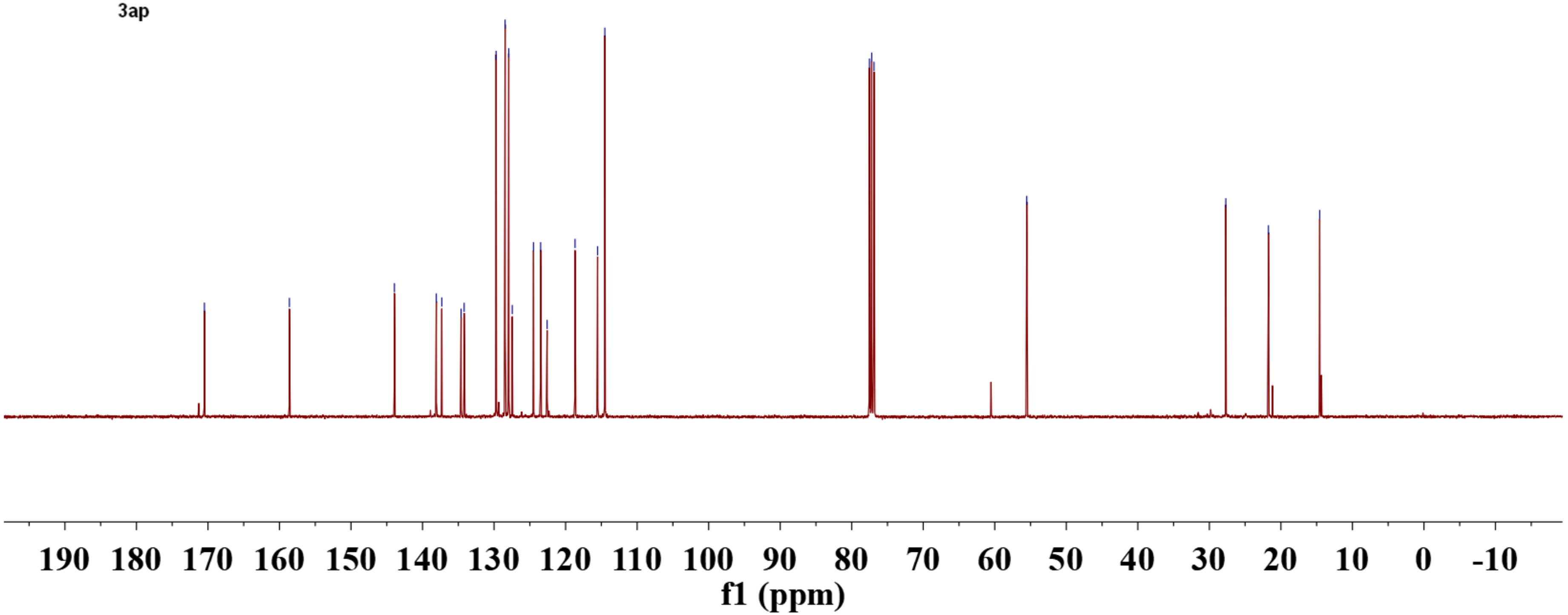

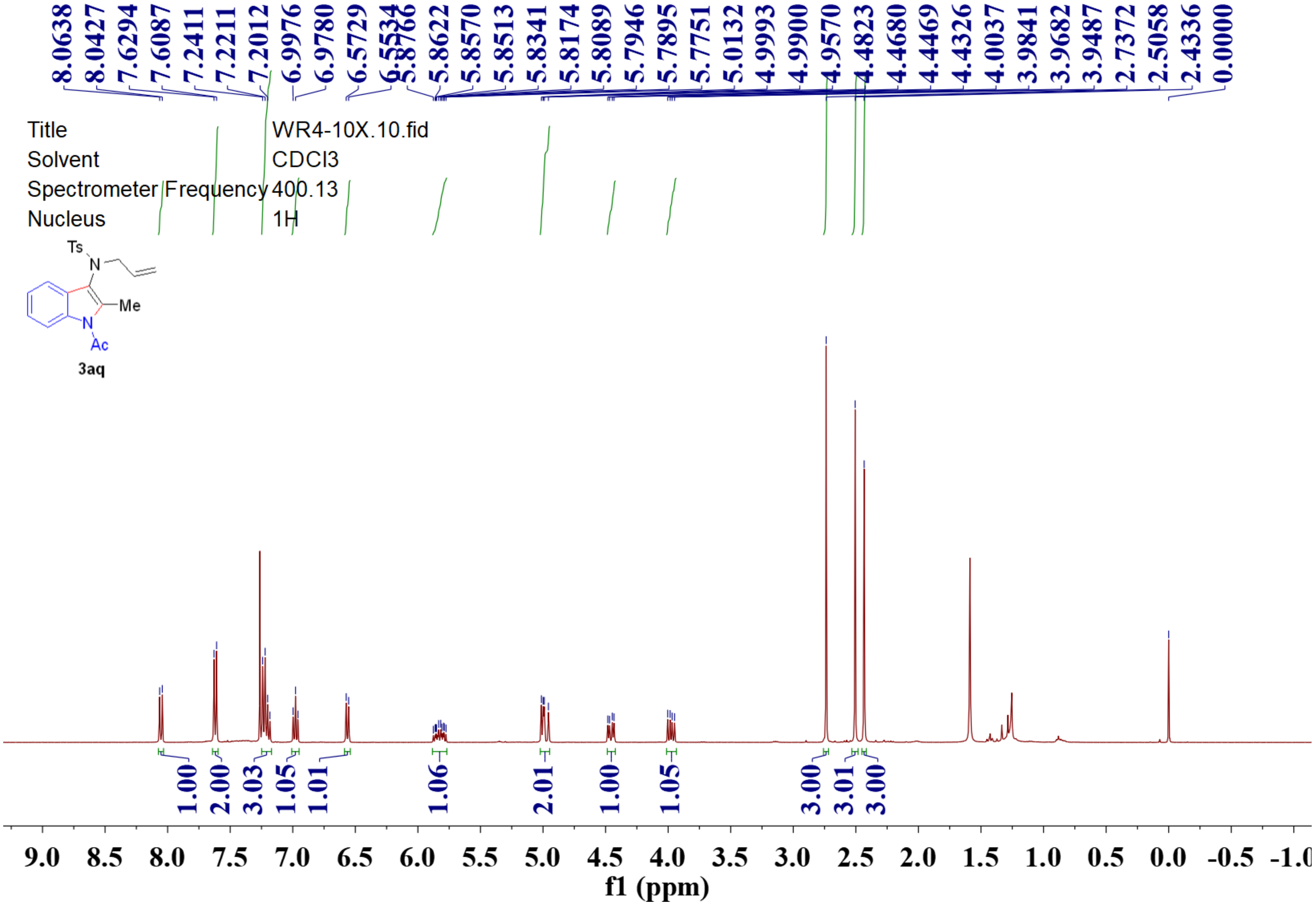

8.0638
8.0427
7.6294
7.6087
7.2411
7.2211
7.2012
6.9976
6.9780
6.5729
6.5534
5.8766
5.8622
5.8570
5.8513
5.8341
5.8174
5.8089
5.7946
5.7895
5.7751
5.0132
4.9993
4.9900
4.9570
4.4823
4.4680
4.4469
4.4326
4.0037
3.9841
3.9682
3.9487
2.7372
2.5058
2.4336
0.0000
Title WR4-10X.10.fid
Solvent CDCl3
Spectrometer Frequency 400.13
Nucleus 1H
Ts
N
Me
N
Ac
3aq
1.00
2.00
3.03
1.05
1.01
1.06
2.01
1.00
1.05
3.00
3.01
3.00
9.0 8.5 8.0 7.5 7.0 6.5 6.0 5.5 5.0 4.5 4.0 3.5 3.0 2.5 2.0 1.5 1.0 0.5 0.0 -0.5 -1.0
f1 (ppm)

Title WR4-10X.11.fid
Solvent CDCl3
Spectrometer Frequency 100.62
Nucleus 13C

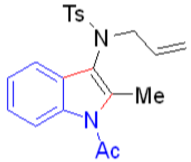

3aq

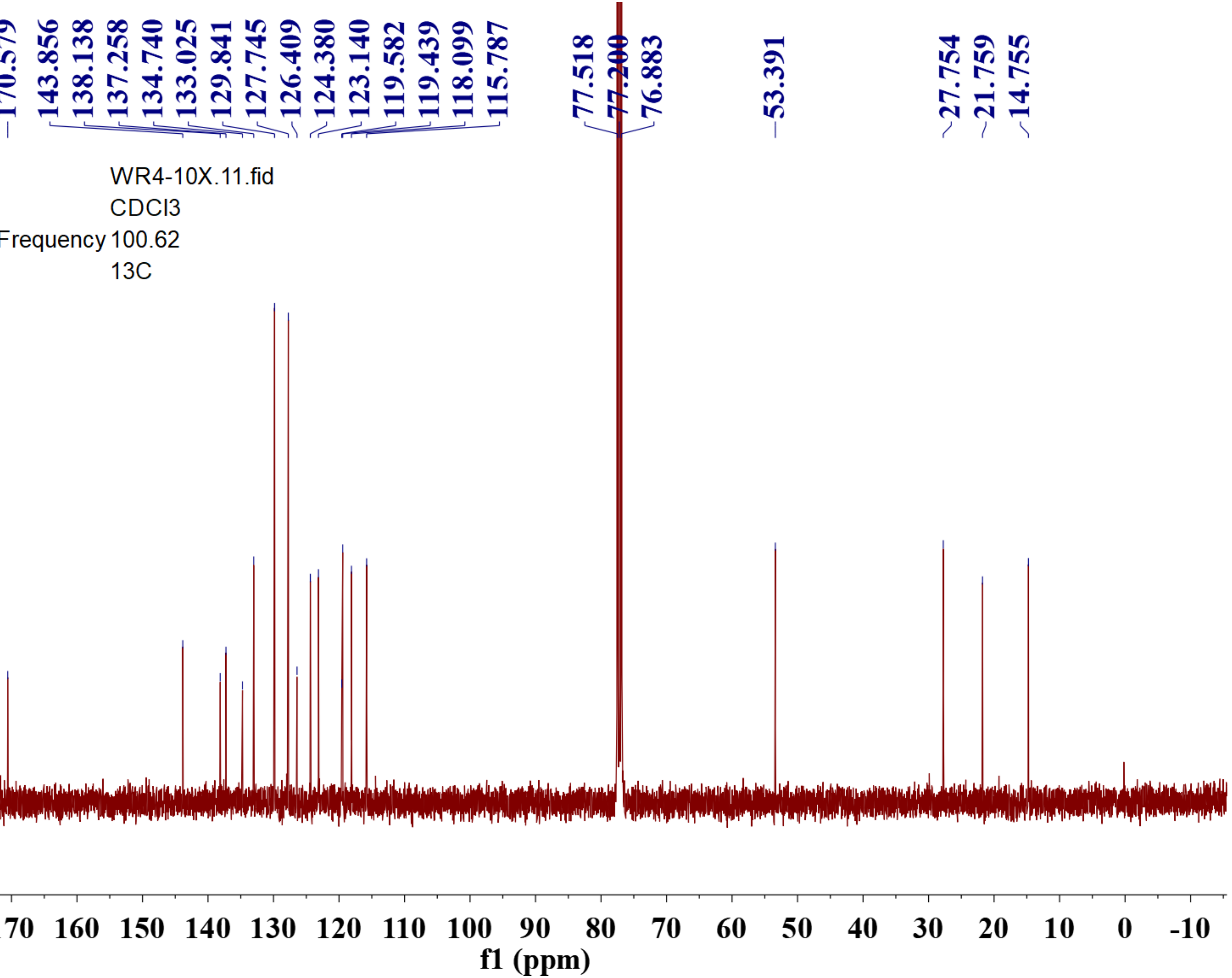

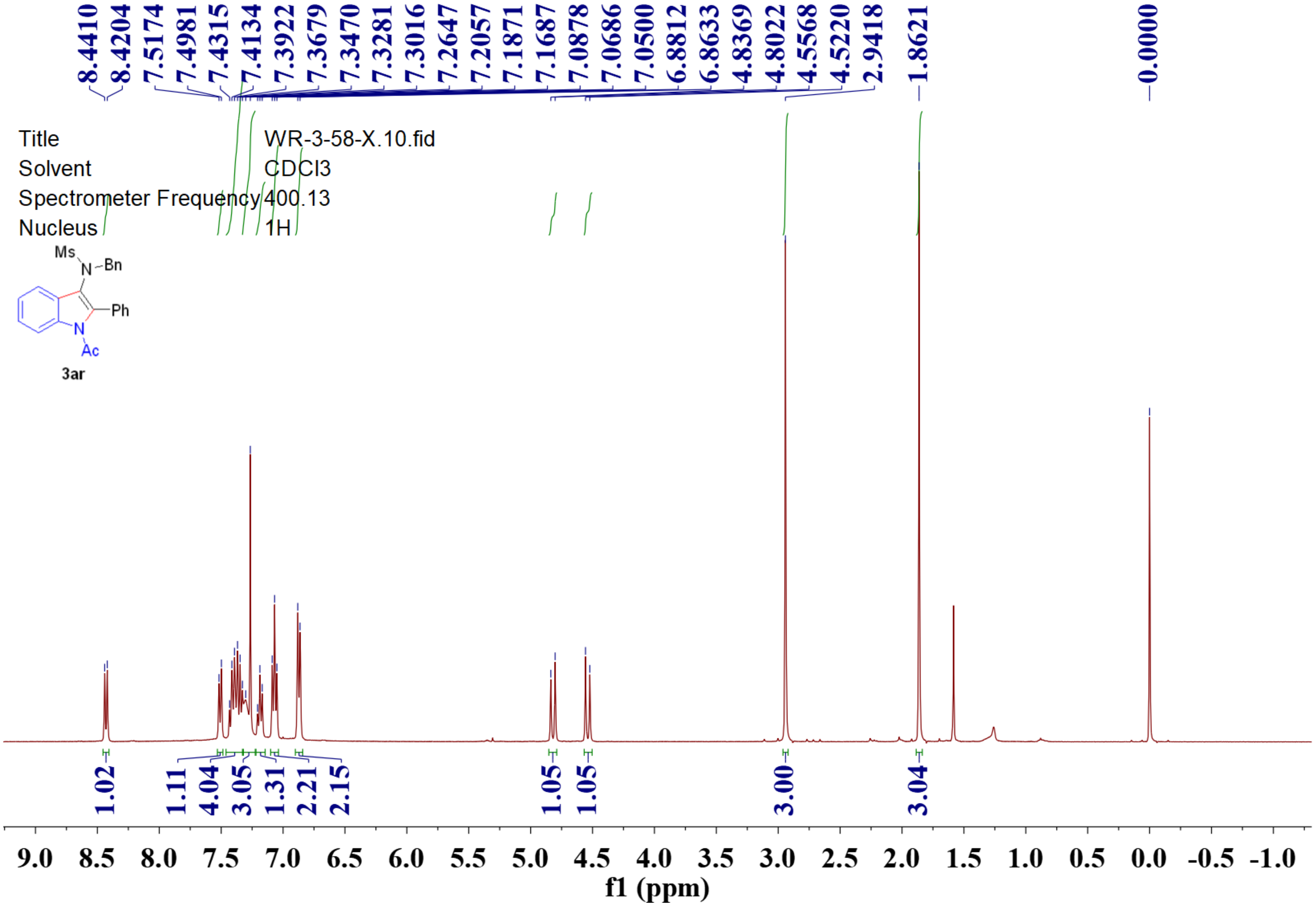
8.4410
8.4204
7.5174
7.4981
7.4315
7.4134
7.3922
7.3679
7.3470
7.3281
7.3016
7.2647
7.2057
7.1871
7.1687
7.0878
7.0686
7.0500
6.8812
6.8633
4.8369
4.8022
4.5568
4.5220
2.9418
1.8621
0.0000
Title WR-3-58-X.10.fid
Solvent CDCl3
Spectrometer Frequency 400.13
Nucleus 1H
Ms
N
Bn
Ph
N
Ac
3ar
1.02
1.11
4.04
3.05
1.31
2.21
2.15
1.05
1.05
3.00
3.04
9.0 8.5 8.0 7.5 7.0 6.5 6.0 5.5 5.0 4.5 4.0 3.5 3.0 2.5 2.0 1.5 1.0 0.5 0.0 -0.5 -1.0
f1 (ppm)

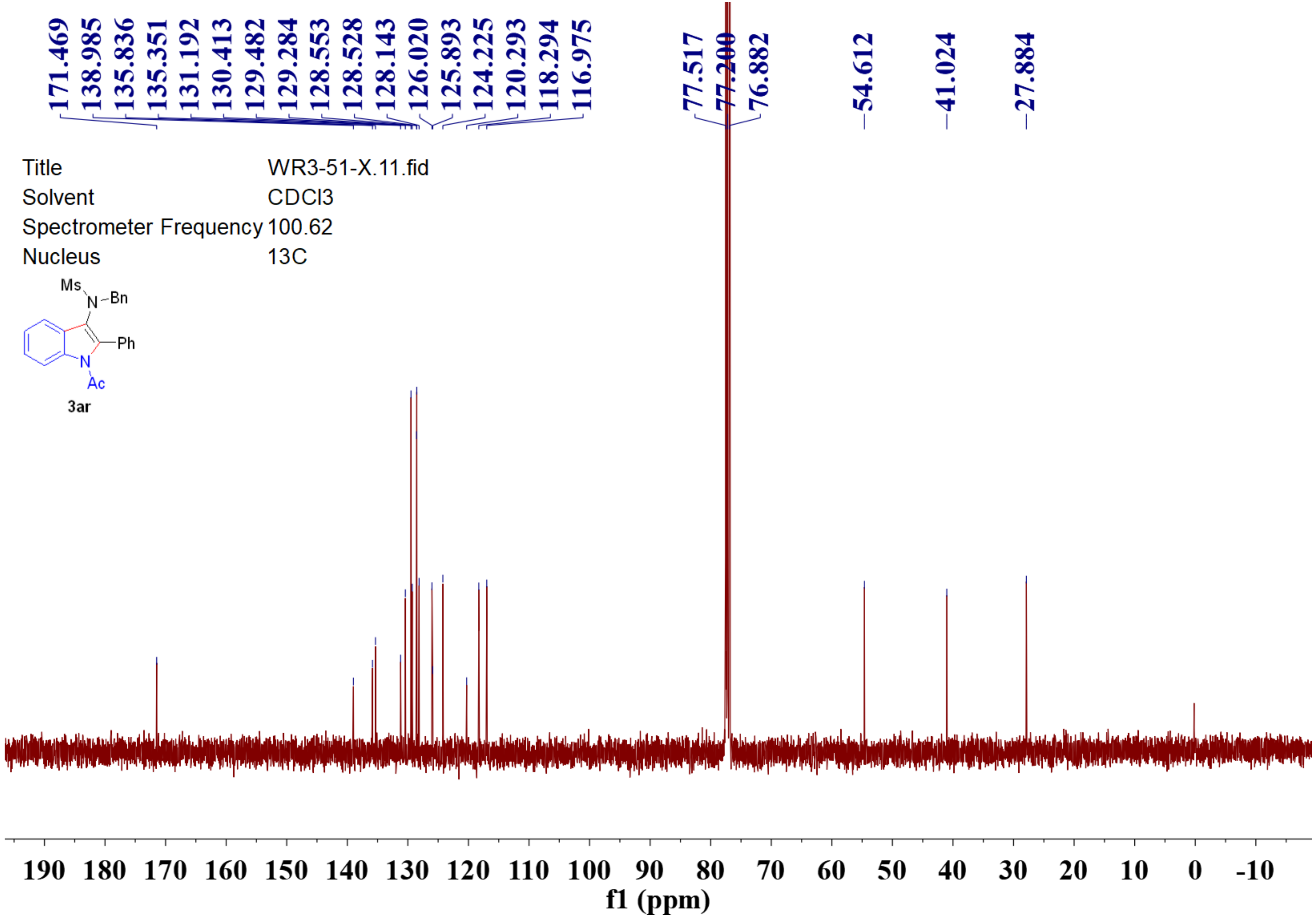
171.469
138.985
135.836
135.351
131.192
130.413
129.482
129.284
128.553
128.528
128.143
126.020
125.893
124.225
120.293
118.294
116.975
77.517
77.200
76.882
54.612
41.024
27.884
Title WR3-51-X.11.fid
Solvent CDCl3
Spectrometer Frequency 100.62
Nucleus 13C
Ms
N
Bn
Ph
N
Ac
3ar
190 180 170 160 150 140 130 120 110 100 90 80 70 60 50 40 30 20 10 0 -10
f1 (ppm)

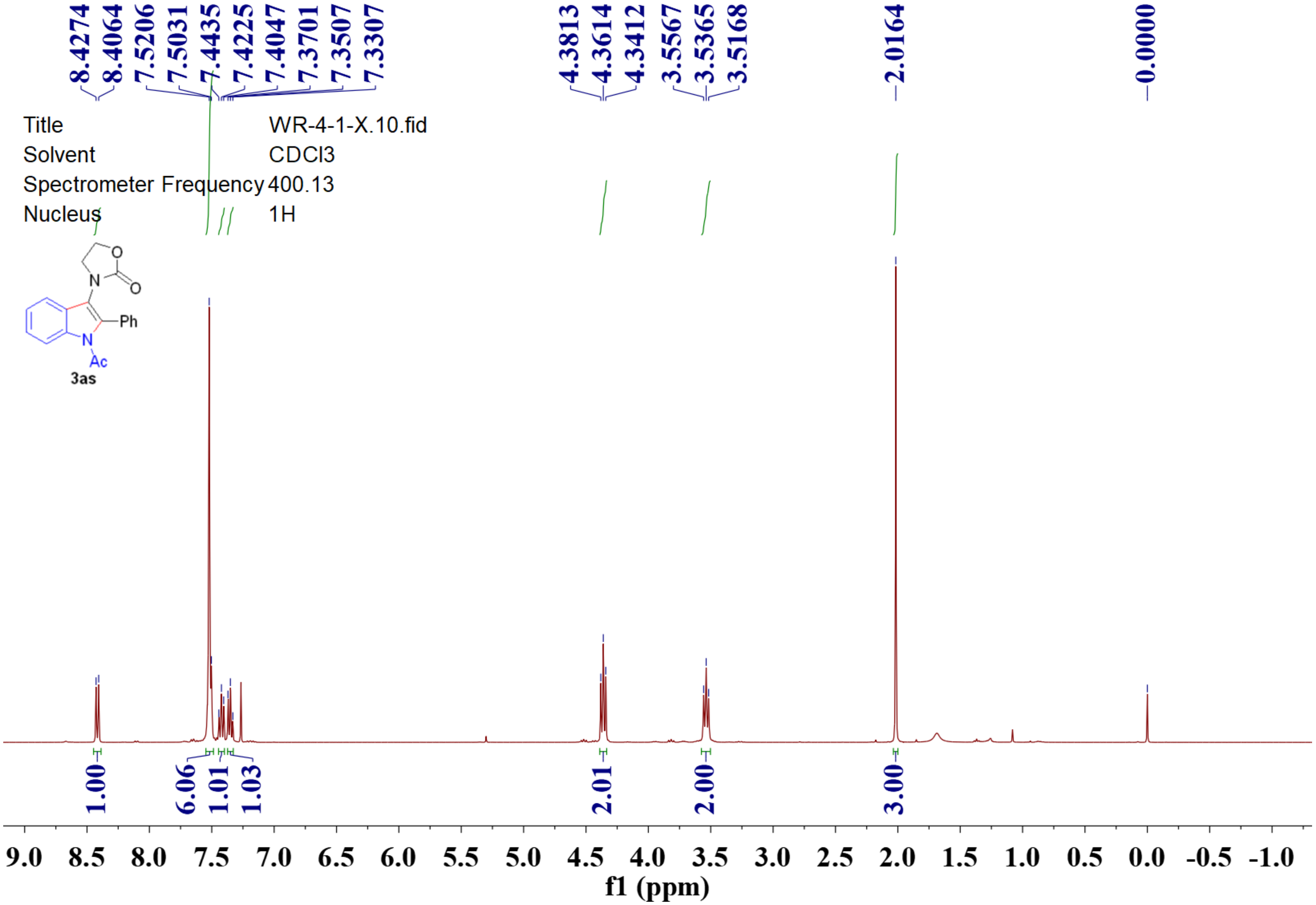
8.4274
8.4064
7.5206
7.5031
7.4435
7.4225
7.4047
7.3701
7.3507
7.3307
4.3813
4.3614
4.3412
3.5567
3.5365
3.5168
2.0164
0.0000
Title WR-4-1-X.10.fid
Solvent CDCl3
Spectrometer Frequency 400.13
Nucleus 1H
O
N
O
Ph
N
Ac
3as
1.00
6.06
1.01
1.03
2.01
2.00
3.00
9.0 8.5 8.0 7.5 7.0 6.5 6.0 5.5 5.0 4.5 4.0 3.5 3.0 2.5 2.0 1.5 1.0 0.5 0.0 -0.5 -1.0
f1 (ppm)

Title WR-4-1-X.10.fid
Solvent CDCl3
Spectrometer Frequency 100.62
Nucleus 13C

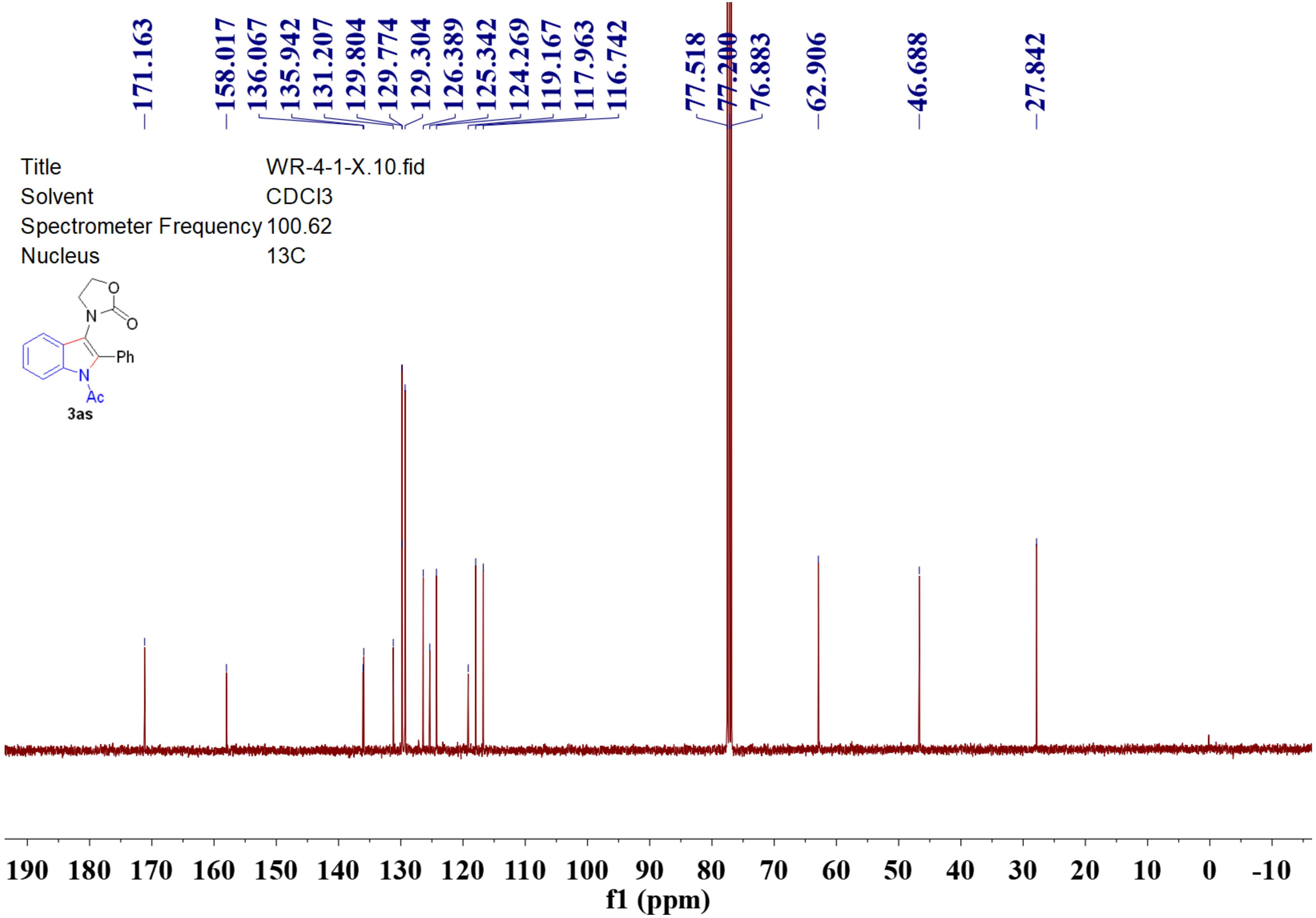

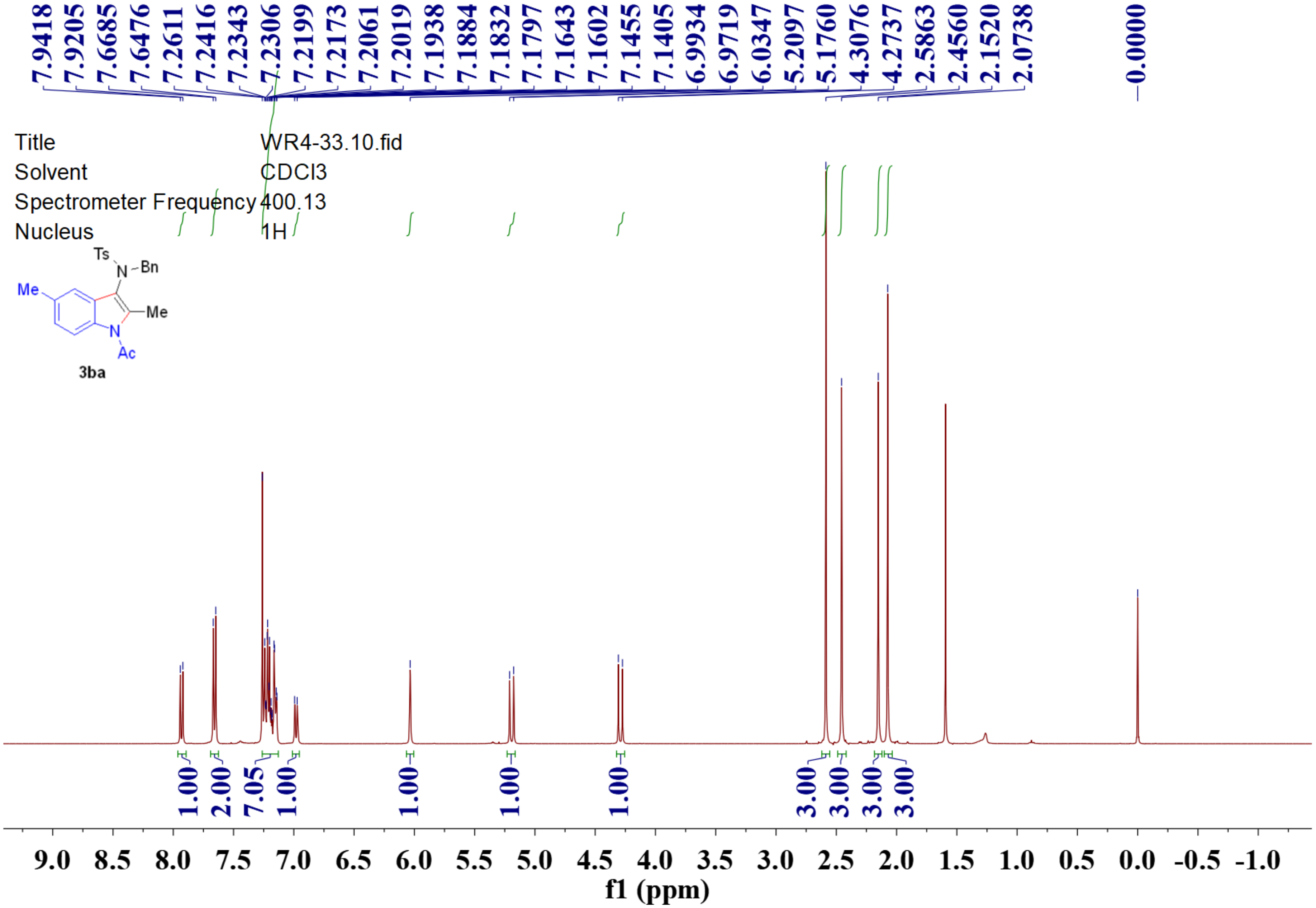

7.9418
7.9205
7.6685
7.6476
7.2611
7.2416
7.2343
7.2306
7.2199
7.2173
7.2061
7.2019
7.1938
7.1884
7.1832
7.1797
7.1643
7.1602
7.1455
7.1405
6.9934
6.9719
6.0347
5.2097
5.1760
4.3076
4.2737
2.5863
2.4560
2.1520
2.0738
0.0000
Title WR4-33.10.fid
Solvent CDCl3
Spectrometer Frequency 400.13
Nucleus 1H
Ts
N
Bn
Me
Me
N
Ac
3ba
1.00
2.00
7.05
1.00
1.00
1.00
1.00
3.00
3.00
3.00
3.00
9.0 8.5 8.0 7.5 7.0 6.5 6.0 5.5 5.0 4.5 4.0 3.5 3.0 2.5 2.0 1.5 1.0 0.5 0.0 -0.5 -1.0
f1 (ppm)

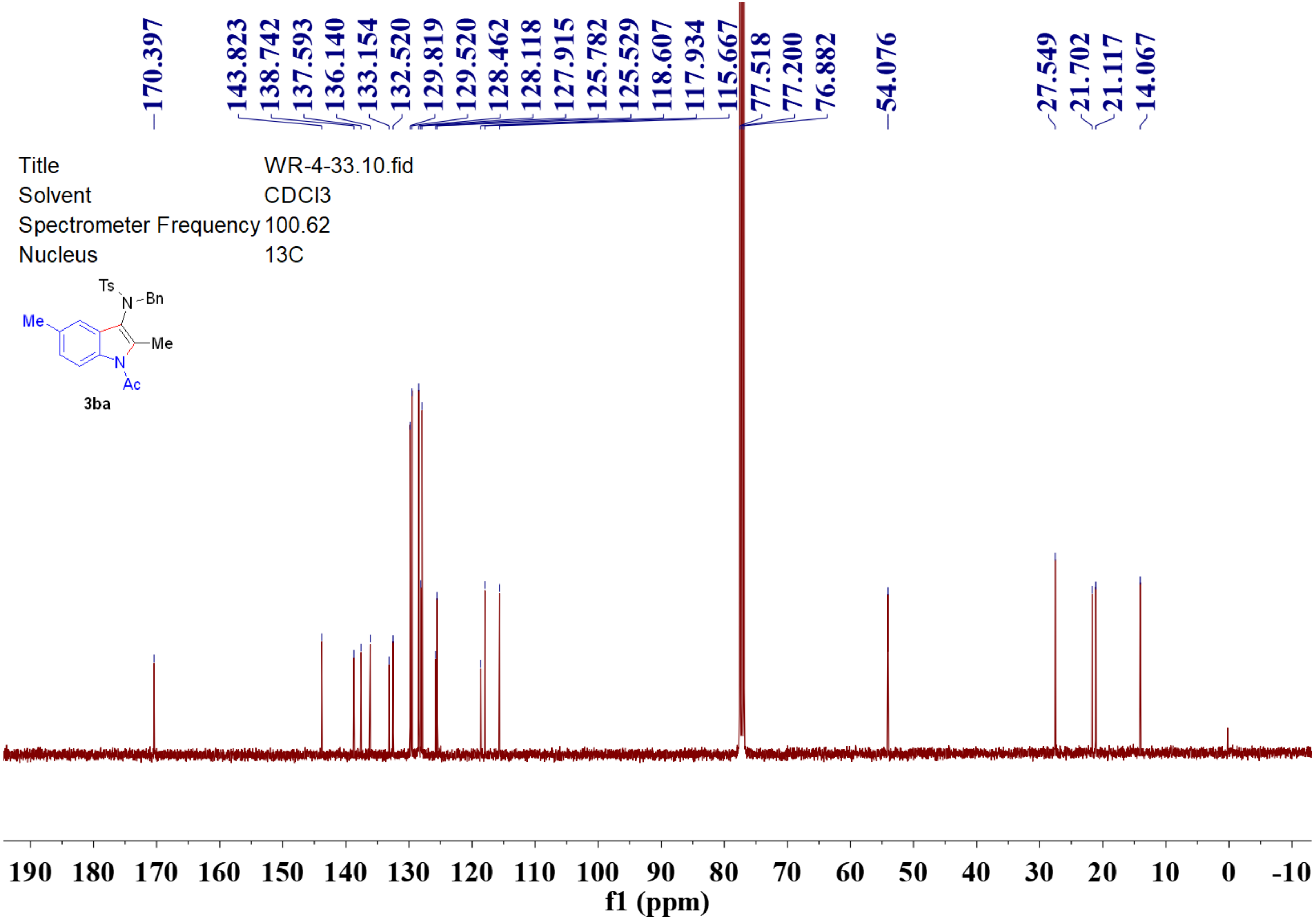
170.397
143.823
138.742
137.593
136.140
133.154
132.520
129.819
129.520
128.462
128.118
127.915
125.782
125.529
118.607
117.934
115.667
77.518
77.200
76.882
54.076
27.549
21.702
21.117
14.067
Title WR-4-33.10.fid
Solvent CDCl3
Spectrometer Frequency 100.62
Nucleus 13C
Ts
N
Bn
Me
Me
N
Ac
3ba
190 180 170 160 150 140 130 120 110 100 90 80 70 60 50 40 30 20 10 0 -10
f1 (ppm)

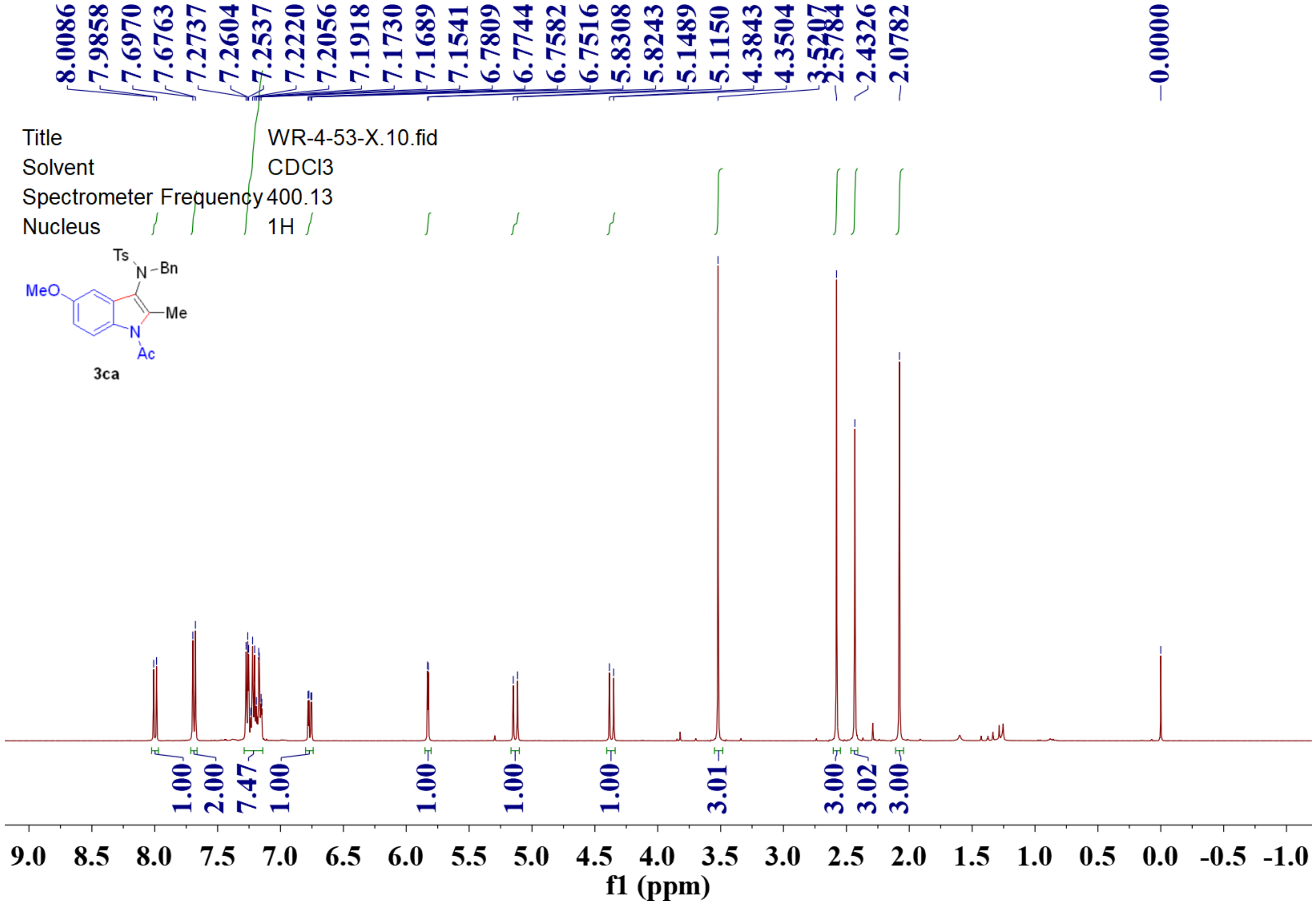

8.0086
7.9858
7.6970
7.6763
7.2737
7.2604
7.2537
7.2220
7.2056
7.1918
7.1730
7.1689
7.1541
6.7809
6.7744
6.7582
6.7516
5.8308
5.8243
5.1489
5.1150
4.3843
4.3504
3.5207
2.5784
2.4326
2.0782
0.0000
Title WR-4-53-X.10.fid
Solvent CDCl3
Spectrometer Frequency 400.13
Nucleus 1H
Ts
N
Bn
MeO
Me
N
Ac
3ca
1.00
2.00
7.47
1.00
1.00
1.00
1.00
3.01
3.00
3.02
3.00
9.0 8.5 8.0 7.5 7.0 6.5 6.0 5.5 5.0 4.5 4.0 3.5 3.0 2.5 2.0 1.5 1.0 0.5 0.0 -0.5 -1.0
f1 (ppm)

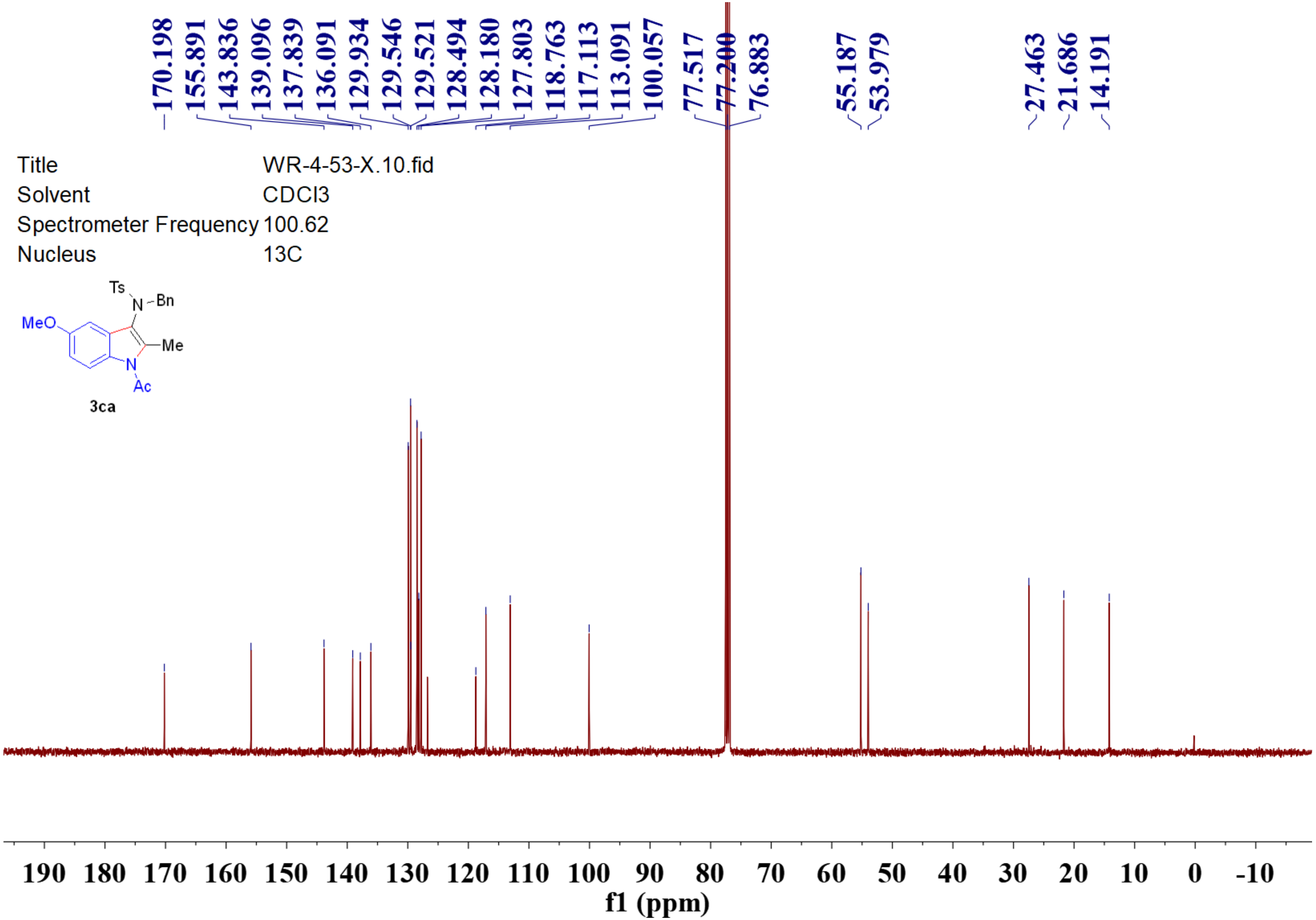
170.198
155.891
143.836
139.096
137.839
136.091
129.934
129.546
129.521
128.494
128.180
127.803
118.763
117.113
113.091
100.057
77.517
77.200
76.883
55.187
53.979
27.463
21.686
14.191
Title WR-4-53-X.10.fid
Solvent CDCl3
Spectrometer Frequency 100.62
Nucleus 13C
Ts
N
Bn
MeO
Me
N
Ac
3ca
190 180 170 160 150 140 130 120 110 100 90 80 70 60 50 40 30 20 10 0 -10
f1 (ppm)

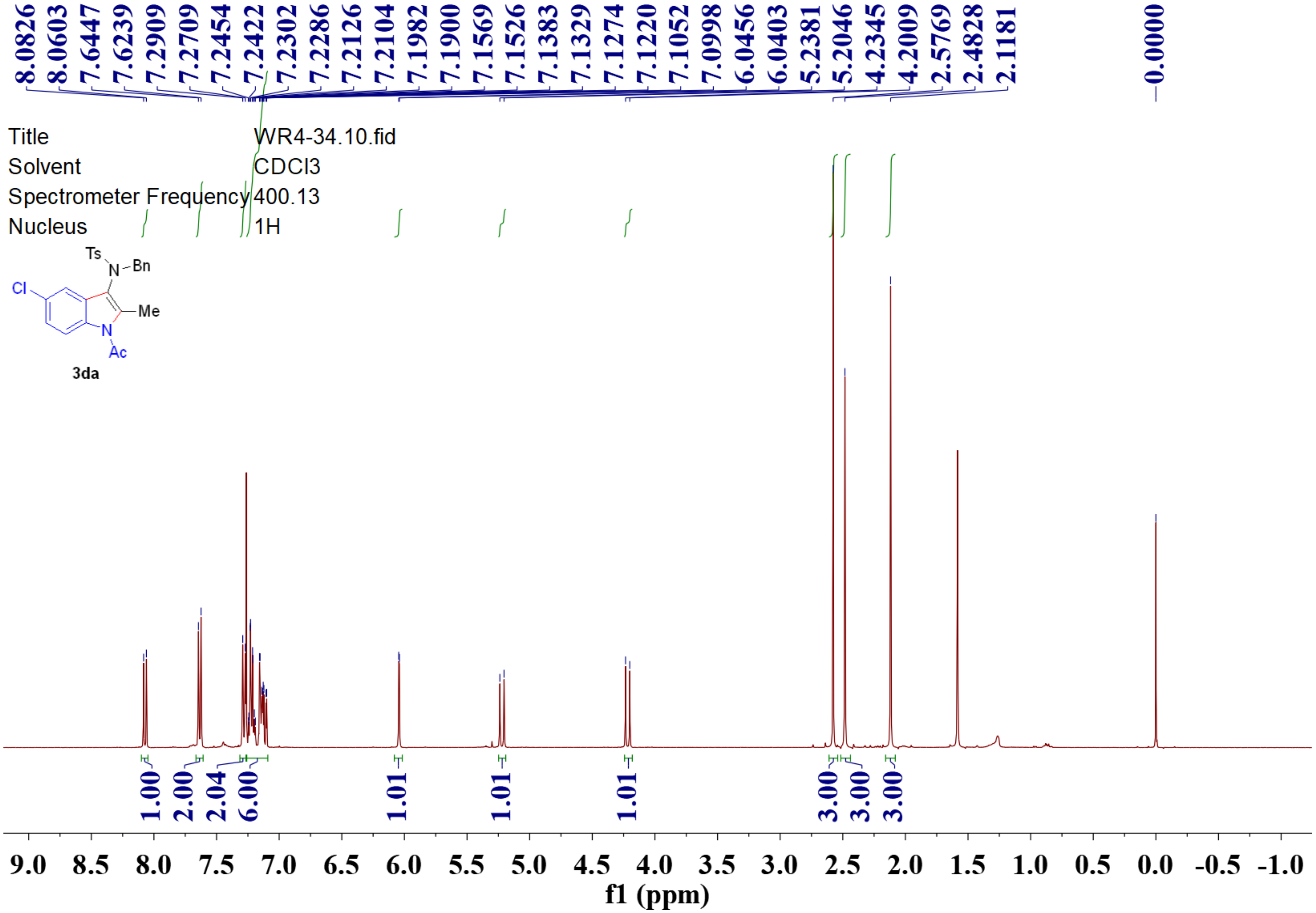
8.0826
8.0603
7.6447
7.6239
7.2909
7.2709
7.2454
7.2422
7.2302
7.2286
7.2126
7.2104
7.1982
7.1900
7.1569
7.1526
7.1383
7.1329
7.1274
7.1220
7.1052
7.0998
6.0456
6.0403
5.2381
5.2046
4.2345
4.2009
2.5769
2.4828
2.1181
-0.0000
Title WR4-34.10.fid
Solvent CDCl3
Spectrometer Frequency 400.13
Nucleus 1H
Ts
N
Bn
Cl
Me
N
Ac
3da
1.00
2.00
2.04
6.00
1.01
1.01
1.01
3.00
3.00
3.00
9.0 8.5 8.0 7.5 7.0 6.5 6.0 5.5 5.0 4.5 4.0 3.5 3.0 2.5 2.0 1.5 1.0 0.5 0.0 -0.5 -1.0
f1 (ppm)

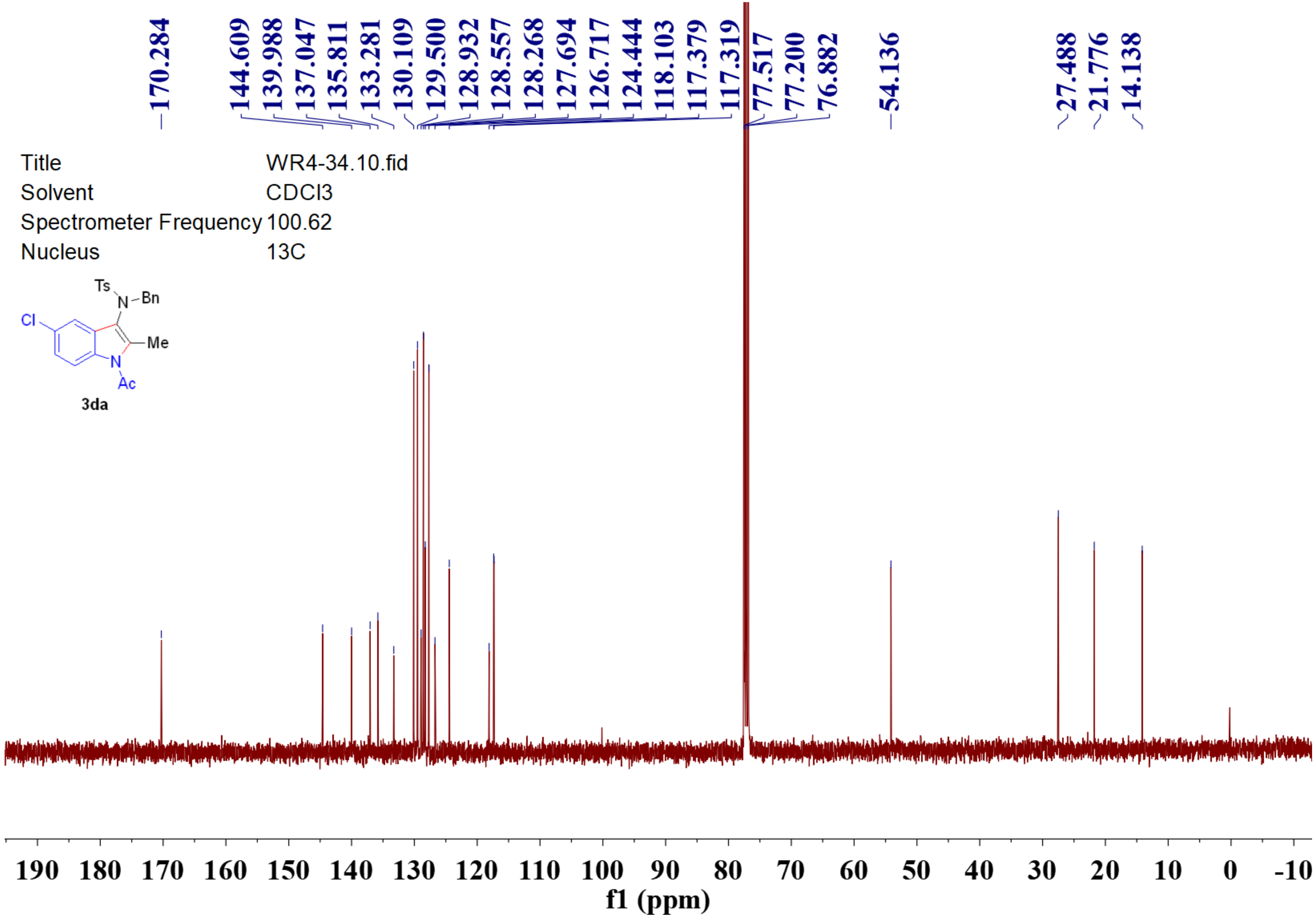
170.284
144.609
139.988
137.047
135.811
133.281
130.109
129.500
128.932
128.557
128.268
127.694
126.717
124.444
118.103
117.379
117.319
77.517
77.200
76.882
54.136
27.488
21.776
14.138
Title WR4-34.10.fid
Solvent CDCl3
Spectrometer Frequency 100.62
Nucleus 13C
Ts
N
Bn
Cl
Me
N
Ac
3da
190 180 170 160 150 140 130 120 110 100 90 80 70 60 50 40 30 20 10 0 -10
f1 (ppm)

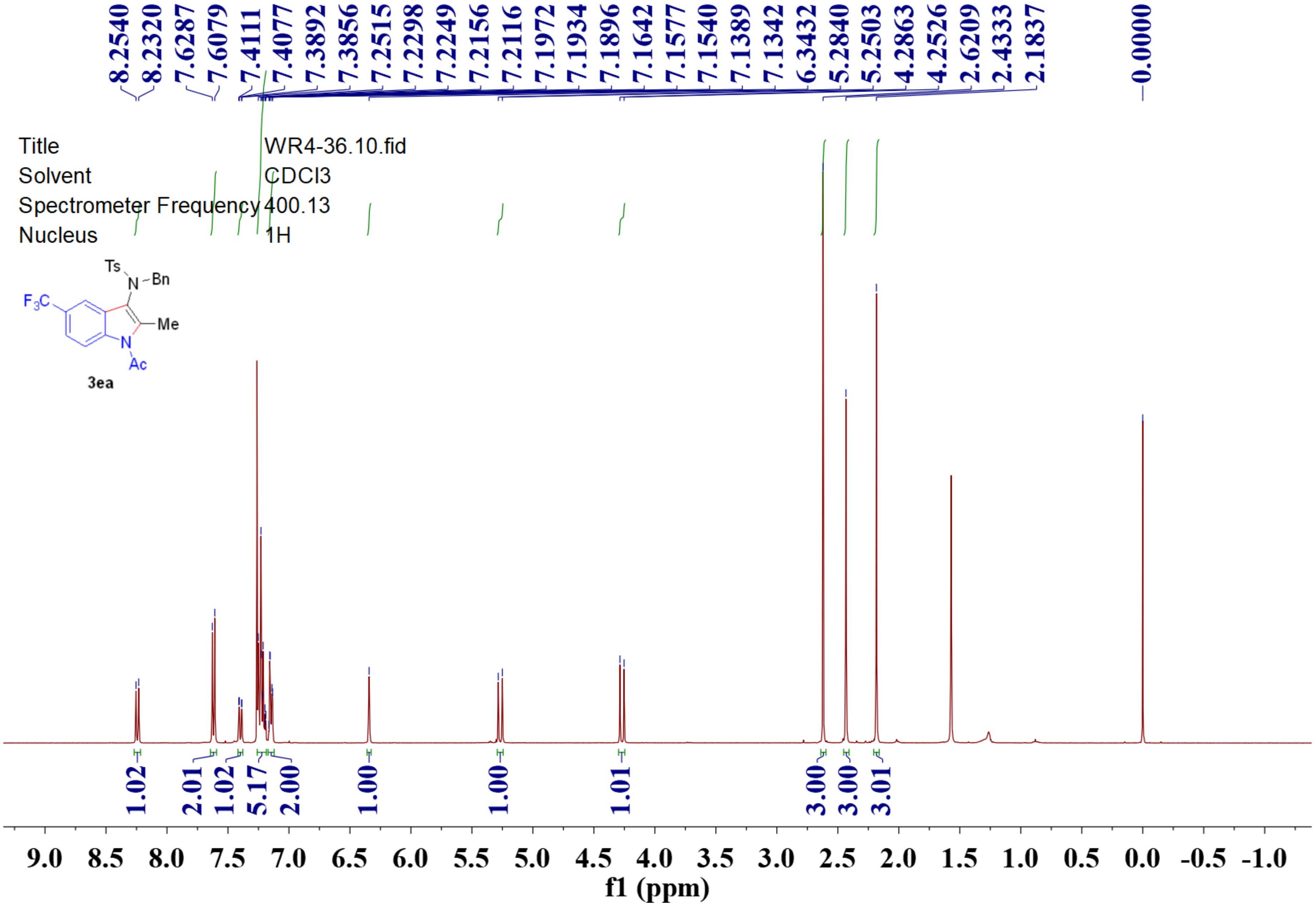

8.2540
8.2320
7.6287
7.6079
7.4111
7.4077
7.3892
7.3856
7.2515
7.2298
7.2249
7.2156
7.2116
7.1972
7.1934
7.1896
7.1642
7.1577
7.1540
7.1389
7.1342
6.3432
5.2840
5.2503
4.2863
4.2526
2.6209
2.4333
2.1837
-0.0000
Title WR4-36.10.fid
Solvent CDCl3
Spectrometer Frequency 400.13
Nucleus 1H
Ts
N
Bn
F3C
Me
N
Ac
3ea
1.02
2.01
1.02
5.17
2.00
1.00
1.00
1.01
3.00
3.00
3.01
9.0 8.5 8.0 7.5 7.0 6.5 6.0 5.5 5.0 4.5 4.0 3.5 3.0 2.5 2.0 1.5 1.0 0.5 0.0 -0.5 -1.0
f1 (ppm)

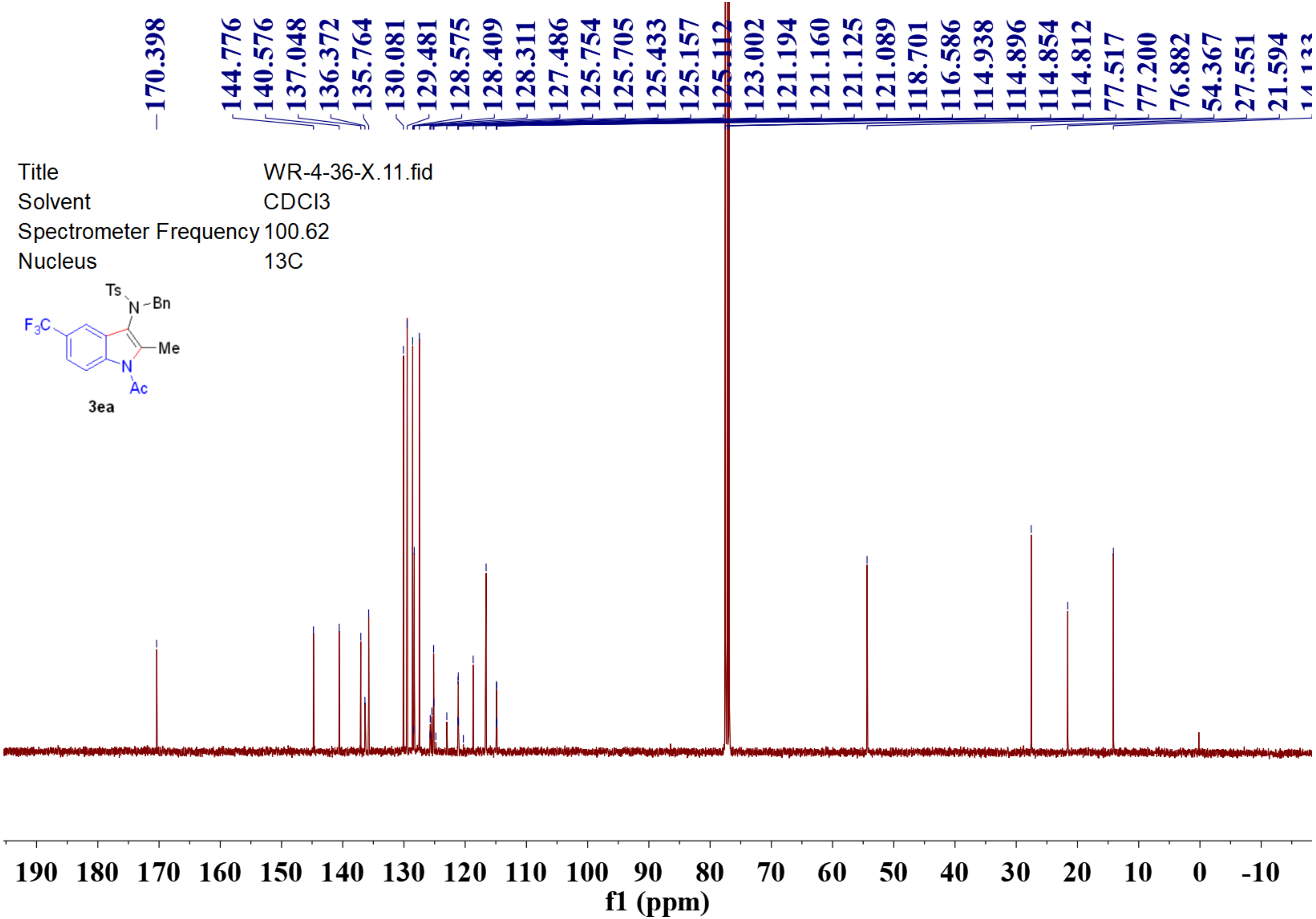
170.398
144.776
140.576
137.048
136.372
135.764
130.081
129.481
128.575
128.409
128.311
127.486
125.754
125.705
125.433
125.157
125.112
123.002
121.194
121.160
121.125
121.089
118.701
116.586
114.938
114.896
114.854
114.812
77.517
77.200
76.882
54.367
27.551
21.594
Title WR-4-36-X.11.fid
Solvent CDCl3
Spectrometer Frequency 100.62
Nucleus 13C
Ts
N
Bn
F3C
Me
N
Ac
3ea
190 180 170 160 150 140 130 120 110 100 90 80 70 60 50 40 30 20 10 0 -10
f1 (ppm)

Title WR4-36F.10.fid
Solvent CDCl3
Spectrometer Frequency 376.46
Nucleus 19F

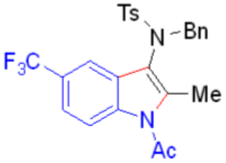

3ea

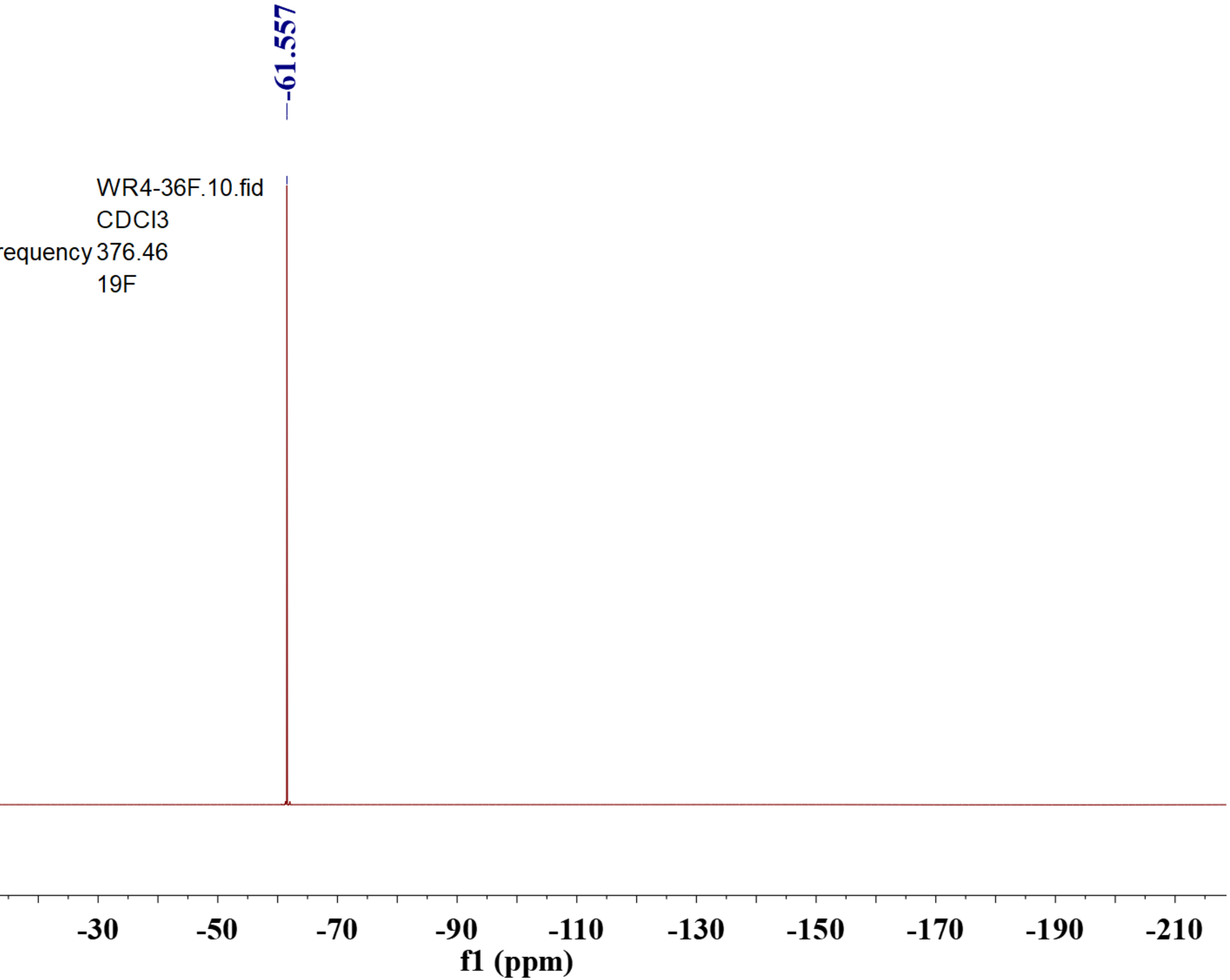

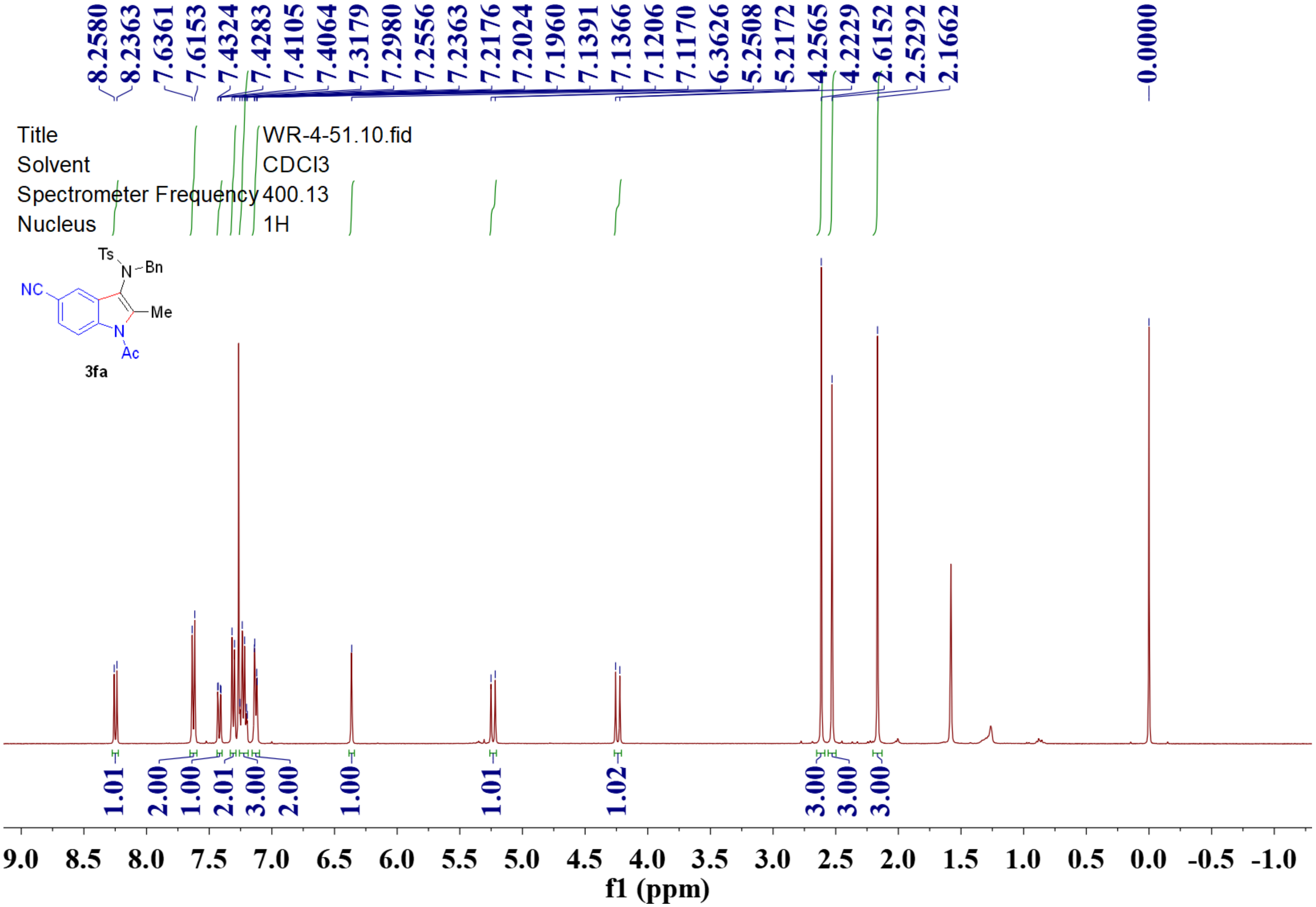

8.2580
8.2363
7.6361
7.6153
7.4324
7.4283
7.4105
7.4064
7.3179
7.2980
7.2556
7.2363
7.2176
7.2024
7.1960
7.1391
7.1366
7.1206
7.1170
6.3626
5.2508
5.2172
4.2565
4.2229
2.6152
2.5292
2.1662
0.0000
Title WR-4-51.10.fid
Solvent CDCl3
Spectrometer Frequency 400.13
Nucleus 1H
Ts
N
Bn
NC
Me
N
Ac
3fa
1.01
2.00
1.00
2.01
3.00
2.00
1.00
1.01
1.02
3.00
3.00
3.00
9.0 8.5 8.0 7.5 7.0 6.5 6.0 5.5 5.0 4.5 4.0 3.5 3.0 2.5 2.0 1.5 1.0 0.5 0.0 -0.5 -1.0
f1 (ppm)

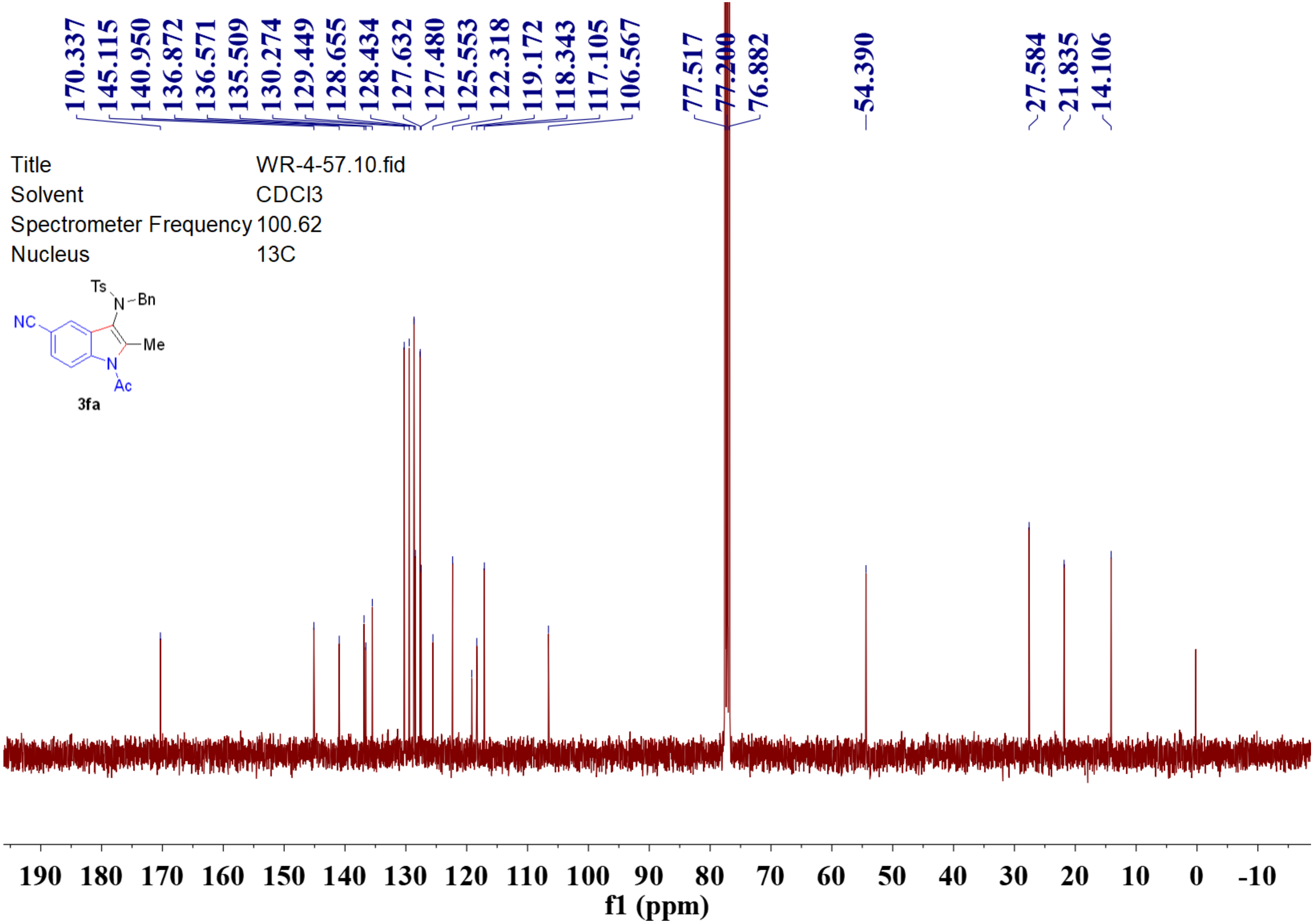

170.337
145.115
140.950
136.872
136.571
135.509
130.274
129.449
128.655
128.434
127.632
127.480
125.553
122.318
119.172
118.343
117.105
106.567
77.517
77.200
76.882
54.390
27.584
21.835
14.106
Title WR-4-57.10.fid
Solvent CDCl3
Spectrometer Frequency 100.62
Nucleus 13C
Ts
N
Bn
NC
Me
N
Ac
3fa
190 180 170 160 150 140 130 120 110 100 90 80 70 60 50 40 30 20 10 0 -10
f1 (ppm)

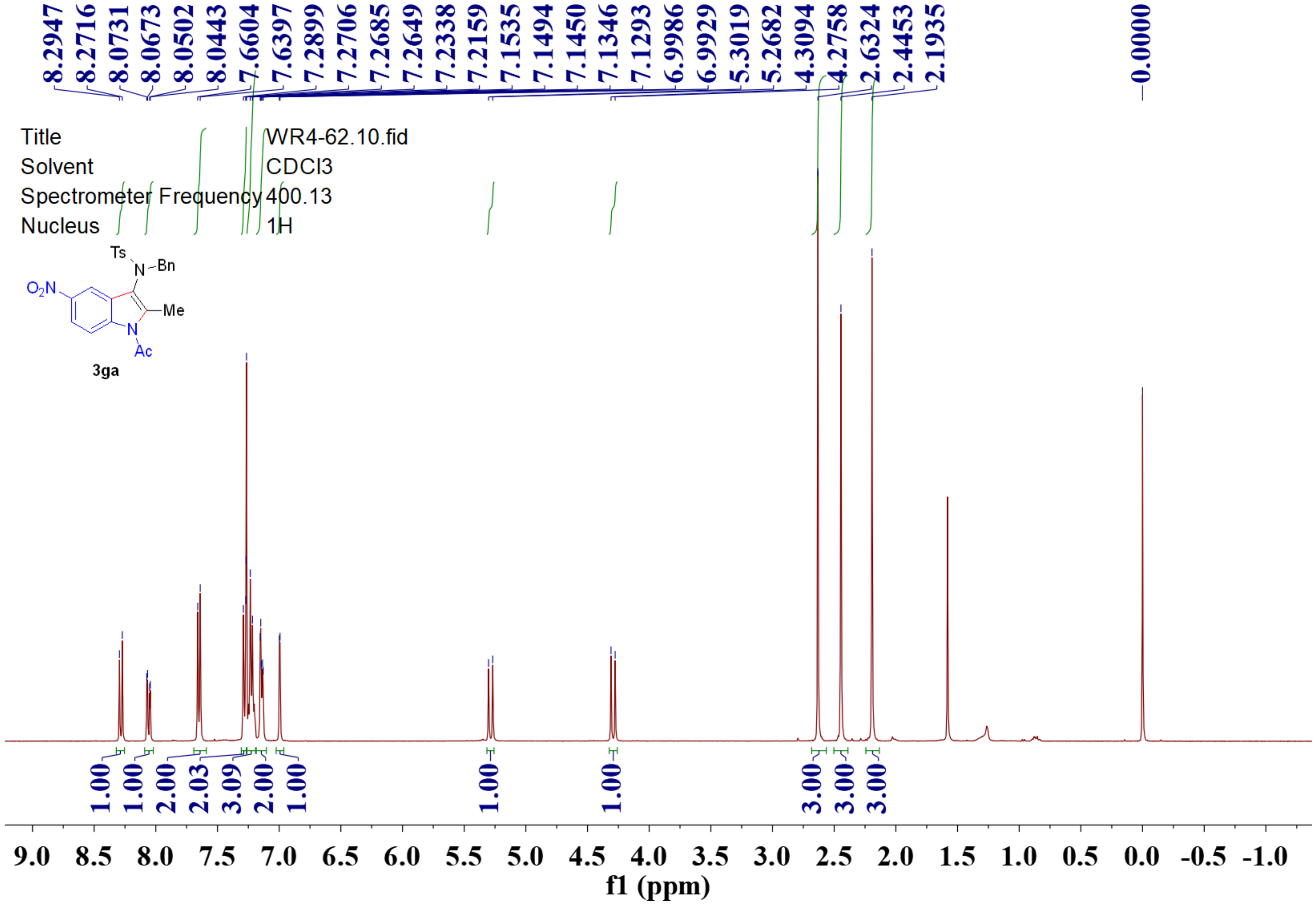
8.2947
8.2716
8.0731
8.0673
8.0502
8.0443
7.6604
7.6397
7.2899
7.2706
7.2685
7.2649
7.2338
7.2159
7.1535
7.1494
7.1450
7.1346
7.1293
6.9986
6.9929
5.3019
5.2682
4.3094
4.2758
2.6324
2.4453
2.1935
-0.0000
Title WR4-62.10.fid
Solvent CDCl3
Spectrometer Frequency 400.13
Nucleus 1H
Ts
N
Bn
O2N
Me
N
Ac
3ga
1.00
1.00
2.00
2.03
3.09
2.00
1.00
1.00
1.00
3.00
3.00
3.00
9.0 8.5 8.0 7.5 7.0 6.5 6.0 5.5 5.0 4.5 4.0 3.5 3.0 2.5 2.0 1.5 1.0 0.5 0.0 -0.5 -1.0
f1 (ppm)

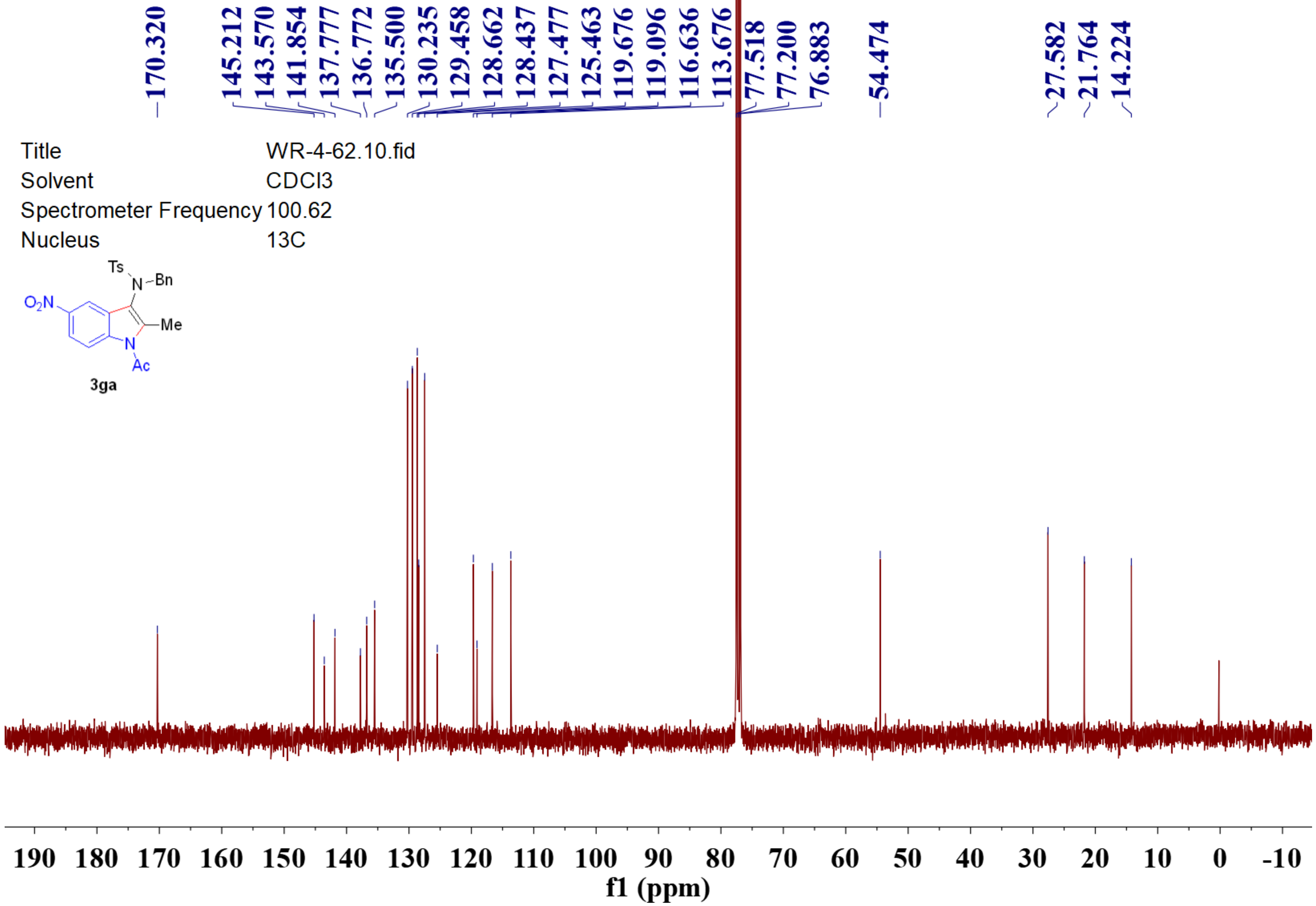
170.320
145.212
143.570
141.854
137.777
136.772
135.500
130.235
129.458
128.662
128.437
127.477
125.463
119.676
119.096
116.636
113.676
77.518
77.200
76.883
54.474
27.582
21.764
14.224
Title WR-4-62.10.fid
Solvent CDCl3
Spectrometer Frequency 100.62
Nucleus 13C
Ts
N
Bn
O2N
Me
N
Ac
3ga
190 180 170 160 150 140 130 120 110 100 90 80 70 60 50 40 30 20 10 0 -10
f1 (ppm)

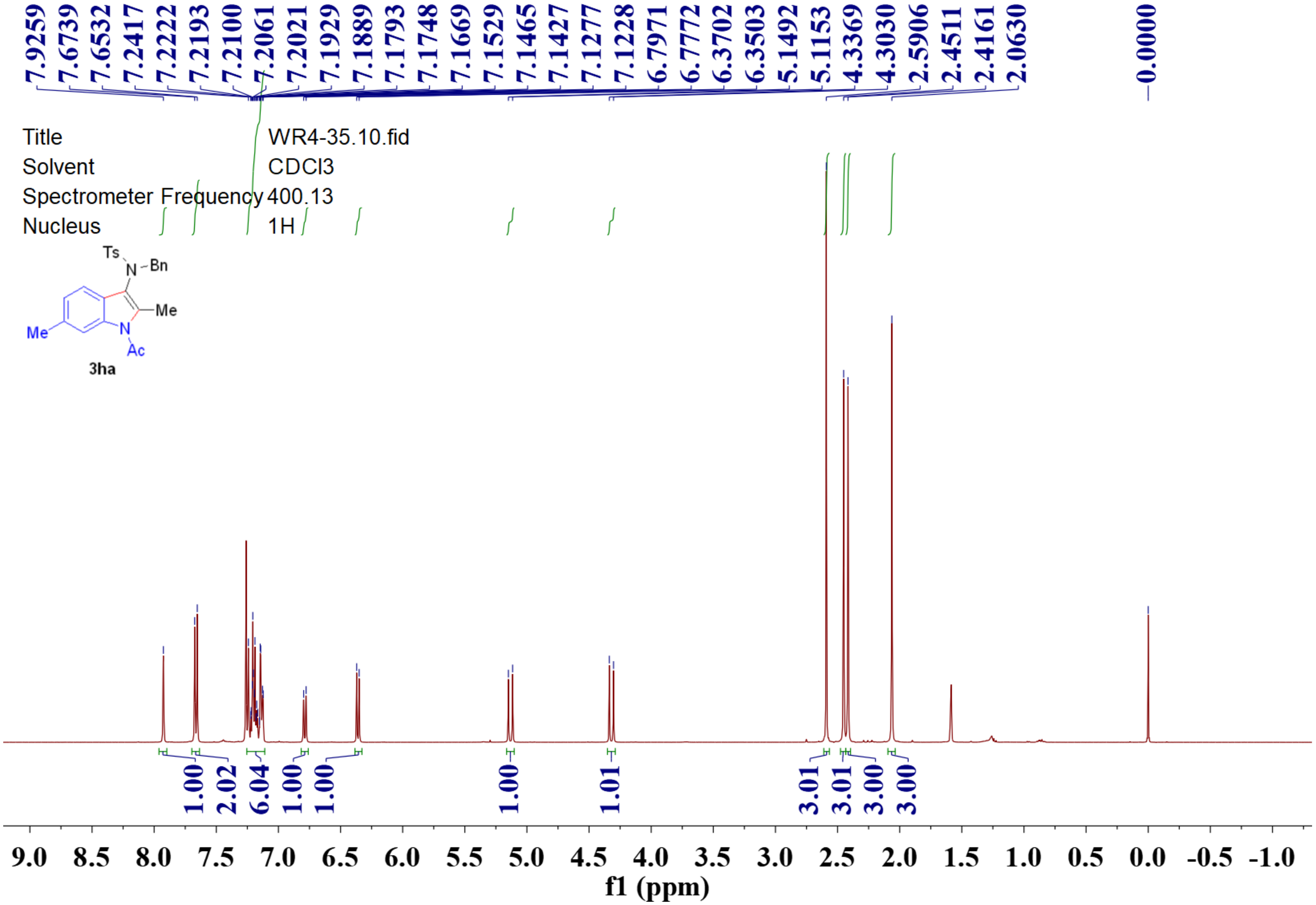
7.9259 7.6739 7.6532 7.2417 7.2222 7.2193 7.2100 7.2061 7.2021 7.1929 7.1889 7.1793 7.1748 7.1669 7.1529 7.1465 7.1427 7.1277 7.1228 6.7971 6.7772 6.3702 6.3503 5.1492 5.1153 4.3369 4.3030 2.5906 2.4511 2.4161 2.0630
0.0000
Title WR4-35.10.fid
Solvent CDCl3
Spectrometer Frequency 400.13
Nucleus 1H
Ts
N
Bn
Me
Me
N
Ac
3ha
1.00 2.02 6.04 1.00 1.00 1.00 1.01 3.01 3.01 3.00 3.00
9.0 8.5 8.0 7.5 7.0 6.5 6.0 5.5 5.0 4.5 4.0 3.5 3.0 2.5 2.0 1.5 1.0 0.5 0.0 -0.5 -1.0
f1 (ppm)

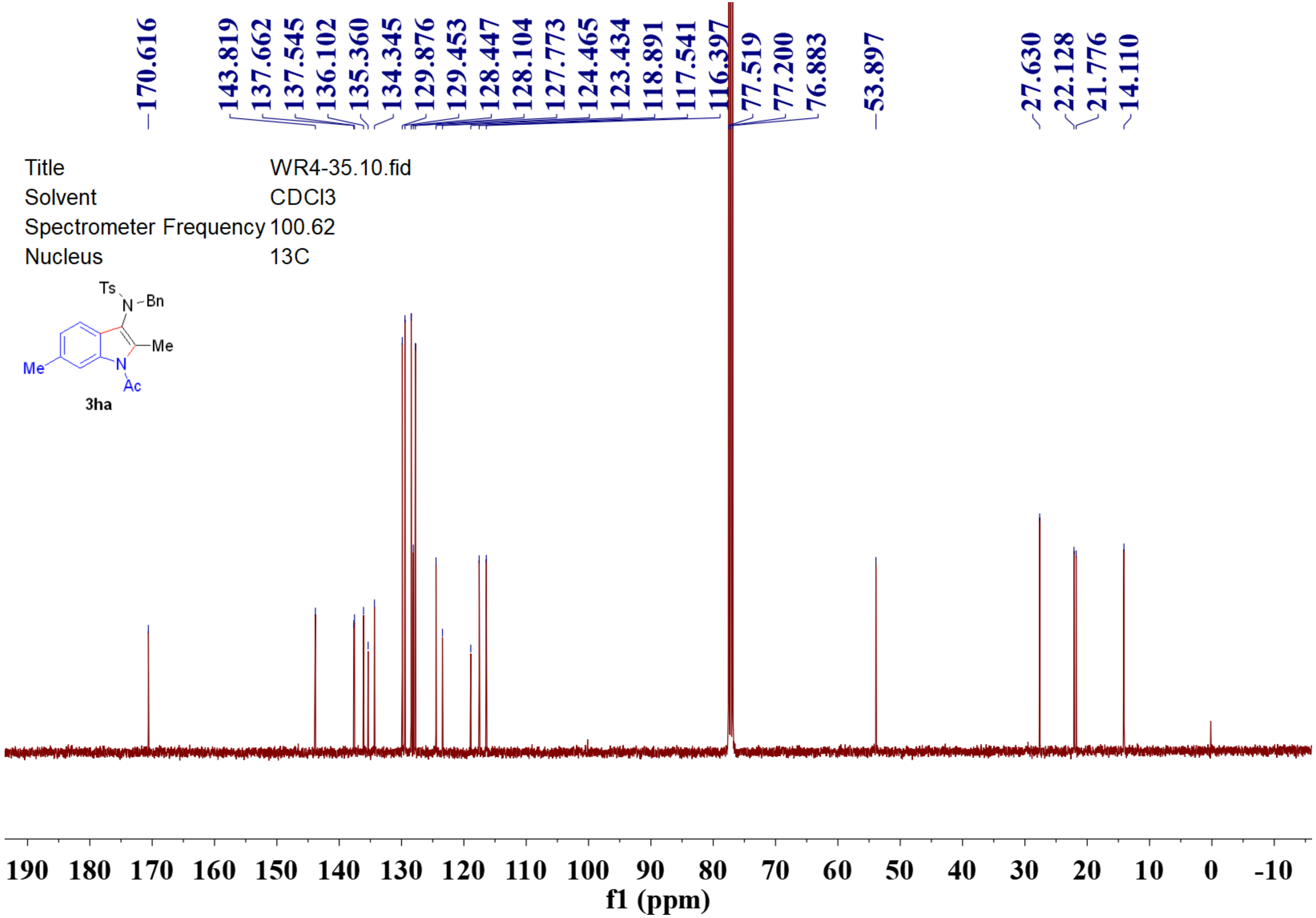

170.616
143.819
137.662
137.545
136.102
135.360
134.345
129.876
129.453
128.447
128.104
127.773
124.465
123.434
118.891
117.541
116.397
77.519
77.200
76.883
53.897
27.630
22.128
21.776
14.110
Title WR4-35.10.fid
Solvent CDCl3
Spectrometer Frequency 100.62
Nucleus 13C
Ts
N
Bn
Me
Me
N
Ac
3ha
190 180 170 160 150 140 130 120 110 100 90 80 70 60 50 40 30 20 10 0 -10
f1 (ppm)

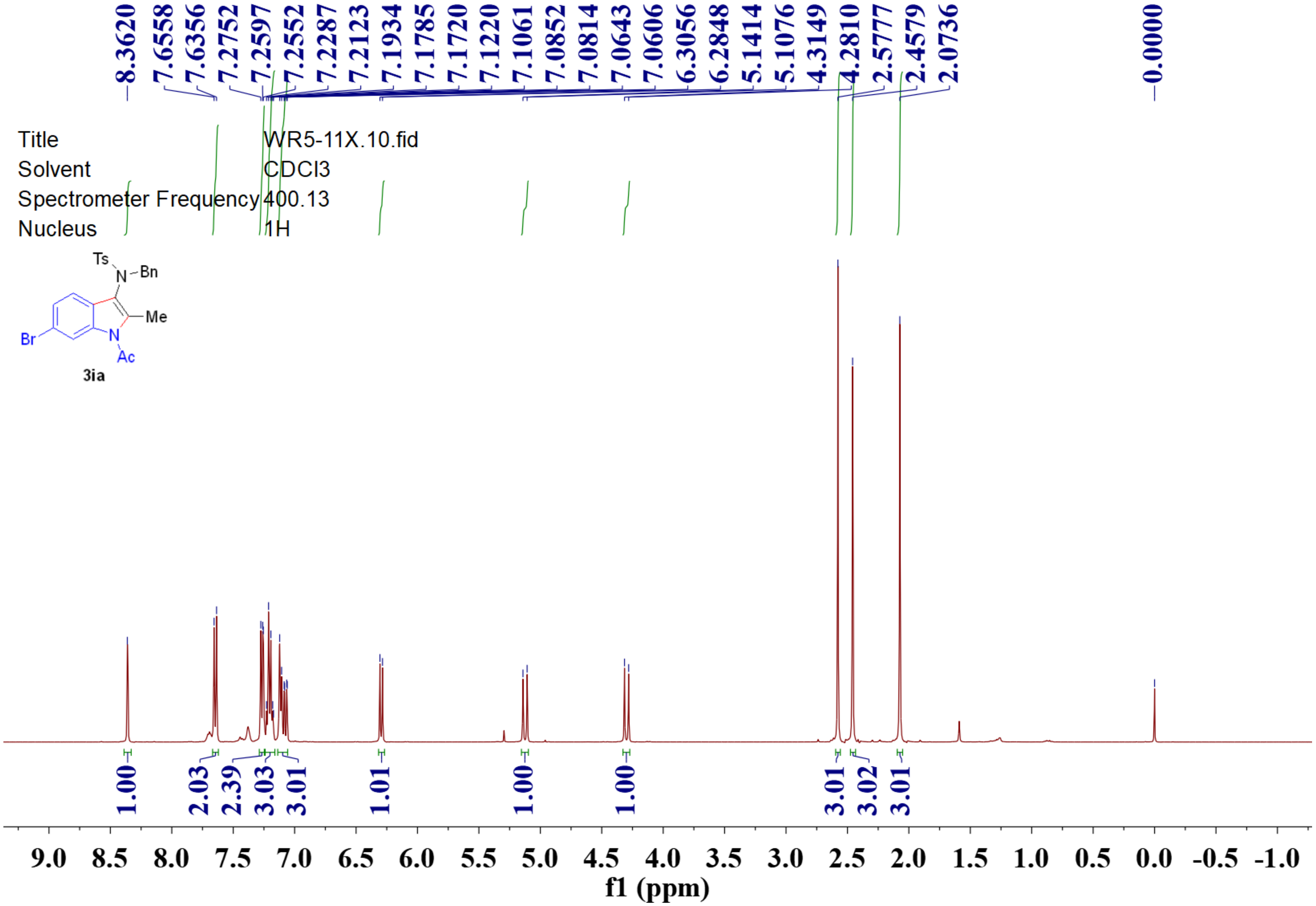
8.3620
7.6558
7.6356
7.2752
7.2597
7.2552
7.2287
7.2123
7.1934
7.1785
7.1720
7.1220
7.1061
7.0852
7.0814
7.0643
7.0606
6.3056
6.2848
5.1414
5.1076
4.3149
4.2810
2.5777
2.4579
2.0736
0.0000
Title WR5-11X.10.fid
Solvent CDCl3
Spectrometer Frequency 400.13
Nucleus 1H
Ts
N
Bn
Me
Br
N
Ac
3ia
1.00
2.03
2.39
3.03
3.01
1.01
1.00
1.00
3.01
3.02
3.01
9.0 8.5 8.0 7.5 7.0 6.5 6.0 5.5 5.0 4.5 4.0 3.5 3.0 2.5 2.0 1.5 1.0 0.5 0.0 -0.5 -1.0
f1 (ppm)

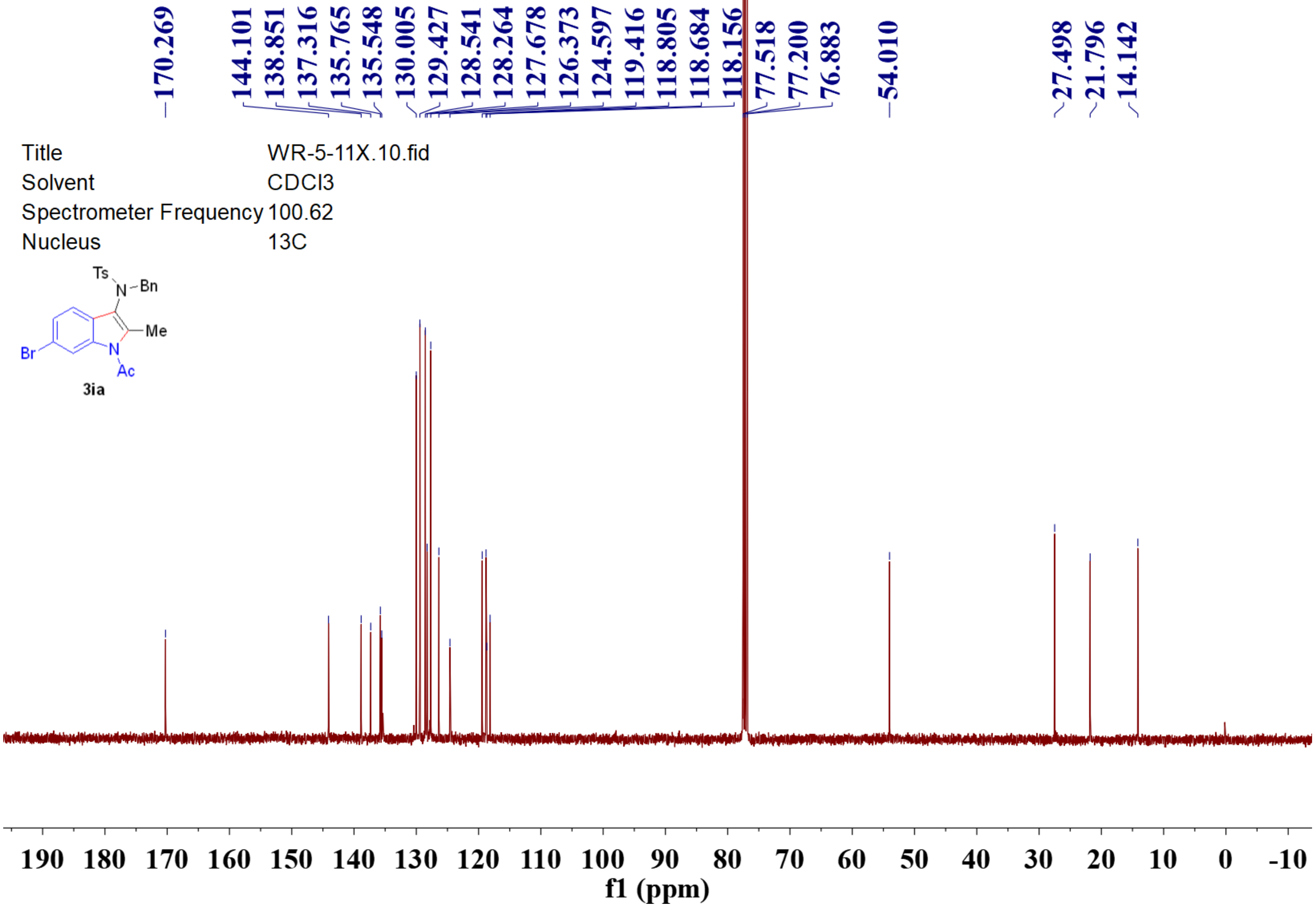
170.269
144.101
138.851
137.316
135.765
135.548
130.005
129.427
128.541
128.264
127.678
126.373
124.597
119.416
118.805
118.684
118.156
77.518
77.200
76.883
54.010
27.498
21.796
14.142
Title WR-5-11X.10.fid
Solvent CDCl3
Spectrometer Frequency 100.62
Nucleus 13C
Ts
N
Bn
Me
Br
N
Ac
3ia
190 180 170 160 150 140 130 120 110 100 90 80 70 60 50 40 30 20 10 0 -10
f1 (ppm)

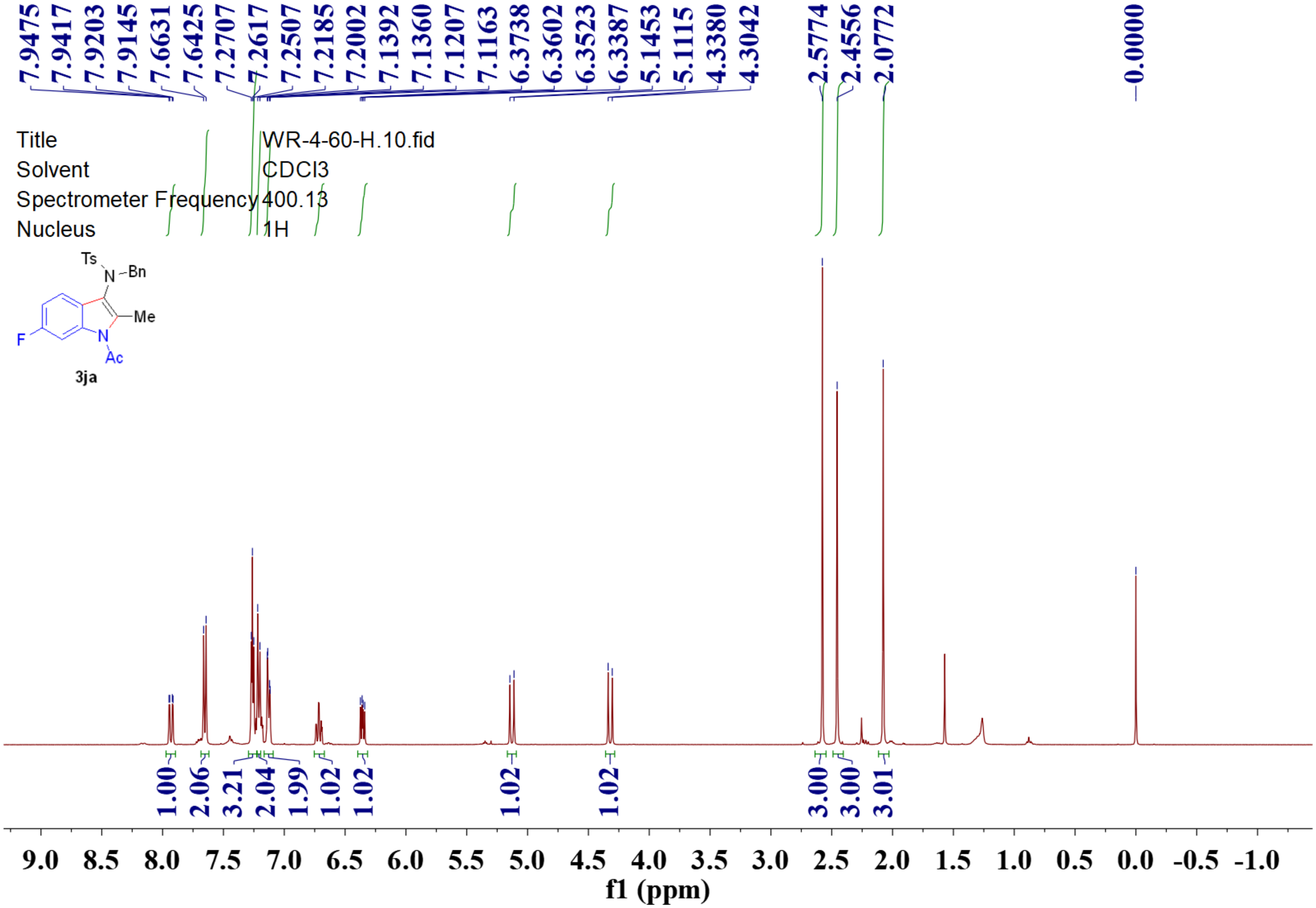
7.9475
7.9417
7.9203
7.9145
7.6631
7.6425
7.2707
7.2617
7.2507
7.2185
7.2002
7.1392
7.1360
7.1207
7.1163
6.3738
6.3602
6.3523
6.3387
5.1453
5.1115
4.3380
4.3042
2.5774
2.4556
2.0772
-0.0000
Title WR-4-60-H.10.fid
Solvent CDCl3
Spectrometer Frequency 400.13
Nucleus 1H
Ts
N
Bn
Me
F
N
Ac
3ja
1.00
2.06
3.21
2.04
1.99
1.02
1.02
1.02
1.02
3.00
3.00
3.01
9.0 8.5 8.0 7.5 7.0 6.5 6.0 5.5 5.0 4.5 4.0 3.5 3.0 2.5 2.0 1.5 1.0 0.5 0.0 -0.5 -1.0
f1 (ppm)

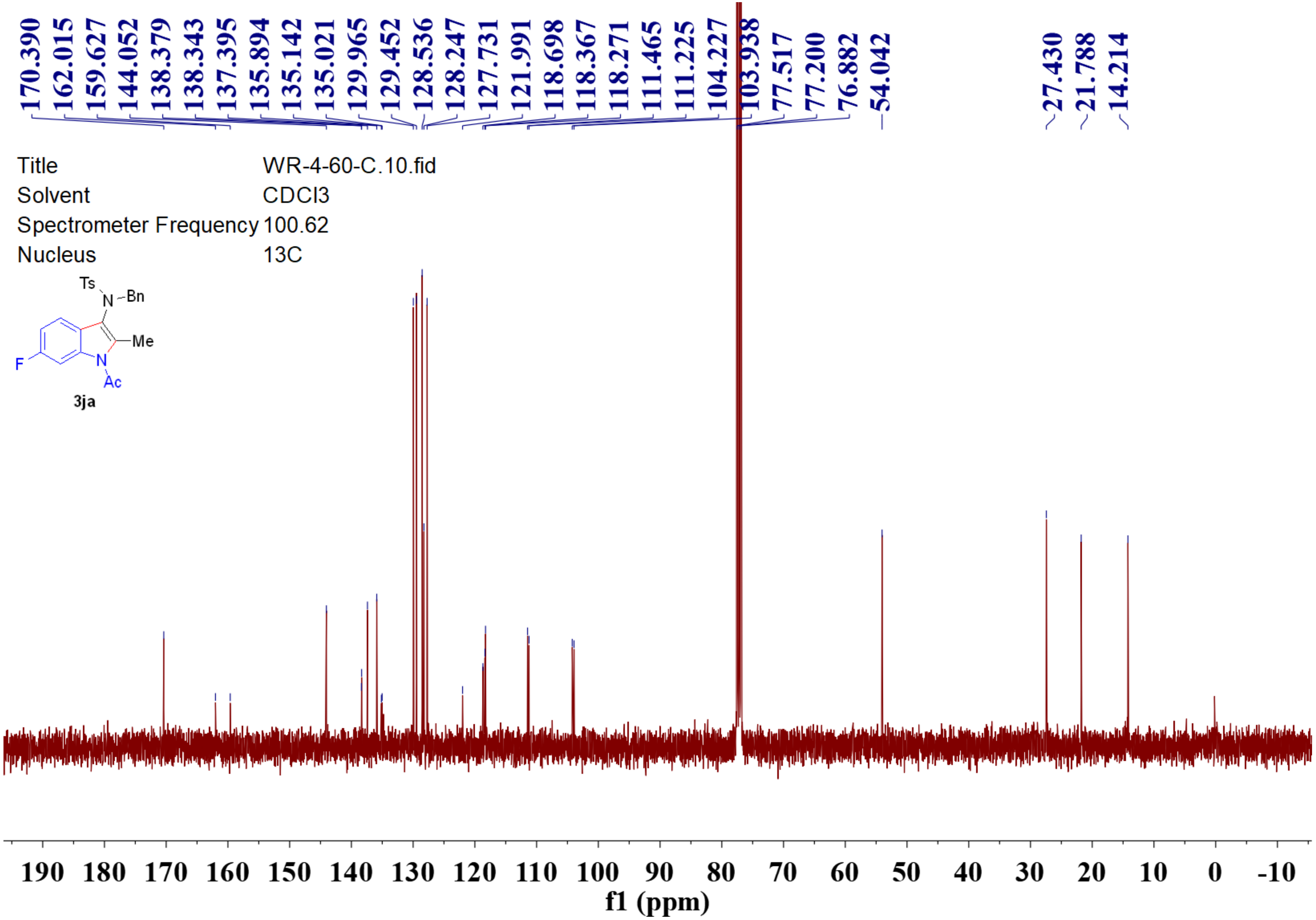

170.390
162.015
159.627
144.052
138.379
138.343
137.395
135.894
135.142
135.021
129.965
129.452
128.536
128.247
127.731
121.991
118.698
118.367
118.271
111.465
111.225
104.227
103.938
77.517
77.200
76.882
54.042
27.430
21.788
14.214
Title WR-4-60-C.10.fid
Solvent CDCl3
Spectrometer Frequency 100.62
Nucleus 13C
Ts
N
Bn
Me
F
N
Ac
3ja
190 180 170 160 150 140 130 120 110 100 90 80 70 60 50 40 30 20 10 0 -10
f1 (ppm)

Title WR-4-60-C.11.fid
Solvent CDCl3
Spectrometer Frequency 376.46
Nucleus 19F

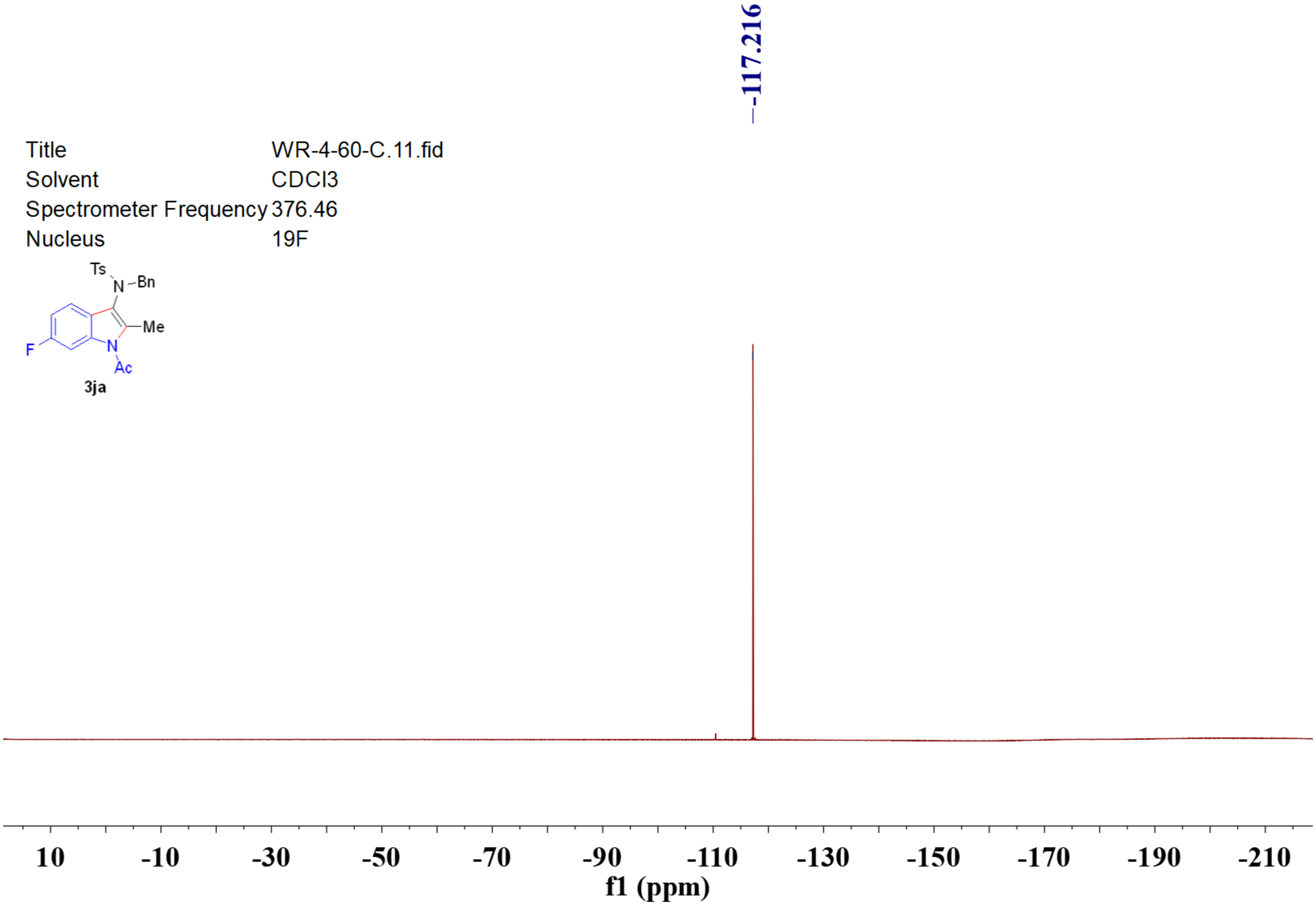

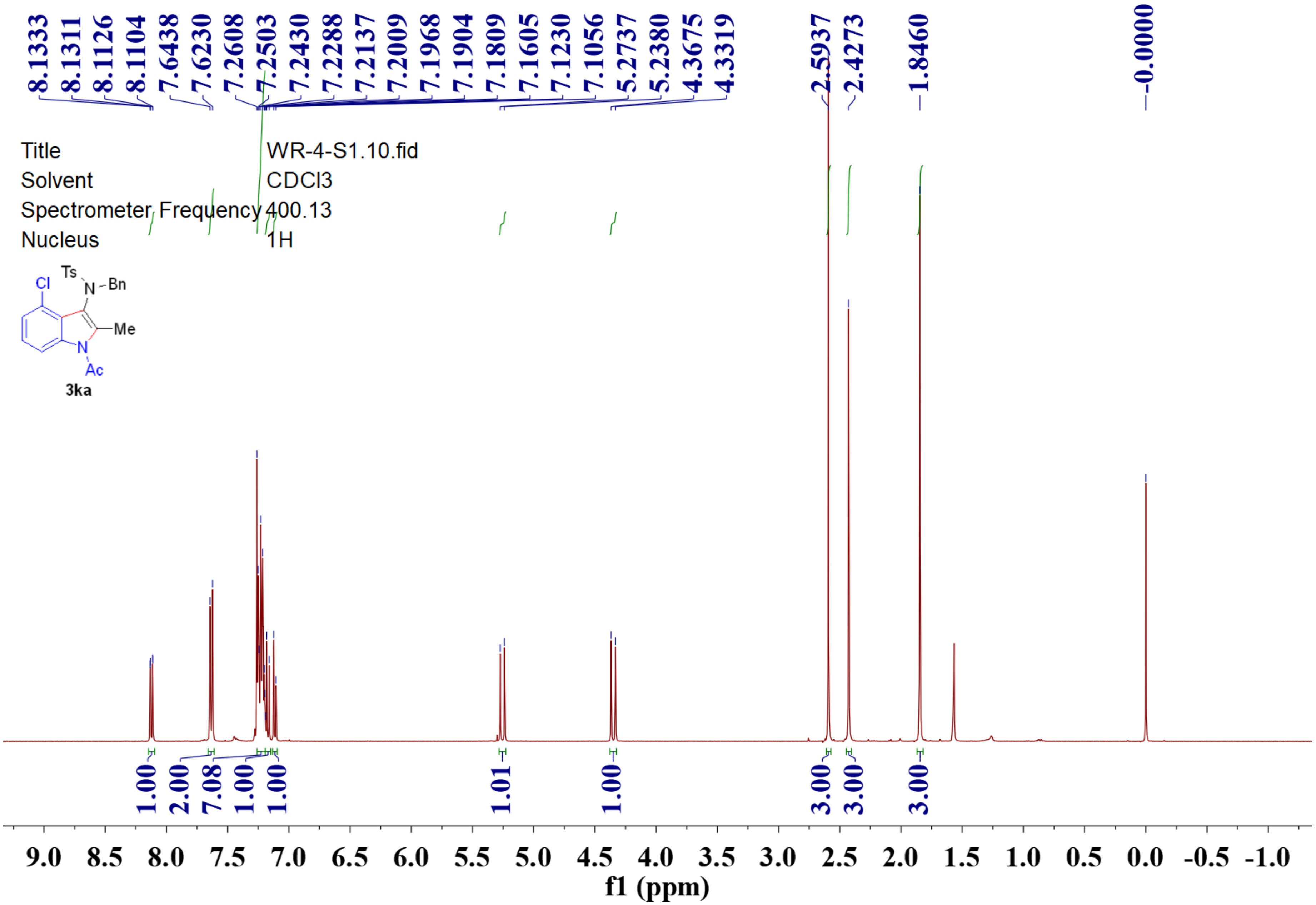
8.1333
8.1311
8.1126
8.1104
7.6438
7.6230
7.2608
7.2503
7.2430
7.2288
7.2137
7.2009
7.1968
7.1904
7.1809
7.1605
7.1230
7.1056
5.2737
5.2380
4.3675
4.3319
2.5937
2.4273
1.8460
-0.0000
Title
WR-4-S1.10.fid
Solvent
CDCl3
Spectrometer Frequency
400.13
Nucleus
1H
Cl
Ts
N
Bn
Me
N
Ac
3ka
1.00
2.00
7.08
1.00
1.00
1.01
1.00
3.00
3.00
3.00
9.0 8.5 8.0 7.5 7.0 6.5 6.0 5.5 5.0 4.5 4.0 3.5 3.0 2.5 2.0 1.5 1.0 0.5 0.0 -0.5 -1.0
f1 (ppm)

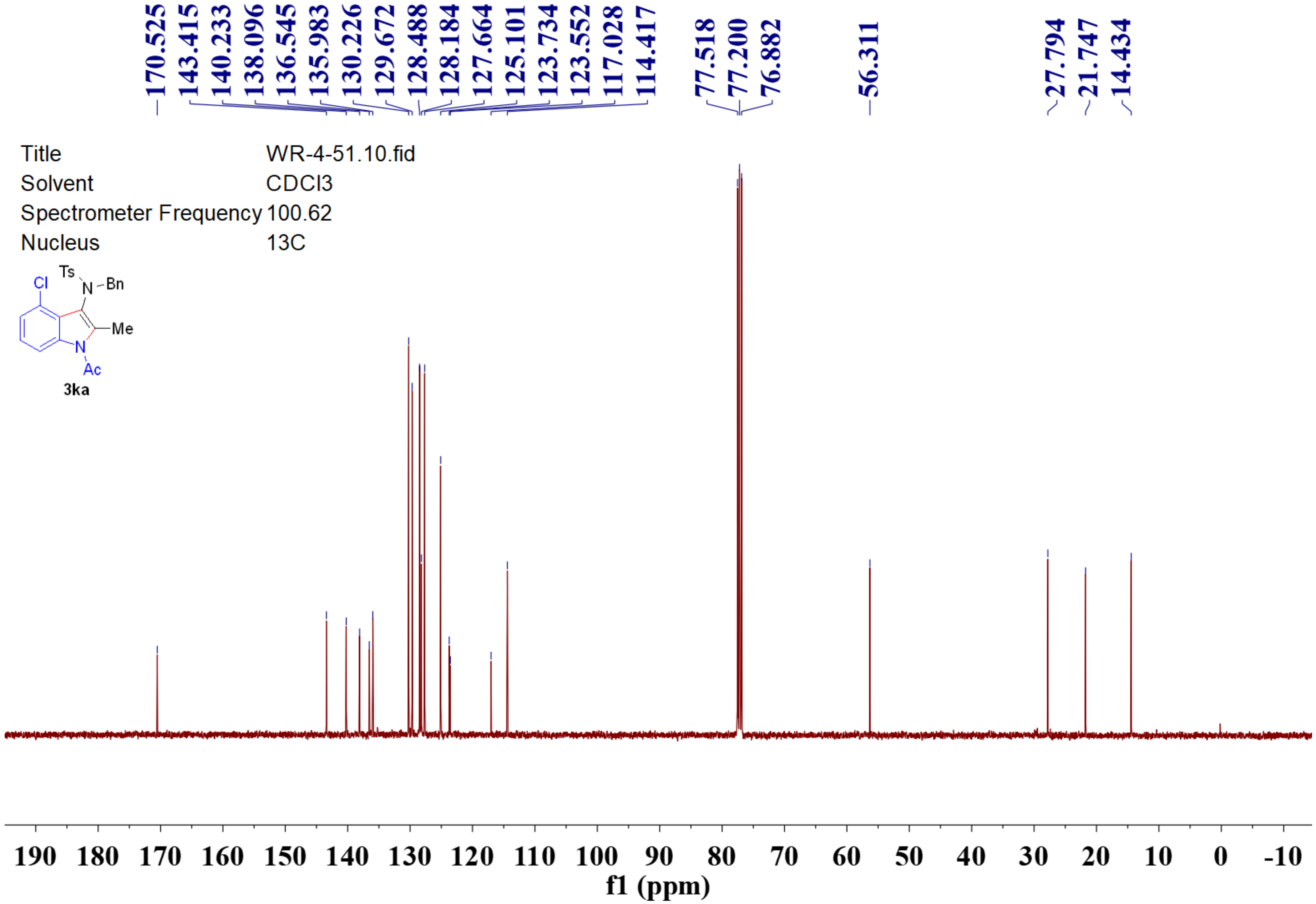
170.525
143.415
140.233
138.096
136.545
135.983
130.226
129.672
128.488
128.184
127.664
125.101
123.734
123.552
117.028
114.417
77.518
77.200
76.882
56.311
27.794
21.747
14.434
Title WR-4-51.10.fid
Solvent CDCl3
Spectrometer Frequency 100.62
Nucleus 13C
Cl
Ts
N
Bn
Me
N
Ac
3ka
190 180 170 160 150 140 130 120 110 100 90 80 70 60 50 40 30 20 10 0 -10
f1 (ppm)

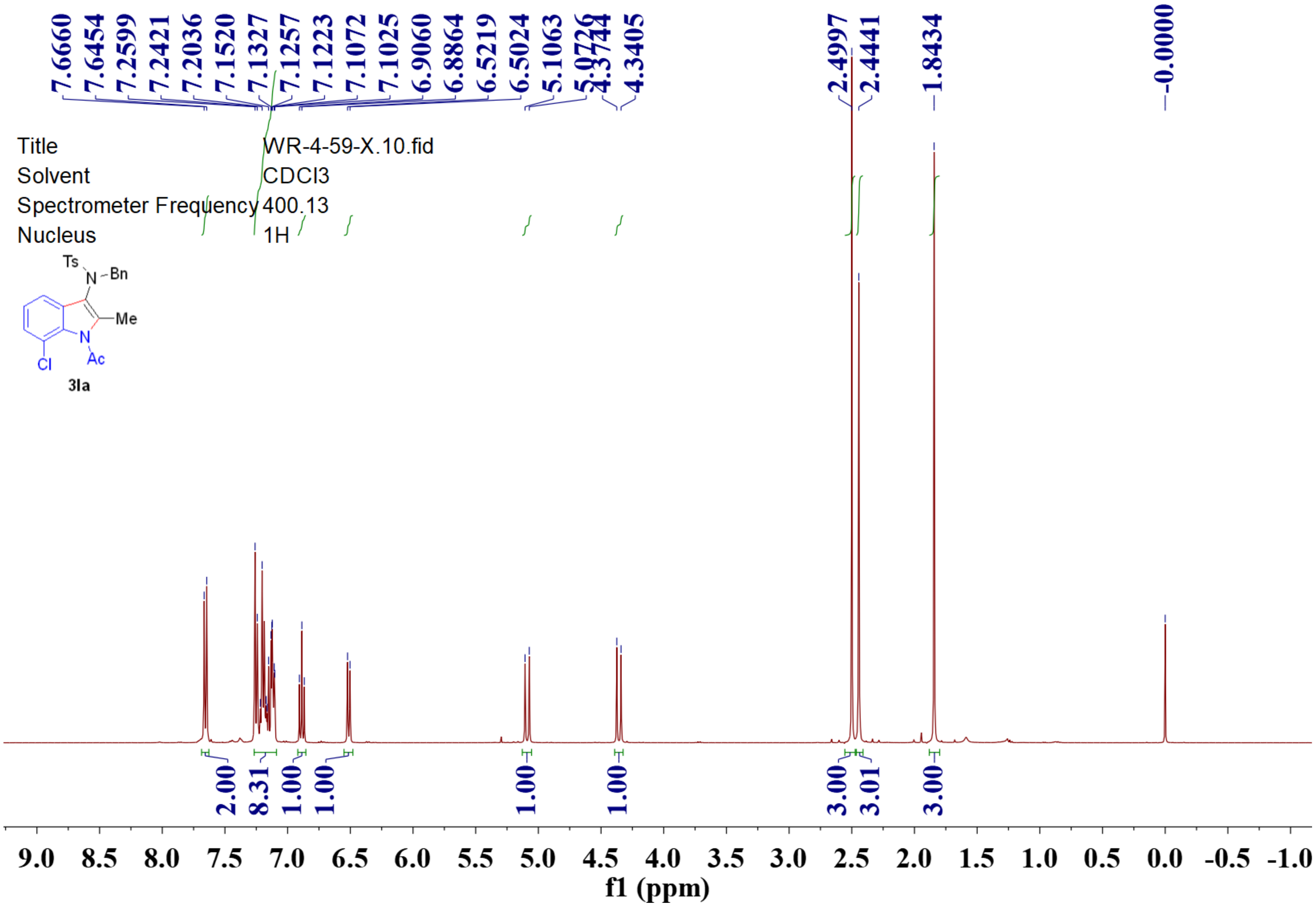
7.6660
7.6454
7.2599
7.2421
7.2036
7.1520
7.1327
7.1257
7.1223
7.1072
7.1025
6.9060
6.8864
6.5219
6.5024
5.1063
5.0726
4.3744
4.3405
2.4997
2.4441
1.8434
-0.0000
Title WR-4-59-X.10.fid
Solvent CDCl3
Spectrometer Frequency 400.13
Nucleus 1H
Ts
N
Bn
Me
N
Cl
Ac
3la
2.00
8.31
1.00
1.00
1.00
1.00
3.00
3.01
3.00
9.0 8.5 8.0 7.5 7.0 6.5 6.0 5.5 5.0 4.5 4.0 3.5 3.0 2.5 2.0 1.5 1.0 0.5 0.0 -0.5 -1.0
f1 (ppm)

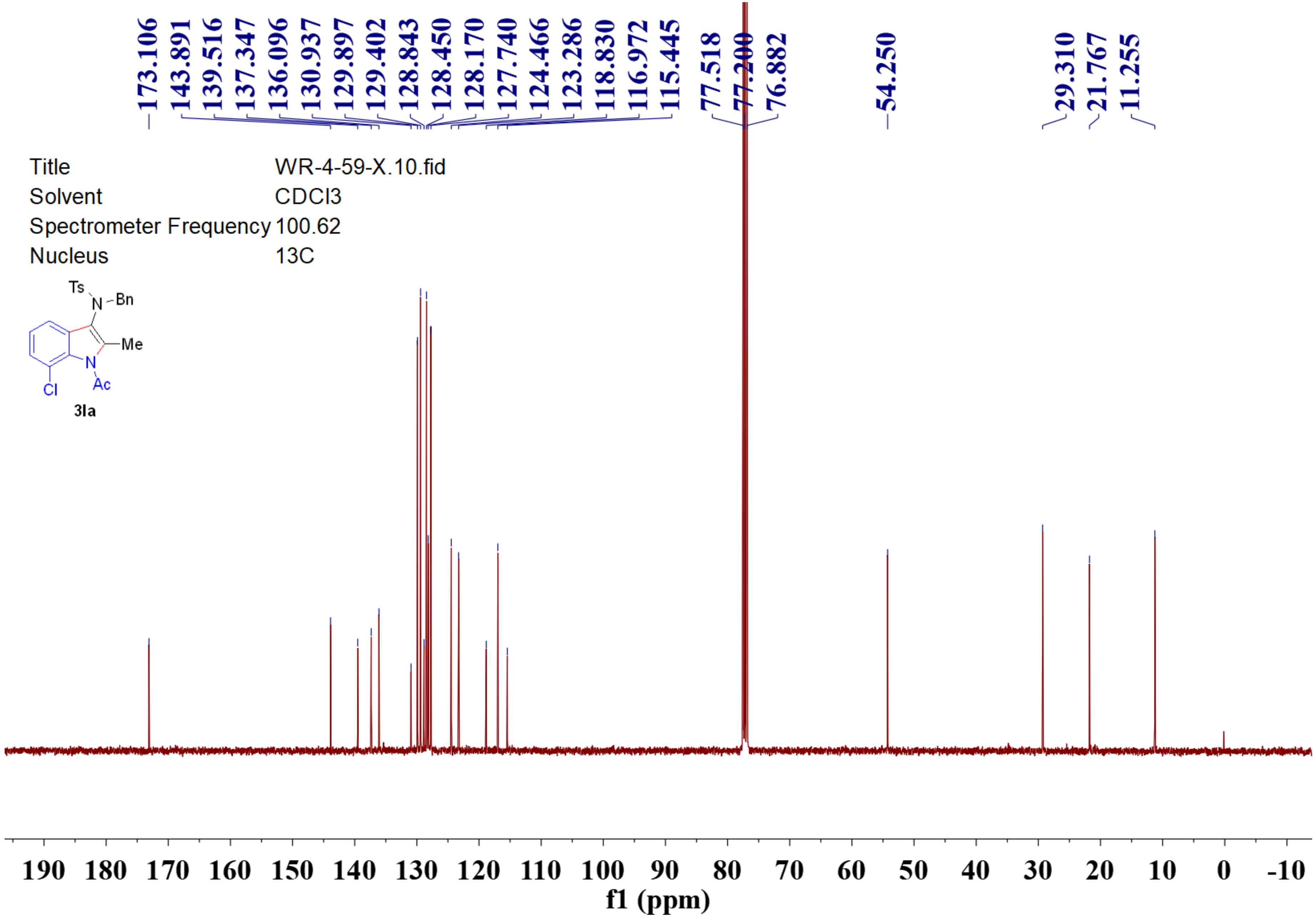

173.106
143.891
139.516
137.347
136.096
130.937
129.897
129.402
128.843
128.450
128.170
127.740
124.466
123.286
118.830
116.972
115.445
77.518
77.200
76.882
54.250
29.310
21.767
11.255
Title WR-4-59-X.10.fid
Solvent CDCl3
Spectrometer Frequency 100.62
Nucleus 13C
Ts
N
Bn
Me
N
Ac
Cl
3la
190 180 170 160 150 140 130 120 110 100 90 80 70 60 50 40 30 20 10 0 -10
f1 (ppm)

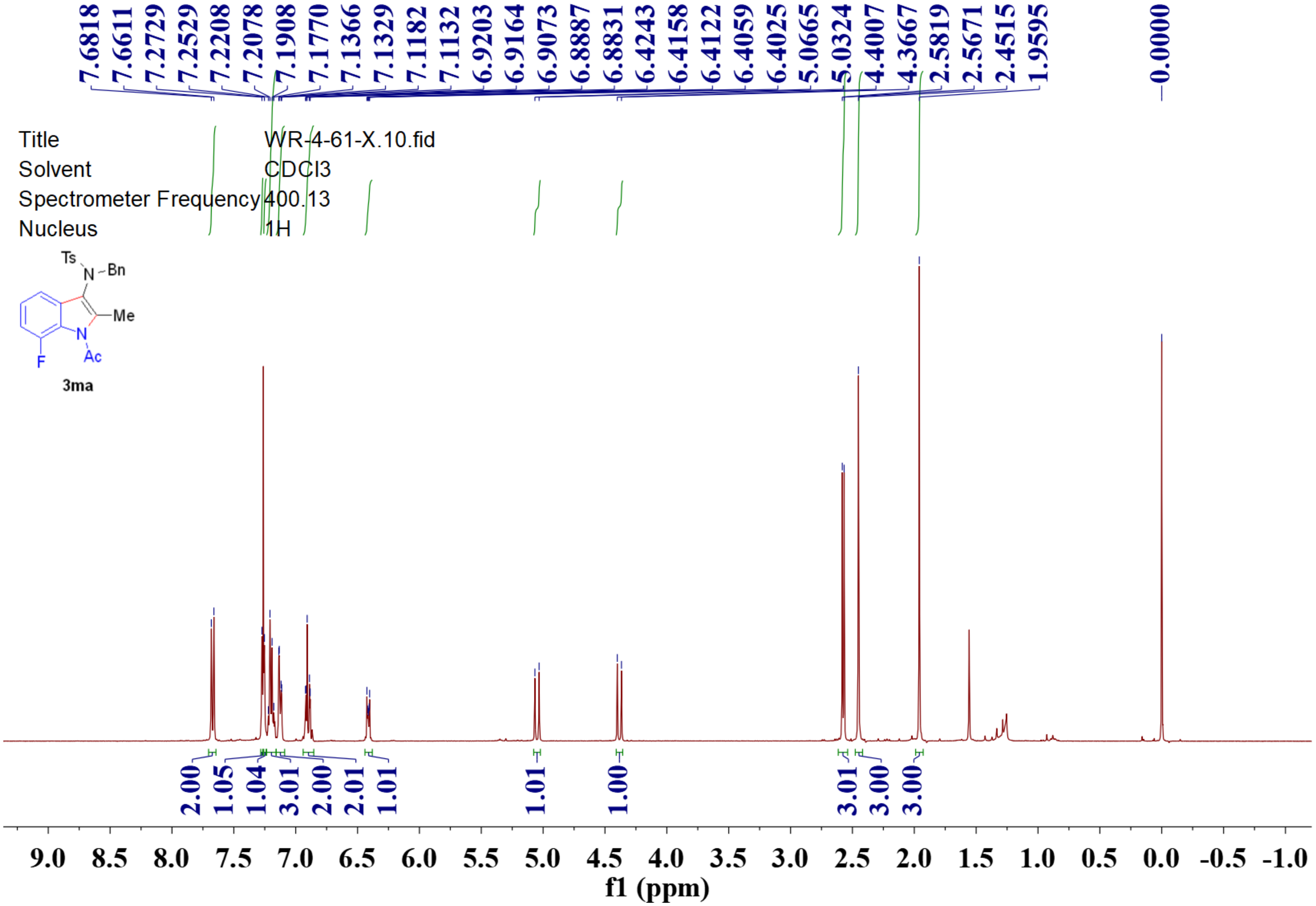
7.6818
7.6611
7.2729
7.2529
7.2208
7.2078
7.1908
7.1770
7.1366
7.1329
7.1182
7.1132
6.9203
6.9164
6.9073
6.8887
6.8831
6.4243
6.4158
6.4122
6.4059
6.4025
5.0665
5.0324
4.4007
4.3667
2.5819
2.5671
2.4515
1.9595
0.0000
Title WR-4-61-X.10.fid
Solvent CDCl3
Spectrometer Frequency 400.13
Nucleus 1H
Ts
N
Bn
Me
N
F
Ac
3ma
2.00
1.05
1.04
3.01
2.00
2.01
1.01
1.01
1.00
3.01
3.00
3.00
9.0
8.5
8.0
7.5
7.0
6.5
6.0
5.5
5.0
4.5
4.0
3.5
3.0
2.5
2.0
1.5
1.0
0.5
0.0
-0.5
-1.0
f1 (ppm)

Title	WR-4-61-C.10.fid
Solvent	CDCl3
Spectrometer Frequency	100.62
Nucleus	13C

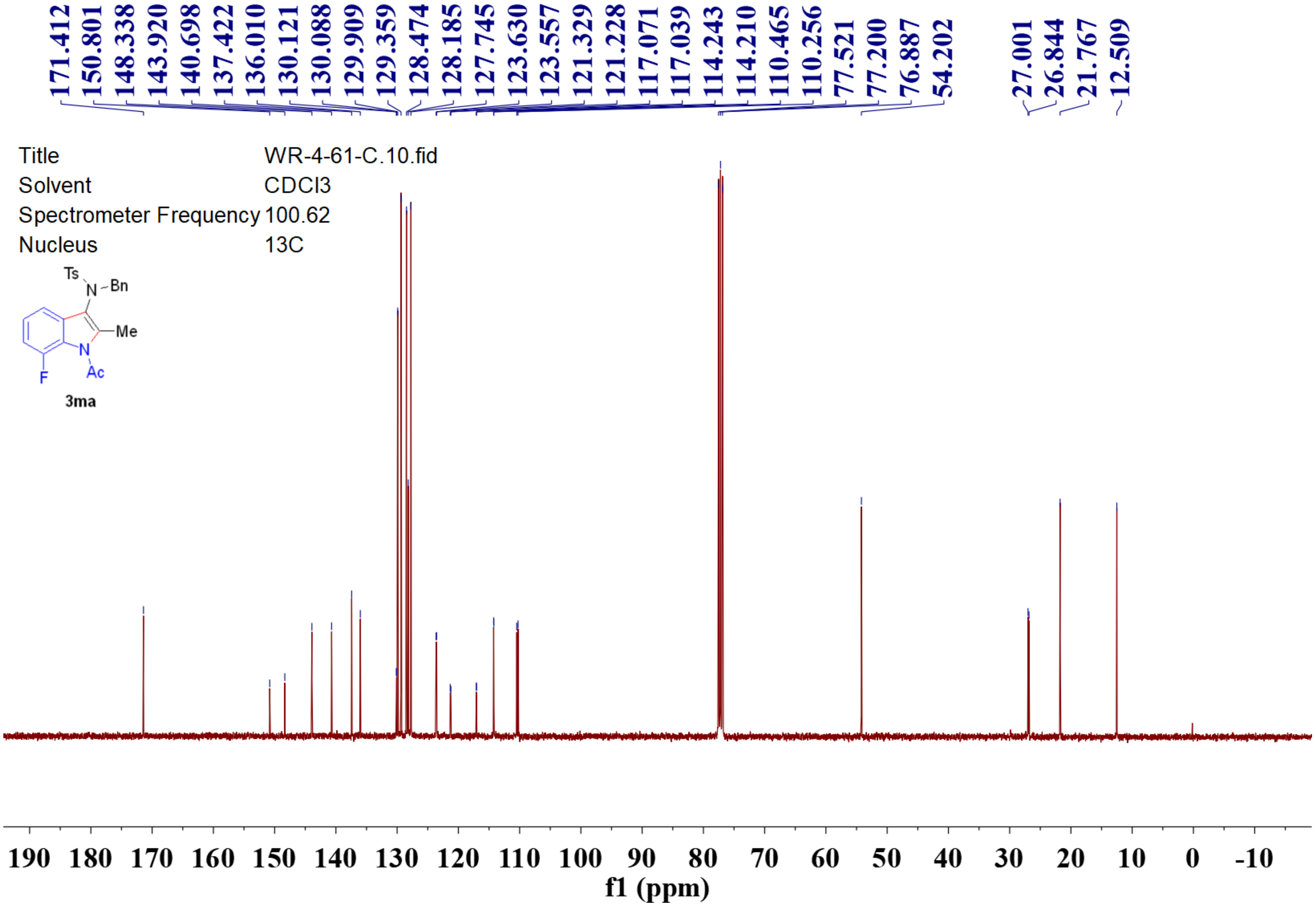

Title WR-4-61-F.10.fid
Solvent CDCl3
Spectrometer Frequency 376.46
Nucleus 19F

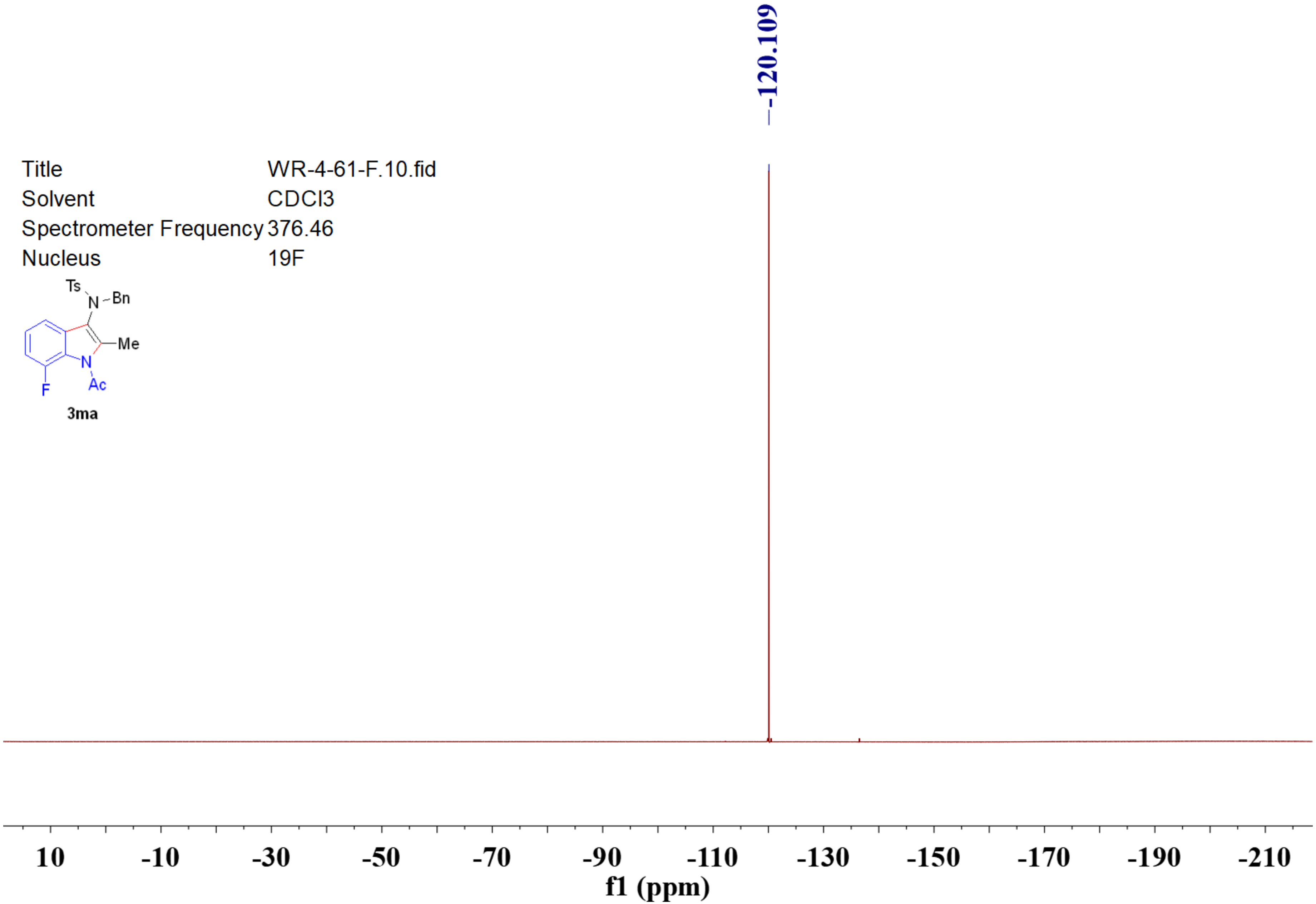

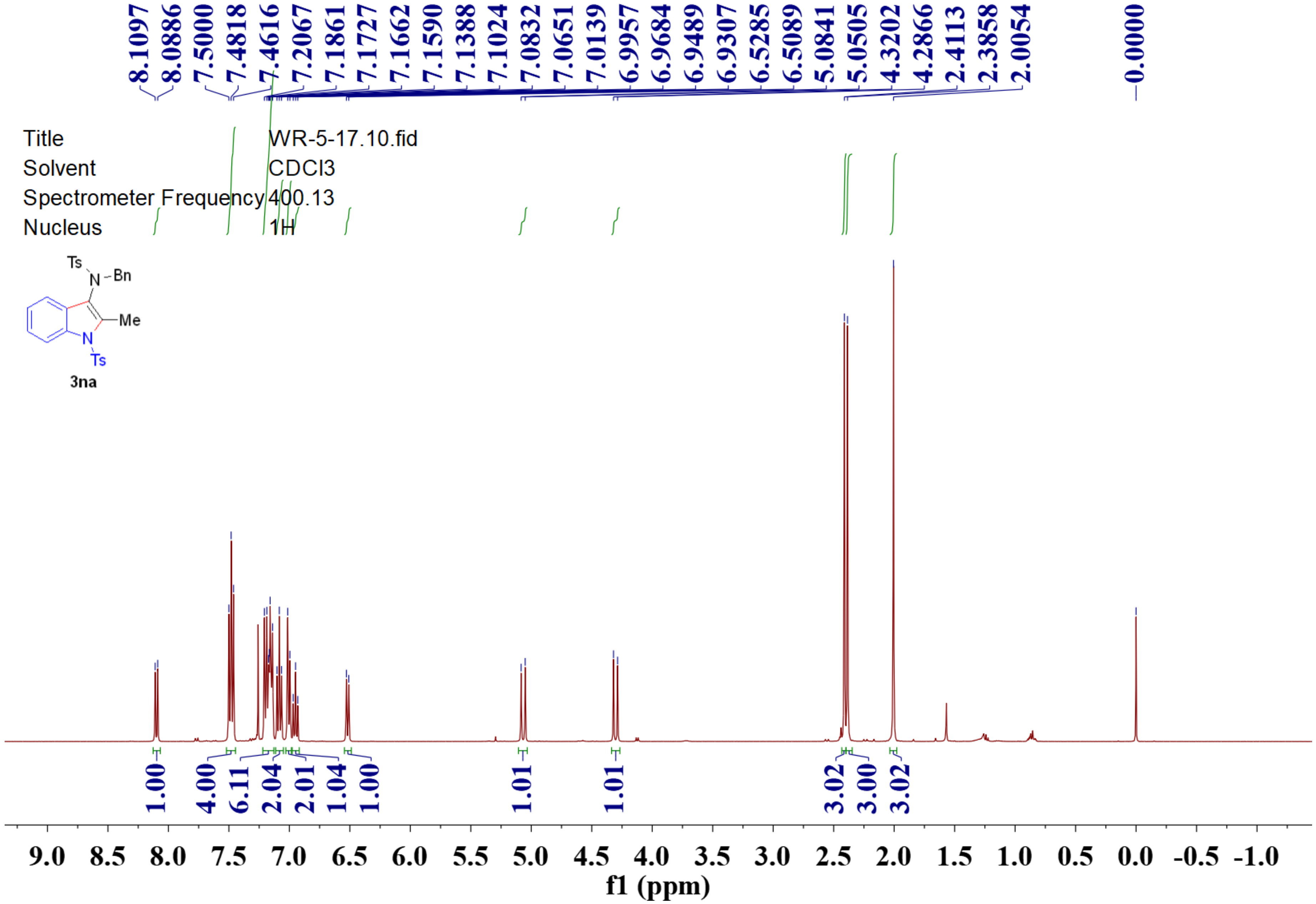
8.1097
8.0886
7.5000
7.4818
7.4616
7.2067
7.1861
7.1727
7.1662
7.1590
7.1388
7.1024
7.0832
7.0651
7.0139
6.9957
6.9684
6.9489
6.9307
6.5285
6.5089
5.0841
5.0505
4.3202
4.2866
2.4113
2.3858
2.0054
0.0000
Title
WR-5-17.10.fid
Solvent
CDCl3
Spectrometer Frequency
400.13
Nucleus
1H
Ts
N
Bn
Me
N
Ts
3na
1.00
4.00
6.11
2.04
2.01
1.04
1.00
1.01
1.01
3.02
3.00
3.02
9.0
8.5
8.0
7.5
7.0
6.5
6.0
5.5
5.0
4.5
4.0
3.5
3.0
2.5
2.0
1.5
1.0
0.5
0.0
-0.5
-1.0
f1 (ppm)

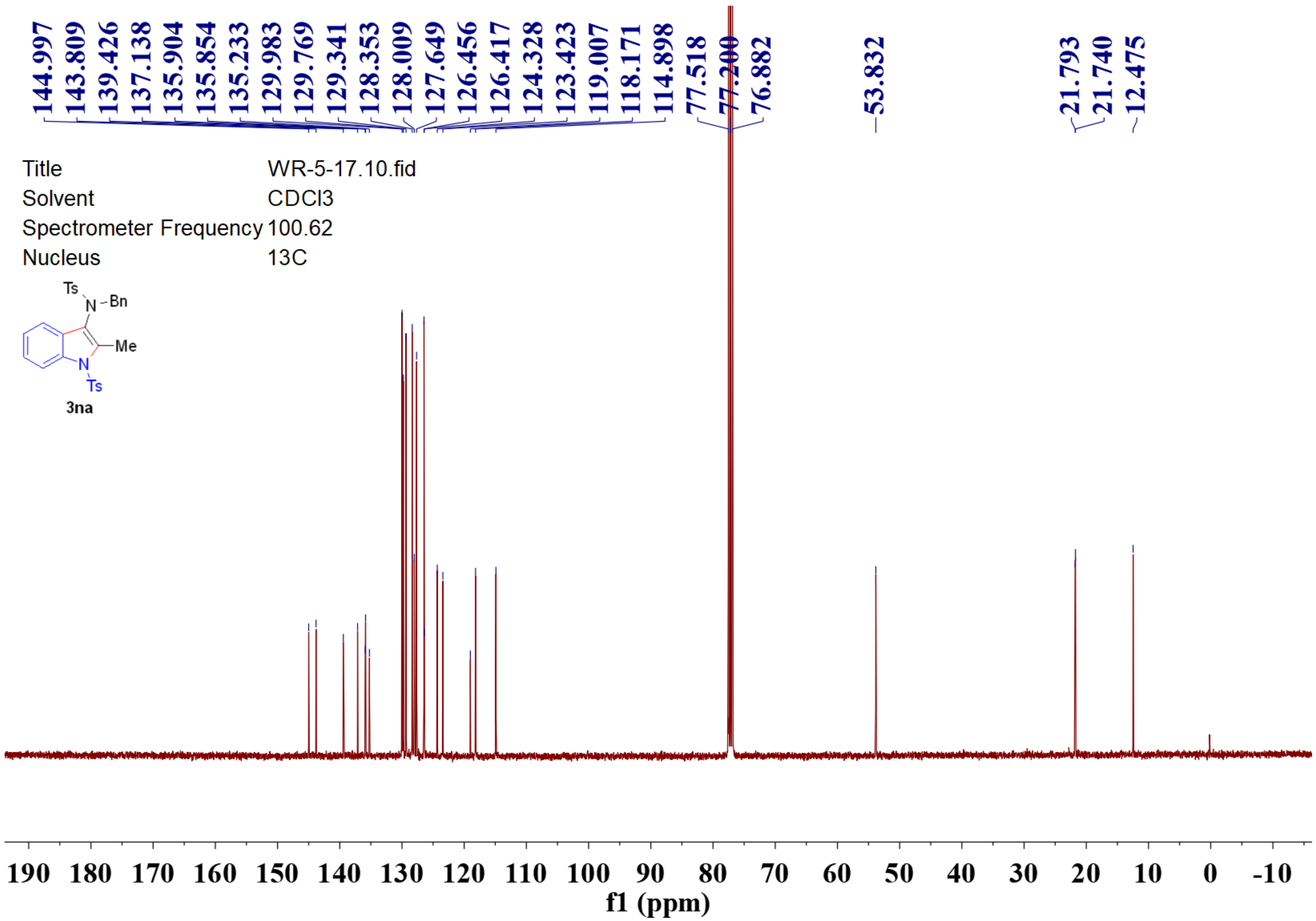
144.997
143.809
139.426
137.138
135.904
135.854
135.233
129.983
129.769
129.341
128.353
128.009
127.649
126.456
126.417
124.328
123.423
119.007
118.171
114.898
77.518
77.200
76.882
53.832
21.793
21.740
12.475
Title WR-5-17.10.fid
Solvent CDCl3
Spectrometer Frequency 100.62
Nucleus 13C
Ts
N
Bn
Me
N
Ts
3na
190 180 170 160 150 140 130 120 110 100 90 80 70 60 50 40 30 20 10 0 -10
f1 (ppm)

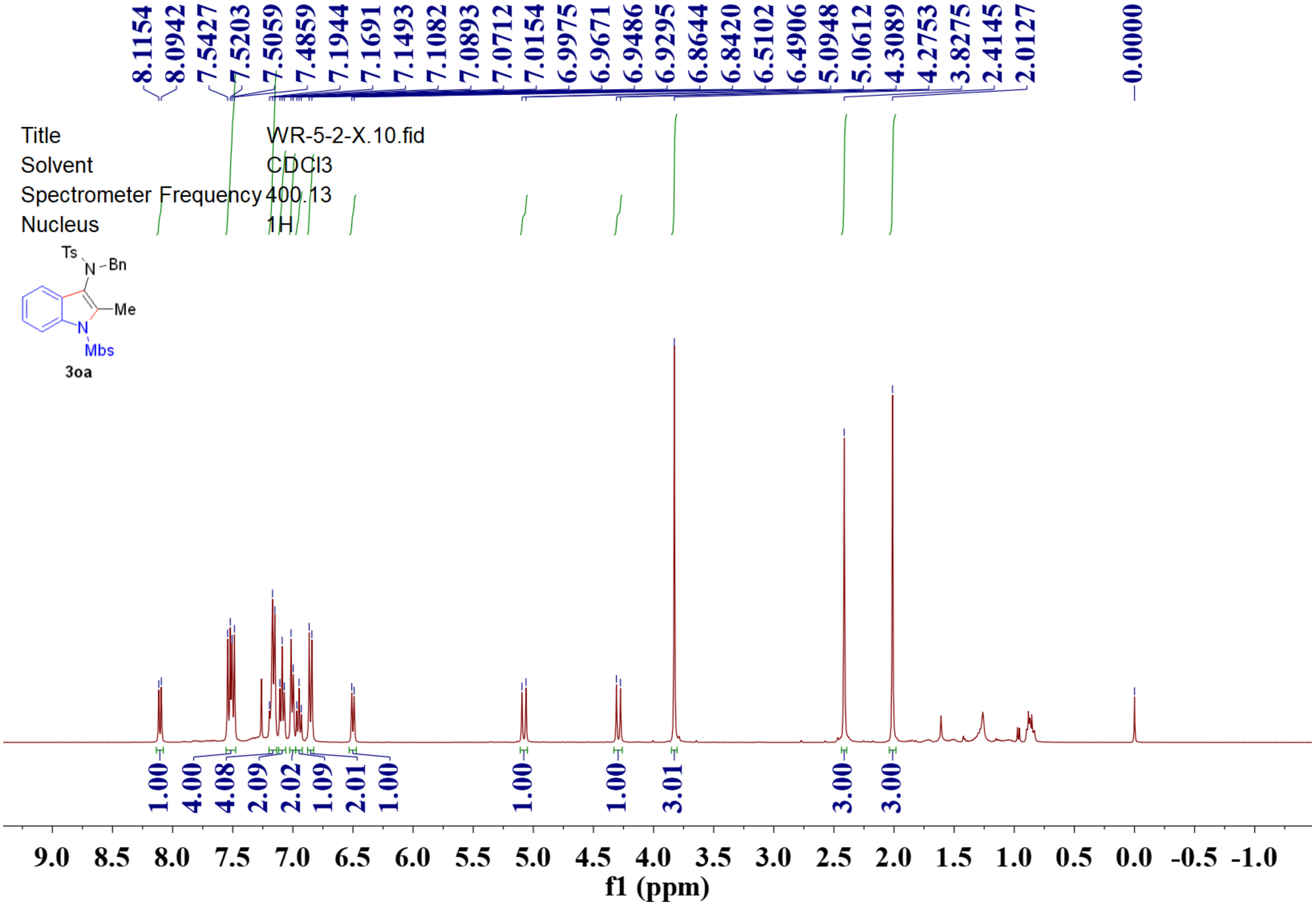
8.1154 8.0942 7.5427 7.5203 7.5059 7.4859 7.1944 7.1691 7.1493 7.1082 7.0893 7.0712 7.0154 6.9975 6.9671 6.9486 6.9295 6.8644 6.8420 6.5102 6.4906 5.0948 5.0612 4.3089 4.2753 3.8275 2.4145 2.0127
-0.0000
Title WR-5-2-X.10.fid
Solvent CDCl3
Spectrometer Frequency 400.13
Nucleus 1H
Ts
N
Bn
Me
N
Mbs
3oa
1.00 4.00 4.08 2.09 2.02 1.09 2.01 1.00
1.00
1.00
3.01
3.00
3.00
9.0 8.5 8.0 7.5 7.0 6.5 6.0 5.5 5.0 4.5 4.0 3.5 3.0 2.5 2.0 1.5 1.0 0.5 0.0 -0.5 -1.0
f1 (ppm)

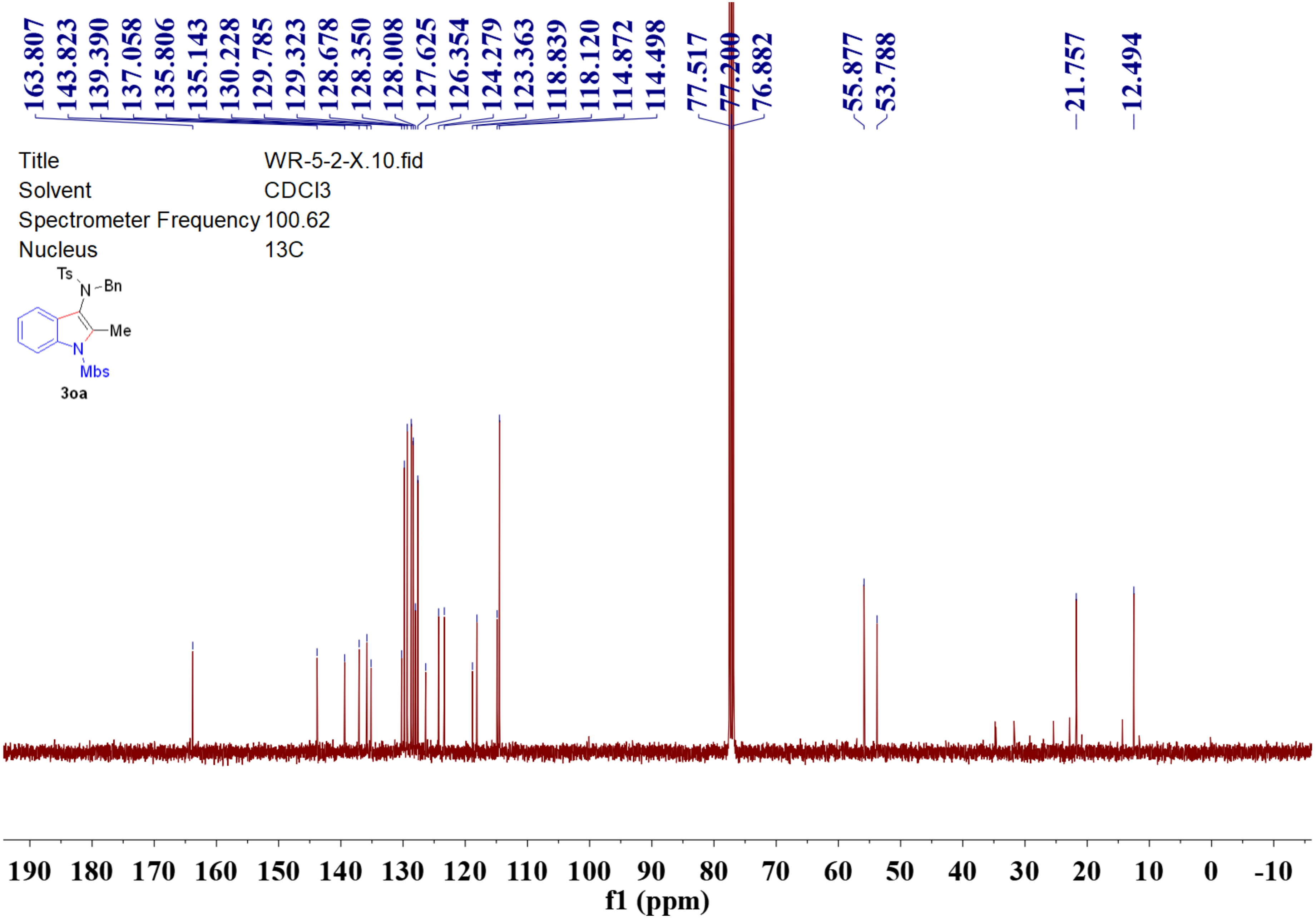

163.807
143.823
139.390
137.058
135.806
135.143
130.228
129.785
129.323
128.678
128.350
128.008
127.625
126.354
124.279
123.363
118.839
118.120
114.872
114.498
77.517
77.200
76.882
55.877
53.788
21.757
12.494
Title WR-5-2-X.10.fid
Solvent CDCl3
Spectrometer Frequency 100.62
Nucleus 13C
Ts
N
Bn
Me
N
Mbs
3oa
190 180 170 160 150 140 130 120 110 100 90 80 70 60 50 40 30 20 10 0 -10
f1 (ppm)

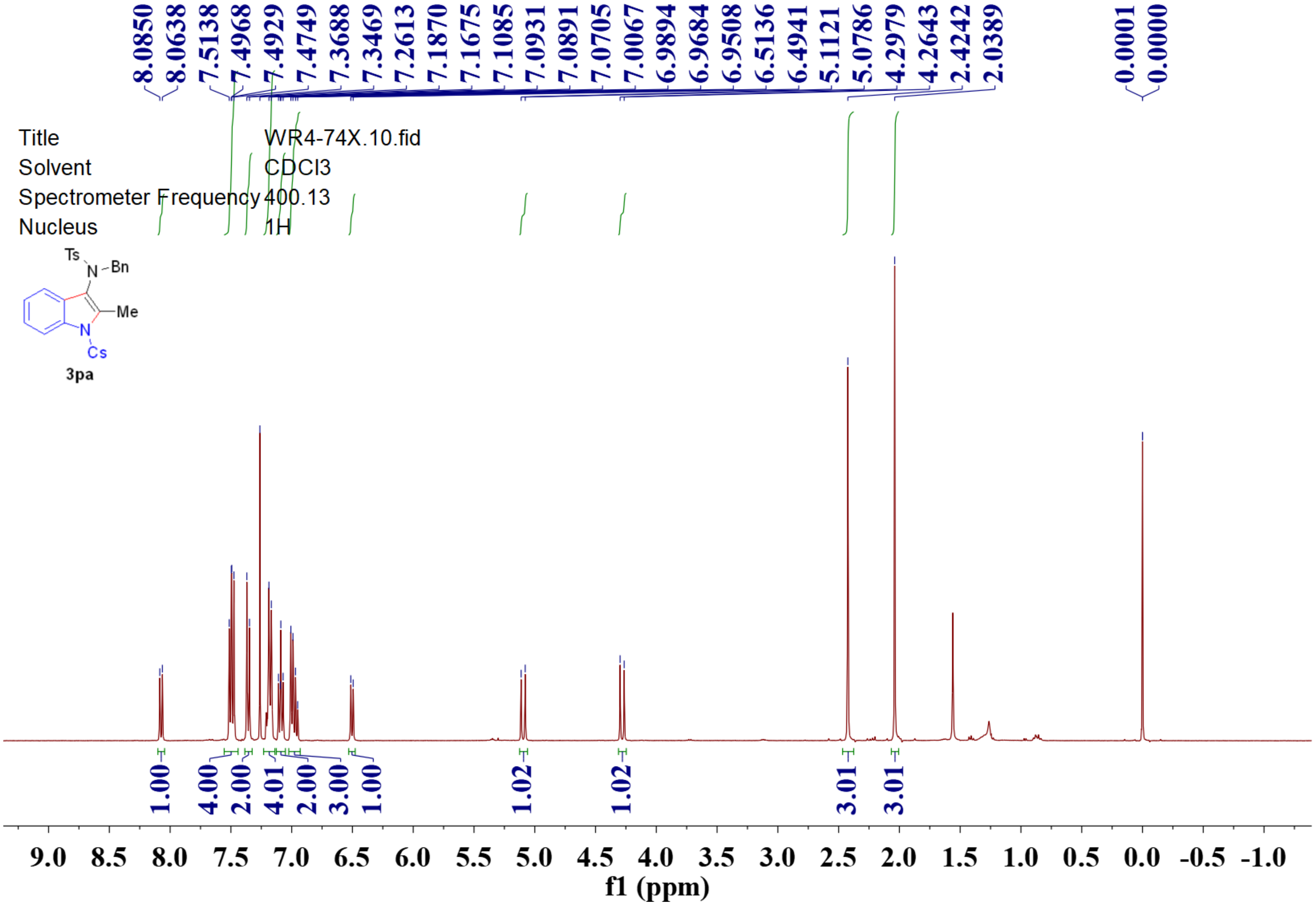
8.0850
8.0638
7.5138
7.4968
7.4929
7.4749
7.3688
7.3469
7.2613
7.1870
7.1675
7.1085
7.0931
7.0891
7.0705
7.0067
6.9894
6.9684
6.9508
6.5136
6.4941
5.1121
5.0786
4.2979
4.2643
2.4242
2.0389
0.0001
0.0000
Title WR4-74X.10.fid
Solvent CDCl3
Spectrometer Frequency 400.13
Nucleus 1H
Ts
N
Bn
Me
N
Cs
3pa
1.00
4.00
2.00
4.01
2.00
3.00
1.00
1.02
1.02
3.01
3.01
9.0 8.5 8.0 7.5 7.0 6.5 6.0 5.5 5.0 4.5 4.0 3.5 3.0 2.5 2.0 1.5 1.0 0.5 0.0 -0.5 -1.0
f1 (ppm)

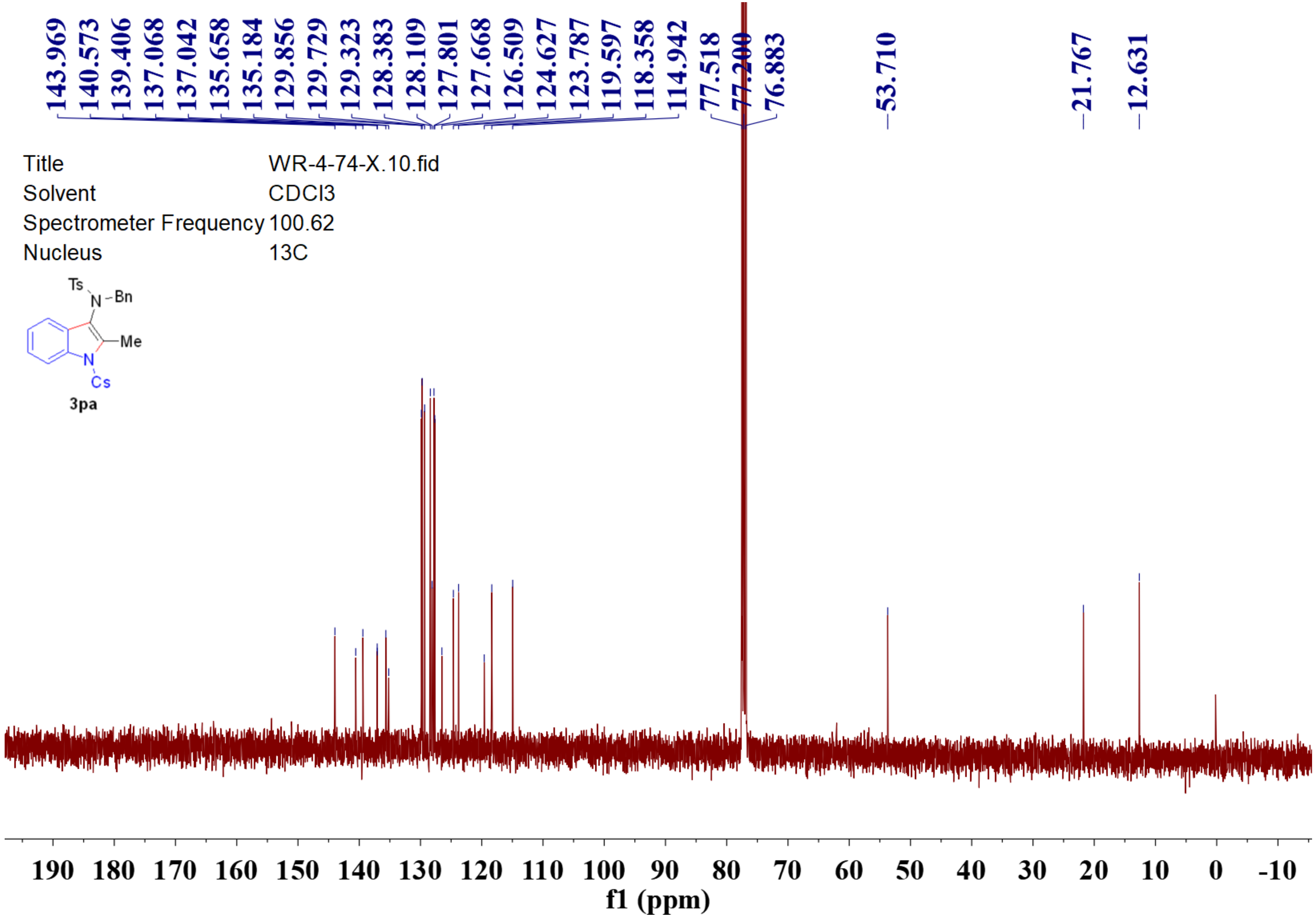
143.969
140.573
139.406
137.068
137.042
135.658
135.184
129.856
129.729
129.323
128.383
128.109
127.801
127.668
126.509
124.627
123.787
119.597
118.358
114.942
77.518
77.200
76.883
53.710
21.767
12.631
Title WR-4-74-X.10.fid
Solvent CDCl3
Spectrometer Frequency 100.62
Nucleus 13C
Ts
N
Bn
Me
N
Cs
3pa
190 180 170 160 150 140 130 120 110 100 90 80 70 60 50 40 30 20 10 0 -10
f1 (ppm)

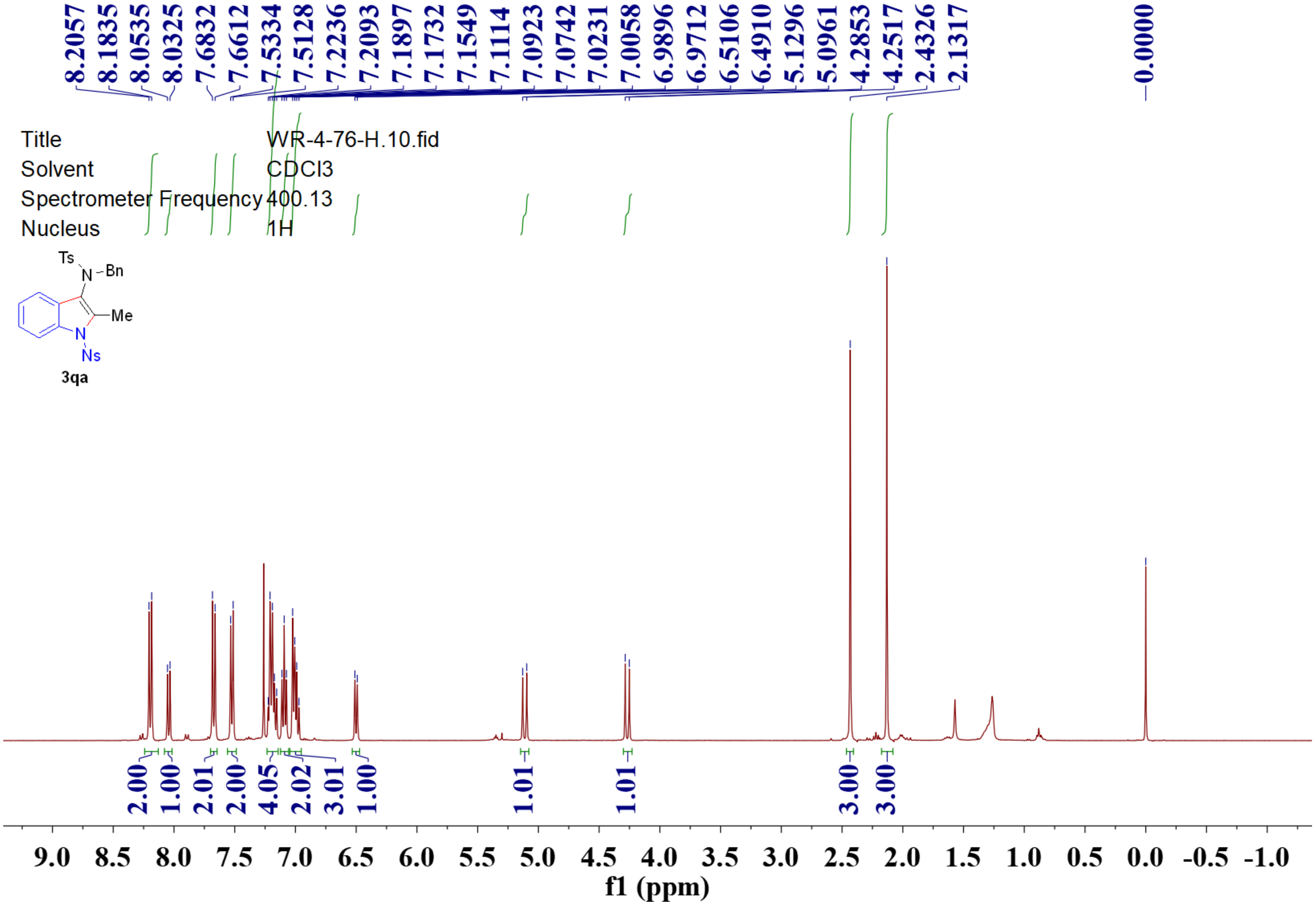

8.2057
8.1835
8.0535
8.0325
7.6832
7.6612
7.5334
7.5128
7.2236
7.2093
7.1897
7.1732
7.1549
7.1114
7.0923
7.0742
7.0231
7.0058
6.9896
6.9712
6.5106
6.4910
5.1296
5.0961
4.2853
4.2517
2.4326
2.1317
-0.0000
Title WR-4-76-H.10.fid
Solvent CDCl3
Spectrometer Frequency 400.13
Nucleus 1H
2.00
1.00
2.01
2.00
4.05
2.02
3.01
1.00
1.01
1.01
3.00
3.00
9.0 8.5 8.0 7.5 7.0 6.5 6.0 5.5 5.0 4.5 4.0 3.5 3.0 2.5 2.0 1.5 1.0 0.5 0.0 -0.5 -1.0
f1 (ppm)
Ts
N
Bn
Me
N
Ns

3qa

Title WR-4-76-C.10.fid
Solvent CDCl3
Spectrometer Frequency 100.62
Nucleus 13C

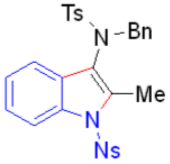

3qa

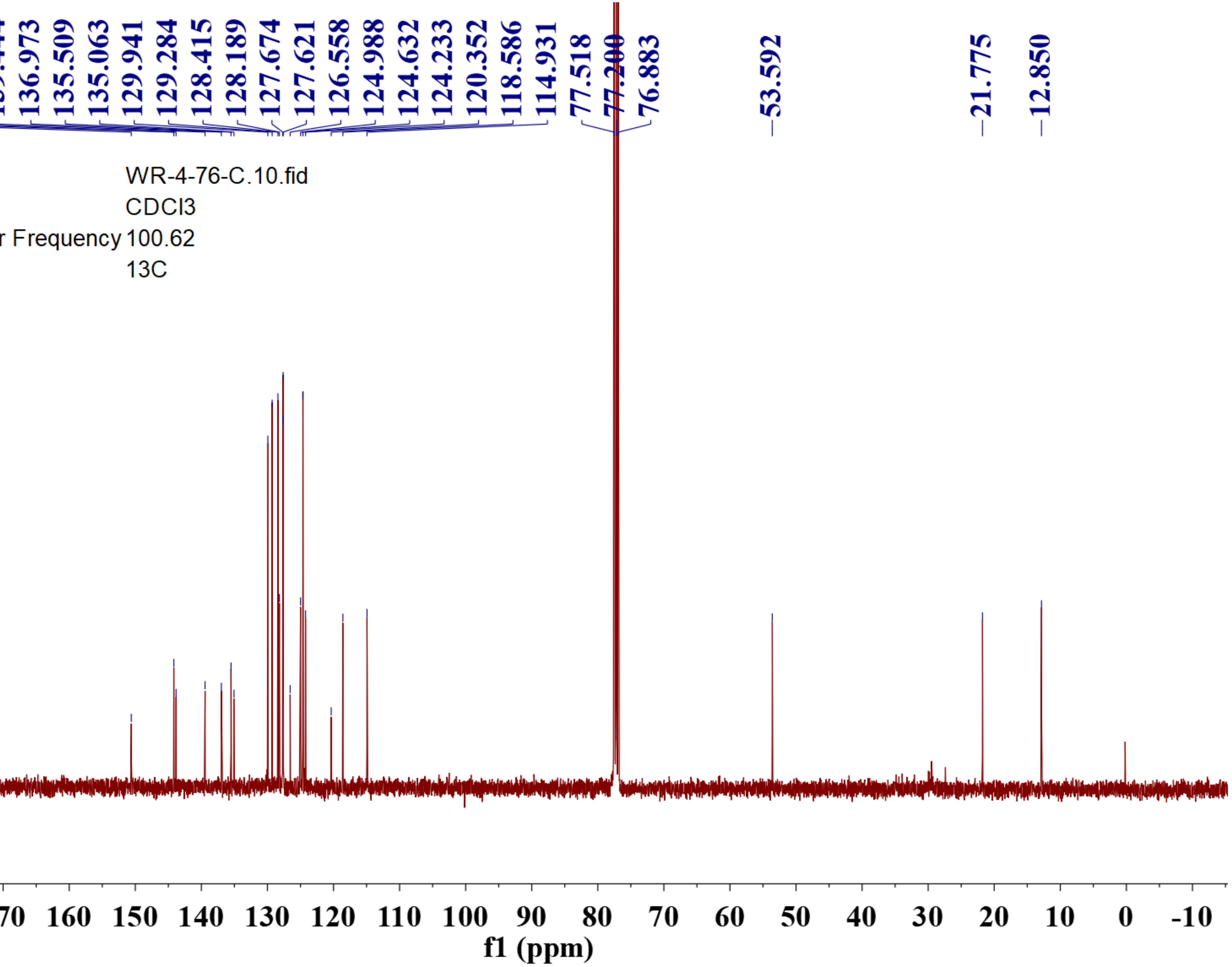

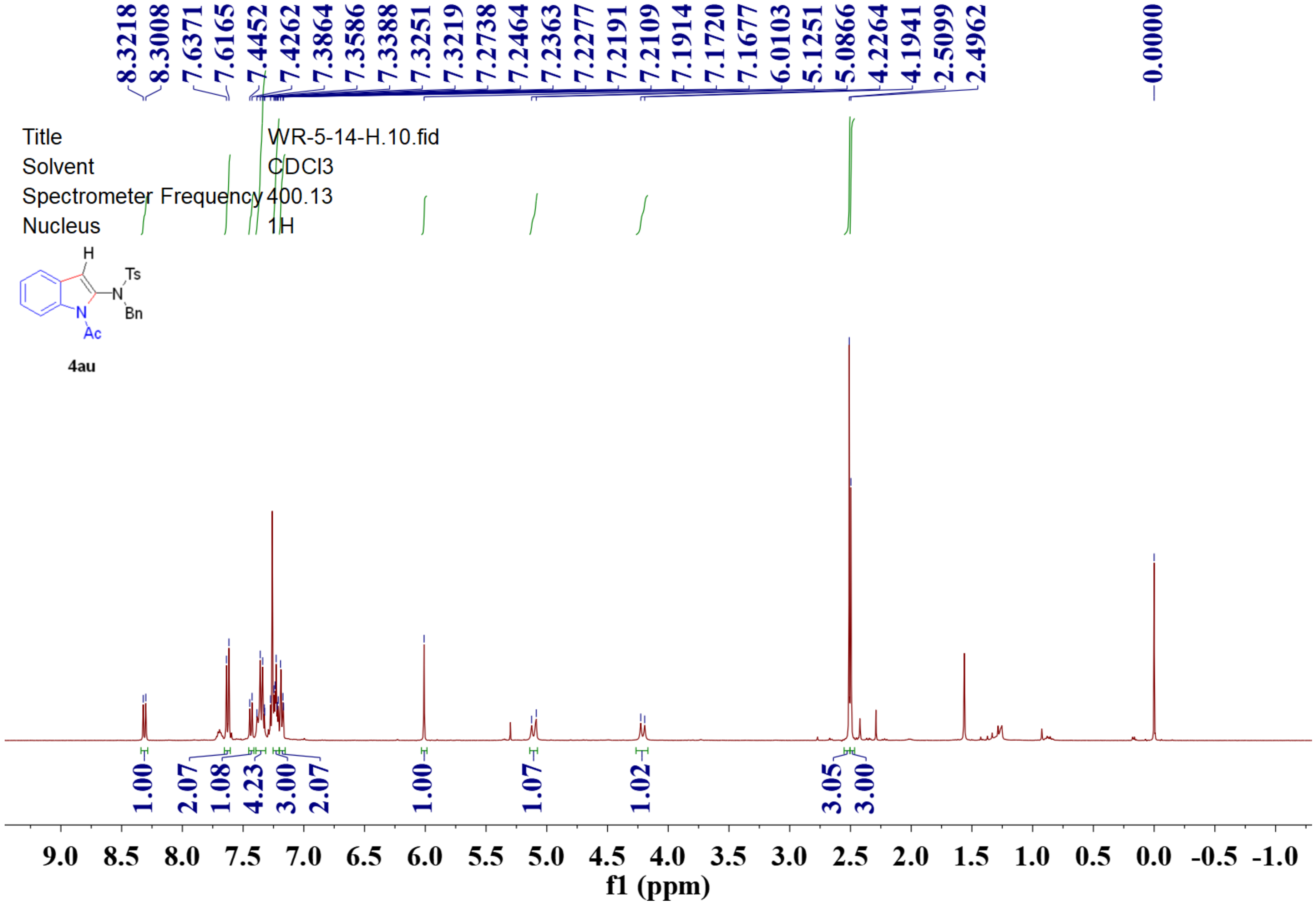
8.3218
8.3008
7.6371
7.6165
7.4452
7.4262
7.3864
7.3586
7.3388
7.3251
7.3219
7.2738
7.2464
7.2363
7.2277
7.2191
7.2109
7.1914
7.1720
7.1677
6.0103
5.1251
5.0866
4.2264
4.1941
2.5099
2.4962
-0.0000
Title WR-5-14-H.10.fid
Solvent CDCl3
Spectrometer Frequency 400.13
Nucleus 1H
H
Ts
N
N
Bn
Ac
4au
1.00
2.07
1.08
4.23
3.00
2.07
1.00
1.07
1.02
3.05
3.00
9.0 8.5 8.0 7.5 7.0 6.5 6.0 5.5 5.0 4.5 4.0 3.5 3.0 2.5 2.0 1.5 1.0 0.5 0.0 -0.5 -1.0
f1 (ppm)

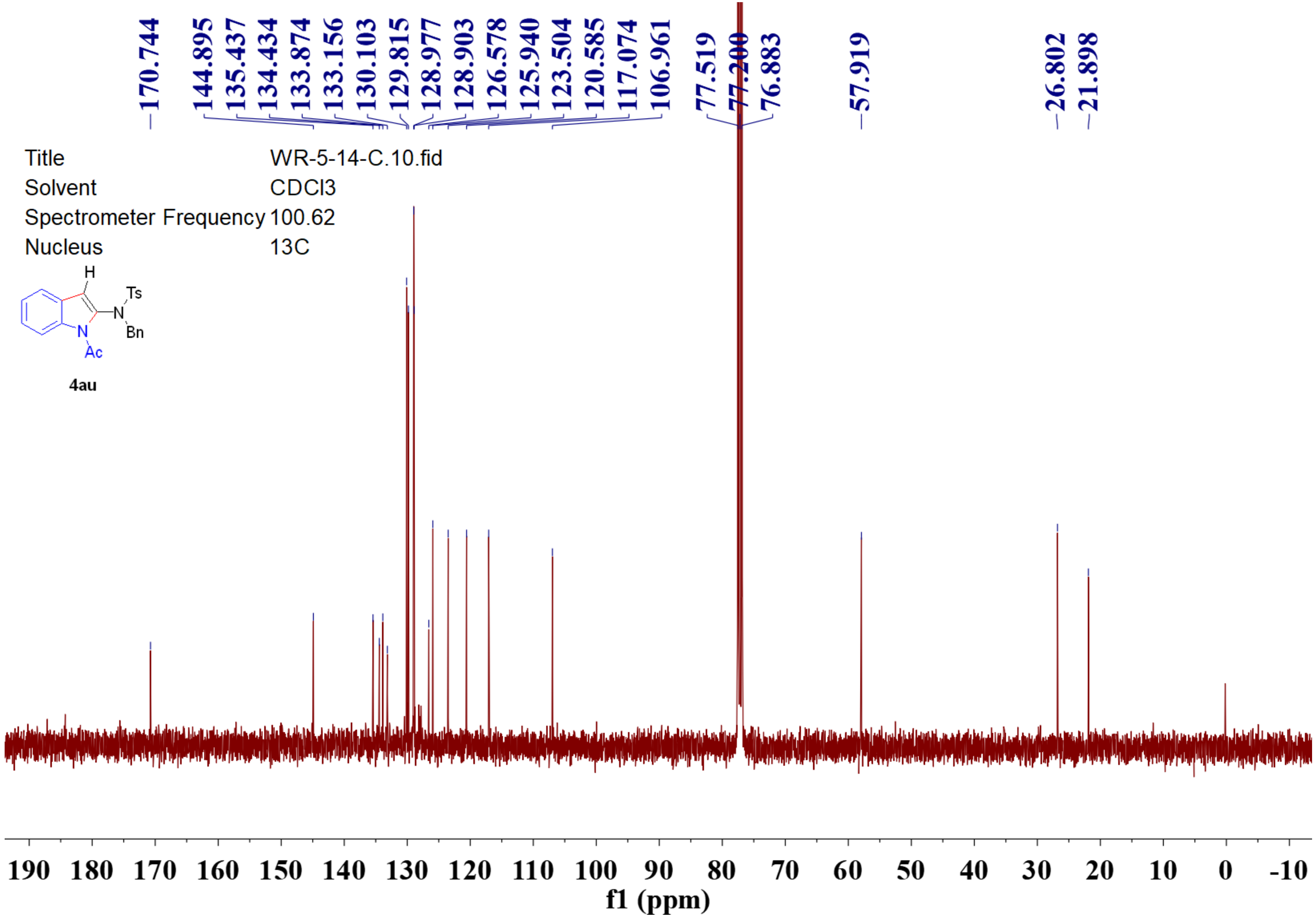
170.744
144.895
135.437
134.434
133.874
133.156
130.103
129.815
128.977
128.903
126.578
125.940
123.504
120.585
117.074
106.961
77.519
77.200
76.883
57.919
26.802
21.898
Title WR-5-14-C.10.fid
Solvent CDCl3
Spectrometer Frequency 100.62
Nucleus 13C
H
Ts
N
N
Bn
Ac
4au
190 180 170 160 150 140 130 120 110 100 90 80 70 60 50 40 30 20 10 0 -10
f1 (ppm)

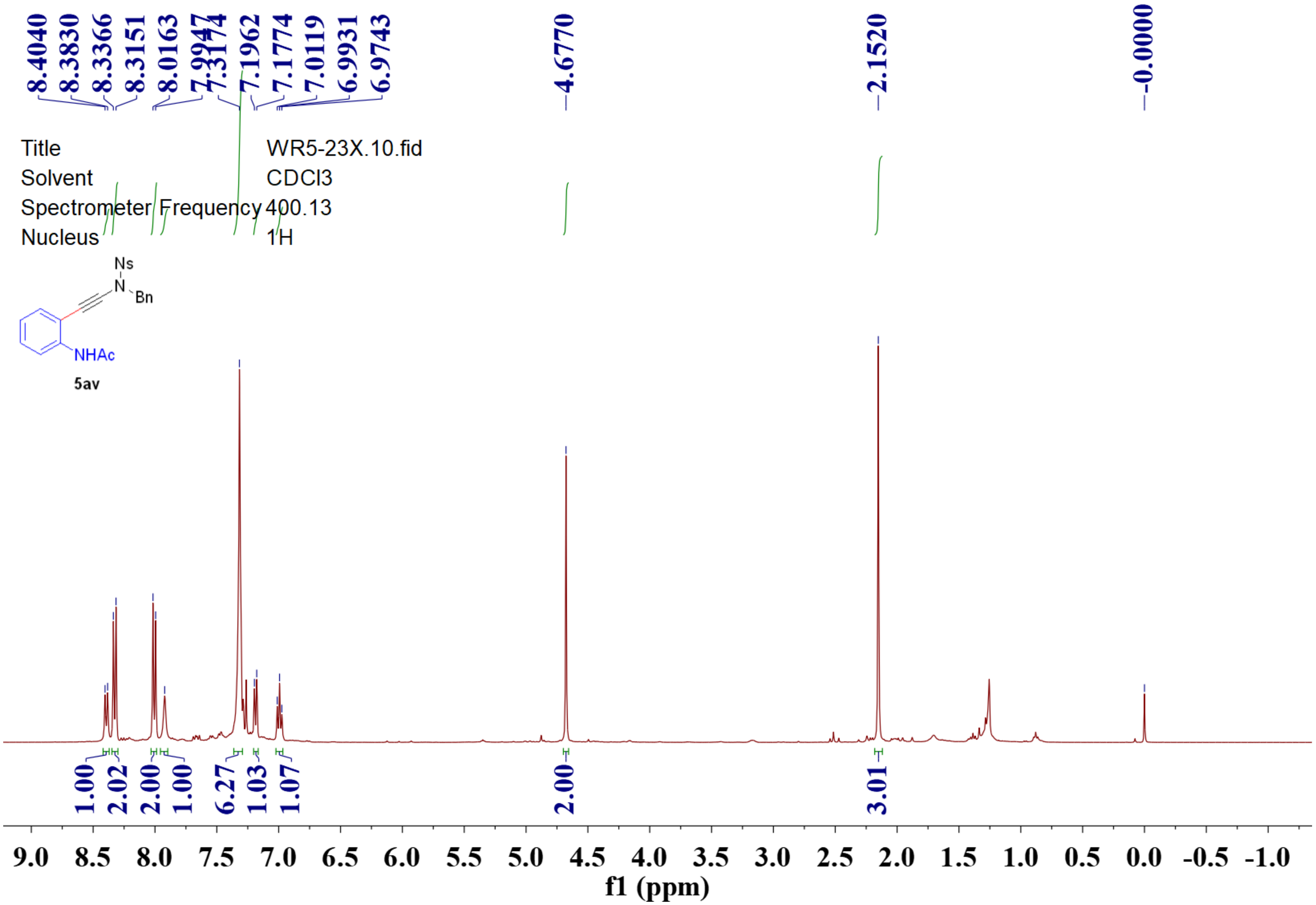
8.4040
8.3830
8.3366
8.3151
8.0163
7.9947
7.3174
7.1962
7.1774
7.0119
6.9931
6.9743
4.6770
2.1520
-0.0000
Title WR5-23X.10.fid
Solvent CDCl3
Spectrometer Frequency 400.13
Nucleus 1H
Ns
N
Bn
NHAc
5av
1.00
2.02
2.00
1.00
6.27
1.03
1.07
2.00
3.01
9.0 8.5 8.0 7.5 7.0 6.5 6.0 5.5 5.0 4.5 4.0 3.5 3.0 2.5 2.0 1.5 1.0 0.5 0.0 -0.5 -1.0
f1 (ppm)

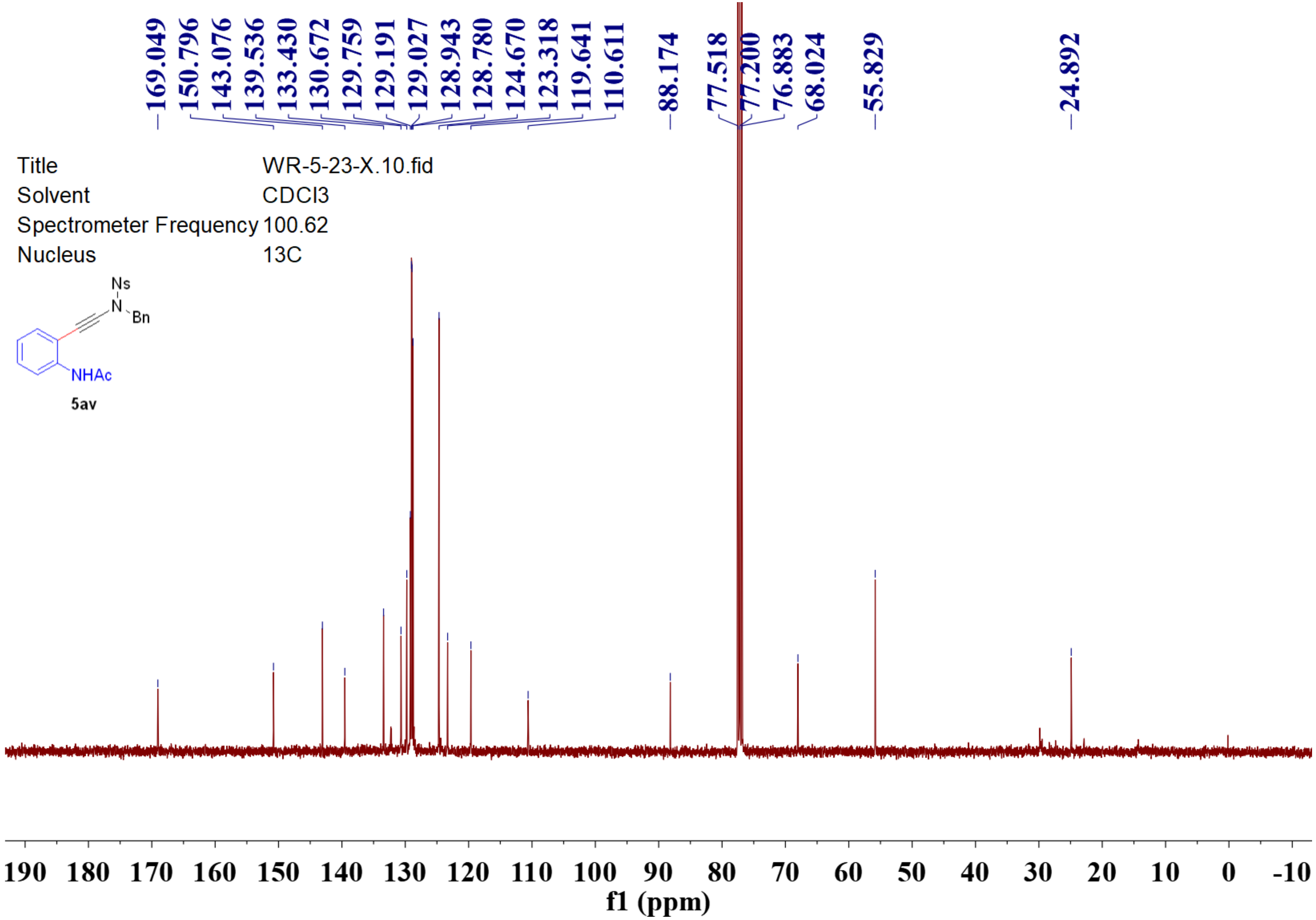
169.049
150.796
143.076
139.536
133.430
130.672
129.759
129.191
129.027
128.943
128.780
124.670
123.318
119.641
110.611
88.174
77.518
77.200
76.883
68.024
55.829
24.892
Title WR-5-23-X.10.fid
Solvent CDCl3
Spectrometer Frequency 100.62
Nucleus 13C
Ns
N
Bn
NHAc
5av
190 180 170 160 150 140 130 120 110 100 90 80 70 60 50 40 30 20 10 0 -10
f1 (ppm)

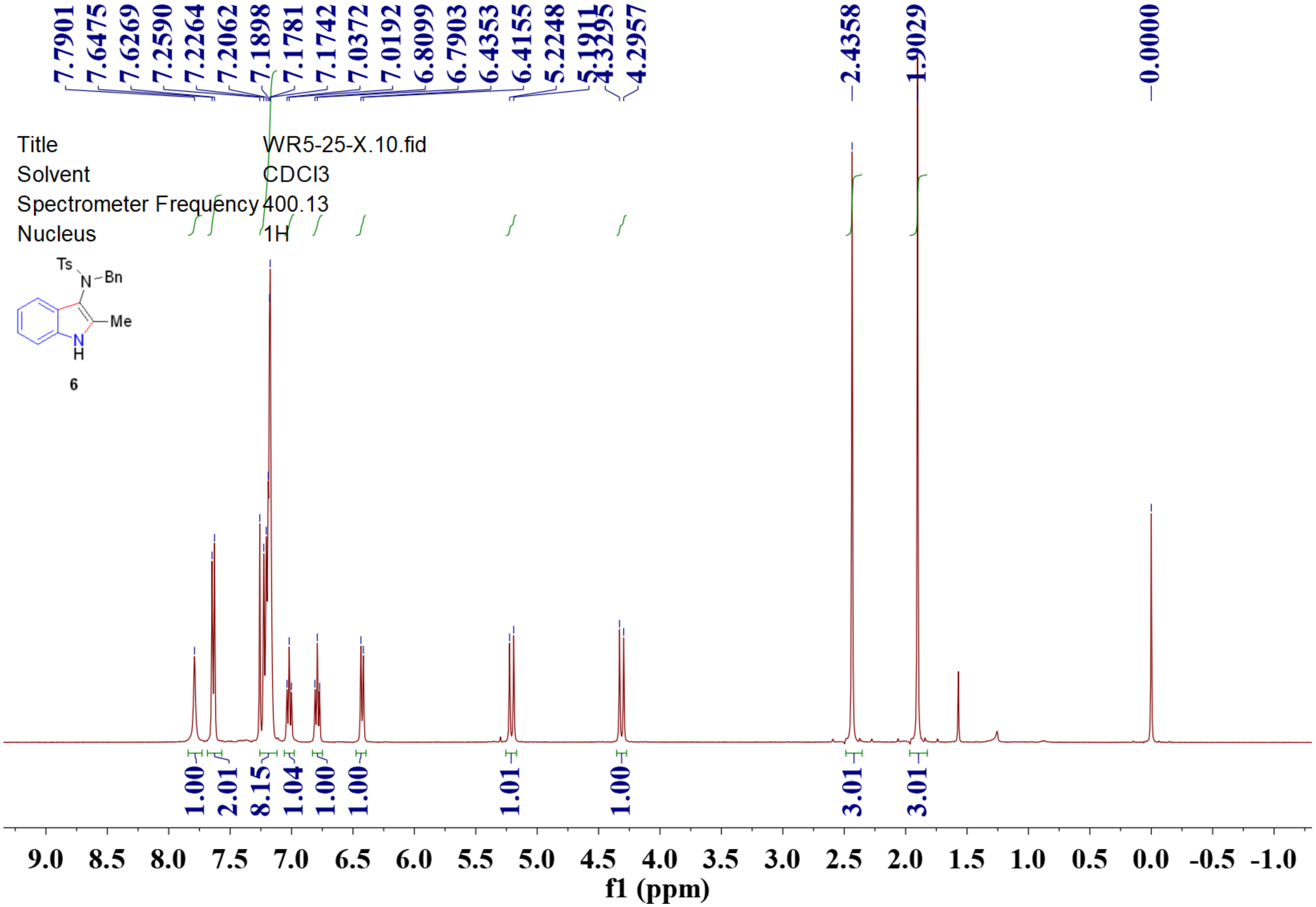
7.7901
7.6475
7.6269
7.2590
7.2264
7.2062
7.1898
7.1781
7.1742
7.0372
7.0192
6.8099
6.7903
6.4353
6.4155
5.2248
5.1911
4.3295
4.2957
2.4358
1.9029
0.0000
Title WR5-25-X.10.fid
Solvent CDCl3
Spectrometer Frequency 400.13
Nucleus 1H
Ts
N
Bn
Me
N
H
6
1.00
2.01
8.15
1.04
1.00
1.00
1.01
1.00
3.01
3.01
9.0 8.5 8.0 7.5 7.0 6.5 6.0 5.5 5.0 4.5 4.0 3.5 3.0 2.5 2.0 1.5 1.0 0.5 0.0 -0.5 -1.0
f1 (ppm)

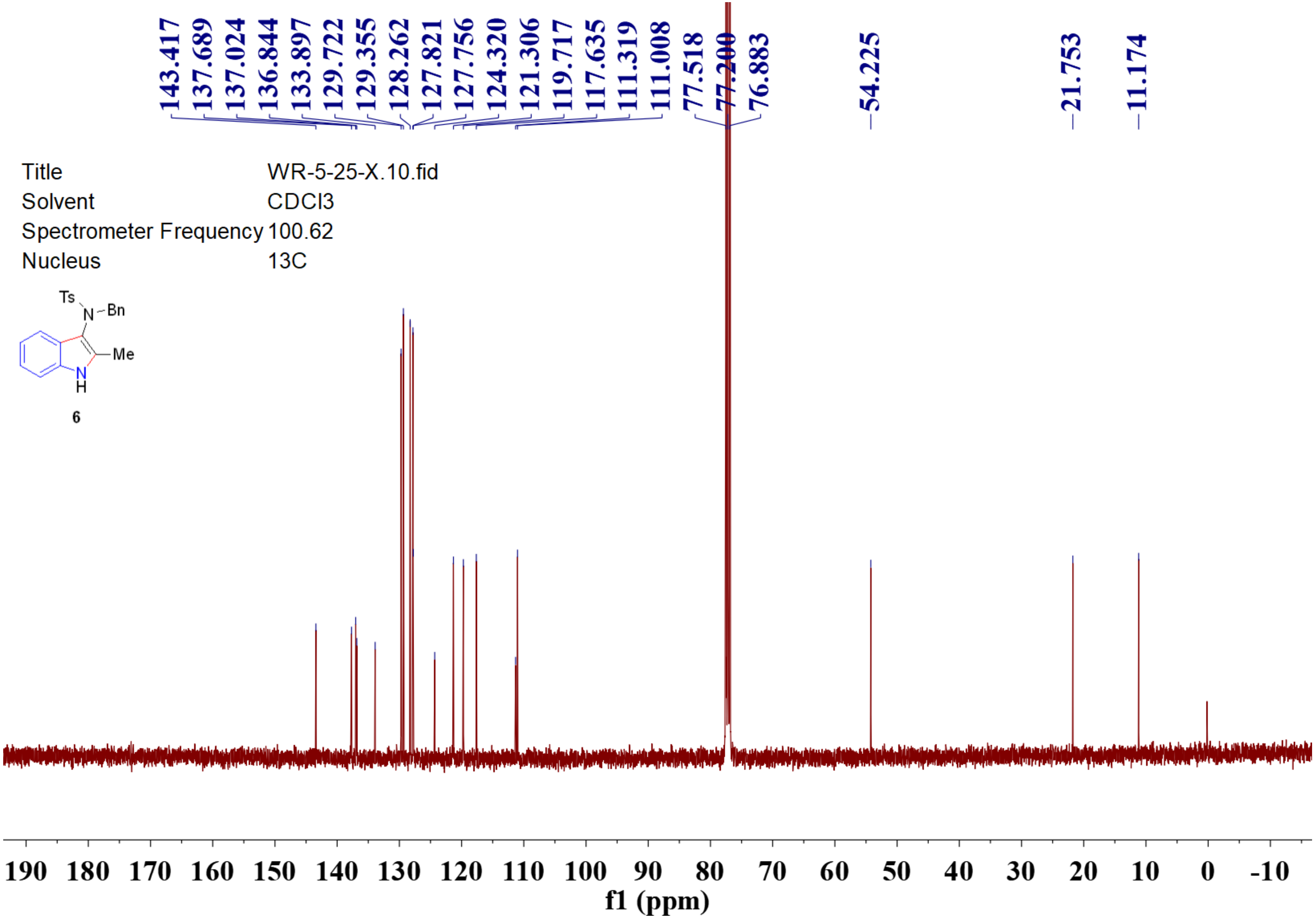
143.417
137.689
137.024
136.844
133.897
129.722
129.355
128.262
127.821
127.756
124.320
121.306
119.717
117.635
111.319
111.008
77.518
77.200
76.883
54.225
21.753
11.174
Title WR-5-25-X.10.fid
Solvent CDCl3
Spectrometer Frequency 100.62
Nucleus 13C
Ts
N
Bn
Me
N
H
6
190 180 170 160 150 140 130 120 110 100 90 80 70 60 50 40 30 20 10 0 -10
f1 (ppm)

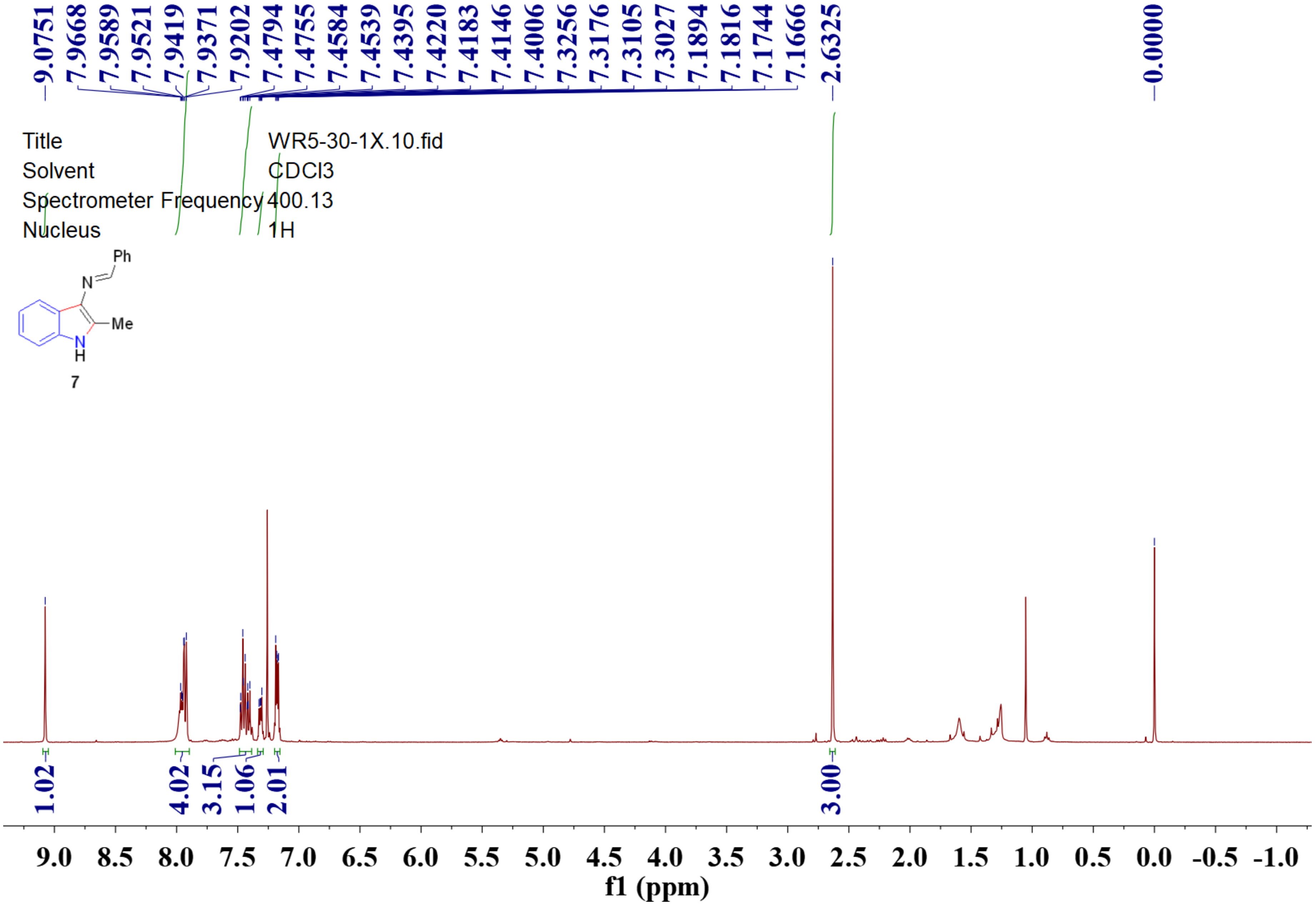

9.0751
7.9668
7.9589
7.9521
7.9419
7.9371
7.9202
7.4794
7.4755
7.4584
7.4539
7.4395
7.4220
7.4183
7.4146
7.4006
7.3256
7.3176
7.3105
7.3027
7.1894
7.1816
7.1744
7.1666
2.6325
0.0000
Title WR5-30-1X.10.fid
Solvent CDCl3
Spectrometer Frequency 400.13
Nucleus 1H
Ph
N
Me
N
H
7
1.02
4.02
3.15
1.06
2.01
3.00
9.0 8.5 8.0 7.5 7.0 6.5 6.0 5.5 5.0 4.5 4.0 3.5 3.0 2.5 2.0 1.5 1.0 0.5 0.0 -0.5 -1.0
f1 (ppm)

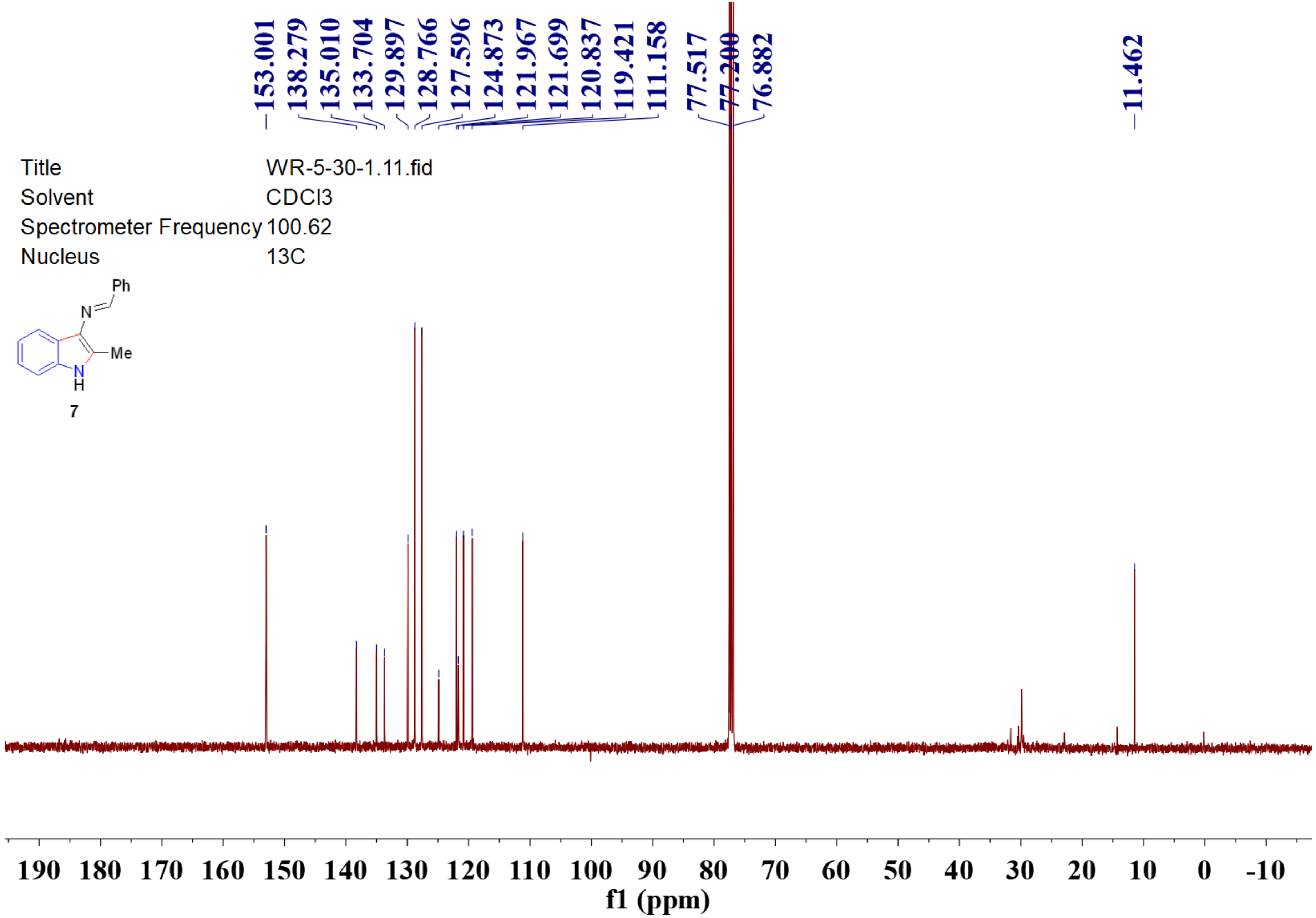
153.001
138.279
135.010
133.704
129.897
128.766
127.596
124.873
121.967
121.699
120.837
119.421
111.158
77.517
77.200
76.882
11.462
Title WR-5-30-1.11.fid
Solvent CDCl3
Spectrometer Frequency 100.62
Nucleus 13C
Ph
N
Me
N
H
7
190 180 170 160 150 140 130 120 110 100 90 80 70 60 50 40 30 20 10 0 -10
f1 (ppm)
